# Different binding motifs of the celiac disease-associated HLA molecules DQ2.5, DQ2.2, and DQ7.5 revealed by relative quantitative proteomics of endogenous peptide repertoires

**DOI:** 10.1007/s00251-014-0819-9

**Published:** 2014-12-12

**Authors:** Elin Bergseng, Siri Dørum, Magnus Ø. Arntzen, Morten Nielsen, Ståle Nygård, Søren Buus, Gustavo A. de Souza, Ludvig M. Sollid

**Affiliations:** 1 Centre for Immune Regulation, Department of Immunology, University of Oslo and Oslo University Hospital-Rikshospitalet, Oslo, Norway; 2 The Biotechnology Centre of Oslo, University of Oslo, Oslo, Norway; 3 Center for Biological Sequence Analysis, Technical University of Denmark, Lyngby, Denmark; 4 Instituto de Investigaciones Biotecnológicas, Universidad Nacional de San Martín, San Martín Buenos Aires, Argentina; 5 Bioinformatics Core Facility, Oslo University Hospital and University of Oslo, Oslo, Norway; 6 Laboratory of Experimental Immunology, University of Copenhagen, Copenhagen, Denmark; 7 Proteomics Core Facility, Oslo University Hospital-Rikshospitalet, Oslo, Norway

**Keywords:** Antigen presentation/processing, Binding motif, Celiac disease, Mass spectrometry, MHC

## Abstract

Celiac disease is caused by intolerance to cereal gluten proteins, and HLA-DQ molecules are involved in the disease pathogenesis by presentation of gluten peptides to CD4^+^ T cells. The α- or β-chain sharing HLA molecules DQ2.5, DQ2.2, and DQ7.5 display different risks for the disease. It was recently demonstrated that T cells of DQ2.5 and DQ2.2 patients recognize distinct sets of gluten epitopes, suggesting that these two DQ2 variants select different peptides for display. To explore whether this is the case, we performed a comprehensive comparison of the endogenous self-peptides bound to HLA-DQ molecules of B-lymphoblastoid cell lines. Peptides were eluted from affinity-purified HLA molecules of nine cell lines and subjected to quadrupole orbitrap mass spectrometry and MaxQuant software analysis. Altogether, 12,712 endogenous peptides were identified at very different relative abundances. Hierarchical clustering of normalized quantitative data demonstrated significant differences in repertoires of peptides between the three DQ variant molecules. The neural network-based method, *NNAlign*, was used to identify peptide-binding motifs. The binding motifs of DQ2.5 and DQ7.5 concurred with previously established binding motifs. The binding motif of DQ2.2 was strikingly different from that of DQ2.5 with position P3 being a major anchor having a preference for threonine and serine. This is notable as three recently identified epitopes of gluten recognized by T cells of DQ2.2 celiac patients harbor serine at position P3. This study demonstrates that relative quantitative comparison of endogenous peptides sampled from our protein metabolism by HLA molecules provides clues to understand HLA association with disease.

**Electronic supplementary material:**

The online version of this article (doi:10.1007/s00251-014-0819-9) contains supplementary material, which is available to authorized users.

## Introduction

Major histocompatibility complex (MHC) II molecules display peptides on the surface of professional antigen presenting cells for presentation to CD4^+^ T cells (Germain 1994). The peptides derive from proteolytically processed proteins, either exogenous proteins taken up by endocytosis or from endogenous proteins made for export via the Golgi pathway. This proteolysis is performed by cathepsins and other enzymes present in endosomes. In the same endosomal compartments, the MHC II molecules are loaded with these exogenously derived peptides by exchange of class II-associated invariant chain (CLIP) peptides that have occupied the binding groove since assembly of the MHC II molecules in the endoplasmic reticulum. Peptides that make stable peptide-MHC complexes will accumulate on the cell surface. These peptides are particularly prone to be recognized by T cells as the amount of peptide-MHC complexes matters for T-cell activation (Henrickson et al. 2008).

For many autoimmune diseases, MHC II genes are major genetic susceptibility determinants (Lettre and Rioux 2008). In keeping with this, presentation of antigenic peptides to CD4^+^ T cells is inferred to be central in the pathogenesis of these conditions. Celiac disease, a chronic inflammatory disorder of the small intestine caused by an inappropriate immune response to dietary gluten proteins of wheat, rye, and barley, is one of the diseases with the most marked HLA association. There is a strong HLA association with DQ2.5 (DQA1*05:01, DQB1*02:01) and weaker associations with DQ8 (DQA1*03, DQB1*03:02) and DQ2.2 (DQA1*02:01, DQB1*02:02) (Sollid and Lie 2005). The very few patients, who are not DQ2.5, DQ2.2, or DQ8, are almost all DQ7.5 (DQA1*05, DQB1*03:01) (Karell et al. 2003). Notably, DQ2.5 shares the β-chain with DQ2.2 (with exception of one residue difference at position 135 in the membrane proximal domain) and the α-chain with DQ7.5. HLA-DQ molecules predispose to celiac disease by presenting gluten peptides to CD4^+^ T cells (Lundin et al. 1993). A number of gluten epitopes that are presented to T cells in the context of DQ2.5 or DQ8 have been characterized (Sollid et al. 2012) and work is ongoing to define DQ2.2 restricted T-cell epitopes (Bodd et al. 2012; Dorum et al. 2014). Initial analysis indicated that the peptide-binding motifs of DQ2.5 and DQ2.2 are similar (Johansen et al. 1996a; Johansen et al. 1996b; Stepniak et al. 2008; Vader et al. 2003; van de Wal et al. 1996; van de Wal et al. 1997; Vartdal et al. 1996), albeit it was suggested that proline at P3 is disfavored by DQ2.2, but not by DQ2.5 (Vader et al. 2003), that serine and threonine is favored by DQ2.2 at P3 (van de Wal et al. 1997), and that there could be differences in binding specificity at P6 (Johansen et al. 1996a). The recent finding that gluten-reactive gut T cells of DQ2.2 celiac disease patients recognize epitopes, which are different from those of DQ2.5 celiac disease patients, strongly indicate that these DQ molecules do select different peptides for presentation (Bodd et al. 2012).

Peptide analysis by mass spectrometry (MS) has made huge advances in recent time due to improved sensitivity of instruments and new software that allows reliable measurement of ion intensity. Characterization of endogenously bound peptides will to a great extent reflect which peptides generate kinetically stable peptide-MHC II complexes. Given the impact of quantity of MHC-peptide complexes for activation of T cells, we have undertaken a comprehensive quantitative and comparative mass spectrometric analysis of endogenous peptides bound to DQ2.5, DQ2.2, and DQ7.5 molecules isolated from a panel of nine cell lines. This should also improve our understanding of how these DQ molecules select their peptides for presentation.

## Materials and methods

### Cell lines and culturing

Table 1 lists the Epstein–Barr virus (EBV)-transformed B-lymphoblastoid cell lines (B-LCLs) used in this study. The cell lines 9087 STEINLIN, 9088 PF04015, 9047 PLH, 9050 MOU, 9051 PITOUT, 9037 SWEIG, 9064 AMALA, and 9089 BOB were obtained from the Tenth International Histocompatibility Workshop (http://www.ihwg.org/cellbank), whereas the cell line CD114 was derived from a celiac disease patient. All cell lines were grown in RPMI medium supplemented with 10 % FCS.

**Table 1:** EBV-transformed B-lymphoblastoid cell lines

Cell line	Serological profile			DNA typing profile					
	DR	DQ	DP	DRB1*	DRB3/B4/B5	DQA1*	DQB1*	DPA1*	DPB1*
CD114	3	2.5	1,4	03:01	DRB3*01:01	05:01	02:01	Nd	01:01/04:01
9087 STEINLIN	3	2.5	3,4	03:01	DRB3*01:01	05:01	02:01	01	03:01/04:01
9088 PF04015	3	2.5	1,4	03:01	DRB3*01:01	05:01	02:01	01,02:01	01:01/04:01
9047 PLH	7	2.2	nd	07:01	DRB4*01:01	02:01	02:02	01:03	15:01
9050 MOU	7	2.2	2	07:01	DRB4*01:01	02:01	02:02	01:03	02:01
9051 PITOUT	7	2.2	4	07:01	DRB4*01:01	02:01	02:02	01	04:01
9037 SWEIG	11	7.5	4	11:01	DRB3*02:02	05:05	03:01	01:03	04:02
9064 AMALA	14	7.5	nd	14:02	DRB3*01:01	05:01/05:03	03:01	01:03	04:02/94:01
9089 BOB	11	7.5	4	11:04	DRB3*02:02	05:05	03:01	01	04:02

*Nd* not determined

### Isolation of HLA-DQ-associated peptides

The cells, 7 × 10^7^ for each technical replicate, were lysed for 30 min on ice at a concentration of 10^8^ cells/ml in PBS containing 1 % Nonidet P-40, 5 mM sodium orthovanadate, 25 mM iodoacetamide, 1 mM PMSF, and a protease inhibitor cocktail (complete, EDTA-free tablets; Roche Diagnostics). The lysate was clarified by centrifugation at 15,700×*g* for 20 min at 4 °C, and DQ molecules were immunoprecipitated from the supernatant using SPV-L3 (anti-DQ) (Spits et al. 1984) or 2.12.E11 (anti-DQ2) (Viken et al. 1995) covalently cross-linked to Protein A Sepharose CL-4B. The sepharose were then washed 10 times with 1 ml PBS containing 0.1 % octyl glucoside, 10 times with 1 ml Milli-Q water containing 0.1 % octyl glucoside and finally three times with 1 ml Milli-Q water. After this extensive washing, peptides were acid eluted with 0.1 % trifluoroacetic acid at 37 °C for 5 min two times.

### Analysis by mass spectrometry

All samples were purified by reversed phase chromatography using microcolumns prepared by placing a disk of C18 Empore Extraction Disk (Varian, St. Paul, MN, USA) into 200-μl pipet tips. Peptides were eluted by applying 60 μl of 70 % ACN and 0.1 % formic acid in water. ACN was evaporated in a vacuum drier, and the samples were diluted in 0.1 % formic acid in water before the peptides were analyzed on a Dionex Ultimate 3000 nano-LC system (Dionex, Sunnyvale, CA, USA), which was connected to a quadrupole-Orbitrap (QExactive) mass spectrometer (ThermoElectron, Bremen, Germany) equipped with a nanoelectrospray ion source (Proxeon/Thermo). An Acclaim PepMap100 RSLC column (C18, 2 μm beads, 100 Å, 75 μm inner diameter) (Dionex, Sunnyvale, CA, USA) of a 15-cm bed length was used to separate the peptides. The flow rate used was 0.3 μl/min and the solvent gradient was 5 to 50 % B in 45 min (solvent A: 0.1 % formic acid, solvent B: 90 % ACN/0.1 % formic acid). The mass spectrometer was operated in the data-dependent acquisition mode using the Xcalibur 2.2 software. Single MS full-scan in the Orbitrap (300–1750 m/z, 70,000 resolution at m/z 200, AGC target 1 × 10^6^ maximum IT 20 ms) were followed by 10 data-dependent MS/MS scans in the Orbitrap after accumulation of 1 × 10^6^ ions in the C-trap or an injection time of 120 ms at 35,000 resolution (Kelstrup et al. 2012) (isolation width 2.0 m/z, underfill ratio 0.1 %, dynamic exclusion 20 s) or after accumulation of 2 × 10^5^ ions in the C-trap or an injection time of 60 ms at 17,500 resolution (isolation width 3.0 m/z, underfill ratio 0.4 %, dynamic exclusion 20 s). The normalized collision energy was set to 25 or 30 %.

### Peptide identification and peak volume calculation

MS raw files were submitted to MaxQuant software version 1.3.0.5 (19) using Andromeda as a search engine for peptide and protein identification. Pyro-glu (N-term Q and N-term E), deamidation (NQ), and oxidation (M) were set as variable modifications, and we used a first search error window of 20 ppm and main search error of 6 ppm. No fixed modifications were used. Unspecific enzyme option was selected and no miscleavages were allowed. Mass tolerance for fragment ions was set to 20 ppm. Minimal unique peptides were set to 1, and a false discovery rate of 0.01 (1 %) was used in all instances, and maximum PEP score allowed was 0.1. Identification of peptides was based on parent ion mass and fragmentation spectra. For identification of peptides, retention time alignment was used, ions that had the same parent ion mass and which eluted with the same LC-retention time as a peptide with identified fragmentation spectra were correlated with an alignment window of 3 min. A human database (downloaded from www.UniProt.org December 2013, 89,628 entries) was searched to identify peptides. We selected for generation of contaminants and reversed sequences to assign the presence of contamination and false discovery rates. Quantitative information provided by MaxQuant at the peptide level was obtained using an area under curve assignment, using acquisition features such as mass width, retention time profile, and MS1 ion scan intensity. This value was calculated using a three-dimensional approach and represents the ion peak volume. Peak volumes were calculated only if a minimal of two MS counts were observed (MaxQuant default). To allow sample comparison, the data was normalized to eliminate false-positive comparisons based on sample loading variation. Therefore, for each sample, the peak volume of each individual peptide was normalized using a scale ratio approach where individual peptide values was divided by the sum of the peak volumes for all identified peptides in that same sample, which transform all individual peptide values to a 0–1 scale. The comparison of the amounts of individual peptides across samples was therefore relative.

### Statistical analysis

The statistical tool R version 2.15.2 with the package gplots version 2.11.0 was used to generate a heatmap. Quantitative values obtained from MS acquisition were used as basis for the clustering. Columns were hierarchical clustered with “average” as agglomeration method and “correlation” as distance matrix. Rows were ordered by hierarchical clustering, but with “ward” as agglomeration method and “euclidean” as distance matrix. To evaluate whether there were differences in the quantitative peptide elution data between the various DQ-types beyond what could be expected by chance, we used two approaches. In the first approach, we applied the method SAM (significance analysis of microarrays) (Tusher et al. 2001) to generate a *Z* score for each peptide and each DQ comparison, followed by the convest procedure of the limma R/Bioconductor package (Langaas et al. 2005) to estimate the proportions of peptides with a real difference between the DQ types. In the second approach, we used the globaltest of the R/Bioconductor package (Goeman et al. 2004) to obtain *p* values for overall DQ type differences. The histograms of *Z* scores were generated by the method *locfdr* (Efron 2007).

### Neural network analysis for prediction of binding motifs

Binding motifs for the DQ2.2, DQ2.5, and DQ7.5 molecules were estimated using the *NetMHCIIpan*-*3.0* (Karosiene et al. 2013) and *NNAlign* methods (Andreatta et al. 2011). When using the *NetMHCIIpan*-*3.0* method, sequence logos were constructed as “Shannon logos” from the predicted 9mer binding core of the strongest 2000 predicted binders from a set of 200,000 random natural 15mer peptides using the *Seq2Logo* method with default settings (Thomsen and Nielsen 2012). For the *NNAlign* method, the quantitative peptide elution data (excluding the CLIP peptides) were used for the analysis. Prior to applying the *NNAlign* method, the quantitative abundance values were log-transformed using the relation log(*X* + 0.00001), where *X* is the quantitative abundance value associated with each peptide. Next, the *NNAlign* method was run with settings corresponding to linear rescaling, motif length of 9, Blosum peptide encoding, 3 hidden neurons, 5 network seeds, 5-fold cross validation without early stopping, and common motifs to remove peptide redundancy, and default setting for all other parameters. Logos for the sequence motifs obtained by the *NNAlign* method were constructed as “Shannon logos” using *Seq2Logo* with default settings.

### Competitive peptide-binding assay

Peptide binding was measured in a competitive peptide-binding assay as described previously (Vader et al. 2003). Lysates of Epstein–Barr virus-transformed B-cell lines CD 114 and 9050 MOU (2 × 10^5^ cells/well) were the source of DQ2.5 and DQ2.2 molecules, respectively. Half maximal inhibitory concentrations (IC_50_) were established by measuring the inhibitory effect of binding of indicator peptide (biotin-EPRAPWIEQEGPEYW; used at 0.4 μM or 0.5 μM). Two independent 4-fold titration experiments were performed for each peptide.

## Results

### Relative quantitative analysis of endogenous peptide repertoires of HLA-DQ2.5, HLA-DQ2.2, and HLA-DQ7.5

**Fig. 1:**
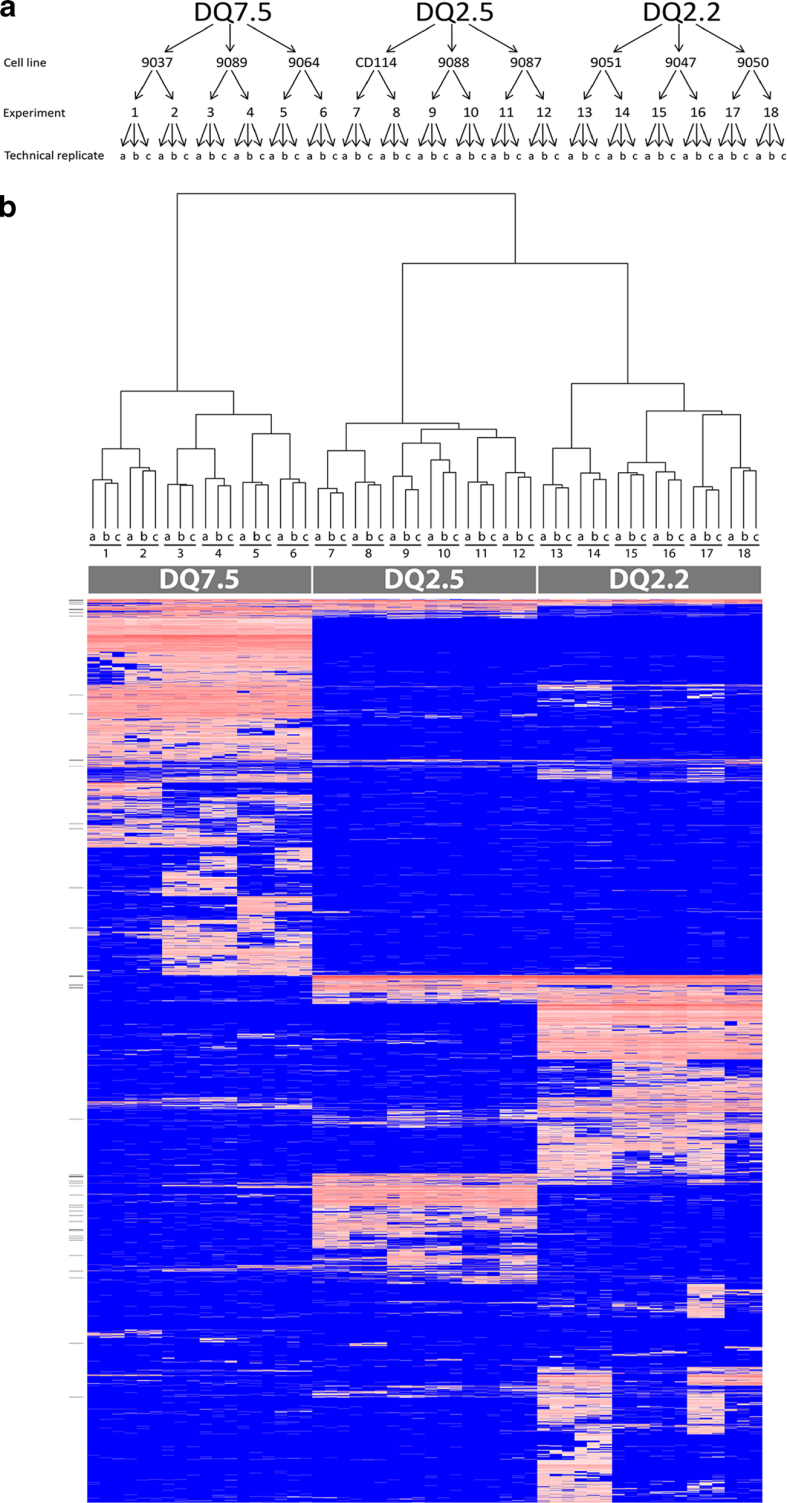
Peptide elution from nine cell lines carrying DQ2.5, DQ2.2, and DQ7.5. **a** Experimental setup of peptide elution experiments. **b** Heatmap and dendrogram of eluted peptides from the nine cell lines carrying DQ7.5, DQ2.5, and DQ2.2. Peptides were eluted from three cell lines expressing DQ7.5, three cell lines expressing DQ2.5, and three cell lines expressing DQ2.2. Two biological replicates of each cell line and three technical replicates for each of these were run. Normalized ion peak volumes were log transformed. The heatmap and dendrogram were created with the statistical software R. The CLIP peptides are indicated with *short lines* on the left side of the heatmap. *Blue* = absent. *Red* = log-transformed ion peak volumes

HLA-DQ molecules were affinity purified with the anti-DQ antibody SPV-L3 from nine EBV-transformed B-LCLs, three cell lines for each HLA type, DQ2.5, DQ2.2, and DQ7.5 (Table 1 and Fig. 1a). Bound peptides were acid eluted and subjected to LC-MS/MS and MaxQuant software analysis. Two experiments were performed for each cell line, and each sample was run in three technical replicates on the LC-MS/MS; in total, 54 samples were analyzed. The information of identified peptides is given in Supplemental Table 1. The data contains peptide sequence information, mass, charge, scoring, and quantitative information. For technical reasons, some experiments gave very low total peptide ion peak volumes and these were not included in the analysis. The MaxQuant software permits estimation of peptide abundance as ion peak volumes, which are based on peptide retention time, mass accuracy, and MS ion intensity as quantifiable parameters. Hierarchical clustering analysis of normalized quantitative data showed that the repertoires of identified peptides were distinct for each of the three DQ molecules, with high similarity between cell lines of the same DQ type (Fig. 1b).

**Fig. 2:**
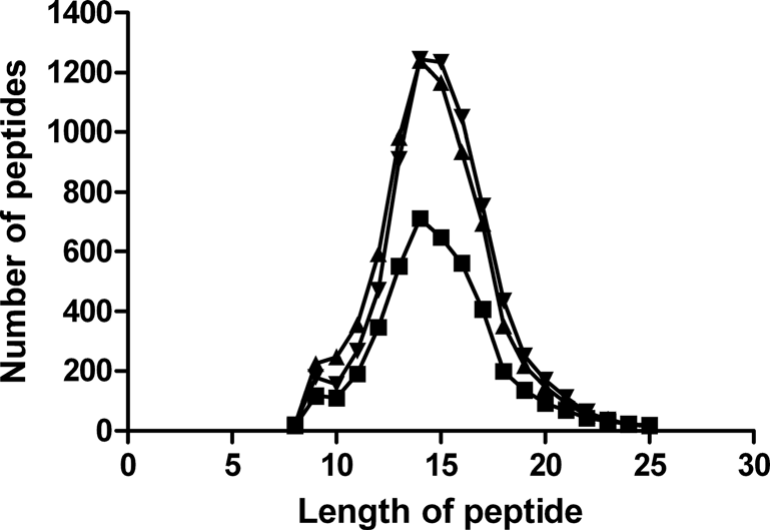
Length distribution of eluted peptides. The number of eluted peptides from DQ2.5 (■), DQ2.2 (▲), and DQ7.5 (▼), and their length distribution are given

The length of the eluted peptides varied between 8 and 25 residues with a peak in distribution at 14 (Fig. 2). Most of the eluted peptides could be assigned to nested sets of sequences. This is in agreement with earlier reports (Chicz et al. 1993; Lippolis et al. 2002). We identified 4267 peptides in the eluates of DQ2.5 molecules, 7395 peptides in the eluates of DQ2.2 molecules, and 7380 peptides in the eluates of DQ7.5 molecules. Altogether, 12,712 unique peptides were identified. The identity and quantity of peptides eluted from the different DQ-types were highly significantly different (globaltest results: DQ2.5 vs DQ2.2, *p* = 9.41 × 10^−24^; DQ2.5 vs DQ7.5, *p* = 1.82 × 10^−24^; and DQ2.2 vs DQ7.5, *p* = 5.43 × 10^−31^). Generating *Z* scores for each peptide and each DQ comparison, we estimated the proportion of peptides with a real difference (convest-procedure) to be the following: DQ2.2 vs DQ2.5: 0.67, DQ2.5 vs DQ7.5: 0.72, and DQ2.2 vs DQ7.5: 0.90 (Supplemental Fig. 1). We also compared peptides eluted from DQ2.2 molecules of the same cell line (9050 MOU) that had been affinity purified with either the SPV-L3 (pan DQ-specific) or 2.12.E11 (DQ2-specific) monoclonal antibodies. Clustering analysis showed that the peptides eluted from the HLA molecules purified with the two different monoclonal antibodies clustered together (data not shown).

### Characteristics of endogenous peptides of HLA-DQ2.5, HLA-DQ2.2, and HLA-DQ7.5

**Fig. 3:**
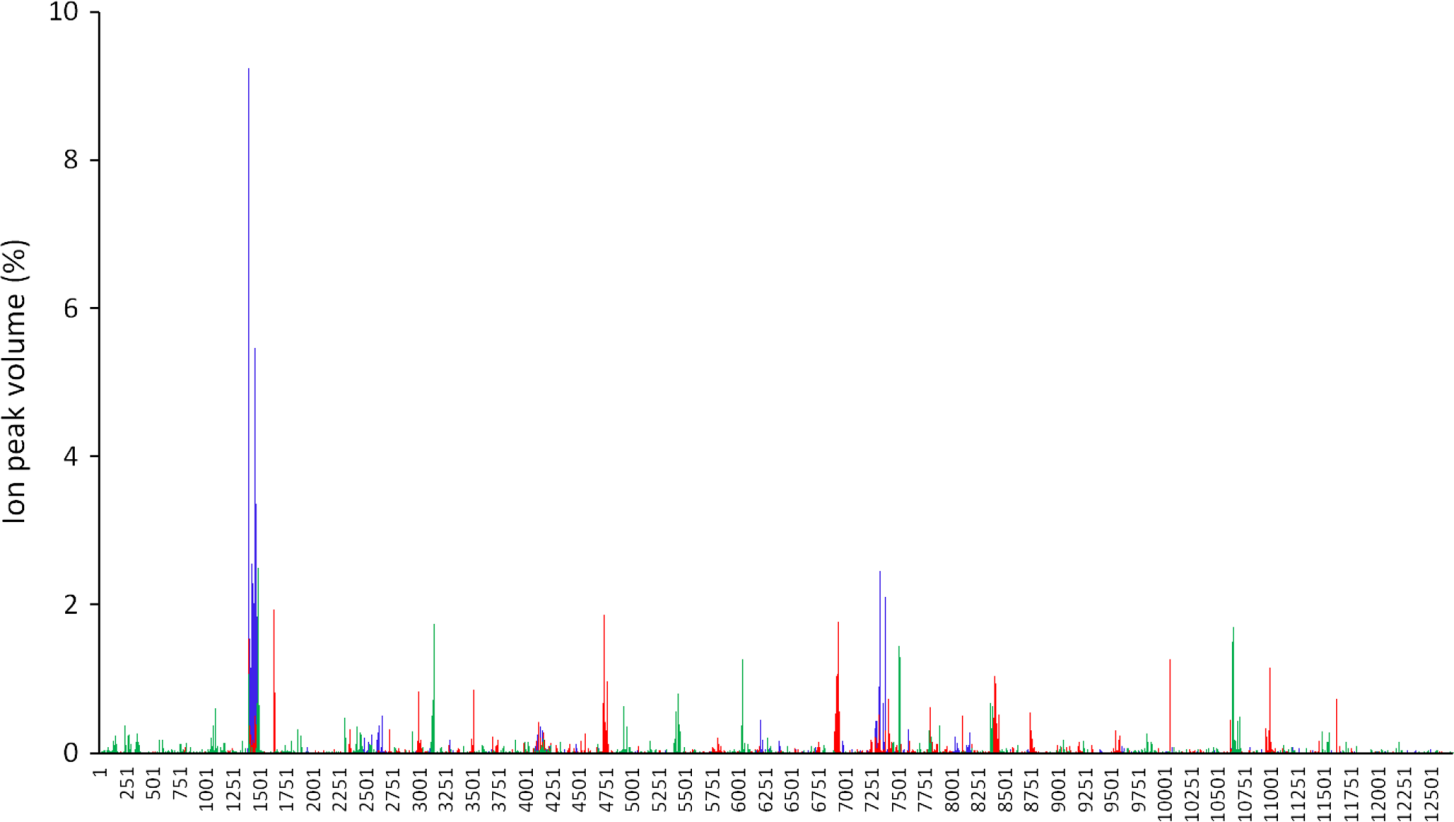
Normalized ion peak volumes of eluted peptides from DQ2.5 (*blue*), DQ2.2 (*red*), and DQ7.5 (*green*). The peptides are presented according to number and sequences as given in Supplemental Table 2. The ion peak volumes were normalized against total ion peak volumes of all eluted peptides for each technical replicate and are shown in percentage (%). The average ion peak volume of all samples for each HLA type is shown

We found greater differences in diversity and relative abundance of endogenous peptides from DQ2.2 and DQ7.5 compared to DQ2.5 (Tables 2, 3, and 4; Fig. 3). This is seen by inspecting the entire array of eluted peptides (Fig. 3 and Supplemental Table 2), but is also apparent from looking at the 20 most abundant peptides eluted from each molecule (Tables 2, 3, and 4). The normalized ion peak volume represented by these 20 peptides was 52 % for DQ2.5, 23 % for DQ2.2, and 23 % for DQ7.5. For DQ2.5, 16 of the 20 peptides were length variants of CLIP (from invariant chain/CD74), and two peptides were length variants of a HLA class I peptide known to be a DQ2.5 high-affinity binder (Table 2). For DQ2.2, seven of the peptides derived from β_2_-microglobulin (representing two different core sequences), five of the peptides derived from serglycin, and three peptides derived from invariant chain (CD74) of which only one were a typical CLIP peptide (Table 3). For DQ7.5, seven of the 20 most abundant peptides were CLIP peptides, and there were peptides derived from transferrin receptor (five peptides with two different sequences) and MHC class II DRα-chain (three peptides) (Table 4).

**Table 2:** Twenty most abundant peptides eluted from DQ2.5

Peptide	Protein	Normalized ion peak volume (%)
LPKPPKPVSKMRMATPLLMQALP	CD74	9.2
PKPPKPVSKMRMATPLLMQALP	CD74	5.5
RMATPLLMQALPMGALPQ	CD74	5.5
MATPLLMQALPMGALPQ	CD74	3.4
RMATPLLMQALPMGALP	CD74	2.7
KPPKPVSKMRMATPLLMQALPM	CD74	2.6
EPRAPWIEQEGPEYWDRN	MHC class I	2.5
MATPLLMQALPMGALP	CD74	2.3
KMRMATPLLMQALPMGALPQ	CD74	2.3
EPRAPWIEQEGPEYWDQE	MHC class I	2.1
MRMATPLLMQALPMGALPQ	CD74	2.0
MATPLLMQALPM	CD74	1.8
RMATPLLMQALPM	CD74	1.7
KPPKPVSKMRMATPLLMQALP	CD74	1.5
RMATPLLMQALPMGAL	CD74	1.5
MATPLLMQALPMGAL	CD74	1.5
LPKPPKPVSKMRMATPLLMQALPM	CD74	1.1
ATPLLMQALPMGALPQ	CD74	1.0
SLDRNLPSDSQDLGQHGLEEDFML	Serglycin	0.9
PSDSQDLGQHGLEEDFML	Serglycin	0.9

**Table 3:** Twenty most abundant peptides eluted from DQ2.2

Peptide	Protein	Normalized ion peak volume (%)
DPSSGLGVTKQDLGPVPM	CD74	1.9
SLDRNLPSDSQDLGQHGLEEDFML	Serglycin	1.9
SGFHPSDIEVDLLK	β_2_-microglobulin	1.8
LPKPPKPVSKMRMATPLLMQALP	CD74	1.5
YRYDLASGATEQLPLT	Sortilin-related receptor	1.3
LPSADEIYDCKVEHWG	MHC class II DQA	1.2
QDLGQHGLEEDFML	Serglycin	1.1
HPSDIEVDLLK	β_2_-microglobulin	1.1
YYTEFTPTEKDEY	β_2_-microglobulin	1.0
GFHPSDIEVDLLK	β_2_-microglobulin	1.0
LPSDSQDLGQHGLEEDFML	Serglycin	1.0
QDLGQHGLEEDFM	Serglycin	1.0
YYTEFTPTEKDE	β_2_-microglobulin	1.0
YLLYYTEFTPTEKDEY	β_2_-microglobulin	1.0
YLLYYTEFTPTEKDE	β_2_-microglobulin	0.9
LPSTEDVYDCRVEHWG	MHC class II DRA	0.9
IDNKGIDSDASYPYK	Cathepsin S	0.9
VKTLTGKTITLEVEPSDT	Ubiquitin	0.8
PSDSQDLGQHGLEEDFML	Serglycin	0.8
GVTKQDLGPVPM	CD74	0.8

**Table 4:** Twenty most abundant peptides eluted from DQ7.5

Sequence	Protein	Normalized ion peak volume (%)
TPLLMQALPMGALPQ	CD74	2.5
ASFEAQGALANIAVDKA	MHC class II DRA	1.7
NPGGYVAYSKAATVTGKL	Transferrin receptor	1.7
TPLLMQALPMGALPQGP	CD74	1.5
NPGGYVAYSKAATVTG	Transferrin receptor	1.5
VPVPQFGGGDPADIIHD	Integral membrane protein 2C	1.4
TPLLMQALPMGALPQGPM	CD74	1.4
VPVPQFGGGDPADIIHDF	Integral membrane protein 2C	1.3
NPGGYVAYSKAATVTGK	Transferrin receptor	1.3
IPELNKVARAAAEVAGQF	Transferrin receptor	1.3
ASFEAQGALANIAVDK	MHC class II DRA	1.1
LPKPPKPVSKMRMATPLLMQALP	CD74	1.1
VDNALQSGNSQESVTEQ	Ig kappa chain C region	0.8
SFEAQGALANIAVDKA	MHC class II DRA	0.7
IEKVEHSDLSFSKDWS	β_2_-microglobulin	0.7
ATPLLMQALPMGALPQGPM	CD74	0.7
ATPLLMQALPMGALPQGP	CD74	0.7
IPELNKVARAAAEVAG	Transferrin receptor	0.6
LMQALPMGALPQGP	CD74	0.6
IEKVEHSDLSFSKD	β_2_-microglobulin	0.6

The abundance of CLIP peptides was particularly evident for DQ2.5. The sum of the normalized ion peak volumes of all CLIP variants was 53 % for DQ2.5, 5 % for DQ2.2, and 12 % for DQ7.5. DQ2.5, and DQ2.2 displayed similar sets of CLIP peptides, but each peptide species was present at very different ion peak volumes (Supplemental Fig. 2). Several CLIP peptides were unique to DQ7.5, and some of these did not contain the conventional CLIP1 or the alternative CLIP2 cores (Supplemental Fig. 2).

### Defining peptide-binding motifs from elution data with neural network analysis

**Fig. 4:**
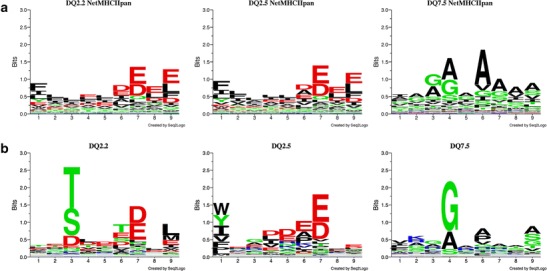
Sequence logos showing the peptide-binding motifs of DQ2.2, DQ2.5, and DQ7.5. **a** Sequence logos defined by *NetMHCIIpan*-*3.0*. The sequence logos of DQ2.5 and DQ7.5 are based on previously published experimental data of peptide binding whereas the sequence logo of DQ2.2 is a prediction based on extrapolation from other MHC class II molecules characterized by experimental peptide-binding data. **b** Sequence logos defined by *NNAlign* using quantitative elution data as listed in Supplemental Table 2 excluding CLIP-peptides (entries 1395–1515). Logos were made using *Seq2Logo* as described in the text

To assess the binding specificity of the DQ2.5, DQ2.2, and DQ7.5 molecules, we subjected the quantitative values for eluted peptides as listed in Supplemental Table 2 to the neural network-based method *NNAlign* (Andreatta et al. 2011). The peptide-binding motifs obtained were compared with peptide-binding motifs predicted by the *NetMHCIIpan*-*3.0* (Karosiene et al. 2013) that was trained on an extensive set of experimental MHC class II peptide-binding data including peptide binding to DQ2.5 and DQ7.5, but not including peptide-binding data to DQ2.2 (Wang et al. 2008). This latter method also allows prediction of the binding motif of DQ2.2 even though no peptide-binding data was available for this molecule when the method was constructed. The results obtained by both methods are depicted as sequence logos in Fig. 4. The *NetMHCIIpan* analysis, based on previously available data, revealed very similar peptide-binding motifs for DQ2.5 and DQ2.2, which were different from the binding motif of DQ7.5 (Fig. 4a). By contrast, the *NNAlign* analysis, which was based on the quantitative elution data provided here, revealed distinct binding motifs of DQ2.2 and DQ2.5 (Fig. 4b). As its main characteristic, DQ2.2 has a unique binding motif with a preference at P3 for threonine and serine and to lesser extent for aspartate. In contrast, there is no amino acid preference observed at P3 for DQ2.5 or DQ7.5. The binding motifs of DQ2.5 and DQ7.5, but not that of DQ2.2, correspond very well with the predictions obtained with the *NetMHCIIpan* method. As a strong S/T anchor has not been indicated in the binding data for any of the DQ molecules utilized to train *NetMHCIIpan*, it is expected that this method does not report the P3 S/T anchor for DQ2.2. Notably, the binding motifs of DQ2.5 and DQ7.5 obtained by *NNAlign* analysis were similar to the binding motifs reported previously for both molecules. We also performed a *NNAlign* analysis on a filtered dataset of 10,661 peptides established by removing peptides with length <11 residues and peptides not being part of nested sets. This analysis gave almost identical binding motifs for all three molecules (data not shown).

### Substitution analysis shows the importance of serine at position P3 of the binding frame

**Fig. 5:**
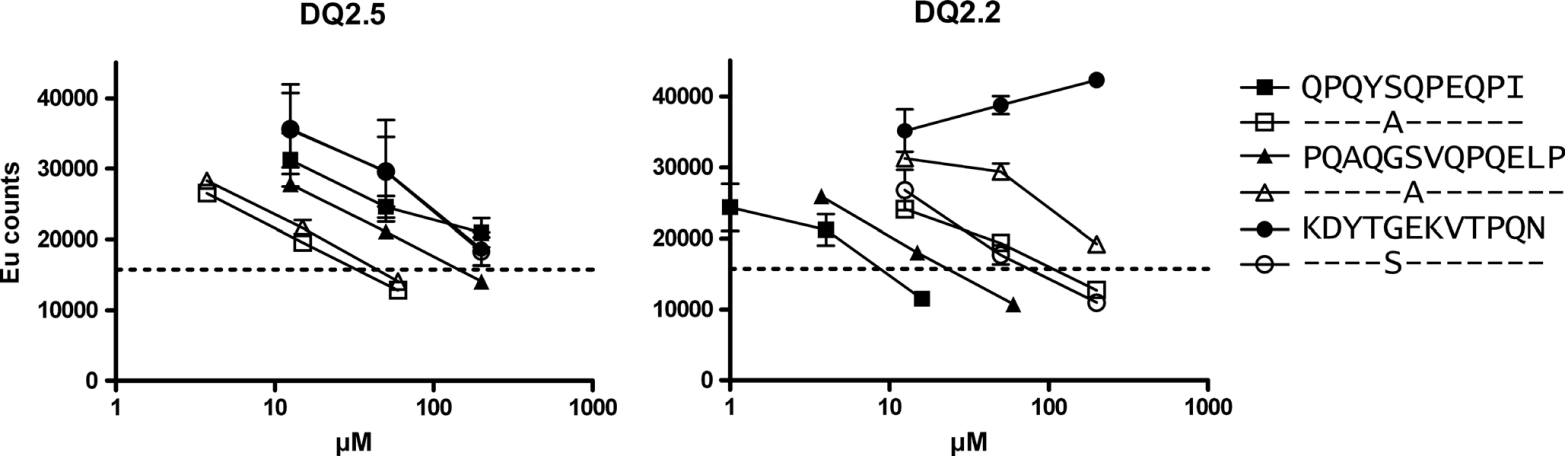
Binding of peptides to DQ2.5 and DQ2.2. Peptide binding of two DQ2.2 gluten epitopes and one DQ2.5 self-peptide with P3 substitutes was assessed in a competitive-binding assay. The two DQ2.2 gluten epitopes were tested with Ser to Ala susbstitutions at position P3, while the DQ2.5-binding peptide was tested with Gly to Ser substitution at position P3. The binding register of this peptide was predicted by the *NNAlign* method, and to avoid a register shift that could confound binding assay results, a Lys residue was introduced at position P5. Two independent 4-fold titration experiments were performed. The results shown are from one representative experiment

The importance of serine or threonine was investigated by testing peptides with and without substitution at position P3 in a biochemical competition assay for binding to DQ2.5 and DQ2.2 (Fig. 5). Three peptides were tested; two newly identified DQ2.2 gluten epitopes, DQ2.2-glia-α1 (core sequence QGSVQPQEL) and DQ2.2-glia-α2 (core sequence QYSQPEQPI), and one of the DQ2.5 self-peptides identified in this study (core sequence YTGEDVTPQ). We found that all peptides containing serine at position P3 bound with higher affinity to DQ2.2 compared with alanine or glycine at the same position (Fig. 5). The opposite tendency was seen for binding to DQ2.5.

## Discussion

We have performed a relative quantitative comparison of the endogenous peptide repertoires of DQ2.5, DQ2.2, and DQ7.5. DQ molecules were affinity purified from a panel of nine cell lines with three cell lines of each DQ-type. Bound peptides were eluted and subjected to MS for identification and quantification. Large differences in normalized ion peak volumes of distinct peptides were observed. The repertoires of peptides were unique for each of the three DQ molecules. Notably, the two DQ2 variants had clearly different peptide-binding motifs. This provides an explanation for why DQ2.5 and DQ2.2 celiac disease patients select distinct sets of gluten peptides for presentation to CD4^+^ T cells, which again explains why the two DQ2 variants have widely different risks for celiac disease.

Our approach of quantitative proteomics of endogenous HLA-bound peptide repertoires is feasible due to recent breakthroughs in MS with increase in instrument sensitivity as well as improved software, allowing precise measurement of ion peak volumes across different samples. We performed a large-scale study where altogether 54 samples of nine distinct cell lines were examined by relative comparison. Ionization intensity for different peptides may vary during and across MS sample acquisition, but this variation is fairly modest with LC and modern electrospray instrumentation. The same patterns of peptides were observed for technical and biological replicates as well as for samples representing the same HLA allelic molecule of different cell lines. This strongly suggests that the different quantitative patterns of peptides eluted of different DQ molecules reflect differences in the peptide-binding characteristics of the distinct DQ molecules.

Analysis of endogenous peptides to determine peptide-binding specificity of MHC class II molecules has previously been done by N-terminal pool sequencing (Falk et al. 1994; Godkin et al. 1997; Khalil-Daher et al. 1998; Vartdal et al. 1996). In contrast to MCH class I molecules where this approach has proven very successful due to the uniform length of peptide ligands, the analysis is complicated by the variable length and ragged ends of MHC class II ligands. We here derived the binding specificity from quantitative data on several thousand individual peptides from each HLA-DQ molecule using the artificial neural network tool *NNAlign. NNAlign* allows identification of the binding motifs in quantitative peptide data sets also in situations where the peptides have variable length. This approach has several advantages compared to the pool sequencing. First and foremost, the method is fully automated and unbiased. Second, this method returns a model (an artificial neural network) that readily can be applied to searches for peptides matching the binding specificity in any protein/proteome of interest. Finally, this model produces quantitative prediction values that are directly correlated to the quantitative values associated with the peptides used to construct the method. This latter feature is essential when applying the model to identify novel potential ligands and as guide to prioritize any subsequent peptide selection.

The *NetMHCIIpan* method, which is based on a large body of experimental peptide affinity binding data to a large panel of MHC class II molecules (including DQ2.5, but not DQ2.2) and therefore is believed to be able to predict the binding motif of any MHC class II molecule, indicates that the binding motifs of DQ2.5 and DQ2.2 are almost identical (Fig. 4a). However, using the *NNAlign* method to analyze the present quantitative peptide-DQ elution data revealed that DQ2.2 has a unique peptide-binding motif with a preference for threonine and serine, and to a lesser extent for aspartate, at the P3 position as the major requirement. DQ2.5 and DQ2.2 are highly homologous molecules deviating by 10 amino acids in the membrane distal domains of which only one residue, DQα22, make contact with the peptide in the x-ray crystal structure of DQ2.5 (Kim et al. 2004). Given this similarity, the prominent distinction at P3 is remarkable. The *NNAlign* analysis of eluted peptides predicts similar preference at P6 and P7 for DQ2.2 and DQ2.5, whereas there are differences at the P1 and P9 positions, with indications for an anchor at P9 for DQ2.2 and no anchor at P9 for DQ2.5. These observations are at odds with previous reports, which have indicated that both P1 and P9 are anchors for DQ2.5 and DQ2.2 (Johansen et al. 1996a; Johansen et al. 1996b; Stepniak et al. 2008; van de Wal et al. 1996; van de Wal et al. 1997; Vartdal et al. 1996).

The risk for celiac disease associated with DQ2.5 and DQ2.2 is remarkably different (Margaritte-Jeannin et al. 2004; Sollid and Lie 2005). Studying gluten-reactive T cells from DQ2.5 celiac patients with antigen-presenting cells expressing DQ2.5 or DQ2.2, reactivity to all gluten epitopes defined by DQ2.5 restricted T cells were observed albeit with small quantitative differences (Qiao et al. 2005; Vader et al. 2003). It was only when antigen presenting cells were pulsed with antigen and incubated for extended time before mixing with T cells that qualitative differences were observed showing that epitopes could make kinetically stable complexes with DQ2.5 molecules but not with DQ2.2 molecules (Fallang et al. 2009). In this setting, epitopes defined by DQ2.5 restricted T cells were only presented by DQ2.5- and not by DQ2.2-expressing antigen-presenting cells. Thus, we reasoned that different gluten epitopes would be presented in DQ2.5- vs DQ2.2-positive celiac patients. Isolating gluten-reactive T cells from celiac disease patients expressing DQ2.2, we demonstrated that distinct gluten epitopes were recognized by CD4^+^ T cells in DQ2.5 compared to DQ2.2 celiac patients, and the first DQ2.2 restricted epitope, DQ2.2-glut-L1 (9-mer core region: PFSEQEQPV) was identified (Bodd et al. 2012). This epitope binds with high kinetic stability to DQ2.2, but not to DQ2.5. The epitope has a serine residue at position P3, which is important for binding as its substitution with alanine or glycine, but not with threonine, gives impaired binding (Bodd et al. 2012). Later, it was demonstrated that six out of six gluten peptides, which could be enriched for binding to DQ2.2 from complex gluten digests, harbored a serine at position P3, and two of these peptides (DQ2.2-glia-α1: QGSVQPQEL and DQ2.2-glia-α2: QYSQPEQPI) were proven to be celiac disease T-cell epitopes (Dorum et al. 2014). Thus, studies of gluten epitopes in celiac disease strongly suggest that DQ2.5 and DQ2.2, despite previous studies reporting strong similarity in their peptide-binding motifs (Johansen et al. 1996a; van de Wal et al. 1997), display different peptides for presentation to T cells.

The polymorphism at DQα22, where DQ2.5 has a tyrosine and DQ2.2 has a phenylalanine, was linked with the ability of gluten epitopes to make kinetically stable complexes to DQ2.5 (Fallang et al. 2009). The tyrosine of DQ2.5 participates in a hydrogen bond via a water molecule to the main chain of the peptide as well as to histidine at DQα24 (Kim et al. 2004). The phenylalanine of DQ2.2 lacks the hydroxyl group of tyrosine and is unable to participate in this hydrogen-bonding network. Modeling suggested that in peptides with serine or threonine at P3, the hydroxyl group of these residues could hydrogen bond to histidine DQα24, which would explain why such peptides could bind stably to DQ2.2 (Bodd et al. 2012). Our current analysis of accumulated endogenous peptides of DQ2.2 strongly supports that serine or threonine residues at position P3 are instrumental for stable binding of peptides to this HLA-DQ molecule. Substitution analysis of three peptides, two DQ2.2 gluten epitopes, and one DQ2.5 self-peptide demonstrated that peptides with serine at position P3 bound with higher affinity to DQ2.2 than peptides with alanine or glycine at position P3. Our findings are in line with a previous study by van de Wal and co-workers which demonstrated that substitution of the P3 glutamine residue of the MHC class I 51–60 endogenous ligand with serine, threonine, or aspartate led to an improved peptide binding to DQ2.2 (van de Wal et al. 1997).

Celiac disease patients who are not DQ2.5, DQ8, or DQ2.2 carry DQ7.5 (Karell et al. 2003). It is likely that such patients, similar to patients who are DQ2.5, DQ8, or DQ2.2 have gluten reactive CD4^+^ T cells in the intestinal mucosa. However, whereas the DQ2.5, DQ8, and DQ2.2 restricted gluten epitopes have negative-charged anchor residues (Bodd et al. 2012; Sollid et al. 2012), the binding motif of DQ7.5, which does not include the involvement of charged residues, suggests that DQ7.5 restricted gluten epitopes (if they exist) are qualitatively different—likely without the key role of glutamate residues resulting from transglutaminase 2-mediated deamidation as seen for the gluten epitopes characterized to date. Altogether, the peptide elution data support the notion that the three celiac disease-associated molecules DQ2.5, DQ2.2, and DQ7.5 have different specificity requirements for peptide binding, which would lead to different repertoires of HLA ligands and different amounts of gluten peptides being presented to CD4+ T cells. This again could account for why the three DQ variants have distinct risks for celiac disease.

As expected, there was a dominance of CLIP peptides for DQ2.5 with higher ion peak volumes than for CLIP peptides eluted from DQ2.2 and DQ7.5. Correspondingly, the remaining number of identified endogenous peptides for DQ2.5 was lower than the number of endogenous peptides identified for DQ2.2 and DQ7.5. This disproportionate representation of individual peptide sequences is likely explained by the dominance of CLIP peptides for DQ2.5. The CLIP peptides eluted from DQ2.5 and DQ2.2 contained the conventional CLIP1 (Malcherek et al. 1995) or the alternative CLIP2 (Fallang et al. 2008; Wiesner et al. 2008) cores, or both. Interestingly, several CLIP peptides were unique to DQ7.5, and some of these did not contain the CLIP1 or CLIP2 cores. Possibly, DQ7.5 can bind some CLIP peptides in yet another unique register, likely explained by the peptide-binding specificity of this molecule.

Taken together, we here demonstrate that the three DQ molecules associated with celiac disease, DQ2.5, DQ2.2, and DQ7.5 display endogenous peptides that differ in sequence and quantity. Even the highly homologous DQ2 variants, DQ2.5 and DQ2.2, differ in their peptide-binding preference. This difference translates into selection of distinctive gluten epitopes by the three DQ allotypes for presentation to T cells in celiac disease.

## Electronic supplementary material

Below is the link to the electronic supplementary material.

Supplemental Fig. 1 (DOCX 327 kb)

Supplemental Fig. 2 (DOCX 428 kb)

Supplemental Table 1 (XLSX 4583 kb)

Supplemental Table 2 (DOCX 993 kb)
