# Supplementary material for: Different binding motifs of the celiac disease-associated HLA molecules DQ2.5, DQ2.2, and DQ7.5 revealed by relative quantitative proteomics of endogenous peptide repertoires
Source: Immunogenetics. 2014 Dec 12;67(2):73–84. doi: 10.1007/s00251-014-0819-9 (PMC4297300; doi:10.1007/s00251-014-0819-9)
Supplement: Supplementary file 1 — (DOCX 327 kb) [file 251_2014_819_MOESM1_ESM.docx]

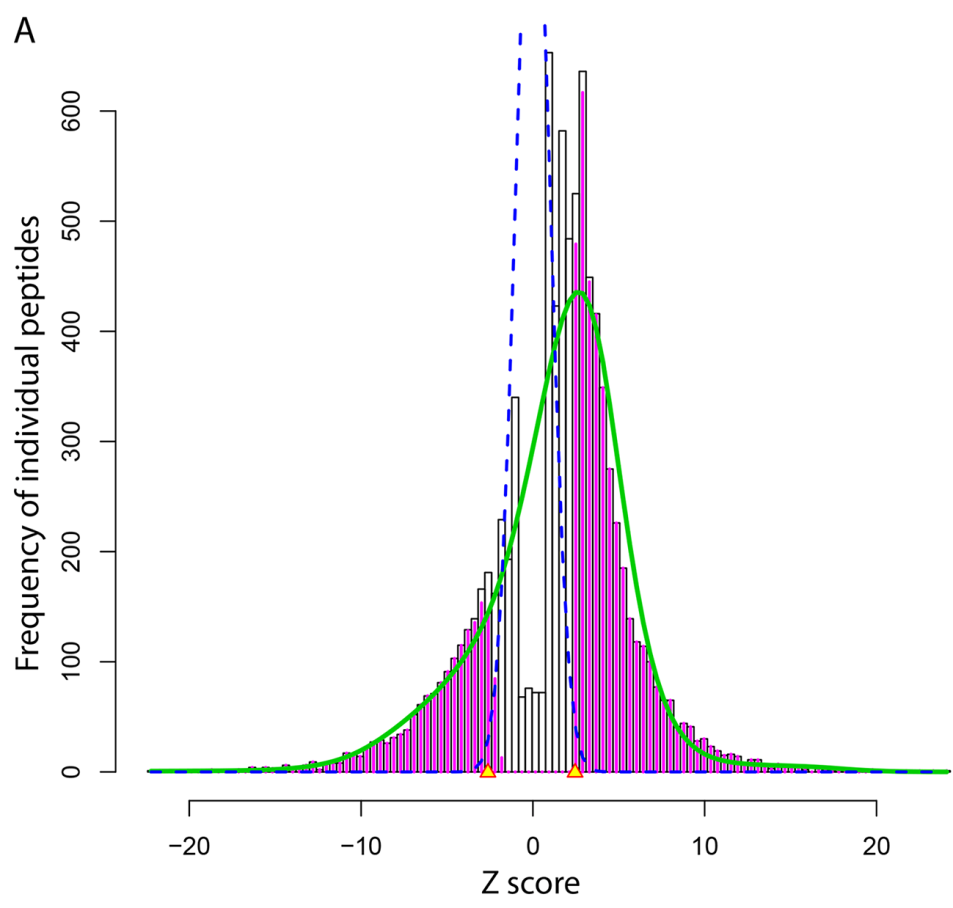

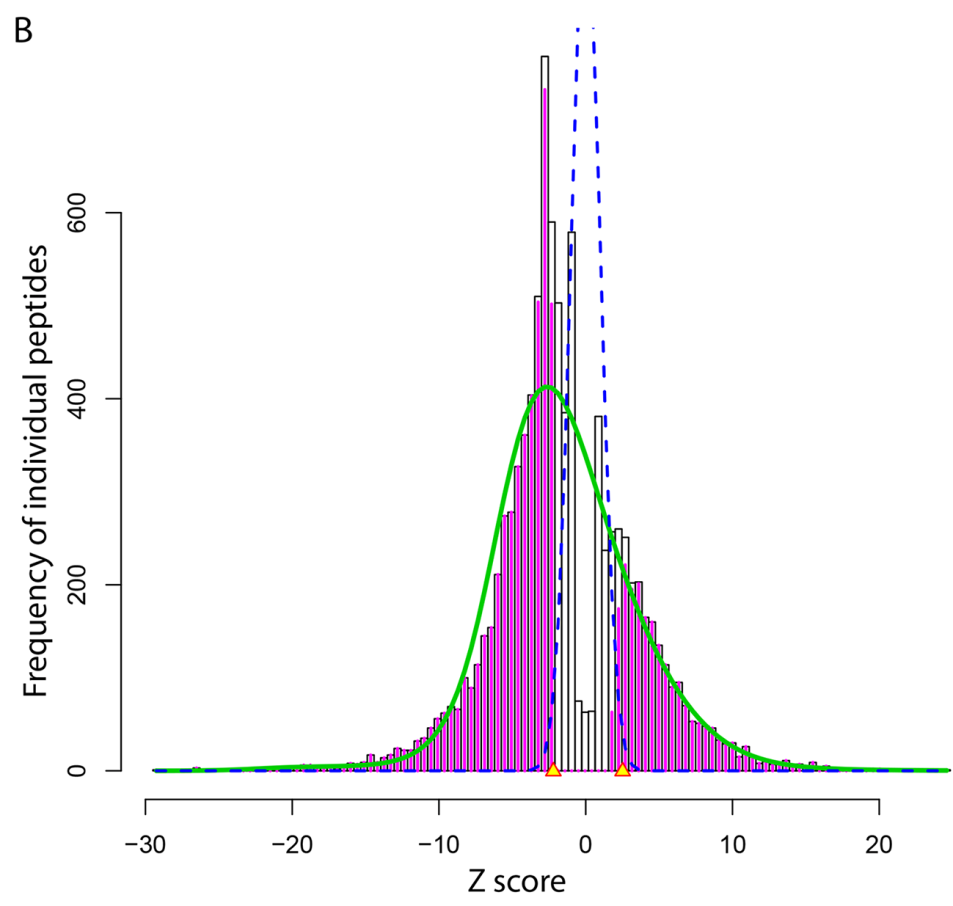

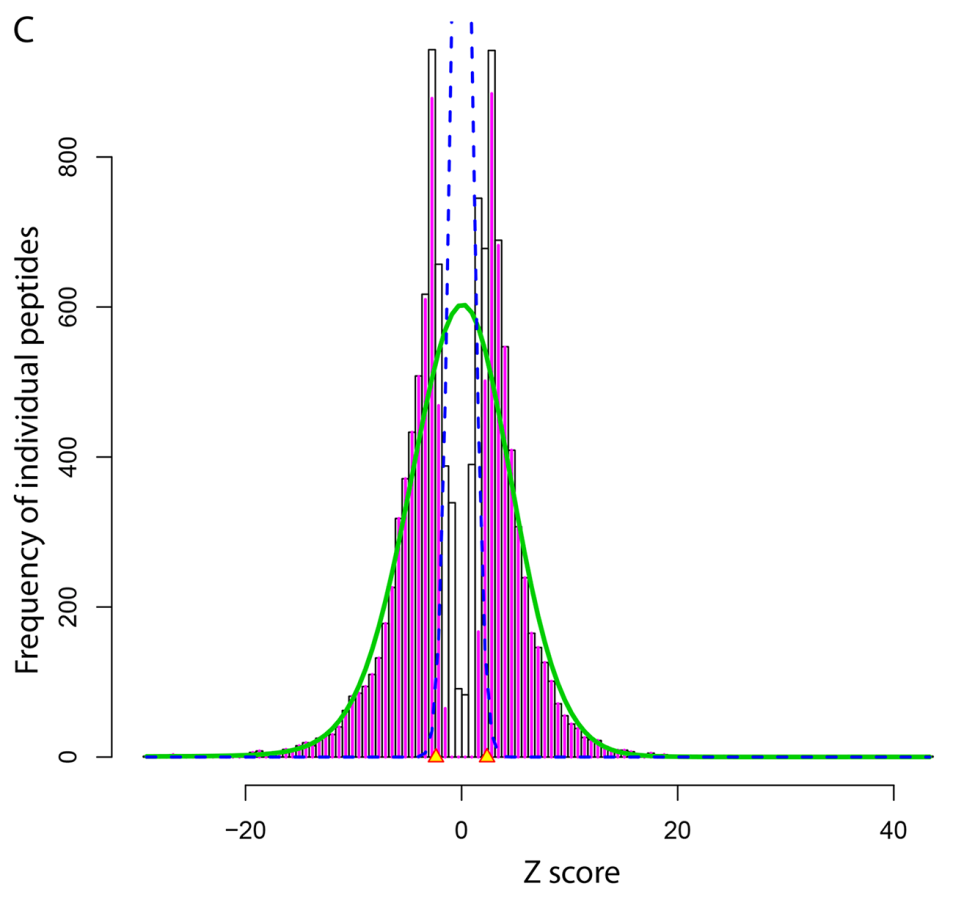


**Supplemental Figure S1. Histograms of Z scores for each peptide generated by the method *locfdr*.** The white bars represent the observed test statistics based on a modified t-test (SAM: significance analysis of microarrays) for each peptide for the various DQ type comparisons. The blue dotted lines show what one would expect if there were no real differences (just random, due to technical noise etc.) and the yellow triangles mark the 2.5% and the 97.5% quantiles in this distribution. The pink bars denote what the method estimates as real differences, and represent the difference between the white bars and the blue dotted line. The green lines denote the smoothed distribution of the observed histogram. *A* DQ2.2 vs DQ2.5, *B* DQ2.5 vs DQ7.5, *C* DQ2.2 vs DQ7.5.
