# Supplementary material for: Different binding motifs of the celiac disease-associated HLA molecules DQ2.5, DQ2.2, and DQ7.5 revealed by relative quantitative proteomics of endogenous peptide repertoires
Source: Immunogenetics. 2014 Dec 12;67(2):73–84. doi: 10.1007/s00251-014-0819-9 (PMC4297300; doi:10.1007/s00251-014-0819-9)
Supplement: Supplementary file 2 — (DOCX 428 kb) [file 251_2014_819_MOESM2_ESM.docx]

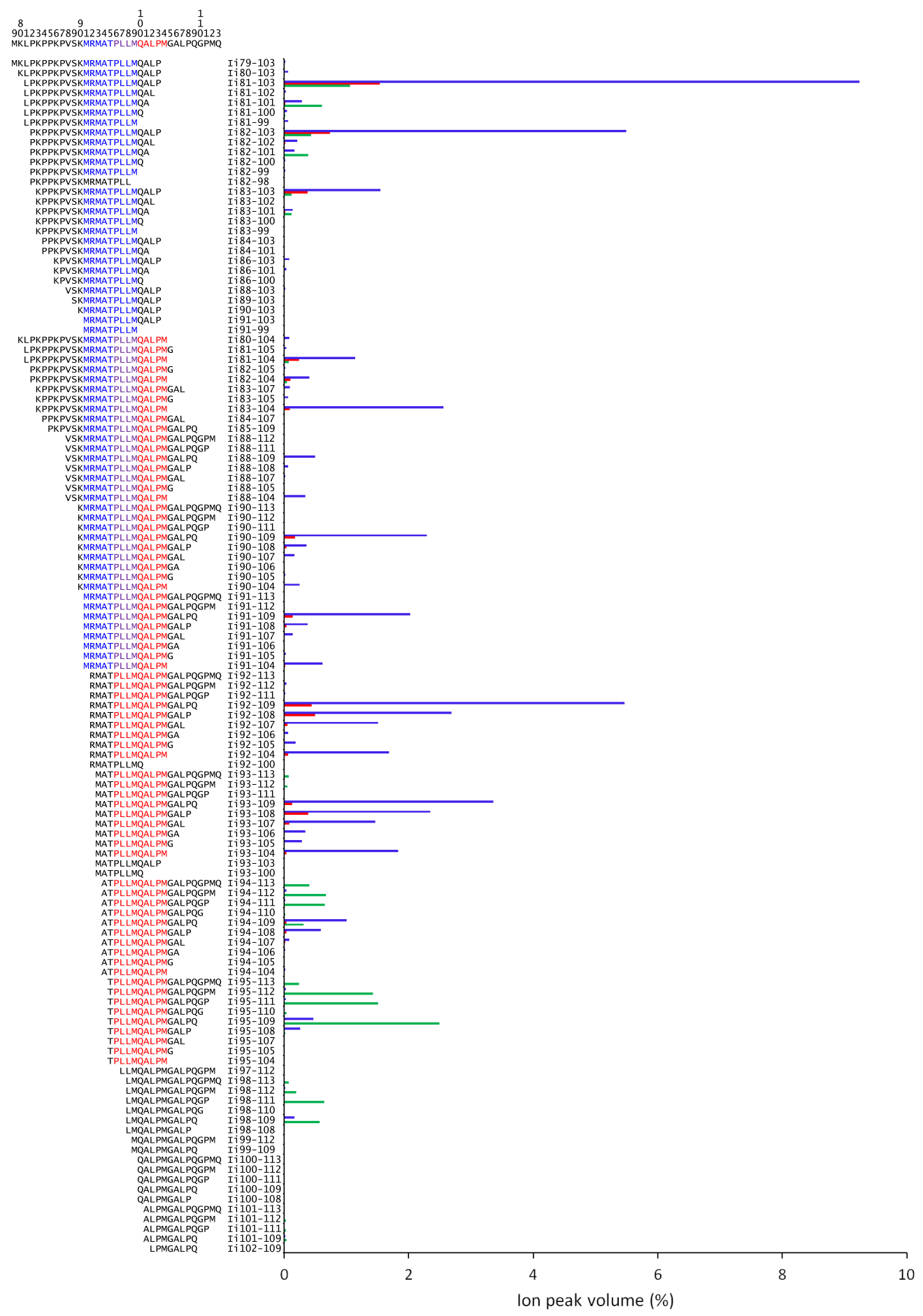


**Supplemental Figure S2.** **Ion peak volumes of eluted CLIP peptides from DQ2.5 (blue), DQ2.2 (red) and DQ7.5 (green).** The peptides are grouped after sequences and display a zoom in of all CLIP peptides in the range 1395-1515 as shown in Figure 3. The ion peak volumes were normalized against total ion peak volumes of all eluted peptides for each technical replicate and are shown in %. The CLIP1 binding frame is given in blue. The CLIP2 binding frame is shown in red. The overlapping sequence between CLIP1 and CLIP2 is shown in purple.
