# Supplementary material for: Different binding motifs of the celiac disease-associated HLA molecules DQ2.5, DQ2.2, and DQ7.5 revealed by relative quantitative proteomics of endogenous peptide repertoires
Source: Immunogenetics. 2014 Dec 12;67(2):73–84. doi: 10.1007/s00251-014-0819-9 (PMC4297300; doi:10.1007/s00251-014-0819-9)
Supplement: Supplementary file 4 — (DOCX 993 kb) [file 251_2014_819_MOESM4_ESM.docx]

**Table S2.** Average normalized ion peak volume in % for the eluted peptides from DQ2.5, DQ2.2 and DQ7.5^a)^

| Peptide number | Sequence | DQ2.5 | DQ2.2 | DQ7.5 |
| --- | --- | --- | --- | --- |
| 1 | AAAAAAAAAAAAA | 0 | 0 | 0.00577819 |
| 2 | AAAAAAAAAAAAAA | 0 | 0 | 0.008003277 |
| 3 | AAAAAAAAAAAAAAA | 0 | 0 | 0.022440847 |
| 4 | AAAAAAAAAAAAAGAGA | 0 | 0 | 0.003655584 |
| 5 | AAAAAAAAAAAGAGA | 0 | 0 | 0.010805966 |
| 6 | AAAAAAAAAAAGAGAG | 0 | 0 | 0.002158076 |
| 7 | AAAAAAAAAAGAGAG | 0 | 0 | 0.000394303 |
| 8 | AAAAAAAAAAGAGAGA | 0 | 0 | 0.003283764 |
| 9 | AAAAAAAAAGAGAG | 0 | 0 | 0.000755383 |
| 10 | AAAAAAAAAGAGAGA | 0 | 0 | 0.00882343 |
| 11 | AAAAAAAAPAAAAT | 0 | 0 | 0.000638788 |
| 12 | AAPGSAAPAAGSAP | 2.23591E-05 | 0 | 0.000707764 |
| 13 | APGSAAPAAGSAPAA | 0 | 0 | 0.000157049 |
| 14 | ASGGAAAAAAAPAAP | 0 | 0 | 0.000326066 |
| 15 | GPAPAAGAAPAGGP | 0 | 0 | 0.000756475 |
| 16 | GPAPAAGAAPAGGPA | 0 | 0 | 0.008365584 |
| 17 | GPAPAAGAAPAGGPAP | 0 | 0 | 0.026869509 |
| 18 | GPAPAAGAAPAGGPAPS | 0 | 1.83188E-05 | 0.000355168 |
| 19 | PGSAAPAAGSAPAAA | 0 | 0 | 0.000597021 |
| 20 | SGGAAAAAAAPAAPA | 0 | 0 | 0.000931213 |
| 21 | SGGAAAAAAAPAAPAAP | 0 | 0 | 0.000499676 |
| 22 | SGGAAAAAAAPAAPAAPR | 0 | 0 | 0.000432917 |
| 23 | TAAAAAAAAAAAA | 0 | 0 | 0 |
| 24 | TAAAAAAAAAAAAAA | 0 | 0 | 0.001491239 |
| 25 | TPGAATASASGAA | 0 | 1.78868E-05 | 0.000581669 |
| 26 | TPPAPAPAAPPSTP | 0 | 0 | 0.004694134 |
| 27 | TPPAPAPAAPPSTPAAPK | 0 | 0 | 0.001288611 |
| 28 | AAAAAAAPAAPAAPR | 0 | 0 | 0.004418621 |
| 29 | APVAAATTAAPAAAAAPAK | 0 | 0 | 0.000102423 |
| 30 | GAAAAAAAPAAPAAPR | 0 | 0 | 0.000504464 |
| 31 | AKEAAAAAAAAAAAAAAA | 0 | 0 | 0.026840361 |
| 32 | EPAAAGSGAASPSA | 0 | 0 | 0.006736864 |
| 33 | EPAAAGSGAASPSAA | 0 | 0 | 0.007357376 |
| 34 | EPGGGAAGPAAAA | 0 | 8.5146E-05 | 0.002143998 |
| 35 | HQPSASGGAAAAAAAPA | 0 | 1.98626E-05 | 0.002975279 |
| 36 | RPGDGGTASAGAAGPG | 0 | 0 | 0.00114783 |
| 37 | RPGDGGTASAGAAGPGAA | 0 | 0 | 0.005471188 |
| 38 | RPGDGGTASAGAAGPGAAT | 0 | 0 | 0.039806768 |
| 39 | RPGDGGTASAGAAGPGAATK | 0 | 0 | 0.000704115 |
| 40 | APQPAPGAAAASKP | 0 | 0 | 0.001162754 |
| 41 | RPGDGGTASAGAAGPGAATKA | 0 | 0 | 0.000661107 |
| 42 | EAAAGAGAAAPASQHP | 0 | 0 | 0.001720535 |
| 43 | EAAAGAGAAAPASQHPA | 0 | 0 | 0.001500257 |
| 44 | EEPAAAGSGAASPSAAE | 0 | 5.70432E-05 | 0.001433201 |
| 45 | EEPAAAGSGAASPSAAEK | 0 | 0 | 0.002124363 |
| 46 | EEPAAAGSGAASPSAAEKG | 0 | 0 | 0.001867428 |
| 47 | EPAAAGSGAASPSAAE | 0 | 1.62834E-05 | 0.043131975 |
| 48 | EPAAAGSGAASPSAAEK | 0 | 0 | 0.0097618 |
| 49 | EPAAAGSGAASPSAAEKG | 0 | 0 | 0.005832221 |
| 50 | EPAAAGSGAASPSAAEKGEP | 0 | 0 | 0.001447248 |
| 51 | EPAAAGSGAASPSAAEKGEPA | 0 | 0 | 0.003548931 |
| 52 | APAADKEEPAAAGSGAASPS | 0 | 8.45708E-05 | 0.000558279 |
| 53 | APAADKEEPAAAGSGAASPSAA | 0 | 0 | 0.000496532 |
| 54 | APAADKEEPAAAGSGAASPSAAEK | 0 | 0 | 0.000724574 |
| 55 | KEEPAAAGSGAASPS | 0 | 0 | 0.000641272 |
| 56 | APAAEGGKDEAAGGAAAAAAEAG | 0 | 0 | 0.003522139 |
| 57 | EAAGGAAAAAAEAG | 0 | 0 | 0.000113158 |
| 58 | EGGKDEAAGGAAAAAAEAG | 0 | 0 | 0.000972443 |
| 59 | GGKDEAAGGAAAAAAEAG | 0 | 0 | 0.007158668 |
| 60 | GKDEAAGGAAAAAAEAG | 0 | 4.22815E-05 | 0.007784253 |
| 61 | GKDEAAGGAAAAAAEA | 0 | 0 | 0.009308185 |
| 62 | KDEAAGGAAAAAAEA | 0 | 0 | 0.00431184 |
| 63 | GGKDEAAGGAAAAAAE | 0 | 0 | 0.000146001 |
| 64 | GKDEAAGGAAAAAAE | 0 | 0 | 0.001083218 |
| 65 | KDEAAGGAAAAAAE | 0 | 0 | 0.000290869 |
| 66 | AAAAAAAAAAAAAQ | 0 | 0 | 0.009000582 |
| 67 | AAAAAAAAAAAAAQQ | 0 | 0 | 0.000311371 |
| 68 | AAAAAAAAAAAAQ | 0 | 0 | 0.001139404 |
| 69 | AAPAAGSAPAAAE | 2.48968E-05 | 1.70282E-05 | 0.001824648 |
| 70 | APAGGPAPSTAAAPAEEK | 0 | 0.00014095 | 0 |
| 71 | APAGGPAPSTAAAPAEEKK | 0 | 0.000304147 | 0.000258853 |
| 72 | APGSAAPAAGSAPAAAEEKK | 0 | 2.03212E-05 | 0.030632201 |
| 73 | APGSAAPAAGSAPAAAEEKKD | 0 | 0 | 0.006860623 |
| 74 | APGSAAPAAGSAPAAAEEKKDE | 0 | 0 | 0.002981356 |
| 75 | APTTAATTAATAAQ | 0 | 0.000155952 | 0.000252532 |
| 76 | GSAAPAAGSAPAAAEEK | 0 | 0 | 0.002533859 |
| 77 | SAAPAAGSAPAAAEEK | 0 | 0 | 0.000899579 |
| 78 | SPGPGAGAPGAD | 0 | 0 | 0.001289447 |
| 79 | AAAAAAAAAAAAQQ | 0 | 0 | 0.017772214 |
| 80 | AAAAAAAAAAAQQ | 0 | 0 | 0.000758232 |
| 81 | AAPAAGSAPAAAEE | 0 | 4.12357E-05 | 0.008007834 |
| 82 | AAPAAGSAPAAAEEK | 0 | 0 | 0.007713698 |
| 83 | APAAGSAPAAAEE | 0 | 0 | 0.000626429 |
| 84 | APGSAAPAAGSAPAAAEEK | 0 | 0 | 0.076823055 |
| 85 | APAAGSAPAAAEEK | 0 | 0 | 0.004596178 |
| 86 | APAAGSAPAAAEEKK | 0 | 0 | 0.00131986 |
| 87 | APAAGSAPAAAEEKKD | 0 | 0 | 0.000264574 |
| 88 | GPAPSTAAAPAEEKK | 0 | 3.47666E-05 | 0 |
| 89 | APGSAAPAAGSAPAAAE | 0 | 4.45126E-05 | 0.004742807 |
| 90 | APGSAAPAAGSAPAAAEE | 0 | 6.07843E-05 | 0.032263288 |
| 91 | LPTASAGASTSASED | 0 | 0 | 0.003195567 |
| 92 | AAEGEAASAASSTSSP | 0 | 0 | 0.002514631 |
| 93 | AAEGEAASAASSTSSPK | 0 | 0 | 0.006949754 |
| 94 | AEGEAASAASSTSSP | 0 | 0 | 0.002199477 |
| 95 | EGEAASAASSTSSP | 0 | 0 | 0.002049777 |
| 96 | EPGSPTAAEGEAASAASSTSSPK | 0 | 0 | 0.015101347 |
| 97 | SPTAAEGEAASAASSTSSPK | 0 | 0 | 0.002550212 |
| 98 | SPTAAEGEAASAASSTSSP | 0 | 0 | 0.005162211 |
| 99 | EPGSPTAAEGEAASAA | 0 | 0 | 0.016504188 |
| 100 | EPGSPTAAEGEAASAAS | 0 | 0.000146635 | 0.003543344 |
| 101 | EPGSPTAAEGEAASAASS | 0 | 0 | 0.013845913 |
| 102 | EPGSPTAAEGEAASAASST | 0 | 0 | 0.003680203 |
| 103 | EPGSPTAAEGEAASAASSTS | 0 | 0 | 0.000577916 |
| 104 | EPGSPTAAEGEAASAASSTSSP | 0 | 0 | 0.012727659 |
| 105 | DPAAAAAATAAPGQTP | 4.14184E-05 | 2.88578E-05 | 0.002725621 |
| 106 | DPAAAAAATAAPGQTPA | 0 | 0 | 0.001452912 |
| 107 | SPGPGAGAPGADA | 0 | 0 | 0.006645494 |
| 108 | RSPGPGAGAPGADA | 0 | 0 | 0.001794865 |
| 109 | GPASAGGSAEALLS | 0 | 0 | 0.001834945 |
| 110 | SPGPASAGGSAEALLS | 0 | 0 | 0.023013815 |
| 111 | SPGPASAGGSAEALLSD | 0 | 0 | 0.004435966 |
| 112 | SPGPASAGGSAEALLSDL | 0 | 0 | 0.002225496 |
| 113 | SPGPASAGGSAEAL | 0 | 2.52697E-05 | 0.000948539 |
| 114 | SPPPAATAAAATADV | 0 | 0 | 0.002332524 |
| 115 | SPPPAATAAAATADVT | 0.002740806 | 0 | 0.031049567 |
| 116 | SPPPAATAAAATADVTL | 0 | 0 | 0.002967789 |
| 117 | AARAAAAAAAAAA | 0 | 0 | 0.008492266 |
| 118 | AARAAAAAAAAAAA | 0 | 0 | 0.008956907 |
| 119 | APGTKGTAAAAAAAAA | 0 | 0.000258147 | 0.001131643 |
| 120 | APGTKGTAAAAAAAAAA | 0 | 0 | 0.005048986 |
| 121 | PGTKGTAAAAAAAAAA | 0 | 0 | 0.000350033 |
| 122 | HPDPRAPGAAAPSSGPGP | 0 | 0 | 0.008035124 |
| 123 | KASASAPAPASAT | 0 | 0 | 0.00312656 |
| 124 | TKGTAAAAAAAAAA | 0 | 5.64641E-05 | 0.001019829 |
| 125 | ADKASASAPAPA | 0.001240333 | 0 | 0.002856825 |
| 126 | ADKASASAPAPAS | 0 | 0 | 0.026113679 |
| 127 | ADKASASAPAPASA | 0 | 0 | 0.098492945 |
| 128 | ADKASASAPAPASAT | 1.00897E-05 | 0 | 0.169704529 |
| 129 | ADKASASAPAPASATE | 0 | 0 | 0.004021391 |
| 130 | ADKASASAPAPASATEIL | 0 | 0 | 0.000899317 |
| 131 | DKADKASASAPAPASATEIL | 0 | 0 | 0.000689452 |
| 132 | GDKADKASASAPAPASATEIL | 0.000154337 | 0 | 0.005783781 |
| 133 | DKASASAPAPASA | 0 | 0 | 0.000714358 |
| 134 | DKASASAPAPASAT | 0 | 0 | 0.003705278 |
| 135 | DKASASAPAPASATE | 0 | 0 | 0.000685397 |
| 136 | KADKASASAPAPASATE | 0 | 2.60073E-05 | 0.001941876 |
| 137 | KASASAPAPASA | 0 | 0 | 0.00057583 |
| 138 | HPHPPAAGAGAAGGAE | 0 | 0 | 0.000341475 |
| 139 | KASASAPAPASATE | 0 | 0 | 0.001030658 |
| 140 | RSPGPGAGAPGAD | 0 | 0 | 0.000810747 |
| 141 | DKADKASASAPAPA | 0 | 0 | 0.002877009 |
| 142 | DKADKASASAPAPAS | 1.31922E-05 | 0 | 0.034409603 |
| 143 | DKADKASASAPAPASA | 0 | 0 | 0.104664583 |
| 144 | DKADKASASAPAPASAT | 0 | 0 | 0.228572863 |
| 145 | DKADKASASAPAPASATE | 0 | 0 | 0.012932879 |
| 146 | GDKADKASASAPAPASATE | 0 | 0 | 0.020682017 |
| 147 | GIKGDKADKASASAPAPASATE | 0.000136976 | 0 | 0.010412365 |
| 148 | IKGDKADKASASAPAPASATE | 0 | 0 | 0.008076009 |
| 149 | KADKASASAPAPA | 0 | 6.15247E-07 | 0.001242874 |
| 150 | KGDKADKASASAPAPASATE | 0 | 0 | 0.000481444 |
| 151 | KADKASASAPAPASAT | 0 | 0 | 0.054522425 |
| 152 | GDKADKASASAPAPA | 0 | 0 | 0.001587528 |
| 153 | GDKADKASASAPAPAS | 1.26027E-05 | 0 | 0.027375034 |
| 154 | GDKADKASASAPAPASA | 0 | 7.41927E-06 | 0.057947742 |
| 155 | GDKADKASASAPAPASAT | 0 | 0 | 0.120824411 |
| 156 | GIKGDKADKASASAPAPASAT | 0 | 0 | 0.011484689 |
| 157 | IKGDKADKASASAPAPASAT | 0 | 0 | 0.01079424 |
| 158 | KGDKADKASASAPAPASAT | 0 | 0 | 0.013774102 |
| 159 | GIKGDKADKASASAPAPAS | 3.9483E-06 | 0 | 0.001996578 |
| 160 | GIKGDKADKASASAPAPASA | 0 | 0 | 0.009958589 |
| 161 | IKGDKADKASASAPAPAS | 0 | 0 | 0.005006376 |
| 162 | KADKASASAPAPAS | 0 | 0 | 0.012482468 |
| 163 | KGDKADKASASAPAPAS | 0 | 0 | 0.003341705 |
| 164 | IKGDKADKASASAPAPASA | 0 | 0 | 0.007694921 |
| 165 | KADKASASAPAPASA | 0 | 0 | 0.028706987 |
| 166 | KGDKADKASASAPAPASA | 0 | 0 | 0.009580027 |
| 167 | SERTPGAATASASGAA | 0 | 0 | 0.000930665 |
| 168 | SNRAASAAGAAGSAGG | 0 | 0 | 0.000333221 |
| 169 | SNRAASAAGAAGSAGGS | 0 | 0 | 0.000117371 |
| 170 | ASAPAPASATEIL | 0 | 0.00029802 | 0.006458079 |
| 171 | DKASASAPAPASATEIL | 0 | 0.00031458 | 0.002790566 |
| 172 | SASAPAPASATEIL | 0.004169211 | 0 | 0.016304332 |
| 173 | LPSSARAGAGSGSSPG | 0 | 0 | 0.002747746 |
| 174 | LPSSARAGAGSGSSPGGQ | 0 | 0 | 0.001385652 |
| 175 | GPGPGGPGGAGVARGGAG | 0 | 0 | 0.001206472 |
| 176 | GPGPGGPGGAGVARGGAGGGPS | 0 | 0 | 0.000777867 |
| 177 | AGPGPAAGAALPDQSF | 0 | 0 | 0.001352078 |
| 178 | GPGPAAGAALPDQS | 0 | 2.33306E-05 | 0.0015594 |
| 179 | GPGPAAGAALPDQSF | 0 | 0 | 0.006388948 |
| 180 | RPGPGAGPGPAAGAALPDQSF | 0 | 0 | 0.004181353 |
| 181 | RPGPGAGPGPAAGAALPDQ | 0 | 0 | 0.000760115 |
| 182 | DLVSENAGRAAAAAAA | 0 | 0 | 0.002893442 |
| 183 | ENAGRAAAAAAA | 0 | 0 | 0.000604473 |
| 184 | LVSENAGRAAAAAAA | 0 | 0 | 0.007149856 |
| 185 | LVSENAGRAAAAAA | 0 | 0 | 0.00120201 |
| 186 | ENAGRAAAAAAAAA | 0 | 0 | 0.002261624 |
| 187 | NAGRAAAAAAAAA | 0 | 0 | 0.002461891 |
| 188 | NAGRAAAAAAAAAA | 0 | 0 | 0.002844747 |
| 189 | NAGRAAAAAAAAAAA | 0 | 0 | 0.001954356 |
| 190 | AAAAAAAAAAKAA | 9.48431E-05 | 0 | 0.001692027 |
| 191 | APPSPGAAPAPAGRPP | 0 | 0 | 0.001803517 |
| 192 | APTSGTSAKTP | 0 | 0 | 0.000396356 |
| 193 | DAGAPTAPAASATRAAQVR | 0 | 0.026603954 | 3.39881E-05 |
| 194 | DAGAPTAPAASATRAAQVRR | 0 | 0.00095011 | 0 |
| 195 | APTSGTSAKTPSE | 0 | 0 | 0.000708375 |
| 196 | APTSGTSAKTPSEG | 0.000102031 | 0 | 0.001149718 |
| 197 | LAPTSGTSAKTPSEG | 0 | 0 | 0.000793507 |
| 198 | VDLAPTSGTSAKTPSEG | 0 | 0 | 0.002082522 |
| 199 | LAPTSGTSAKTPSE | 0 | 4.53971E-06 | 0.00127503 |
| 200 | VDLAPTSGTSAKTPSE | 0 | 0 | 0.002193136 |
| 201 | AEGEAASAASSTSSPK | 0 | 0 | 0.002011917 |
| 202 | DAASAASAASSAH | 0 | 0 | 0.00043437 |
| 203 | EGEAASAASSTSSPK | 0 | 0 | 0.000672004 |
| 204 | EDAASAASAASSAH | 0 | 0 | 0.001234983 |
| 205 | LEDAASAASAASSAH | 0 | 0 | 0.000629259 |
| 206 | DAASAASAASSAHVA | 0 | 0 | 0.000352208 |
| 207 | APAAAAAPAKVEAK | 0 | 0 | 0.000149291 |
| 208 | APAAAAAPAKVEAKE | 0 | 0 | 0.000112917 |
| 209 | ASPGSAASPRL | 2.7194E-05 | 0.003923231 | 0 |
| 210 | ASPASSTTARVIT | 0 | 0 | 0.000407266 |
| 211 | ASPASSTTARVITN | 0 | 0 | 0.002521975 |
| 212 | ARASPGSAASPRLR | 0 | 0.000459055 | 0 |
| 213 | ASPGSAASPRLR | 0 | 0.009656474 | 0.001660694 |
| 214 | GARASPGSAASPRLR | 0 | 0.001136352 | 7.86671E-06 |
| 215 | GARASPGSAASPRLREGPE | 0 | 9.10377E-05 | 0 |
| 216 | PGSAASPRLR | 0 | 0.000895784 | 0 |
| 217 | RASPGSAASPRLR | 0 | 0.00144088 | 0 |
| 218 | SPGSAASPRLR | 0 | 0.003076728 | 0 |
| 219 | IPSAAAAAAAAGRIA | 0 | 0 | 0.001402241 |
| 220 | IPSAAAAAAAAGRIAIPG | 0 | 0 | 0.000897467 |
| 221 | AGGGPGGGRPGAGTP | 0 | 0 | 0.00093948 |
| 222 | AGGGPGGGRPGAGTPL | 0 | 0 | 0.001739315 |
| 223 | GGGPGGGRPGAGTP | 8.18936E-06 | 0 | 0.001579607 |
| 224 | GPGGGRPGAGTP | 0 | 0 | 0.005242481 |
| 225 | GGGPGGGRPGAGTPL | 0 | 0 | 0.0117417 |
| 226 | GGPGGGRPGAGTPL | 0 | 0 | 0.013550885 |
| 227 | GPGGGRPGAGTPL | 0 | 0 | 0.012734022 |
| 228 | GGPGGGRPGAGTPLR | 0 | 0 | 0.000482518 |
| 229 | GPGGGRPGAGTPLR | 0 | 0 | 0.001699696 |
| 230 | APEPSSPGAARAAAAAAR | 0 | 0 | 0.001827684 |
| 231 | PAKGGAPAPSR | 0 | 0 | 0.027122316 |
| 232 | PAKGGAPAPSRGPQ | 0 | 0 | 0.000679328 |
| 233 | RVPAKGGAPAPSRGPQ | 0 | 0 | 0.025610339 |
| 234 | RVPAKGGAPAPSRGPQE | 0 | 0 | 0.000301527 |
| 235 | SRVPAKGGAPAPSRGPQE | 0 | 0 | 0.000483001 |
| 236 | VPAKGGAPAPSRGPQ | 0 | 0 | 0.370213875 |
| 237 | VPAKGGAPAPSRG | 0 | 1.28511E-06 | 0.005451515 |
| 238 | VPAKGGAPAPSRGP | 0 | 0 | 0.042224149 |
| 239 | VPAKGGAPAPSRGPQE | 0 | 0 | 0.017056733 |
| 240 | RVPAKGGAPAPSR | 0 | 0 | 0.005246232 |
| 241 | RVPAKGGAPAPSRGP | 0 | 0 | 0.002388402 |
| 242 | SRVPAKGGAPAPSRGPQ | 0 | 0 | 0.021977419 |
| 243 | VPAKGGAPAPS | 0 | 0 | 0.001151549 |
| 244 | VPAKGGAPAPSR | 0 | 1.79995E-05 | 0.104620486 |
| 245 | SRVPAKGGAPAPSR | 0 | 0 | 0.002869233 |
| 246 | VSGARASPGSAASPR | 0 | 0 | 0.000589874 |
| 247 | APGHPLAPGPHPAAPSSWGP | 0 | 0 | 0.009273109 |
| 248 | APGHPLAPGPHPAAPSSWGPR | 0 | 0 | 0.001420855 |
| 249 | APGHPLAPGPHPAAPSSWGPRP | 0 | 0 | 0.00439928 |
| 250 | APGHPLAPGPHPAAPSSWGPRPR | 0 | 0 | 0.003127783 |
| 251 | APGPHPAAPSSWGP | 0 | 0 | 0.009481017 |
| 252 | HPLAPGPHPAAPSSWGPRP | 0 | 0 | 0.004163994 |
| 253 | APGPHPAAPSSWGPR | 0 | 0 | 0.002919497 |
| 254 | APGPHPAAPSSWGPRP | 0 | 0 | 0.008907339 |
| 255 | APGPHPAAPSSWGPRPR | 0 | 6.94123E-06 | 0.002204215 |
| 256 | HPLAPGPHPAAPSSWGPRPR | 0 | 3.39049E-05 | 0.003766996 |
| 257 | GPPAAAPGHPLAPGPHPAAPSSWGP | 0 | 0 | 0.003045829 |
| 258 | LPAGPRPSTPGRA | 0 | 0 | 0.011687667 |
| 259 | AGPRPSTPGRAH | 0 | 0 | 0.000446042 |
| 260 | GPRPSTPGRAH | 0 | 0 | 0.010313778 |
| 261 | LPAGPRPSTPGRAH | 0 | 0 | 0.077544933 |
| 262 | LPAGPRPSTPGRAHL | 0 | 0 | 0.072397016 |
| 263 | LPAGPRPSTPGRAHLV | 0 | 0 | 0.005751175 |
| 264 | LPAGPRPSTPGRAHLVS | 0 | 0 | 0.007358929 |
| 265 | PAGPRPSTPGRAH | 0 | 0 | 0.003848032 |
| 266 | GPRPSTPGRAHL | 0 | 0 | 0.009298669 |
| 267 | GPRPSTPGRAHLVS | 0 | 0 | 0.000346452 |
| 268 | APSSKSTSGGTAAL | 0 | 0 | 0.000631225 |
| 269 | APSSKSTSGGTAALG | 0 | 0 | 0.236941669 |
| 270 | APSSKSTSGGTAALGC | 0 | 0 | 0.006794288 |
| 271 | APSSKSTSGGTAALGCL | 0 | 0 | 0.005801838 |
| 272 | FPLAPSSKSTSGGTAA | 0 | 0 | 0.026491296 |
| 273 | FPLAPSSKSTSGGTAALG | 0 | 0 | 0.050345036 |
| 274 | GPSVFPLAPSSKSTSGGTAALG | 0 | 0 | 0.242383036 |
| 275 | PLAPSSKSTSGGTAA | 0 | 0 | 0.015676416 |
| 276 | GPSVFPLAPSSKSTSGGTAA | 0 | 0 | 0.055117244 |
| 277 | VFPLAPSSKSTSGGTAA | 0 | 0 | 0.003753671 |
| 278 | PLAPSSKSTSGGTAALG | 0 | 0 | 0.076589261 |
| 279 | AGGGGGGAAAAGRAY | 0 | 4.598E-05 | 0.001668402 |
| 280 | GGAAAAGRAY | 0 | 0 | 0.004608635 |
| 281 | GGGAAAAGRAY | 0 | 0 | 0.001336726 |
| 282 | GGGGAAAAGRAY | 0 | 0 | 0.000571814 |
| 283 | GGGGGGAAAAGRAY | 0 | 0 | 0.000824474 |
| 284 | GGAAAAGRAYS | 0 | 0 | 0.000733138 |
| 285 | GGAAAAGRAYSFK | 0 | 0 | 0.003741299 |
| 286 | GGAAAAGRAYSFKV | 0.000947103 | 0 | 0.000542164 |
| 287 | APGTAAGRARRCAGFL | 0 | 1.74849E-05 | 0.001933977 |
| 288 | GPGAPGTAAGRARRCAGFL | 0 | 0.000211349 | 0.046375891 |
| 289 | PGTAAGRAR | 0 | 0 | 0.017202723 |
| 290 | GAPGTAAGRAR | 0 | 0 | 0.009599143 |
| 291 | GPAAGPGAPGTAAGRAR | 0 | 0 | 0.002618793 |
| 292 | GPGAPGTAAGRAR | 0 | 0 | 0.11480606 |
| 293 | PGAPGTAAGRAR | 0 | 0 | 0.004508298 |
| 294 | GPGAPGTAAGRARR | 0 | 0 | 0.015821417 |
| 295 | GPGAPGTAAGRARRC | 0 | 0 | 2.9771E-05 |
| 296 | GPGAPGTAAGRARRCAG | 0 | 0 | 0.00107517 |
| 297 | GPGAPGTAAGRARRCAGF | 0 | 2.64348E-05 | 0.001112305 |
| 298 | VPRKVDGGGTSAASKP | 0 | 0 | 0.003167945 |
| 299 | VPRKVDGGGTSAASKPK | 0 | 0 | 0.000790723 |
| 300 | LVSSKPQAHGAPAPPSGSAPH | 0 | 0 | 0.002375405 |
| 301 | APVAAATTAAPAAAAAPA | 0 | 8.59558E-05 | 0.014272116 |
| 302 | AAAAAAAGAGAGAKQTPA | 0 | 0 | 0.001066808 |
| 303 | AAAAAAGAGAGAKQTPA | 0 | 0 | 0.001443031 |
| 304 | APGAPAAAPPAKEIPE | 0 | 0 | 0.000379893 |
| 305 | AVSGARASPGSAA | 0 | 0 | 0.002181143 |
| 306 | AVSGARASPGSAASP | 0 | 0 | 0.000403319 |
| 307 | WAVSGARASPGSAA | 0 | 0 | 0.001361138 |
| 308 | AGSGAGGRAAPVE | 0 | 0 | 0.000465345 |
| 309 | LPPGGGAARAGPG | 0 | 0 | 0.004608729 |
| 310 | TPDSIASSSSAAHPP | 0 | 0 | 0.000387268 |
| 311 | TPDSIASSSSAAHPPG | 0 | 6.60703E-05 | 0.00350169 |
| 312 | DIGGDAGGGTAGKSGP | 0 | 0 | 0.001483797 |
| 313 | DIGGDAGGGTAGKSGPR | 0 | 0 | 0.000581888 |
| 314 | IGGDAGGGTAGKSGP | 0 | 0 | 0.002740435 |
| 315 | IGGDAGGGTAGKSGPR | 0 | 0 | 0.003725028 |
| 316 | QPPSPQPGPPAASHTP | 1.0095E-05 | 0.000103215 | 0.002177055 |
| 317 | QPPSPQPGPPAASHTPE | 0 | 0 | 0.001201242 |
| 318 | GPGAATRGSGVGECG | 0 | 0 | 0.000824754 |
| 319 | IQSTPGSGHAPRTSSS | 0 | 0 | 0.00037772 |
| 320 | AGPHSAASEGTVQ | 0 | 0 | 0 |
| 321 | AGPHSAASEGTVQR | 0 | 0 | 6.2904E-05 |
| 322 | APAGSHAAPAQPGP | 0 | 0 | 0.014057051 |
| 323 | APAGSHAAPAQPGPT | 0 | 0 | 0.00598461 |
| 324 | APAGSHAAPAQPGPTS | 0 | 0 | 0.001869745 |
| 325 | FGAPAGSHAAPAQPGPT | 0 | 0 | 0.009054875 |
| 326 | GFGAPAGSHAAPAQPGPT | 0 | 0 | 0.000968912 |
| 327 | PDPDPEPAGGSRPGPAVP | 0 | 0 | 0.000945879 |
| 328 | PDPEPAGGSRPGPAVP | 0 | 0 | 0.004336569 |
| 329 | PEPAGGSRPGPAVP | 1.55419E-05 | 0 | 0.001567679 |
| 330 | PDPEPAGGSRPGPAVPG | 1.2967E-05 | 0 | 0.004114383 |
| 331 | AAYYSAAGPRPGADR | 0 | 7.69617E-05 | 0.002930423 |
| 332 | AAYYSAAGPRPGADRH | 0 | 4.95974E-05 | 0.004521125 |
| 333 | APAGGKASWGADR | 0 | 0 | 0.000573811 |
| 334 | APAGGKASWGADRS | 0 | 0 | 0.000513545 |
| 335 | SSAPPRTAF | 0 | 0.000697283 | 0 |
| 336 | AQRDAWGPGAAKSGVGTA | 0 | 4.57982E-05 | 0.005609632 |
| 337 | AWGPGAAKSGVGT | 0 | 0 | 0.03870533 |
| 338 | AWGPGAAKSGVGTA | 0 | 0 | 0.048075945 |
| 339 | DAWGPGAAKSGVGTA | 0 | 0 | 0.145657497 |
| 340 | GPGAAKSGVGT | 0 | 0 | 0.003733824 |
| 341 | GPGGPRGGLGG | 0 | 0 | 0.000348822 |
| 342 | QRDAWGPGAAKSGVGTA | 6.32413E-05 | 0 | 0.034238837 |
| 343 | RDAWGPGAAKSGVGTA | 0 | 0 | 0.094743667 |
| 344 | WGPGAAKSGVGT | 0 | 0 | 0.044433788 |
| 345 | RDAWGPGAAKSGVGTAL | 0 | 0 | 0.012704223 |
| 346 | WGPGAAKSGVGTA | 0 | 3.05811E-05 | 0.029452035 |
| 347 | DAWGPGAAKSGVG | 0 | 0 | 0.037932911 |
| 348 | DAWGPGAAKSGVGT | 0 | 0 | 0.152077497 |
| 349 | QRDAWGPGAAKSGVGT | 0 | 1.25553E-05 | 0.039763926 |
| 350 | QRDAWGPGAAKSGVGTAL | 0 | 0 | 0.002678369 |
| 351 | RDAWGPGAAKSGVGT | 0 | 6.49775E-06 | 0.146532313 |
| 352 | AWGPGAAKSGVGTAL | 0 | 0 | 0.067882447 |
| 353 | DAWGPGAAKSGVGTAL | 0 | 0 | 0.264500886 |
| 354 | DAWGPGAAKSGVGTALL | 0 | 0 | 0.018979444 |
| 355 | GPGAAKSGVGTA | 0 | 0 | 0.001177676 |
| 356 | GPGAAKSGVGTAL | 0 | 0 | 0.002443411 |
| 357 | WGPGAAKSGVGTAL | 0 | 0 | 0.035065564 |
| 358 | QRDAWGPGAAKSGVG | 0 | 6.15159E-05 | 0.050454755 |
| 359 | RDAWGPGAAKSGVG | 0 | 0 | 0.145249439 |
| 360 | SLDSPTSGRPGVTS | 0 | 1.2742E-05 | 0.000938655 |
| 361 | DQWSSSSPHSN | 0 | 0 | 0.000491989 |
| 362 | DQWSSSSPHSNVS | 0 | 0 | 0.000469325 |
| 363 | SPDQWSSSSPHSNVS | 0 | 0.00014998 | 0.022561368 |
| 364 | SPDQWSSSSPHSNVSD | 0 | 0 | 0.015954997 |
| 365 | SPESPDQWSSSSPHSNVSD | 0 | 0 | 0.002642944 |
| 366 | TPSPESPDQWSSSSPHSNVSD | 0 | 0 | 0.076989154 |
| 367 | TPSPESPDQWSSSSPHSNVSDW | 0 | 0 | 0.007880886 |
| 368 | TPSPESPDQWSSSSPHSNVSDWS | 0 | 0 | 0.005804002 |
| 369 | SPESPDQWSSSSPHSNVS | 0 | 0 | 0.002120171 |
| 370 | PSPESPDQWSSSSPHSN | 0 | 3.35236E-05 | 0.00014311 |
| 371 | SPDQWSSSSPHSN | 0 | 0 | 0.036255592 |
| 372 | SPESPDQWSSSSPHSN | 0 | 0 | 0.00844234 |
| 373 | TPSPESPDQWSSSSPHSN | 0 | 8.38269E-05 | 0.11324801 |
| 374 | TPSPESPDQWSSSSPHSNV | 0 | 0 | 0.001876691 |
| 375 | TPSPESPDQWSSSSPHSNVS | 0 | 0 | 0.052486593 |
| 376 | TPSPESPDQWSSSSPHSA | 0 | 0 | 0.000728753 |
| 377 | TPSPESPDQWSSSSPHSASD | 0 | 0 | 0.001603044 |
| 378 | TPSPESPDQWSSSSPHSASDWS | 0 | 0 | 0.003787463 |
| 379 | EIQDWSASSPHSAA | 0 | 0 | 0.000277543 |
| 380 | IQDWSASSPHSA | 0 | 0 | 0.000843814 |
| 381 | IQDWSASSPHSAA | 0 | 0 | 0.001740851 |
| 382 | GPYPGPGGHTATVLVPSG | 0 | 0.006924923 | 0.000402638 |
| 383 | TPGPYPGPGGHTATVLVPSG | 0 | 0.000877881 | 0 |
| 384 | VPGAAASRTSEIEG | 0 | 0 | 0.000654341 |
| 385 | ADYSWYQAGSARSGP | 0 | 8.93744E-05 | 0.002701216 |
| 386 | ADYSWYQAGSARSGPV | 0 | 0 | 0.000566584 |
| 387 | DYSWYQAGSARSGP | 0 | 0 | 0.017206664 |
| 388 | WYQAGSARSGP | 0 | 0 | 0 |
| 389 | YQAGSARSGP | 0 | 0 | 0.000430945 |
| 390 | DYSWYQAGSARSGPV | 0 | 0 | 0.003166985 |
| 391 | DYSWYQAGSARSGPVT | 0 | 0 | 0.010265007 |
| 392 | YQAGSARSGPVT | 0 | 0 | 0.000688371 |
| 393 | DPGASRGGTTGETVVG | 0 | 0 | 0.007110903 |
| 394 | VPAAARAGAIAPCE | 0 | 0 | 0.001484591 |
| 395 | VPAAARAGAIAPCEVT | 0 | 0 | 0.001316283 |
| 396 | APRLLIYGASSR | 0 | 0 | 0.000611296 |
| 397 | APRLLIYGASSRATGIP | 0 | 0 | 0.00085955 |
| 398 | APRLLIYGASSRATGIPD | 0 | 0 | 0.008291931 |
| 399 | KPGQAPRLLIYGASSRATGIPD | 0 | 0 | 0.000894727 |
| 400 | KPGQAPRLLIYGASSR | 0 | 0 | 0.001597905 |
| 401 | GQAPRLLIY | 0 | 0 | 0.001658511 |
| 402 | KPGQAPRLLIYDASNRATGIP | 7.24689E-05 | 0 | 0.001075962 |
| 403 | AEEAAGASPAKANGQE | 0 | 0 | 0.000206555 |
| 404 | APRGDVTAEEAAGASP | 0 | 0 | 0 |
| 405 | APRGDVTAEEAAGASPA | 0.001262314 | 0 | 0.000383892 |
| 406 | APRGDVTAEEAAGASPAK | 0 | 0 | 0.000772344 |
| 407 | DVTAEEAAGASPAK | 0 | 7.59907E-06 | 0.004191113 |
| 408 | GDVTAEEAAGASPAK | 0.00020095 | 0 | 0.001496373 |
| 409 | VTAEEAAGASPAK | 0 | 0 | 0.001058638 |
| 410 | AGVETTTPSKQ | 0 | 0.001619236 | 0 |
| 411 | AGVETTTPSKQSN | 0 | 0.000627661 | 0 |
| 412 | AGVETTTPSKQSNN | 0 | 0.001363059 | 0 |
| 413 | AGVETTTPSKQSNNK | 7.92927E-06 | 0.002081833 | 0 |
| 414 | GVETTTPSKQ | 0 | 0.002041573 | 0 |
| 415 | SPVKAGVETTTPSKQSNNK | 0 | 0.005699782 | 0 |
| 416 | SPVKAGVETTTPSKQSNNKY | 0 | 0.000610381 | 0 |
| 417 | VETTTPSKQ | 0 | 0.000449607 | 0 |
| 418 | GVETTTPSKQSN | 0 | 0.000554865 | 0 |
| 419 | SPVKAGVETTTPSKQSN | 0 | 0.00449701 | 0 |
| 420 | SPVKAGVETTTPSKQSNN | 0 | 0.005378551 | 0 |
| 421 | SPVKAGVETTTPS | 0 | 0.000573775 | 0 |
| 422 | SPVKAGVETTTPSKQ | 0 | 0.013100389 | 1.54279E-05 |
| 423 | SPVKAGVETTTPSKQS | 0 | 0.000726112 | 0 |
| 424 | DETTPTSTRADVE | 2.9046E-05 | 0.000812096 | 1.21134E-05 |
| 425 | DETTPTSTRADVEASPG | 0 | 0.000167833 | 0 |
| 426 | ETTPTSTRADVE | 0 | 0.000114678 | 0 |
| 427 | GLDETTPTSTRADVE | 0 | 0.000248445 | 8.40755E-05 |
| 428 | MDGGGSPKGDVD | 0 | 9.60166E-05 | 0.001307889 |
| 429 | TGLSMDGGGSPKGDVD | 0 | 0.000636618 | 0.001116685 |
| 430 | VESTGTAKAEAESR | 0 | 1.69167E-05 | 0 |
| 431 | VESTGTAKAEAESRA | 0 | 0.000131487 | 0 |
| 432 | APSDPATTTAKADAASS | 0 | 0.001022325 | 0.000119893 |
| 433 | APSDPATTTAKADAASSLTVD | 0 | 0 | 0.001361574 |
| 434 | DTPDAATTSRSDQ | 0 | 0 | 0.000304509 |
| 435 | DTPDAATTSRSDQL | 0 | 6.92636E-05 | 0.001036542 |
| 436 | DTPDAATTSRSDQLTPQ | 5.98585E-05 | 0.000885843 | 0 |
| 437 | TPDAATTSRSDQL | 0 | 0.000531527 | 3.36145E-05 |
| 438 | TPDAATTSRSDQLTPQ | 0.000133174 | 0.008238731 | 0.000395977 |
| 439 | TPDAATTSRSDQLTPQG | 0 | 0.000794747 | 0 |
| 440 | GFGDGGPSGASGRDN | 0 | 0 | 0.000224715 |
| 441 | DPEEGAAASISQ | 0 | 0 | 0.003467448 |
| 442 | DPEEGAAASISQE | 0 | 0 | 0.001920332 |
| 443 | DPEEGAAASISQEP | 0.000451879 | 0.000358414 | 0.004830695 |
| 444 | DPEEGAAASISQEPS | 0.000254394 | 0.001331768 | 0.00857706 |
| 445 | VEDPEEGAAASISQEPS | 0 | 0 | 0.006036962 |
| 446 | LPGTAVPSVPEDAAPAS | 0 | 0.002131036 | 0.002454942 |
| 447 | LPGTAVPSVPEDAAPASR | 0 | 0.000724035 | 0.000655591 |
| 448 | LPGTAVPSVPEDAAPASRDG | 0 | 0.00115029 | 0.000199175 |
| 449 | VPSVPEDAAPAS | 0 | 0.000366766 | 0 |
| 450 | VPSVPEDAAPASR | 0 | 0.000736701 | 0 |
| 451 | VPSVPEDAAPASRDG | 0 | 0.001538586 | 6.13583E-05 |
| 452 | VPSVPEDAAPASRDGGGVR | 0 | 0.000111022 | 0 |
| 453 | MRYVASYL | 0.001187517 | 9.83299E-05 | 0.000318524 |
| 454 | MRYVASYLL | 0.013943859 | 0.003964687 | 0.006500668 |
| 455 | MRYVASYLLAALG | 0.000613951 | 0 | 0 |
| 456 | MRYVASYLLAALGGNSSPSAKD | 0.003358925 | 0.000572508 | 0 |
| 457 | ALQNIIPASTGAAK | 0 | 0 | 0.006197127 |
| 458 | LQNIIPASTGAAK | 0 | 0 | 0.000191191 |
| 459 | QNIIPASTGAAKA | 0 | 0 | 0.000175554 |
| 460 | APGPHAMPVAPGP | 0 | 0 | 0 |
| 461 | DPGTTPPGHSVPVPAT | 0 | 0 | 0.002807969 |
| 462 | DPGTTPPGHSVPVPATE | 0 | 3.28662E-05 | 0.017161866 |
| 463 | DPGTTPPGHSVPVPATEL | 0 | 0 | 0.004514749 |
| 464 | DPGTTPPGHSVPVPATELG | 0 | 0 | 0.011915592 |
| 465 | PGTTPPGHSVPVPATE | 0 | 0 | 0.00154199 |
| 466 | TTPPGHSVPVPATE | 0 | 0 | 0.000696173 |
| 467 | IPASTGAAKAVG | 0.000414218 | 0.000125031 | 0.002017971 |
| 468 | IPASTGAAKAVGK | 0 | 0 | 0.002383603 |
| 469 | PASTGAAKAVGKVIPEL | 0 | 0 | 0.001789324 |
| 470 | APRSVAVSSIAGSTE | 8.16768E-05 | 2.03153E-05 | 0.004270387 |
| 471 | APRSVAVSSIAGSTEL | 0 | 0 | 0.002767587 |
| 472 | DPSRAIPLGT | 0.00046084 | 0 | 0.001553251 |
| 473 | DPSRAIPLGTIV | 0 | 0 | 0.00574043 |
| 474 | SPRGKNIQGGKTLSVSQ | 0 | 0 | 0.000453998 |
| 475 | APSAARSLPSAST | 0 | 0 | 0.003451685 |
| 476 | TPGEPAASSSRPVAPS | 0 | 0 | 0.001716084 |
| 477 | APGHAVPPAVK | 0 | 0 | 0.000459615 |
| 478 | DYEYDWAGKPLPTPIG | 0 | 0 | 0.00728748 |
| 479 | YDWAGKPLPTPIG | 5.79859E-05 | 0 | 0.001990172 |
| 480 | YEYDWAGKPLPTPIG | 0 | 0.000336802 | 0.005960349 |
| 481 | GPPKPLPAGRISRSISE | 0 | 0 | 0.002651418 |
| 482 | GPPKPLPAGRISRSISES | 0 | 0 | 0.014081289 |
| 483 | DPEPDQQDPTLGGPARAAEE | 0 | 0 | 0.005113438 |
| 484 | DPEPDQQDPTLGGPARAAEEV | 0 | 0 | 0.001410227 |
| 485 | DPTLGGPARAAEE | 0 | 0 | 0.001151937 |
| 486 | QQDPTLGGPARAAEE | 0 | 0 | 0.00214472 |
| 487 | DPTLGGPARAAEEV | 0 | 0 | 0.001422117 |
| 488 | GPARAAEEVM | 0 | 0 | 0 |
| 489 | FIAPTGHSL | 0 | 0 | 0.004528009 |
| 490 | YVTTSTRTY | 0 | 0.005240142 | 0 |
| 491 | TSIPAAAHTVSN | 0 | 0 | 0.001932706 |
| 492 | TSIPAAAHTVSNAPV | 0 | 0 | 0.003006272 |
| 493 | APTPARPVLTH | 0 | 0 | 0.007113703 |
| 494 | APTPARPVLTHL | 0 | 0 | 0.001309932 |
| 495 | IEGAFSGSGAKTVIPR | 0 | 0 | 0.002166003 |
| 496 | GPPGTGKTLIARAVANETG | 0 | 0 | 0.005670912 |
| 497 | GPPGTGKTLIARAVANETGAF | 0 | 0 | 0.004252469 |
| 498 | TPAAPPKAVLKLEPQWINVLQED | 0.001288012 | 0 | 0 |
| 499 | DISSSTSRTMESESL | 0 | 0.001692207 | 0 |
| 500 | DISSSTSRTMESESLR | 1.80808E-05 | 0.016114218 | 0.000776144 |
| 501 | DISSSTSRTMESESLRT | 0 | 0.004434435 | 0.00031769 |
| 502 | DISSSTSRTMESESLRTLE | 0 | 0.006687895 | 0.000160807 |
| 503 | ISSSTSRTMESESL | 0 | 0.001190343 | 0 |
| 504 | SSSTSRTMESESL | 0 | 0.000609088 | 0 |
| 505 | SSTSRTMESESL | 0 | 0.001917066 | 0 |
| 506 | ISSSTSRTMESESLR | 0 | 0.006167648 | 0.000314254 |
| 507 | ISSSTSRTMESESLRT | 0 | 0.002386175 | 0 |
| 508 | ISSSTSRTMESESLRTLE | 0 | 0.00100915 | 0 |
| 509 | SSSTSRTMESESLR | 0 | 0.003503983 | 0 |
| 510 | SSTSRTMESESLR | 0 | 0.005191985 | 8.70719E-05 |
| 511 | STSRTMESESLR | 0 | 5.68759E-05 | 5.29561E-06 |
| 512 | SSSTSRTMESESLRTLE | 0 | 0.002567117 | 0 |
| 513 | SSTSRTMESESLRT | 1.84696E-05 | 0.008140287 | 1.12875E-05 |
| 514 | SSTSRTMESESLRTLE | 0 | 0.004483884 | 0.00012075 |
| 515 | DNDISSSTSRTME | 0 | 0 | 0 |
| 516 | DNDISSSTSRTMESESLR | 0 | 0.001019031 | 0 |
| 517 | DNDISSSTSRTMESESLRT | 0 | 0.000912034 | 0 |
| 518 | NDNDISSSTSRTM | 0 | 0.000365459 | 4.22728E-05 |
| 519 | NDNDISSSTSRTME | 0 | 0.000318385 | 0.000178871 |
| 520 | NDNDISSSTSRTMES | 0 | 2.99974E-05 | 0.001076168 |
| 521 | NDNDISSSTSRTMESE | 0 | 0.000801882 | 7.86622E-05 |
| 522 | GPGPESSPVVVRTDEDVPSAPP | 0 | 0.001251405 | 0 |
| 523 | SPVVVRTDEDVPSAPP | 0 | 0.020219129 | 0.000323127 |
| 524 | SPVVVRTDEDVPSAPPR | 0 | 0.003613644 | 1.8187E-05 |
| 525 | ADRYVAIARALPAGP | 0 | 0 | 0.002655646 |
| 526 | ADRYVAIARALPAGPRPS | 0 | 0 | 0.002875424 |
| 527 | DRYVAIARALPAGP | 0 | 0 | 0.002288976 |
| 528 | RYVAIARALPAGP | 0 | 0.000417439 | 0.004018669 |
| 529 | YVAIARALPAGP | 0 | 0 | 0.005977673 |
| 530 | DRYVAIARALPAGPRP | 0 | 0 | 4.04056E-05 |
| 531 | RYVAIARALPAGPRP | 0 | 0 | 0.001523029 |
| 532 | YVAIARALPAGPR | 0 | 0 | 0.003420167 |
| 533 | YVAIARALPAGPRP | 0 | 0 | 0.008166764 |
| 534 | RYVAIARALPAGPRPS | 0 | 0 | 0.000519271 |
| 535 | YVAIARALPAGPRPS | 8.87893E-05 | 0.000424523 | 0.022206232 |
| 536 | YVAIARALPAGPRPSTPG | 0 | 0 | 0.003957309 |
| 537 | ISQRSAGGRPGSGP | 5.38491E-06 | 0 | 0.001643627 |
| 538 | AIEVAAGESAGRSPG | 0 | 0 | 0.000184372 |
| 539 | APGGRPAPSQDLN | 0 | 0 | 0.001844106 |
| 540 | HEGAKSASAEELR | 0 | 8.76974E-05 | 0 |
| 541 | HEGAKSASAEELRR | 0 | 0.000314441 | 0 |
| 542 | LHEGAKSASAEELRR | 0 | 9.42694E-05 | 0 |
| 543 | LHEGAKSASAEELR | 0 | 0.001104404 | 0 |
| 544 | SPEPPAKTSTPEDF | 0 | 0.00029582 | 0 |
| 545 | ELVTTKTAGPEQQ | 0 | 0.00018261 | 0 |
| 546 | SDHQVLSGKTTTTN | 0 | 0 | 0.000483505 |
| 547 | SDHQVLSGKTTTTNSKR | 0 | 0 | 0.000180067 |
| 548 | GNLIPARPAPAPP | 0 | 0 | 0.011117321 |
| 549 | SQGNLIPARPAPAPP | 0 | 0 | 0.005400016 |
| 550 | SRVDVYTTHSPAG | 0 | 0.002140225 | 0.000304427 |
| 551 | SRVDVYTTHSPAGTS | 0 | 0.000342023 | 0.000569232 |
| 552 | AAAAAAAGAFAGRR | 5.83429E-06 | 0 | 0.001602102 |
| 553 | AAAAAAAGAFAGRRA | 0 | 0 | 0.000205015 |
| 554 | AGAAGASAASYSSTQ | 0 | 0 | 0.001093631 |
| 555 | GPPAYHETLAGGAAAP | 0 | 2.9154E-05 | 0.001718791 |
| 556 | GPPAYHETLAGGAAAPYP | 0 | 0 | 0.004927555 |
| 557 | GPPAYHETLAGGAAAPYPA | 0 | 0 | 0.174134375 |
| 558 | GPPAYHETLAGGAAAPYPAS | 0.000106864 | 0 | 0.030924933 |
| 559 | GPPAYHETLAGGAAAPYPASQPP | 0.000558172 | 0 | 0.048345549 |
| 560 | PPAYHETLAGGAAAPYPA | 0 | 0.001842707 | 0.008104609 |
| 561 | YHETLAGGAAAPYPA | 0 | 6.08531E-05 | 0.03506226 |
| 562 | HETLAGGAAAPYPA | 0 | 0 | 0.006416883 |
| 563 | HETLAGGAAAPYPAS | 0 | 0 | 0.000379231 |
| 564 | PPAYHETLAGGAAAPYPAS | 0 | 0 | 0.003138268 |
| 565 | PPAYHETLAGGAAAPYPASQPP | 8.75032E-05 | 0 | 0.010747898 |
| 566 | YHETLAGGAAAPYPAS | 0 | 0.000108789 | 0.012094466 |
| 567 | YHETLAGGAAAPYPASQ | 0 | 0 | 0.000581941 |
| 568 | YHETLAGGAAAPYPASQPP | 4.28042E-05 | 0 | 0.013224186 |
| 569 | AGPTASPAYSSYQPTP | 0 | 0 | 0.012545936 |
| 570 | AGPTASPAYSSYQPTPT | 0 | 0 | 0.003166212 |
| 571 | GPTASPAYSSYQPTP | 0 | 0 | 0.00205569 |
| 572 | FPPPASSAAYSWP | 0 | 0.009869343 | 0 |
| 573 | VPNFPPPASSAAYSWP | 0 | 0 | 0 |
| 574 | KPSPASGFSPSVQ | 0 | 0.000144627 | 0.004136506 |
| 575 | TKPSPASGFSPSVQ | 0 | 0 | 0.001978141 |
| 576 | GPGAYPSSGQPSATGAYP | 0 | 0.000155579 | 0.001014847 |
| 577 | QPSATGAYPATGPYG | 0.000109489 | 0 | 0.002621853 |
| 578 | GPSAAFAPAAAEP | 0 | 0 | 0.000882006 |
| 579 | HRGPSAAFAPAAAEPK | 0 | 0 | 0.002355048 |
| 580 | SPPPYTAYAAPAPEQAYG | 0 | 0.000211656 | 0.006567128 |
| 581 | SPPPYTAYAAPAPEVGRT | 0 | 0 | 0.001927513 |
| 582 | SSLYRAGGAYGPGTP | 0 | 7.02179E-05 | 0.001324544 |
| 583 | SSLYRAGGAYGPGTPE | 0 | 0 | 0.001354453 |
| 584 | DWTSGPPASCPSDR | 0 | 0 | 0.000480952 |
| 585 | GTSDWTSGPPASCPSDR | 0 | 0 | 0.052289166 |
| 586 | GTSDWTSGPPASCPSDRK | 0 | 2.22108E-05 | 0.008349466 |
| 587 | SDWTSGPPASCPSDR | 0 | 6.80337E-05 | 0.003160244 |
| 588 | TSDWTSGPPASCPSDR | 0 | 7.18059E-05 | 0.096422353 |
| 589 | WTSGPPASCPSD | 0.000720248 | 0 | 0.053468154 |
| 590 | WTSGPPASCPSDR | 0 | 0.011900526 | 0.008898023 |
| 591 | SDWTSGPPASCPSD | 0 | 0 | 0.005829123 |
| 592 | TSDWTSGPPASCPSD | 0 | 0 | 0.171369773 |
| 593 | TSDWTSGPPASCPSDRK | 0 | 0.000641832 | 0.010234954 |
| 594 | GTSDWTSGPPASCP | 0 | 6.23107E-05 | 0.001758031 |
| 595 | GTSDWTSGPPASCPSD | 0 | 0 | 0.07045114 |
| 596 | TSDWTSGPPASCP | 0 | 0 | 0.002934158 |
| 597 | TSDWTSGPPASCPS | 0 | 0 | 0.013840633 |
| 598 | GPTGGGGGGGFNTVGR | 1.01035E-05 | 0.000102412 | 0.070196683 |
| 599 | YPRQDWTGSTPAYG | 0 | 0 | 0.00154889 |
| 600 | TPLATAFGTSSSSAT | 0 | 0 | 0.005615657 |
| 601 | EPRPAEPPAWAGGARPGP | 0 | 0 | 0.000195421 |
| 602 | EPRPAEPPAWAGGARPGPG | 0 | 0 | 0.002112406 |
| 603 | SALGAAYGTAKSGTG | 0 | 0.000144638 | 0.000711491 |
| 604 | SGAYGAAKAGGSFD | 0 | 0 | 0.000354456 |
| 605 | SPYGGGRPGRVK | 0 | 0 | 0.006768076 |
| 606 | SPKTPGGFSPGIPFQTEEGR | 0 | 0 | 0.000922327 |
| 607 | TPGGFSPGIPFQTEEGR | 0.000677133 | 0 | 0.003926693 |
| 608 | AAAAAAAAAAQQQ | 0 | 0 | 0.008146366 |
| 609 | AASTAASSAAQNAFK | 0 | 0 | 0.001255367 |
| 610 | ASTAASSAAQNAFK | 5.43473E-05 | 0 | 0.001254667 |
| 611 | AAANAAAGAAENAFR | 4.4021E-05 | 7.24954E-05 | 0.000879681 |
| 612 | AAANAAAGAAENAFRAP | 0 | 0 | 0.001157119 |
| 613 | AANAAAGAAENAFR | 0.001218352 | 1.6607E-05 | 0.004548357 |
| 614 | NAAAGAAENAFR | 8.90681E-05 | 0 | 0.006397495 |
| 615 | AANAAAGAAENAFRAP | 0 | 0 | 0.003084297 |
| 616 | ANAAAGAAENAFRA | 8.91524E-05 | 0 | 0 |
| 617 | NAAAGAAENAFRAP | 0 | 9.94635E-05 | 0.008198617 |
| 618 | EPGEAAAGGAAEEARR | 0 | 0 | 0.000519957 |
| 619 | QPEPGEAAAGGAAEEARR | 0 | 0 | 0.02001433 |
| 620 | QPEPGEAAAGGAAEEAR | 0 | 0 | 0.004885232 |
| 621 | APEQRGAGASAPAPAQ | 0 | 0 | 0.000519221 |
| 622 | DPAASSSAEDSLSDA | 0 | 0.002521822 | 4.80059E-05 |
| 623 | VDPAASSSAEDSLSDAA | 0 | 0.001972734 | 0 |
| 624 | APGEEAAAGEEGAAGGDPQEAKP | 0 | 0 | 0.001569946 |
| 625 | EEGAAGGDPQEAKP | 0 | 0 | 0.001848083 |
| 626 | GEEGAAGGDPQEAKP | 0 | 0 | 0.001055432 |
| 627 | EEGAAGGDPQEAKPQ | 0 | 0 | 0.001853562 |
| 628 | EEGAAGGDPQEAKPQE | 0 | 0 | 0.001732593 |
| 629 | EEGAAGGDPQEAKPQEA | 0 | 0 | 0.000668217 |
| 630 | GEEGAAGGDPQEAKPQEA | 0 | 0 | 0.000598064 |
| 631 | GEEGAAGGDPQEAKPQ | 0 | 0 | 0.000591447 |
| 632 | GEEGAAGGDPQEAKPQE | 0 | 0 | 0.001118798 |
| 633 | DEPTTATDPEEPS | 0 | 0.006365138 | 0 |
| 634 | DEPTTATDPEEPSVV | 0 | 0.002021226 | 0.000608939 |
| 635 | DEPTTATDPEEPSVVG | 0 | 0.002724527 | 0 |
| 636 | VNDEPTTATDPEEPSVVG | 0 | 0.006374438 | 6.28535E-05 |
| 637 | VNDEPTTATDPEEP | 0 | 0.006465962 | 0.000295897 |
| 638 | VNDEPTTATDPEEPS | 0 | 0.013385341 | 0.000490569 |
| 639 | VNDEPTTATDPEEPSVV | 0 | 0.008177327 | 0.001004768 |
| 640 | PPAENSSAPEAEQGGAE | 0.007715771 | 0.011792411 | 0.006192337 |
| 641 | EPGGGAAEEAAPP | 0.000113941 | 0 | 0.015840374 |
| 642 | EPGGGAAEEAAPPWA | 0 | 0 | 0.001142927 |
| 643 | VRVEPGGGAAEEAAPP | 0 | 0 | 0.000381613 |
| 644 | AAAMAAAAAETSQR | 0 | 0 | 0.000186764 |
| 645 | AAAMAAAAAETSQRIQ | 0 | 0 | 0.001626765 |
| 646 | AKAAAMAAAAAETSQR | 0 | 0 | 0.001038313 |
| 647 | KAAAMAAAAAETSQR | 0 | 2.596E-05 | 0.000939214 |
| 648 | GVPAAAPGQPAQ | 0 | 0 | 0.001110432 |
| 649 | LDGVPAAAPGQPAQ | 0 | 0.000111713 | 0.034737443 |
| 650 | APAAGPYPSMPSTAADPS | 0 | 0.000181827 | 0.001986065 |
| 651 | AAANAASTAASSAA | 0 | 7.55048E-05 | 0.001488384 |
| 652 | AAGAEGAGAPAAAASAEPK | 0 | 0 | 0.001820073 |
| 653 | AGAEGAGAPAAAASA | 0 | 0 | 0.003232777 |
| 654 | AGAEGAGAPAAAASAEP | 0 | 0.000142272 | 0.00539477 |
| 655 | AGAEGAGAPAAAASAEPK | 0 | 0 | 0.005055599 |
| 656 | APDGAAAAASGSS | 0 | 0 | 0.016061703 |
| 657 | TPASATASSEPAPASAA | 0 | 0.000166011 | 0.001989027 |
| 658 | AGGAAAAAAEAGAA | 0 | 0 | 0.001925366 |
| 659 | AGGAAAAAAEAGAAS | 0 | 0.000139236 | 0.003042137 |
| 660 | AGGAAAAAAEAGAASG | 0 | 2.14705E-05 | 0.000911574 |
| 661 | TPASATASSEPAPASAAK | 0 | 0 | 0.000688661 |
| 662 | HPAASEGAAAAAASPP | 0 | 5.68419E-05 | 0.00299135 |
| 663 | TAAANAASTAASSAA | 0 | 0 | 0.000576899 |
| 664 | GEPGGGAAGPAAAA | 0 | 0 | 0.004542518 |
| 665 | GGEPGGGAAGPAAAA | 0 | 0 | 0.000703762 |
| 666 | GPRGAGDPGGAGASSGPGAP | 0 | 3.46658E-05 | 0.00160031 |
| 667 | GPRGAGDPGGAGASSGPGAPA | 0 | 0 | 0.001097296 |
| 668 | GPRGAGDPGGAGASSGPG | 0 | 0 | 0.004193567 |
| 669 | EAPDGAAAAASGSS | 0 | 0 | 0.002307714 |
| 670 | GPVPQHVVEAPDGAAAAASGSS | 0 | 0 | 0.003149752 |
| 671 | APDGAAAAASGSSL | 0 | 0 | 0.003968556 |
| 672 | APDGAAAAASGSSLD | 0 | 4.19184E-05 | 0.013400378 |
| 673 | APDGAAAAASGSSLDS | 0 | 0 | 0.012923142 |
| 674 | APDGAAAAASGSSLDSF | 0 | 0 | 0.00841207 |
| 675 | AAEAAAAPAESAAPA | 0 | 0 | 0.005504963 |
| 676 | APKAAEAAAAPAESAAPA | 6.9625E-05 | 0 | 0.056736752 |
| 677 | APKAAEAAAAPAESAAPAAG | 0 | 0 | 0.007319585 |
| 678 | APGPAAGGEAPKAAE | 0 | 0 | 0.000371488 |
| 679 | APGPAAGGEAPKAAEA | 0 | 1.53825E-05 | 0.003458118 |
| 680 | APGPAAGGEAPKAAEAA | 0 | 0 | 0.004647194 |
| 681 | APGPAAGGEAPKAAEAAA | 0 | 0 | 0.000353374 |
| 682 | APGPAAGGEAPKAAEAAAAP | 0.000114925 | 0 | 0.006692122 |
| 683 | APGPAAGGEAPKAAEAAAAPAE | 0.000290894 | 0.00024764 | 0.034426229 |
| 684 | SPGAAEPARAP | 0 | 0 | 0.002795975 |
| 685 | SPGAAEPARAPD | 0.00011817 | 0 | 0.004184787 |
| 686 | SPGAAEPARAPDS | 0 | 0 | 0.004795542 |
| 687 | SPGAAEPARAPDSL | 0 | 0 | 0.00166428 |
| 688 | APKAAEAAAAPAE | 0 | 0 | 0.001137725 |
| 689 | APKAAEAAAAPAES | 0 | 0 | 0.000980939 |
| 690 | GPAAGGEAPKAAEAAAAPAE | 0 | 0 | 0.002356434 |
| 691 | EAPKAAEAAAAPAE | 0 | 2.84785E-05 | 0.000784197 |
| 692 | GEAPKAAEAAAAPAE | 0 | 0.00021984 | 0.013058376 |
| 693 | GGEAPKAAEAAAAPAE | 1.73506E-05 | 0 | 0.011165934 |
| 694 | GEAPKAAEAAAAP | 0 | 1.96266E-05 | 0.002123407 |
| 695 | GEAPKAAEAAAAPA | 0 | 0 | 0.001410088 |
| 696 | GGEAPKAAEAAAAPA | 0 | 0 | 0.000301874 |
| 697 | GGEAPKAAEAAAAP | 0 | 0 | 0.001452153 |
| 698 | GAAAAAAEAGAASG | 0 | 0 | 0.002103046 |
| 699 | GAAAAAAEAGAASGE | 0 | 0 | 0 |
| 700 | GAAAAAAEAGAASGEQ | 0 | 0 | 0.008524353 |
| 701 | GGAAAAAAEAGAASGEQ | 0 | 0 | 0.000638746 |
| 702 | TPSGPESGPTPASAEQN | 0 | 0 | 0.003811161 |
| 703 | TPSGPESGPTPASAEQNE | 1.63666E-05 | 0 | 0.00390743 |
| 704 | APGPATTQAGDAARAA | 0 | 0 | 0.000490431 |
| 705 | GPGAPAGAQPAQPP | 0 | 0 | 0.033508451 |
| 706 | GPGAPAGAQPAQPPS | 0.000272913 | 0.000223562 | 0.012655728 |
| 707 | GPGAPAGAQPAQPPSS | 0 | 0 | 0.005245028 |
| 708 | GPAAAQGSAAAPAEP | 0 | 5.28589E-05 | 0.002388032 |
| 709 | GPAAAQGSAAAPAEPK | 0 | 2.32078E-06 | 0.004168347 |
| 710 | GPAAAQGSAAAPAEPKII | 0 | 0.001065806 | 0 |
| 711 | GPPRPSRGPAAAQGSAAAPA | 0 | 0 | 0.005335766 |
| 712 | GPPRPSRGPAAAQGSAAAPAEP | 0 | 7.37067E-05 | 0.009785927 |
| 713 | GPPRPSRGPAAAQGSAAAPAEPK | 0 | 0 | 0.003834318 |
| 714 | TPSGQAGAAASESL | 0 | 0 | 0.009804258 |
| 715 | EKGEPAAAAAPEAG | 0 | 0 | 0.001073992 |
| 716 | EKGEPAAAAAPEAGASP | 0 | 0 | 0.001092811 |
| 717 | KGEPAAAAAPEAGASP | 0 | 0 | 0.001922528 |
| 718 | SPSAAEKGEPAAAAAP | 0 | 0 | 0.000958741 |
| 719 | SPSAAEKGEPAAAAAPE | 0 | 0 | 0.005239821 |
| 720 | SPSAAEKGEPAAAAAPEAG | 0.000181366 | 0 | 0.001774413 |
| 721 | ETPAATEAPSSTPK | 0 | 8.69221E-05 | 0.000136896 |
| 722 | APESFPAAASPGDSATG | 0 | 0 | 0.000406292 |
| 723 | APESFPAAASPGDSATGHRRPP | 0 | 0 | 0.007720529 |
| 724 | YPAGATGAQAAP | 0.000146737 | 4.20453E-05 | 0.004845469 |
| 725 | YPAGATGAQAAPQ | 0 | 0 | 0.003027271 |
| 726 | YPAGATGAQAAPQAQ | 0 | 0.000116416 | 0.014064897 |
| 727 | YPAGATGAQAAPQAQA | 0 | 0 | 0.001538819 |
| 728 | YPAGATGAQAAPQAQAGPT | 0 | 0 | 0.000330803 |
| 729 | RPAGPGAPEAAGTEASSEE | 0 | 3.43757E-05 | 0.001106133 |
| 730 | RPAGPGAPEAAGTEASSEEV | 6.98581E-05 | 0 | 0.000640303 |
| 731 | RPAGPGAPEAAGTEASSEEVG | 2.63622E-05 | 0 | 0.00156279 |
| 732 | ADDTAGAAAATGN | 0 | 0 | 0.000548665 |
| 733 | ADDTAGAAAATGNGDI | 0 | 0 | 0.002452508 |
| 734 | SPIPGAGAYADDTAGAAAATG | 0 | 0 | 0.002306522 |
| 735 | SPIPGAGAYADDTAGAAAATGN | 0 | 0 | 0.027775651 |
| 736 | SPIPGAGAYADDTAGAAAATGNG | 0 | 0 | 0.003863181 |
| 737 | YADDTAGAAAATGN | 0 | 0 | 0.009978746 |
| 738 | YADDTAGAAAATGNG | 0 | 0 | 0.00131784 |
| 739 | APSPVGAGTAAPAPSQ | 0 | 0 | 0.001842841 |
| 740 | APSPVGAGTAAPAPSQTPG | 0 | 0.000538604 | 0.001176373 |
| 741 | APVPAAAPASSSDP | 1.76909E-05 | 0.000179629 | 0.004085979 |
| 742 | APVPAAAPASSSDPA | 0 | 0 | 0.005863307 |
| 743 | TPASITPASATASSEPAP | 0 | 0 | 0.004006295 |
| 744 | TPASITPASATASSEPAPA | 0 | 0.00054799 | 0.004724743 |
| 745 | AVAPAAGQGSHSR | 0 | 0 | 0.002071351 |
| 746 | AVAPAAGQGSHSRQ | 1.55346E-06 | 0 | 0.001039753 |
| 747 | TPGGSGAAVAPAAGQGSHSRQ | 0 | 0 | 0.0044951 |
| 748 | TPGGSGAAVAPAAGQGSHSRQK | 0 | 0 | 0.001218304 |
| 749 | TPGGSGAAVAPAAGQ | 0 | 0 | 0.007156448 |
| 750 | TPGGSGAAVAPAAGQG | 6.37004E-05 | 0 | 0.026531364 |
| 751 | TPGGSGAAVAPAAGQGSH | 0 | 0.000293048 | 0.001596555 |
| 752 | TPGGSGAAVAPAAGQGSHS | 0 | 2.69868E-05 | 0.00351542 |
| 753 | TPGGSGAAVAPAAGQGSHSR | 1.29172E-05 | 0 | 0.010093112 |
| 754 | GPGAGGAGSAVPGGAGP | 0 | 0 | 0.002971742 |
| 755 | GPGGSGAGPGGGLTPSAPP | 0 | 0.00014372 | 0.123675971 |
| 756 | GPGGSGAGPGGGLTPSAPPY | 0 | 0.000205612 | 0.094069507 |
| 757 | GPGGSGAGPGGGLTPSAPPYG | 0 | 9.09356E-05 | 0.02764423 |
| 758 | TPGGSGAAVAPAA | 0 | 0 | 0 |
| 759 | TPGGSGAAVAPAAG | 4.2647E-06 | 0 | 0.005617817 |
| 760 | VPAPTPAPAAAPVPAAAPA | 0 | 0 | 0.003489015 |
| 761 | GPSGAPPMPGGPH | 0 | 0 | 0.003824442 |
| 762 | SGPSGAPPMPGGPH | 0 | 0.000188131 | 0.001397008 |
| 763 | VPSGPSGAPPMPGGPH | 0 | 0 | 0.114223457 |
| 764 | VPSGPSGAPPMPGGPHS | 0 | 0 | 0.009471807 |
| 765 | GPPPAPPGLPAGADCLNSFT | 0.000217885 | 0.000217927 | 0.004555906 |
| 766 | SPTAGTTEPGHQ | 0 | 0 | 0.000950562 |
| 767 | SPTAGTTEPGHQQ | 0 | 0 | 0.003133052 |
| 768 | TPSENTSPTAGTTEPGHQQ | 0 | 0 | 0.001458472 |
| 769 | TSPTAGTTEPGHQQ | 0.000117108 | 0 | 0.00223486 |
| 770 | TSPTAGTTEPGHQQF | 0 | 0 | 0.002139528 |
| 771 | APAEEPAAAAASSA | 0 | 0 | 0.005106814 |
| 772 | EPAAAAASSACAAPSQ | 0 | 0 | 0.000972447 |
| 773 | PAEEPAAAAASSACAAPSQ | 0 | 0 | 0.019921583 |
| 774 | KPSEEEAAVAAGGPPG | 0 | 0 | 0.00664349 |
| 775 | KPSEEEAAVAAGGPPGGPQ | 0 | 0 | 0.006481161 |
| 776 | APPAPSAPNSAPSNLGP | 0.000773066 | 3.75089E-05 | 0.005185782 |
| 777 | APPAPSAPNSAPSNLGPQ | 0 | 0 | 0.005234594 |
| 778 | APPAPSAPNSAPSNLGPQT | 0 | 0 | 0.001436988 |
| 779 | GPGGAAAEVGP | 0 | 0 | 0.008950041 |
| 780 | GPGGAAAEVGPLPK | 0 | 0 | 0.002279013 |
| 781 | GIGSPSTDASASDVH | 0 | 5.73319E-05 | 0 |
| 782 | GIGSPSTDASASDVHG | 0 | 0.001533634 | 0 |
| 783 | GIGSPSTDASASDVHGN | 2.20345E-05 | 0.001028865 | 0 |
| 784 | IGSPSTDASASDVH | 0 | 5.33131E-05 | 0 |
| 785 | IGSPSTDASASDVHG | 0 | 0.000972211 | 0 |
| 786 | AGPGGGSPEPYH | 0 | 0 | 0.001753894 |
| 787 | EPAGPGGGSPEPYH | 7.68763E-06 | 0 | 0.05337185 |
| 788 | EPAGPGGGSPEPYHP | 0 | 0 | 0.021886947 |
| 789 | EPAGPGGGSPEPYHPT | 0 | 0 | 0.011945218 |
| 790 | GPGGGSPEPYH | 0 | 0 | 0.002208952 |
| 791 | VEPAGPGGGSPEPYH | 0 | 0 | 0.001243876 |
| 792 | APAPASATEILL | 0.000441273 | 0.034460524 | 4.8431E-05 |
| 793 | APAPASATEILLTPA | 0 | 0.006146933 | 0 |
| 794 | SAPAPASATEILLTPA | 0 | 0.001105908 | 0.001347268 |
| 795 | SAPAPASATEILL | 5.59336E-05 | 0.020630222 | 0 |
| 796 | QASAAAATAELLKKQ | 0.00011677 | 0 | 0.001444402 |
| 797 | QASAAAATAELLKKQE | 0 | 0 | 0.001187174 |
| 798 | SYSTQASAAAATAE | 0 | 5.37025E-05 | 0.002372376 |
| 799 | SYSTQASAAAATAEL | 0 | 0 | 0.022960789 |
| 800 | SYSTQASAAAATAELL | 0 | 0 | 0.003077826 |
| 801 | GGGGSGGIAEAGSGHM | 0 | 0 | 0.000577158 |
| 802 | SPAPTTVPEAPG | 2.34256E-05 | 0.005818315 | 0 |
| 803 | SPAPTTVPEAPGP | 0.002404448 | 0.074529683 | 0 |
| 804 | TSPAPTTVPEAPGP | 0 | 0.00096297 | 0.001832642 |
| 805 | SPAPTTVPEAPGPL | 0 | 0.001330418 | 4.93037E-05 |
| 806 | SPAPTTVPEAPGPLP | 0 | 0.006672989 | 0 |
| 807 | SPAPTTVPEAPGPLPS | 5.84679E-05 | 0.008509986 | 0 |
| 808 | GPGVPQASGAPATPSA | 0.000671256 | 0.000313807 | 0.008946056 |
| 809 | GPGVPQASGAPATPSALR | 0 | 0 | 0.011697429 |
| 810 | SPDPSSASAAPIG | 0 | 0 | 0.002803353 |
| 811 | VPEPEPGPGGAGAAPLG | 0 | 0 | 0.002762613 |
| 812 | VPEPEPGPGGAGAAPLGT | 0 | 0 | 0.001212786 |
| 813 | EPRPTAPSSGAPGLAG | 0 | 0 | 0.013635972 |
| 814 | EPRPTAPSSGAPGLAGVG | 0 | 0 | 0.003506332 |
| 815 | APPHQEGEPGAGVTPAS | 0 | 0 | 0 |
| 816 | APPHQEGEPGAGVTPASTP | 0 | 0 | 0.003208333 |
| 817 | APPHQEGEPGAGVTPASTPP | 4.5802E-05 | 0 | 0.13163739 |
| 818 | APPHQEGEPGAGVTPASTPPS | 0 | 0 | 0.046783901 |
| 819 | PPHQEGEPGAGVTPASTPP | 0 | 0 | 0.001553499 |
| 820 | AAKSIAAATSALVK | 0 | 0 | 0.001260629 |
| 821 | HMAGAAAAGAVVG | 0 | 0 | 0.001734349 |
| 822 | KVAPAPAVVK | 6.11282E-05 | 0.000414435 | 0 |
| 823 | DVSAGAAGGVVSPR | 0 | 0 | 0.006099556 |
| 824 | ISYDVSAGAAGGVVSPR | 0 | 0 | 0.00945308 |
| 825 | APVPTAPAAGAPLMDFGN | 0 | 0 | 0.000389286 |
| 826 | DEGSPSASTPMINK | 0 | 0 | 0.002907084 |
| 827 | DEGSPSASTPMINKTG | 0 | 0 | 0.001558986 |
| 828 | SPSASTPMINK | 0 | 0 | 0.002324596 |
| 829 | APTGSSPQPLQ | 0 | 0 | 0.001386756 |
| 830 | GAPTGSSPQPLQ | 0 | 0 | 0.0096955 |
| 831 | GAPTGSSPQPLQPL | 0 | 0 | 0.002107705 |
| 832 | IRYMSGGTATG | 0 | 0 | 0.001254933 |
| 833 | IRYMSGGTATGDA | 0 | 0 | 0.004708046 |
| 834 | IRYMSGGTATGDAI | 0 | 0 | 0.003435189 |
| 835 | IRYMSGGTATGDAIS | 3.32791E-05 | 0.000570585 | 0.007989502 |
| 836 | NIRYMSGGTATGDAIS | 0 | 0 | 0.011485929 |
| 837 | NIRYMSGGTATG | 0 | 0 | 0.00543069 |
| 838 | NIRYMSGGTATGDA | 0.001403723 | 0.00085565 | 0.005788765 |
| 839 | NIRYMSGGTATGDAI | 0 | 0 | 0.00272834 |
| 840 | DKRDFVSAGAAAGVAA | 0 | 0 | 0.000694917 |
| 841 | FIGASAASML | 4.71895E-05 | 0 | 0.006639215 |
| 842 | FIGASAASMLI | 0 | 0 | 0.001670388 |
| 843 | GFIGASAASML | 0 | 0 | 0 |
| 844 | TPALAYLASAPPPLCPSG | 0 | 0 | 0.002722337 |
| 845 | VFIGTGATGATLY | 0 | 0.007169347 | 0.006628865 |
| 846 | VPIPWVSGTSASTP | 0 | 0 | 0.024123014 |
| 847 | VPIPWVSGTSASTPV | 0.001853509 | 0 | 0.006115648 |
| 848 | VPIPWVSGTSASTPVF | 0 | 2.59086E-05 | 0.015116311 |
| 849 | VPIPWVSGTSASTPVFG | 0 | 0.00028408 | 0.078223007 |
| 850 | VPIPWVSGTSASTPVFGG | 0 | 0 | 0.001677651 |
| 851 | GSRYIAGGGGTGSIG | 0 | 0 | 0.002733976 |
| 852 | SRYIAGGGGTGSIG | 0 | 0 | 0.002913257 |
| 853 | TAAKFIGAGAATVG | 0 | 0 | 0.002932327 |
| 854 | LPLIAGSAAAGVV | 0 | 0 | 0.000262029 |
| 855 | LPLIAGSAAAGVVF | 0 | 0 | 0.000448266 |
| 856 | AGAAAAGAVVGGLG | 0 | 0 | 0.007831945 |
| 857 | LVTGATVLGTIT | 0 | 0 | 0.007822141 |
| 858 | VTGATVLGTIT | 0 | 0 | 0.000878559 |
| 859 | DPELVDPVVAAGAVVT | 0 | 0 | 0.000709135 |
| 860 | VDPVVAAGAVVT | 0 | 0 | 0.002806962 |
| 861 | QPILTAGTVLPIF | 0 | 0 | 0.000307717 |
| 862 | GSPTAYGVIAAAA | 0 | 0.000116812 | 0.004684348 |
| 863 | GSPTAYGVIAAAAV | 0 | 0 | 0.003297931 |
| 864 | GSPTAYGVIAAAAVL | 0 | 0 | 0.001593101 |
| 865 | SPTAYGVIAAAA | 3.13516E-05 | 0.000275376 | 0.003088409 |
| 866 | SPTAYGVIAAAAV | 0 | 0 | 0.003717869 |
| 867 | SPTAYGVIAAAAVL | 0 | 0 | 0.001764398 |
| 868 | SPTAYGVIAAAAVLSA | 0 | 0 | 0.000413703 |
| 869 | GVIAAAAVLSAS | 0 | 0 | 0.000415647 |
| 870 | SPTAYGVIAAAAVLSAS | 0 | 0.001069041 | 0.001220173 |
| 871 | ARLPISSGSTSSSR | 0 | 1.30471E-05 | 0.000320492 |
| 872 | ARLPISSGSTSSSRI | 0 | 0 | 0.005231836 |
| 873 | LPISSGSTSSSR | 0 | 0 | 0.002155689 |
| 874 | LPISSGSTSSSRI | 4.83244E-05 | 0 | 0.002438111 |
| 875 | VDYKAFIPGGPSPGSR | 0.000205342 | 0.000187004 | 0.007486709 |
| 876 | VDYKAFIPGGPSPGSRV | 0 | 0 | 0.000966683 |
| 877 | DPDEGVAGAPTGSSP | 0 | 0 | 0.001799633 |
| 878 | IVDPAISSSGPAGSYRPYD | 0 | 0 | 0.000273603 |
| 879 | VDPAISSSGPAGSYR | 0 | 0 | 0.011059673 |
| 880 | VDPAISSSGPAGSYRP | 0.000233824 | 0 | 0.002613035 |
| 881 | VDPAISSSGPAGSYRPY | 0 | 0 | 0.021710745 |
| 882 | VDPAISSSGPAGSYRPYD | 0 | 0 | 0.000277644 |
| 883 | TPGPASGSTFRGR | 0.00013012 | 0 | 0.007525291 |
| 884 | TPGPASGSTFRGRRD | 0 | 0 | 0.010681357 |
| 885 | TPGPASGSTFRGRRDVS | 0 | 1.03745E-05 | 0.000394546 |
| 886 | VPTTPGPASGSTFRGR | 0 | 0 | 0.004509455 |
| 887 | VPTTPGPASGSTFRGRR | 0 | 0 | 0.001165244 |
| 888 | VPTTPGPASGSTFRGRRD | 0 | 0 | 0.007373082 |
| 889 | GPPYDRAHVTGASSSSSSST | 0.000156264 | 0 | 0.002744957 |
| 890 | GPPYDRAHVTGASSSSSSSTK | 0 | 0 | 0.000293204 |
| 891 | AVNSYYPTPPAPMPGP | 0.006432589 | 0.000456763 | 0 |
| 892 | AVNSYYPTPPAPMPGPT | 0.009036946 | 0.001602645 | 0 |
| 893 | ETPSAAALAAAR | 0 | 0 | 0.008466465 |
| 894 | VGETPSAAALAAAR | 0.000195276 | 0 | 0.005898902 |
| 895 | VGETPSAAALAAARVE | 0 | 0 | 0.007371498 |
| 896 | TPSAAALAAARVE | 0 | 0 | 0.000932867 |
| 897 | TPSAAALAAARVELPGTA | 0 | 0 | 0.001346627 |
| 898 | GPGPGGGVSGGKAE | 1.97095E-05 | 0 | 0.000258306 |
| 899 | GPGPGGGVSGGKAEAEAA | 0 | 0 | 0.001741828 |
| 900 | SAKQTPAPAAS | 0 | 0 | 0.001142083 |
| 901 | SPGSRETSAATLSPGAS | 0 | 4.96559E-05 | 0.002913359 |
| 902 | SPGSRETSAATLSPGASS | 0 | 0 | 0.001840779 |
| 903 | EPASPGVSPACASP | 0 | 0 | 0.006191212 |
| 904 | EPASPGVSPACASPE | 0 | 0 | 0.003649944 |
| 905 | EPASPGVSPACASPEN | 0 | 0 | 0.015292112 |
| 906 | DPDRPSAPTIGGSSCARMDAE | 0 | 0 | 0.001531664 |
| 907 | EPETWEQILRRNVLQHGAAAAPVS | 0 | 0 | 0.001960286 |
| 908 | NVLQHGAAAAPVS | 0 | 0 | 0.021163905 |
| 909 | RNVLQHGAAAAPVS | 0 | 0 | 0.004524787 |
| 910 | NVLQHGAAAAPVSAPT | 0 | 0 | 0.007727546 |
| 911 | RNVLQHGAAAAPVSAPT | 0 | 0 | 0.001360355 |
| 912 | VLQHGAAAAPVSAPT | 0 | 0 | 0.006717008 |
| 913 | DQTKAQAAAPASVPA | 0 | 1.2585E-05 | 0.002292164 |
| 914 | DQTKAQAAAPASVPAQ | 0 | 0 | 0.002007878 |
| 915 | DQTKAQAAAPASVPAQAPK | 0 | 0 | 0.000240427 |
| 916 | QTKAQAAAPASVPA | 0 | 0 | 0.000261136 |
| 917 | VIEEVEDSKPDTTAPP | 0 | 2.28608E-05 | 0.005575981 |
| 918 | VIEEVEDSKPDTTAPPS | 0 | 0 | 0.002691274 |
| 919 | DMPLWSSSTPSVGR | 0 | 0 | 0.002157804 |
| 920 | DMPLWSSSTPSVGRVS | 0 | 0 | 0.009024038 |
| 921 | MPLWSSSTPSVGR | 0 | 0.000299468 | 0.007222381 |
| 922 | WSSSTPSVGR | 0 | 0 | 0.003759072 |
| 923 | WSSSTPSVGRVS | 4.82147E-05 | 0 | 0.005638232 |
| 924 | YRPGYSSSSTSAAMPH | 0 | 0 | 0.000519706 |
| 925 | TPSSGAWGSAASLGRGP | 0 | 0 | 0.005087806 |
| 926 | TPSSGAWGSAASLGRGPQ | 0 | 7.19187E-05 | 0.00563308 |
| 927 | EPASPAASISRLSGEQ | 0 | 0 | 0.005525499 |
| 928 | DPEPGPGGGMAHG | 0 | 3.89862E-06 | 0.00190346 |
| 929 | DPEPGPGGGMAHGTT | 0 | 0 | 0.000579367 |
| 930 | DPEPGPGGGMAHGTTR | 0 | 5.12542E-05 | 0.001091011 |
| 931 | DPEPGPGGGMAHGTTRH | 0 | 1.05337E-05 | 0.001670504 |
| 932 | DPSGAGVPRT | 0 | 8.07821E-05 | 0.003471052 |
| 933 | DPSGAGVPRTL | 0 | 0 | 0.00933739 |
| 934 | DPSGAGVPRTLE | 0 | 0 | 0.000186544 |
| 935 | DPSGAGVPRTLENP | 0 | 2.94163E-05 | 0.01409835 |
| 936 | EDPSGAGVPRTLENP | 0 | 0 | 0.024426817 |
| 937 | EDPSGAGVPRTLENPV | 0 | 0 | 0.008296841 |
| 938 | EDPSGAGVPRT | 1.13933E-05 | 0 | 0.007749991 |
| 939 | EDPSGAGVPRTL | 0 | 0 | 0.018912089 |
| 940 | EDPSGAGVPRTLE | 0 | 0 | 0.002906666 |
| 941 | EDPSGAGVPRTLEN | 0 | 0 | 0.002697566 |
| 942 | SPSAGVPRASPEPP | 0 | 0 | 0.000381045 |
| 943 | SPSAGVPRASPEPPG | 0 | 0 | 0.004206025 |
| 944 | ISLAVATSSPTLARTST | 0 | 0 | 0.000600053 |
| 945 | IIGTSSVARAW | 0 | 0 | 0.000752885 |
| 946 | IIGTSSVARAWS | 0.000296429 | 0 | 0.007306503 |
| 947 | IIGTSSVARAWSA | 0.000739883 | 0 | 0.001778744 |
| 948 | IIGTSSVARAWSAT | 0 | 0 | 0.007018658 |
| 949 | YIIGTSSVARAWSAT | 0 | 0.000113312 | 0.006666452 |
| 950 | YIIGTSSVARAWS | 0 | 0 | 0.007823528 |
| 951 | YIIGTSSVARAWSA | 0 | 0 | 0.002262648 |
| 952 | SYIIGTSSVARA | 0 | 0 | 0.000800613 |
| 953 | SYIIGTSSVARAWS | 0 | 0 | 0.001583463 |
| 954 | YIIGTSSVARA | 0 | 0 | 0.002411094 |
| 955 | LVVLGSGGVGKSA | 0 | 0 | 0.014298167 |
| 956 | LVVVGAGGVGKSA | 0 | 0 | 0.011472282 |
| 957 | LVVVGAGGVGKSALT | 0 | 0 | 0.005448014 |
| 958 | VIMVGSGGVGKSA | 0 | 0 | 0.050284159 |
| 959 | VIMVGSGGVGKSAL | 0.000150456 | 6.19769E-05 | 0.00132557 |
| 960 | VIMVGSGGVGKSALT | 0.000805043 | 9.17942E-05 | 0.01917618 |
| 961 | ITGSAASISLAQ | 0 | 0 | 0.001886216 |
| 962 | IVASTASSVAL | 0 | 2.51882E-05 | 0.002309782 |
| 963 | VPTLSIVASTASSVA | 0.000352474 | 0 | 0.011126773 |
| 964 | VVLVTGAGAGLGRAY | 0.000397586 | 0 | 0.001758176 |
| 965 | SVHVDSGASAITRES | 0 | 0 | 0.000778102 |
| 966 | PPPPATPGAGGLAVRTFVSH | 0 | 0.000258331 | 0.005175378 |
| 967 | WPPPPATPGAGGLAVRTFVSH | 0 | 0 | 0.006514184 |
| 968 | WPPPPATPGAGGLAVRTFV | 0 | 0 | 0.002313013 |
| 969 | WPPPPATPGAGGLAVRTFVS | 0 | 0 | 0.001606471 |
| 970 | GPGATAGGAEKSNVK | 0 | 0 | 0.007027519 |
| 971 | GPGATAGGAEKSNVKI | 0 | 0 | 0.001306709 |
| 972 | MGPGATAGGAEKSN | 2.60931E-06 | 0 | 0.000457783 |
| 973 | MGTSTGATANHGTSA | 0 | 7.29045E-06 | 0.000277997 |
| 974 | QPGPSGAPAQRATR | 0 | 0 | 0.001124312 |
| 975 | QPGPSGAPAQRATRG | 0 | 0 | 0.007978922 |
| 976 | SQPGPSGAPAQRATRG | 0 | 0 | 0.002495485 |
| 977 | SPAPGSPAQVKGQE | 0 | 0 | 0.00120571 |
| 978 | APPSALFAAAAAAAAA | 0 | 0 | 0 |
| 979 | APPSALFAAAAAAAAAAA | 0 | 0 | 0.005179821 |
| 980 | GPAGGSPPGIDPT | 0 | 0 | 0.000675974 |
| 981 | NPAPQIVQAASSAPALE | 0 | 0 | 0.004226482 |
| 982 | NPAPQIVQAASSAPALET | 8.84262E-05 | 4.76534E-05 | 0.005701037 |
| 983 | NPAPQIVQAASSAPALETD | 0 | 0 | 0.001185442 |
| 984 | PAPQIVQAASSAPALE | 0 | 0 | 0.004600161 |
| 985 | PAPQIVQAASSAPALET | 0 | 0 | 0.003587823 |
| 986 | RPPPPPPASVSPQAEP | 0 | 0.000311954 | 0 |
| 987 | SPKAGKPSAAAASVSTQHG | 0 | 0 | 0.002074462 |
| 988 | AHASTSAMADRIN | 0 | 0.003511844 | 1.23506E-05 |
| 989 | AHASTSAMADRINIK | 4.94272E-06 | 0.015462195 | 0.000257702 |
| 990 | AHASTSAMADRINIKR | 7.49321E-05 | 0.011105595 | 0 |
| 991 | ASTSAMADRIN | 0 | 3.58415E-05 | 0 |
| 992 | WAHASTSAMADRINIKR | 0 | 0.016704375 | 0 |
| 993 | HASTSAMADRINIK | 0 | 0.000348773 | 0 |
| 994 | HASTSAMADRINIKR | 0 | 0.001286045 | 0.000113078 |
| 995 | WAHASTSAMAD | 0 | 0 | 0.000335748 |
| 996 | WAHASTSAMADR | 5.8423E-06 | 0.002114519 | 0.001467349 |
| 997 | WAHASTSAMADRIN | 2.4215E-06 | 0.008225358 | 0.002046629 |
| 998 | WAHASTSAMADRINI | 0 | 0.000436271 | 0 |
| 999 | WAHASTSAMADRINIK | 1.92998E-05 | 0.018162272 | 0.000121814 |
| 1000 | GSCWAHASTSAMA | 0 | 0 | 0.001603188 |
| 1001 | GSCWAHASTSAMAD | 0 | 0 | 0.005523272 |
| 1002 | GSCWAHASTSAMADR | 0 | 0.000350068 | 0.024533195 |
| 1003 | GSCWAHASTSAMADRIN | 0 | 9.48515E-05 | 0.01242245 |
| 1004 | ELSFGRGSTAPVG | 0 | 0.000223027 | 0.010235193 |
| 1005 | ELSFGRGSTAPVGGG | 0 | 0 | 0.003415126 |
| 1006 | LRELSFGRGSTAPVG | 0 | 0 | 0.00129848 |
| 1007 | RELSFGRGSTAPVG | 0 | 4.60115E-06 | 0.0050541 |
| 1008 | RPSGVPDRFSGSKSGTSASL | 0 | 0 | 0.002157216 |
| 1009 | VPDRFSGSKSGTSAS | 0 | 0 | 0.001201037 |
| 1010 | VPDRFSGSKSGTSASL | 6.39803E-06 | 0 | 0.005213857 |
| 1011 | EVWTQRLHGGSAPLPQDRG | 0 | 0 | 0.004455871 |
| 1012 | QRLHGGSAPLPQD | 0 | 0 | 0.001422224 |
| 1013 | TQRLHGGSAPLPQD | 0 | 0 | 0.010190546 |
| 1014 | VWTQRLHGGSAPLPQD | 3.00651E-05 | 0 | 0.004511254 |
| 1015 | WTQRLHGGSAPLPQD | 7.25684E-05 | 8.53393E-05 | 0.017096225 |
| 1016 | TQRLHGGSAPLPQ | 0 | 0 | 0.004129638 |
| 1017 | WTQRLHGGSAPLPQ | 0 | 0 | 0.003399428 |
| 1018 | WTQRLHGGSAPLPQDRG | 0 | 0 | 0.001200388 |
| 1019 | KVKAPPSVPR | 0 | 7.04026E-05 | 0 |
| 1020 | APSPPPPYPVTPGYPEPALHPGP | 0 | 0 | 0.022508463 |
| 1021 | APSPPPPYPVTPGYPEPALHPGPG | 0 | 0 | 0.05527647 |
| 1022 | PPPPYPVTPGYPEPALHPGP | 0 | 0 | 0.003949042 |
| 1023 | PSPPPPYPVTPGYPEPALHPGP | 0 | 0 | 0.012880041 |
| 1024 | SPPPPYPVTPGYPEPALHPGP | 0 | 0.000125471 | 0.024839333 |
| 1025 | PSPPPPYPVTPGYPEPALHPGPG | 0 | 0 | 0.01878172 |
| 1026 | SPPPPYPVTPGYPEPALHPG | 0 | 0 | 0.003601203 |
| 1027 | SPPPPYPVTPGYPEPALHPGPG | 0 | 0 | 0.019975976 |
| 1028 | PPPYPVTPGYPEPALHPGPGQ | 0 | 0 | 0 |
| 1029 | GPTFGIKGGAAGGGYS | 0 | 0 | 0.009009458 |
| 1030 | GPTPPVRGAASSPAAVS | 0 | 0 | 0.001515687 |
| 1031 | QPLRSAAGAAA | 0 | 0 | 0 |
| 1032 | QPLRSAAGAAALA | 0.00036183 | 0 | 0.028812899 |
| 1033 | QPLRSAAGAAALAL | 0 | 0 | 0.007171079 |
| 1034 | QPLRSAAGAAALALT | 0.000177882 | 0 | 0.029812537 |
| 1035 | QPLRSAAGAAALALTV | 0 | 0 | 0.021793616 |
| 1036 | QPLRSAAGAAALALTVD | 0 | 0 | 0.044244609 |
| 1037 | SAAGAAALALTVD | 0 | 0 | 0.001537143 |
| 1038 | IPLYPVRSAAAAAAN | 0 | 0 | 0.006355327 |
| 1039 | APSSSLPTSLTIHHP | 0 | 0 | 0.000925651 |
| 1040 | SPSSLSASVGDRVT | 0 | 0 | 0.004386966 |
| 1041 | SPSTLSASVGDRVT | 0 | 0 | 0.000257056 |
| 1042 | DPQGVTCGAATLSAER | 0 | 0 | 0.001554231 |
| 1043 | DPQGVTCGAATLSAERV | 0 | 0 | 0.001401898 |
| 1044 | EKPSSIPTDKL | 0 | 5.13323E-05 | 0 |
| 1045 | KPSSIPTDKL | 0 | 0.005496641 | 0 |
| 1046 | KPSSIPTDKLL | 0 | 0.000447048 | 0 |
| 1047 | KPSSIPTDKLLVIT | 0 | 8.8632E-05 | 0 |
| 1048 | AAAIAYGLDKKVGAE | 0 | 0 | 0.000280264 |
| 1049 | EPTAAAIAYGLDKKVGAE | 0 | 0 | 0.010317509 |
| 1050 | EPTAAAIAYGLDKK | 0 | 0 | 0.19854006 |
| 1051 | EPTAAAIAYGLDKKV | 0 | 0 | 0.009897882 |
| 1052 | EPTAAAIAYGLDKKVG | 0 | 0.002735699 | 0.011330251 |
| 1053 | EPTAAAIAYGLDKR | 0 | 0 | 0.022546227 |
| 1054 | EPTAAAIAYGLDR | 0 | 6.37454E-05 | 0.013768264 |
| 1055 | EPTAAALAYG | 0 | 0 | 0.001066901 |
| 1056 | EPTAAALAYGL | 0 | 0 | 0.002184729 |
| 1057 | EPTAAALAYGLD | 0 | 0 | 0.019513991 |
| 1058 | EPTAAALAYGLDK | 0.00013868 | 0 | 0.099593731 |
| 1059 | IINEPTAAAIAYGLDKKVG | 0 | 0 | 0.012727378 |
| 1060 | INEPTAAAIAYGLDKKVG | 0 | 0 | 0.006719825 |
| 1061 | INEPTAAAIAYGLDKREGEKN | 0 | 0 | 0.000827998 |
| 1062 | NEPTAAAIAYGLDKK | 0 | 5.40549E-05 | 0.038745346 |
| 1063 | NEPTAAAIAYGLDKR | 0 | 0 | 0.00287696 |
| 1064 | NEPTAAAIAYGLDR | 0 | 0 | 0.000400772 |
| 1065 | NEPTAAALAYGLDK | 9.22148E-05 | 0.000151675 | 0.019221622 |
| 1066 | IINEPTAAAIA | 6.74746E-05 | 0 | 0.004444182 |
| 1067 | IINEPTAAAIAYG | 0 | 0 | 0.012441125 |
| 1068 | IINEPTAAAIAYGL | 0 | 0 | 0.005199405 |
| 1069 | IINEPTAAAIAYGLD | 0.000395401 | 0.000104045 | 0.045227122 |
| 1070 | IINEPTAAAIAYGLDK | 0 | 0 | 0.08102128 |
| 1071 | IINEPTAAAIAYGLDKK | 0 | 0 | 0.373503191 |
| 1072 | IINEPTAAAIAYGLDKKV | 0 | 0 | 0.034616724 |
| 1073 | IINEPTAAAIAYGLDKR | 0.000268288 | 0.000196906 | 0.037235118 |
| 1074 | IINEPTAAAIAYGLDR | 0.001121273 | 0 | 0.008044666 |
| 1075 | EPTAAAIAYGLDKRE | 0 | 0.000138905 | 0.004738556 |
| 1076 | IINEPTAAAIAYGLDKRE | 0 | 0.000156412 | 0.015100073 |
| 1077 | IINEPTAAAIAYGLDKREG | 0 | 0 | 0.00226251 |
| 1078 | IINEPTAAAIAYGLDKREGEKN | 0 | 0 | 0.00249351 |
| 1079 | INEPTAAAIAYGLDKRE | 0 | 0 | 0.011705582 |
| 1080 | PTAAAIAYGLDKK | 0 | 0 | 0.003065365 |
| 1081 | EPTAAAIAYGLDRT | 0 | 0.002941724 | 0.00766457 |
| 1082 | IINEPTAAAIAYGLDRT | 0.000299206 | 0 | 0.012045797 |
| 1083 | IINEPTAAAIAYGLDRTG | 0 | 0 | 0.001241875 |
| 1084 | INEPTAAAIAYGLDRT | 0 | 0 | 0.020636076 |
| 1085 | INEPTAAAIAYGLDKK | 0.002692682 | 0 | 0.601576758 |
| 1086 | INEPTAAAIAYGLDKKV | 0 | 2.20888E-05 | 0.022582475 |
| 1087 | INEPTAAAIAYGLDKR | 0 | 0.000198267 | 0.074174951 |
| 1088 | INEPTAAAIAYGLDR | 0 | 0 | 0.03696633 |
| 1089 | INEPTAAALAYG | 6.96933E-05 | 0 | 0.039307968 |
| 1090 | INEPTAAALAYGL | 0 | 0.000227549 | 0.006623793 |
| 1091 | INEPTAAALAYGLD | 0 | 0 | 0.057491062 |
| 1092 | INEPTAAALAYGLDK | 0.000848495 | 4.09239E-05 | 0.214627632 |
| 1093 | INEPTAAAIAYGLDRTG | 0 | 0.00069759 | 0.003102156 |
| 1094 | INEPTAAAMAYGLHK | 4.39466E-05 | 0 | 0.009766638 |
| 1095 | VINEPTAAAMAYGLHK | 0 | 2.99438E-05 | 0.004762592 |
| 1096 | APPGTPAIAWGEVPRHQL | 0 | 5.42852E-05 | 0.001249086 |
| 1097 | ELVGSVGGAVTFPLK | 0 | 0 | 0.001893535 |
| 1098 | ELVGSVGGAVTFPLKSKVK | 0 | 0 | 0.000467733 |
| 1099 | LVGSVGGAVTFPLK | 0 | 0 | 0.01020461 |
| 1100 | SVGGAVTFPLK | 0 | 0 | 0.024679759 |
| 1101 | VGSVGGAVTFPLK | 0 | 0 | 0.070297657 |
| 1102 | GPVKELVGSVGGAVTFPLK | 0 | 0 | 0.000477483 |
| 1103 | GPVKELVGSVGGAVTFPLKSK | 0 | 0 | 0.001349346 |
| 1104 | LVGSVGGAVTFPLKSK | 0 | 0 | 0.019683116 |
| 1105 | LVGSVGGAVTFPLKSKV | 0 | 0 | 0.004231708 |
| 1106 | SVGGAVTFPLKSK | 0.000933902 | 0.000243971 | 0.013556528 |
| 1107 | VGSVGGAVTFPLKSK | 2.29098E-05 | 0 | 0.093356426 |
| 1108 | VGSVGGAVTFPLKS | 0.000411972 | 0 | 0.04752689 |
| 1109 | VGSVGGAVTFPLKSKV | 0 | 0 | 0.002970281 |
| 1110 | VGSVGGAVTFPLKSKVK | 0 | 2.37458E-05 | 0.001019494 |
| 1111 | LRELLTTMGDRFTDE | 0 | 0.000719018 | 0 |
| 1112 | YLRELLTTM | 0.00115455 | 0 | 7.61016E-05 |
| 1113 | HPSVVPGSIAFSLPQ | 0 | 0 | 0.000836155 |
| 1114 | THPSVVPGSIAFSLPQ | 0 | 0 | 0.002949365 |
| 1115 | TEQQAGASASMAVD | 0 | 0 | 0.000444822 |
| 1116 | AEAAASALADADADL | 0 | 0.001762166 | 0 |
| 1117 | GKKAEAAASALADADADLE | 0 | 0.002045573 | 0 |
| 1118 | KAEAAASALADADADL | 0 | 0.001368034 | 0 |
| 1119 | KAEAAASALADADADLE | 0 | 0.002124929 | 0 |
| 1120 | ILWGEGSSAAIP | 0 | 0 | 0.004345875 |
| 1121 | APGTGLGGDAHFDEDERWTDG | 0.001696846 | 0.000622998 | 0 |
| 1122 | DLPASASLPAKAD | 1.68897E-05 | 0 | 0.006783926 |
| 1123 | DLPASASLPAKADL | 0 | 0 | 0.011165844 |
| 1124 | DLPASASLPAKADLL | 0 | 0 | 0.003280928 |
| 1125 | LPASASLPAKAD | 4.68651E-05 | 0 | 0.001277495 |
| 1126 | LPASASLPAKADL | 0 | 0 | 0.009219126 |
| 1127 | RVSGGAALAAQQS | 0 | 0 | 0.003230673 |
| 1128 | DAGAGIALNDH | 0.000103207 | 0 | 0.004027199 |
| 1129 | DAGAGIALNDHFVK | 0.000298873 | 0 | 0.006691697 |
| 1130 | DTHSSTFDAGAGIALNDHFVK | 0 | 0 | 0.009436626 |
| 1131 | FDAGAGIALNDHFVK | 0 | 0 | 0.001912788 |
| 1132 | SDTHSSTFDAGAGIALNDHFVK | 0 | 0 | 0.00355809 |
| 1133 | STFDAGAGIALNDHFVK | 0 | 9.50504E-05 | 0.084942505 |
| 1134 | TFDAGAGIALNDHFVK | 0 | 2.3205E-05 | 0.040582987 |
| 1135 | FDAGAGIALNDHF | 0 | 0 | 0.000373125 |
| 1136 | FDAGAGIALNDHFV | 0 | 0 | 0.00226133 |
| 1137 | STFDAGAGIALNDHFV | 0 | 0 | 0.01855388 |
| 1138 | TFDAGAGIALNDHFV | 0 | 0 | 0.022916608 |
| 1139 | HSSTFDAGAGIALNDHF | 0 | 0 | 0.001810001 |
| 1140 | SDTHSSTFDAGAGIALNDHF | 0 | 0 | 0.001108382 |
| 1141 | STFDAGAGIALNDHF | 0 | 0 | 0.007207723 |
| 1142 | TFDAGAGIALNDHF | 0 | 0 | 0.005401459 |
| 1143 | DFNSDTHSSTFDAGAGIALNDHFV | 0.000475849 | 0 | 0.000718533 |
| 1144 | DTHSSTFDAGAGIALNDH | 0 | 0 | 0.03899627 |
| 1145 | DTHSSTFDAGAGIALNDHF | 0 | 0 | 0.005860316 |
| 1146 | DTHSSTFDAGAGIALNDHFV | 0 | 0 | 0.003833569 |
| 1147 | FDAGAGIALNDH | 0 | 0 | 0.004485881 |
| 1148 | HSSTFDAGAGIALNDH | 0 | 0 | 0.022215351 |
| 1149 | SSTFDAGAGIALNDH | 0 | 0 | 0.006579376 |
| 1150 | STFDAGAGIALNDH | 0 | 0 | 0.039025572 |
| 1151 | TFDAGAGIALNDH | 0 | 0 | 0.034398146 |
| 1152 | THSSTFDAGAGIALNDH | 0 | 0 | 0.001460853 |
| 1153 | SDTHSSTFDAGAGIALNDH | 0 | 0 | 0.004644636 |
| 1154 | KWNPTAGVAF | 0 | 0 | 0.000856469 |
| 1155 | KHWEFDAPSPLPETTE | 0.002283039 | 0 | 0.000114739 |
| 1156 | DRHMVRFDPGTSIK | 0.001733527 | 0 | 0.00058591 |
| 1157 | SPFHFQPSAASLTANL | 0 | 0 | 0.001184891 |
| 1158 | DPRLYWQGGPALG | 0.002390759 | 0 | 0.009901935 |
| 1159 | DPRLYWQGGPALGR | 0 | 8.07228E-05 | 0.012859337 |
| 1160 | DPRLYWQGGPALGRS | 0 | 0.000275975 | 0.002151963 |
| 1161 | DPRLYWQGGPALGRSF | 0 | 0.001127865 | 0.140051498 |
| 1162 | DPRLYWQGGPALGRSFL | 0.000176823 | 6.56491E-05 | 0.013655479 |
| 1163 | GPQQDPRLYWQGGPALGRSFL | 0 | 0 | 0.070067832 |
| 1164 | GPQQDPRLYWQGGPALGRSFLH | 0 | 0 | 0.002585814 |
| 1165 | GPQQDPRLYWQGGPALGRSFLHGP | 0 | 0 | 0.003924802 |
| 1166 | PRLYWQGGPALGRSF | 4.32912E-05 | 0 | 0.002559569 |
| 1167 | PRLYWQGGPALGRSFL | 0 | 0 | 0.000455089 |
| 1168 | QDPRLYWQGGPALGRSF | 0 | 0 | 0.003536314 |
| 1169 | GPQQDPRLYWQGGPA | 0 | 0 | 0 |
| 1170 | GPQQDPRLYWQGGPALG | 0.000617073 | 8.80362E-05 | 0.057098551 |
| 1171 | GPQQDPRLYWQGGPALGR | 0 | 0 | 0.024658168 |
| 1172 | GPQQDPRLYWQGGPALGRS | 6.63355E-05 | 0.00011514 | 0.014097772 |
| 1173 | GPQQDPRLYWQGGPALGRSF | 0 | 0 | 0.140400695 |
| 1174 | GGPALGRSFLHGP | 0.00024161 | 1.92333E-05 | 0.000746488 |
| 1175 | GGPALGRSFLHGPE | 0 | 6.05648E-05 | 0.014649832 |
| 1176 | QGGPALGRSFLHGPE | 0 | 0 | 0.049176575 |
| 1177 | QGGPALGRSFLHGPELD | 0 | 0 | 0.000603221 |
| 1178 | QGGPALGRSFLHGP | 0.00010437 | 0.00184392 | 0.03107123 |
| 1179 | KVTLTSEEEARLK | 2.66529E-06 | 0.001144627 | 0 |
| 1180 | LTSEEEARL | 0 | 0.000142079 | 0 |
| 1181 | LTSEEEARLK | 0 | 0.002916966 | 3.91064E-06 |
| 1182 | VKVTLTSEEEARLK | 0.000174655 | 0.001079811 | 2.06247E-05 |
| 1183 | DKQREEYQPATPGLG | 0 | 0 | 0.001130436 |
| 1184 | QREEYQPATPGLG | 0 | 0.000202548 | 0.018650108 |
| 1185 | NRIEYEPGTGALAVFPN | 0 | 0.000105577 | 0.008078679 |
| 1186 | RIEYEPGTGALAVFPN | 0 | 0 | 0.001020264 |
| 1187 | RVDDFEAGAAAGAAPG | 0 | 0 | 0.001697408 |
| 1188 | HFGISAATGGLADDH | 0 | 0 | 0.000150861 |
| 1189 | HFGISAATGGLADDHDV | 0 | 0.000289605 | 0.000691257 |
| 1190 | LRGVGAAATAVTQAL | 0 | 2.92466E-05 | 0.003025101 |
| 1191 | KPGLDTYSLGGGGAARVR | 0 | 0 | 0.00278971 |
| 1192 | TYSLGGGGAARVR | 0 | 3.76946E-05 | 0.001382396 |
| 1193 | AEAKESGPTTY | 0 | 0.000235924 | 0 |
| 1194 | GKQVGSGVTTDQVQ | 0 | 0.000178137 | 0 |
| 1195 | GKQVGSGVTTDQVQAEAKESGPT | 0 | 0.000955622 | 0 |
| 1196 | KQVGSGVTTDQVQ | 0 | 0.000165626 | 0 |
| 1197 | VGSGVTTDQVQ | 7.37851E-05 | 0.001479078 | 0 |
| 1198 | GSGVTTDQVQAEAK | 0.000174374 | 0.006081179 | 0 |
| 1199 | GSGVTTDQVQAEAKE | 0 | 0.007199492 | 0 |
| 1200 | GSGVTTDQVQAEAKES | 0 | 0.001891395 | 0 |
| 1201 | GVTTDQVQAE | 0 | 0.001318029 | 0 |
| 1202 | GVTTDQVQAEAK | 0 | 0.01655307 | 0.000607415 |
| 1203 | SGVTTDQVQAEAK | 2.85697E-05 | 0.015269218 | 6.3752E-05 |
| 1204 | VGSGVTTDQVQAEAKES | 0 | 0.012085016 | 0 |
| 1205 | VGSGVTTDQVQAEAKESGP | 0 | 0.000509919 | 0.001113729 |
| 1206 | VGSGVTTDQVQAEAKESGPT | 0 | 0.010726614 | 0 |
| 1207 | VGSGVTTDQVQAE | 0 | 0.001607732 | 6.76427E-05 |
| 1208 | VGSGVTTDQVQAEAK | 0 | 0.010050873 | 0.002416099 |
| 1209 | VGSGVTTDQVQAEAKE | 0 | 0.033579073 | 2.57695E-05 |
| 1210 | GVTTDQVQAEAKE | 0 | 0.030432035 | 0.000316974 |
| 1211 | GVTTDQVQAEAKES | 0 | 0.000912423 | 4.19322E-05 |
| 1212 | GVTTDQVQAEAKESG | 0 | 0.000261405 | 0 |
| 1213 | GVTTDQVQAEAKESGP | 0 | 0.00114203 | 0 |
| 1214 | GVTTDQVQAEAKESGPT | 2.22955E-05 | 0.005846893 | 0 |
| 1215 | GVTTDQVQAEAKESGPTT | 0 | 0.001496932 | 0 |
| 1216 | SGVTTDQVQAEAKESGPTT | 0.000104811 | 0.001879186 | 0 |
| 1217 | VTTDQVQAEAK | 0 | 0.007638933 | 0 |
| 1218 | VTTDQVQAEAKE | 0 | 0.020158402 | 0 |
| 1219 | VTTDQVQAEAKES | 0 | 0.010569394 | 0.000322475 |
| 1220 | VTTDQVQAEAKESGP | 0 | 0.000343462 | 0.000741782 |
| 1221 | VTTDQVQAEAKESGPT | 0 | 0.007066011 | 0 |
| 1222 | VTTDQVQAEAKESGPTT | 0 | 0.0015171 | 0 |
| 1223 | SGVTTDQVQAE | 0 | 0.00213862 | 8.92474E-05 |
| 1224 | SGVTTDQVQAEA | 0 | 0.001214571 | 0 |
| 1225 | SGVTTDQVQAEAKE | 0 | 0.040833095 | 0 |
| 1226 | SGVTTDQVQAEAKES | 0 | 0.015078612 | 0.000499243 |
| 1227 | SGVTTDQVQAEAKESG | 0 | 0.000535613 | 0 |
| 1228 | SGVTTDQVQAEAKESGP | 0 | 0.000781745 | 0 |
| 1229 | SGVTTDQVQAEAKESGPT | 0 | 0.007709853 | 0 |
| 1230 | KTSQSVSGEEIDAMDVQ | 0.001974299 | 0 | 0 |
| 1231 | SQSVSGEEIDAMDVQ | 0.004458312 | 0 | 0 |
| 1232 | TSQSVSGEEIDAMDVQ | 0.004542081 | 0 | 0 |
| 1233 | ASRDMAGAQAAA | 0 | 0 | 0.001898504 |
| 1234 | ASRDMAGAQAAAVA | 0.000139674 | 0 | 0.01239342 |
| 1235 | ASRDMAGAQAAAVAL | 5.74928E-05 | 0 | 0.002922513 |
| 1236 | ASRDMAGAQAAAVALN | 0 | 2.56296E-05 | 0.009401025 |
| 1237 | GASRDMAGAQAAAVALN | 0 | 0 | 0.000681181 |
| 1238 | GASRDMAGAQAAAVA | 1.82296E-05 | 0 | 0.0027179 |
| 1239 | DIVMTQSPLSLPV | 0.000168718 | 0.002833708 | 0 |
| 1240 | DIVMTQSPLSLPVTP | 0.000865392 | 0.05838657 | 0.003786388 |
| 1241 | DIVMTQSPLSLPVTPG | 0.000766222 | 0.021180702 | 0.000628502 |
| 1242 | DIVMTQSPLSLPVTPGEP | 0 | 0.000727564 | 0 |
| 1243 | DIVMTQSPLSLPVTPGEPA | 0 | 0.01053573 | 0 |
| 1244 | IVMTQSPLSLPVTP | 0 | 0.000495144 | 0 |
| 1245 | TQSPLSLPVTPGEP | 0 | 0.002093511 | 0 |
| 1246 | TQSPLSLPVTPGEPA | 0 | 0.00064692 | 0 |
| 1247 | GITPGQAAAIAS | 0 | 0 | 0.009102982 |
| 1248 | LPVTPGEPASIS | 0 | 0 | 0.031779297 |
| 1249 | SLPVTPGEPASIS | 0 | 0 | 0.00565213 |
| 1250 | SPLSLPVTPGEPASIS | 0 | 0 | 0.004552676 |
| 1251 | ITPGQAAAIASTTIMVP | 0 | 0 | 0.000899539 |
| 1252 | TPGQAAAIASTT | 0 | 0 | 0.002671039 |
| 1253 | TPGQAAAIASTTI | 0 | 0 | 0.001534885 |
| 1254 | TPGQAAAIASTTIM | 3.3685E-05 | 0 | 0.01133626 |
| 1255 | TPGQAAAIASTTIMVP | 0 | 0.000121151 | 0.012098621 |
| 1256 | DPGGSVPSGEASAAFE | 0.000225798 | 0 | 0.005403563 |
| 1257 | DPGGSVPSGEASAAFER | 1.56572E-05 | 0 | 0.04200027 |
| 1258 | DPGGSVPSGEASAAFERL | 0 | 0 | 0.007122524 |
| 1259 | GGSVPSGEASAAFER | 7.26516E-05 | 3.44659E-05 | 0.003915295 |
| 1260 | SPEPASLPAEDIS | 0.000104828 | 0.001373881 | 0 |
| 1261 | SPEPASLPAEDISA | 0 | 0.001306021 | 0 |
| 1262 | SPSSPEPASLPAEDISA | 0.000162473 | 0.001693533 | 0.00012022 |
| 1263 | SPSSPEPASLPAEDIS | 0 | 0.000571333 | 0 |
| 1264 | GEPCYIRDSGVAPTDPGPE | 0 | 0 | 0.004068947 |
| 1265 | SKGLAAAEPTAN | 0 | 0 | 5.48041E-05 |
| 1266 | SKGLAAAEPTANGGL | 0 | 0 | 0.001670613 |
| 1267 | EHRGSEASPALPG | 0.000294547 | 0 | 0.000257502 |
| 1268 | GLEHRGSEASPALPG | 8.25363E-06 | 0 | 0.018990581 |
| 1269 | QGLEHRGSEASPALPG | 0 | 0 | 0.01198618 |
| 1270 | QGLEHRGSEASPALPGLK | 0 | 0 | 0.005867504 |
| 1271 | GLEHRGSEASPAL | 0.000469094 | 3.19851E-05 | 0.001422305 |
| 1272 | GLEHRGSEASPALP | 0.000486472 | 0.000437359 | 0.004379577 |
| 1273 | QGLEHRGSEASPALP | 0.00038312 | 0.000115603 | 0.005358859 |
| 1274 | QGLEHRGSEASPA | 0 | 0 | 0.001288341 |
| 1275 | QGLEHRGSEASPAL | 0 | 0 | 0.002461603 |
| 1276 | ELRRVVAGEGSGSVS | 4.37439E-05 | 0.000235477 | 0.025949168 |
| 1277 | ELRRVVAGEGSGSVSL | 0 | 0 | 0.000329188 |
| 1278 | LRRVVAGEGSGSVS | 0 | 0 | 0.025932157 |
| 1279 | RRVVAGEGSGSVS | 0 | 0 | 0.006875321 |
| 1280 | RVVAGEGSGSV | 0 | 0 | 0.000261981 |
| 1281 | RVVAGEGSGSVS | 0 | 0 | 0.016801046 |
| 1282 | LRRVVAGEGSGSVSL | 0 | 0 | 0.001372061 |
| 1283 | LPFNVIRHAVLLPAD | 0 | 0 | 0.006189135 |
| 1284 | LPFNVIRHAVLLPADSPT | 0 | 0 | 0.007341187 |
| 1285 | QPGQLPFNVIRHAVLLPADSPT | 0 | 0 | 0.001578292 |
| 1286 | QPGQLPFNVIRHAVLLPADSPTAPH | 0 | 0 | 0.003832482 |
| 1287 | QPGQLPFNVIRHAVLLPAD | 0 | 0 | 0.002925506 |
| 1288 | ATNVAAAFEEAVRR | 0.002069054 | 0.00078185 | 0.002070309 |
| 1289 | DATNVAAAFEEAVRR | 0.009661017 | 0.002542061 | 0.001931136 |
| 1290 | DATNVAAAFEEAVRRV | 0.003963674 | 0.000934244 | 0.00422489 |
| 1291 | DATNVAAAFEEAVRRVL | 0.001277223 | 0.000248176 | 0.001881076 |
| 1292 | DATNVAAAFEEAVR | 0.006653078 | 0.002794717 | 0.005179714 |
| 1293 | HPGQISAGYA | 0 | 0 | 0 |
| 1294 | HPGQISAGYAPVLD | 0 | 0 | 0.007417568 |
| 1295 | LVTGGEIASTFDHP | 0 | 0.000434831 | 0.003891265 |
| 1296 | LVTGGEIASTFDHPE | 0 | 0.000197281 | 0.001721237 |
| 1297 | VTGGEIASTFDHP | 0 | 0 | 0.001818771 |
| 1298 | GPATIKTFRTPELPPS | 0 | 0.004547846 | 0 |
| 1299 | TPGGEGPATIKTFRTPELPPS | 0 | 0.006554284 | 0 |
| 1300 | TPGGEGPATIKTFRTPELPP | 0 | 0.003287094 | 0 |
| 1301 | EPSADAPAALYQ | 0.003097752 | 0 | 0 |
| 1302 | EPSADAPAALYQT | 0.009056678 | 0 | 0 |
| 1303 | ILNEPSADAPAALYQT | 0.003040361 | 0 | 0 |
| 1304 | ILNEPSADAPAALYQ | 0.005425304 | 0 | 0 |
| 1305 | FVHAVEQGGTQASA | 0 | 0 | 0.001715997 |
| 1306 | FPDDLPGSNTAAPVQ | 0 | 0.000558113 | 0 |
| 1307 | LPGSNTAAPVQETLH | 0 | 0 | 0.001160251 |
| 1308 | KVEITASQPTSTIILH | 0 | 0 | 0.006181843 |
| 1309 | TKVEITASQPTSTIILH | 0 | 0 | 0.001360329 |
| 1310 | GSLKPNPASPLPASPYG | 0 | 0 | 0.004010875 |
| 1311 | GSTAPVGGGAFPTIVER | 0 | 0 | 0.001793298 |
| 1312 | STAPVGGGAFPTIVE | 0 | 0 | 0.005610402 |
| 1313 | STAPVGGGAFPTIVER | 0 | 0 | 0.00469065 |
| 1314 | LGQAGSSIPSTWPR | 0 | 0.001070534 | 0.001790799 |
| 1315 | DRNPYYGGESASITPL | 0 | 0 | 0.003093664 |
| 1316 | WAEGTAVATRG | 9.5034E-05 | 1.23497E-05 | 0.006729251 |
| 1317 | WWAEGTAVATRG | 0 | 0 | 0.000551292 |
| 1318 | EEAGPLPTKVNLAHS | 0 | 0 | 0.001262918 |
| 1319 | LDPTASISAKVN | 0 | 0 | 0.001053901 |
| 1320 | LDPTASISAKVNN | 0 | 0.000297182 | 0.001237823 |
| 1321 | AAAFLGGVVR | 0 | 0 | 0.002396956 |
| 1322 | DSSFLGGVVH | 0 | 0 | 0.003407424 |
| 1323 | WDSSFLGGVVH | 0 | 1.97643E-05 | 0.030993116 |
| 1324 | WDSSFLGGVVHLE | 0 | 6.44921E-05 | 0.011899561 |
| 1325 | WDSSFLGGVVHLEAG | 0 | 0 | 0.002489553 |
| 1326 | WDSSFLGGVVHLEAGE | 0 | 0 | 0.001417185 |
| 1327 | DTGSFVGGIVL | 0 | 0 | 0.003109642 |
| 1328 | NPPYVPGVVGGTL | 0 | 0 | 0.000672976 |
| 1329 | NPPYVPGVVGGTLQ | 0 | 0 | 0.004181865 |
| 1330 | NPPYVPGVVGGTLQAAT | 0 | 0 | 0.001068105 |
| 1331 | AFLGALASLGSLN | 0.000564856 | 0 | 0.000962067 |
| 1332 | DVAAFLGALASLGSLN | 0 | 0 | 0.00379996 |
| 1333 | VAAFLGALASLGSLN | 0 | 0 | 0.001536413 |
| 1334 | FLGALASLGSLNIPYK | 0 | 0.000463865 | 0 |
| 1335 | LGALASLGSLNIPYK | 0 | 0.002867665 | 0 |
| 1336 | GPPNTSYMPGMPGGISPYPS | 0 | 0 | 0.001327101 |
| 1337 | GTYVSSVPR | 0 | 0.000638308 | 0.00079128 |
| 1338 | HDASFVAAIPSTQ | 0 | 0 | 0.004870125 |
| 1339 | HDASFVAAIPSTQVV | 0 | 5.42926E-05 | 0.008240462 |
| 1340 | AFSAVGALEAQLK | 0.000523193 | 0 | 5.57419E-05 |
| 1341 | FPFLAYSGIPAVS | 9.71676E-05 | 0 | 0.16401358 |
| 1342 | FPFLAYSGIPAVSF | 0.000664559 | 0 | 0.04973673 |
| 1343 | LAYSGIPAVS | 0 | 0 | 0.00516035 |
| 1344 | LDNAAFPFLAYSGIPAVS | 0 | 0 | 0.001142938 |
| 1345 | LAYSGIPAVSF | 0 | 0 | 0.010854944 |
| 1346 | AGGVSGGHINPA | 0 | 0 | 0.00080486 |
| 1347 | AGGVSGGHINPAVS | 0 | 0 | 0.003361054 |
| 1348 | GGVSGGHINPAVS | 0 | 0 | 0.002148995 |
| 1349 | GVSGGHINPAVS | 0 | 0 | 0.002758005 |
| 1350 | VSGGHINPAVS | 0.000118517 | 0 | 0.002042652 |
| 1351 | GGSVSSVSSSRLQ | 7.39803E-05 | 0.000539338 | 0 |
| 1352 | ALREAAAAGLPGLAR | 0 | 0 | 0.001881639 |
| 1353 | EAAAAGLPGLAR | 0 | 0 | 0.010316202 |
| 1354 | LREAAAAGLPGLAR | 0 | 0 | 0.011867118 |
| 1355 | REAAAAGLPGLAR | 0 | 1.57911E-05 | 0.004394374 |
| 1356 | LREAAAAGLPGLARL | 0 | 0 | 0.002570079 |
| 1357 | EAAAAGLPGLARL | 0.000470594 | 0 | 0.000443284 |
| 1358 | EAAAAGLPGLARLFS | 0 | 0 | 0.000756751 |
| 1359 | DPSGSVASISH | 0 | 4.95979E-05 | 0.004668399 |
| 1360 | DPSGSVASISHQ | 1.98086E-05 | 1.76529E-05 | 0.001569635 |
| 1361 | DPSGSVASISHQE | 0 | 1.87175E-05 | 0.009049964 |
| 1362 | DPSGSVASISHQEQ | 0.000656837 | 0.000393348 | 0 |
| 1363 | DPSGSVASISHQEQL | 0 | 0.00066321 | 0.000146271 |
| 1364 | NQDPSGSVASISHQE | 0 | 0 | 0.004139789 |
| 1365 | NQDPSGSVASISHQEQ | 0 | 0 | 0.002245821 |
| 1366 | PSGSVASISHQE | 0 | 0 | 0.000837501 |
| 1367 | PSGSVASISHQEQ | 0 | 6.42926E-06 | 0.002792715 |
| 1368 | QDPSGSVASISHQEQ | 0 | 1.08734E-05 | 0.009194378 |
| 1369 | LNQDPSGSVASISH | 0 | 0 | 0.001164306 |
| 1370 | LNQDPSGSVASISHQ | 0 | 0 | 0.000554804 |
| 1371 | LNQDPSGSVASISHQE | 0 | 7.19309E-05 | 0.010985273 |
| 1372 | LNQDPSGSVASISHQEQ | 0 | 6.86945E-05 | 0.025055259 |
| 1373 | HPEPASGLAALAKV | 0 | 0 | 0.000575453 |
| 1374 | GVGLGAASAVTGIT | 0 | 0 | 0.001190482 |
| 1375 | GVGLGAASAVTGITT | 0 | 3.97168E-05 | 0.002812179 |
| 1376 | LSPSLSPLGGQAAGAMGG | 0 | 0 | 0.05937932 |
| 1377 | LSPSLSPLGGQAAGAMGGLAP | 0 | 0 | 0.01299263 |
| 1378 | WAAGTVAAMSS | 0 | 0 | 0.000651557 |
| 1379 | APGMEPSGSVAGLGELDPG | 0 | 0 | 0.000898296 |
| 1380 | LPYVQYFGGVSALSK | 0 | 0 | 0.001444563 |
| 1381 | LPYVQYFGGVSALSKQ | 0 | 0 | 0.002500372 |
| 1382 | LPYVQYFGGVSALSKQQ | 0 | 0 | 0.00887053 |
| 1383 | LPYVQYFGGVSALSKQQF | 0 | 0 | 0.011371845 |
| 1384 | YFGGVSALSKQ | 0 | 0 | 0.005106001 |
| 1385 | YFGGVSALSKQQ | 0 | 0.000271994 | 0.008658215 |
| 1386 | YFGGVSALSKQQF | 0 | 0 | 0.004004814 |
| 1387 | YFGGVTALSREQ | 0 | 0 | 0.002218396 |
| 1388 | YFGGVTALSREQF | 0 | 0 | 0.001362734 |
| 1389 | YSGYFGGVTALSREQF | 0 | 0.00040216 | 0.00576586 |
| 1390 | YSGYFGGVTALSREQFF | 0 | 0 | 0.000496154 |
| 1391 | YSGYFGGVTALSRE | 0 | 0 | 0.001129765 |
| 1392 | YSGYFGGVTALSREQ | 0 | 0 | 0.006395428 |
| 1393 | LPYPQYFGGVSALTPDQ | 0.000110827 | 0.00012141 | 0.005717146 |
| 1394 | LPYPQYFGGVSALTPDQY | 0 | 0.001309372 | 0.001669946 |
| 1395 | MKLPKPPKPVSKMRMATPLLMQALP | 0.019408024 | 0.000211095 | 0.000655785 |
| 1396 | KLPKPPKPVSKMRMATPLLMQALP | 0.065753594 | 0.002445822 | 0.008284007 |
| 1397 | LPKPPKPVSKMRMATPLLMQALP | 9.242272254 | 1.540305944 | 1.061825974 |
| 1398 | LPKPPKPVSKMRMATPLLMQAL | 0.030908094 | 0.003014499 | 0.007002536 |
| 1399 | LPKPPKPVSKMRMATPLLMQA | 0.280782841 | 0.021527776 | 0.60755328 |
| 1400 | LPKPPKPVSKMRMATPLLMQ | 0.046563439 | 0.008713386 | 0.017950829 |
| 1401 | LPKPPKPVSKMRMATPLLM | 0.061357722 | 0.00710698 | 0.003211192 |
| 1402 | PKPPKPVSKMRMATPLLMQALP | 5.48977529 | 0.734724488 | 0.431793825 |
| 1403 | PKPPKPVSKMRMATPLLMQAL | 0.20704714 | 0.015944148 | 0.00330612 |
| 1404 | PKPPKPVSKMRMATPLLMQA | 0.164205972 | 0.007052671 | 0.385392021 |
| 1405 | PKPPKPVSKMRMATPLLMQ | 0.017457911 | 0.002114016 | 0.01109993 |
| 1406 | PKPPKPVSKMRMATPLLM | 0.015287583 | 0.000380649 | 0.000263379 |
| 1407 | PKPPKPVSKMRMATPLL | 0.000511521 | 0 | 0 |
| 1408 | KPPKPVSKMRMATPLLMQALP | 1.545677612 | 0.377918485 | 0.120559177 |
| 1409 | KPPKPVSKMRMATPLLMQAL | 0.003357393 | 0.001049113 | 0 |
| 1410 | KPPKPVSKMRMATPLLMQA | 0.134931788 | 0.016163597 | 0.116919677 |
| 1411 | KPPKPVSKMRMATPLLMQ | 0.006475102 | 0.002384305 | 0.003846566 |
| 1412 | KPPKPVSKMRMATPLLM | 0.003998532 | 0.000433612 | 0.000296721 |
| 1413 | PPKPVSKMRMATPLLMQALP | 0.001728132 | 0.002079083 | 0 |
| 1414 | PPKPVSKMRMATPLLMQA | 0 | 0.001241956 | 0 |
| 1415 | KPVSKMRMATPLLMQALP | 0.085722745 | 0.002746983 | 0 |
| 1416 | KPVSKMRMATPLLMQA | 0.035999948 | 0.004926979 | 0.002360056 |
| 1417 | KPVSKMRMATPLLMQ | 0 | 0.000257815 | 0 |
| 1418 | VSKMRMATPLLMQALP | 0.01520958 | 0 | 9.34223E-05 |
| 1419 | SKMRMATPLLMQALP | 0.0050204 | 0 | 0.000471612 |
| 1420 | KMRMATPLLMQALP | 0.004570843 | 0.000759261 | 0 |
| 1421 | MRMATPLLMQALP | 0.006653336 | 7.19999E-05 | 0.000255885 |
| 1422 | MRMATPLLM | 0.00071784 | 1.78303E-05 | 0 |
| 1423 | KLPKPPKPVSKMRMATPLLMQALPM | 0.079734139 | 0.002129159 | 0.003981037 |
| 1424 | LPKPPKPVSKMRMATPLLMQALPMG | 0.032644576 | 0.008679643 | 0.000704138 |
| 1425 | LPKPPKPVSKMRMATPLLMQALPM | 1.142797278 | 0.242893499 | 0.077075304 |
| 1426 | PKPPKPVSKMRMATPLLMQALPMG | 0.013845488 | 0.0053137 | 0.000597591 |
| 1427 | PKPPKPVSKMRMATPLLMQALPM | 0.402914384 | 0.105230403 | 0.044553639 |
| 1428 | KPPKPVSKMRMATPLLMQALPMGAL | 0.088447485 | 0 | 0 |
| 1429 | KPPKPVSKMRMATPLLMQALPMG | 0.063854664 | 0 | 0 |
| 1430 | KPPKPVSKMRMATPLLMQALPM | 2.555828426 | 0.094035785 | 0.000127943 |
| 1431 | PPKPVSKMRMATPLLMQALPMGAL | 0.006465711 | 0 | 0 |
| 1432 | PKPVSKMRMATPLLMQALPMGALPQ | 0.002783905 | 0 | 0 |
| 1433 | VSKMRMATPLLMQALPMGALPQGPM | 0.004235475 | 0 | 0 |
| 1434 | VSKMRMATPLLMQALPMGALPQGP | 0.00252871 | 0 | 0 |
| 1435 | VSKMRMATPLLMQALPMGALPQ | 0.499737877 | 0.009582753 | 0 |
| 1436 | VSKMRMATPLLMQALPMGALP | 0.067323898 | 0.000285973 | 0 |
| 1437 | VSKMRMATPLLMQALPMGAL | 0.015920941 | 0 | 0 |
| 1438 | VSKMRMATPLLMQALPMG | 0.00402741 | 0.00034479 | 0 |
| 1439 | VSKMRMATPLLMQALPM | 0.337134191 | 0.002868842 | 0.000336101 |
| 1440 | KMRMATPLLMQALPMGALPQGPMQ | 0.009251345 | 0 | 0 |
| 1441 | KMRMATPLLMQALPMGALPQGPM | 0.010733281 | 0 | 0 |
| 1442 | KMRMATPLLMQALPMGALPQGP | 0.008036884 | 0.000174055 | 0 |
| 1443 | KMRMATPLLMQALPMGALPQ | 2.288790483 | 0.174956733 | 8.56746E-05 |
| 1444 | KMRMATPLLMQALPMGALP | 0.355303282 | 0.031923976 | 0 |
| 1445 | KMRMATPLLMQALPMGAL | 0.162203538 | 0.001473201 | 0 |
| 1446 | KMRMATPLLMQALPMGA | 0.002239929 | 0 | 0 |
| 1447 | KMRMATPLLMQALPMG | 0.017364773 | 0.000180693 | 0 |
| 1448 | KMRMATPLLMQALPM | 0.244576476 | 0.007194376 | 0 |
| 1449 | MRMATPLLMQALPMGALPQGPMQ | 0.006626521 | 0 | 0 |
| 1450 | MRMATPLLMQALPMGALPQGPM | 0.007136847 | 0 | 0 |
| 1451 | MRMATPLLMQALPMGALPQ | 2.025306872 | 0.134314321 | 0.00091474 |
| 1452 | MRMATPLLMQALPMGALP | 0.374100157 | 0.0365485 | 6.4876E-05 |
| 1453 | MRMATPLLMQALPMGAL | 0.133050936 | 0.002560589 | 0 |
| 1454 | MRMATPLLMQALPMGA | 0.002544648 | 0 | 0 |
| 1455 | MRMATPLLMQALPMG | 0.026242644 | 0.000452036 | 0 |
| 1456 | MRMATPLLMQALPM | 0.619103528 | 0.018162091 | 0 |
| 1457 | RMATPLLMQALPMGALPQGPMQ | 0.011488987 | 0 | 8.2829E-05 |
| 1458 | RMATPLLMQALPMGALPQGPM | 0.032962452 | 0.000671199 | 0.000844629 |
| 1459 | RMATPLLMQALPMGALPQGP | 0.014238435 | 0 | 0 |
| 1460 | RMATPLLMQALPMGALPQ | 5.469415793 | 0.441804305 | 0.003087207 |
| 1461 | RMATPLLMQALPMGALP | 2.685092364 | 0.497688349 | 8.93468E-05 |
| 1462 | RMATPLLMQALPMGAL | 1.504859517 | 0.058763564 | 0.000254173 |
| 1463 | RMATPLLMQALPMGA | 0.066852615 | 0.001203339 | 0.001645235 |
| 1464 | RMATPLLMQALPMG | 0.18326819 | 0.00617916 | 0.000135306 |
| 1465 | RMATPLLMQALPM | 1.682285692 | 0.065497172 | 0.000304374 |
| 1466 | RMATPLLMQ | 0.002102992 | 0 | 0 |
| 1467 | MATPLLMQALPMGALPQGPMQ | 0.005849268 | 0.000342108 | 0.071075769 |
| 1468 | MATPLLMQALPMGALPQGPM | 0.010876489 | 0.000343826 | 0.056505249 |
| 1469 | MATPLLMQALPMGALPQGP | 0.004105222 | 0.000899618 | 0.004047907 |
| 1470 | MATPLLMQALPMGALPQ | 3.359522432 | 0.128761626 | 0.00050773 |
| 1471 | MATPLLMQALPMGALP | 2.348370941 | 0.390278869 | 0.000277999 |
| 1472 | MATPLLMQALPMGAL | 1.465955013 | 0.077888925 | 0 |
| 1473 | MATPLLMQALPMGA | 0.344057625 | 0.002969765 | 0 |
| 1474 | MATPLLMQALPMG | 0.287311278 | 0.008276127 | 0 |
| 1475 | MATPLLMQALPM | 1.830541561 | 0.037042763 | 0 |
| 1476 | MATPLLMQALP | 0.008713852 | 0.000380358 | 0 |
| 1477 | MATPLLMQ | 0.006163272 | 0 | 0 |
| 1478 | ATPLLMQALPMGALPQGPMQ | 0.008367862 | 0.001685271 | 0.403091399 |
| 1479 | ATPLLMQALPMGALPQGPM | 0.034738069 | 0.001171479 | 0.670731984 |
| 1480 | ATPLLMQALPMGALPQGP | 0.013285902 | 0.005390596 | 0.651334626 |
| 1481 | ATPLLMQALPMGALPQG | 0.003255011 | 0.000435047 | 0.014999226 |
| 1482 | ATPLLMQALPMGALPQ | 1.006572148 | 0.040434875 | 0.316673519 |
| 1483 | ATPLLMQALPMGALP | 0.584964356 | 0.039846756 | 0.0021949 |
| 1484 | ATPLLMQALPMGAL | 0.083491493 | 0.01516713 | 0 |
| 1485 | ATPLLMQALPMGA | 0.020133024 | 0.000442469 | 0 |
| 1486 | ATPLLMQALPMG | 0.010320336 | 0.00044797 | 0.002562357 |
| 1487 | ATPLLMQALPM | 0.017740527 | 7.71054E-05 | 0 |
| 1488 | TPLLMQALPMGALPQGPMQ | 0.003940714 | 0.000525145 | 0.236892203 |
| 1489 | TPLLMQALPMGALPQGPM | 0.023920504 | 0.001504879 | 1.422181525 |
| 1490 | TPLLMQALPMGALPQGP | 0.030033207 | 0.00074644 | 1.509901087 |
| 1491 | TPLLMQALPMGALPQG | 0.00012744 | 0 | 0.034205544 |
| 1492 | TPLLMQALPMGALPQ | 0.472528398 | 0.006953364 | 2.496017718 |
| 1493 | TPLLMQALPMGALP | 0.257933014 | 0.005570127 | 0.019461385 |
| 1494 | TPLLMQALPMGAL | 0 | 0 | 0 |
| 1495 | TPLLMQALPMG | 0.001740051 | 0 | 0.003216601 |
| 1496 | TPLLMQALPM | 0.001507258 | 0 | 0.000185167 |
| 1497 | LLMQALPMGALPQGPM | 0 | 0 | 0.000869269 |
| 1498 | LMQALPMGALPQGPMQ | 0.002063587 | 0.000265825 | 0.073597621 |
| 1499 | LMQALPMGALPQGPM | 0.016098822 | 0.000295342 | 0.196023979 |
| 1500 | LMQALPMGALPQGP | 0.009073316 | 0.000268727 | 0.642247801 |
| 1501 | LMQALPMGALPQG | 4.98006E-05 | 0 | 0.004819085 |
| 1502 | LMQALPMGALPQ | 0.164655077 | 0.000302274 | 0.568926572 |
| 1503 | LMQALPMGALP | 0.008015315 | 0.000125043 | 0.000103101 |
| 1504 | MQALPMGALPQGPM | 0 | 0 | 0.004279853 |
| 1505 | MQALPMGALPQ | 0.002915409 | 0 | 0 |
| 1506 | QALPMGALPQGPMQ | 0 | 0.00018164 | 0.012344327 |
| 1507 | QALPMGALPQGPM | 0 | 9.15324E-05 | 0.010713873 |
| 1508 | QALPMGALPQGP | 0.000105049 | 0 | 0.002095967 |
| 1509 | QALPMGALPQ | 0.001927166 | 0 | 0.000188693 |
| 1510 | QALPMGALP | 0.002496317 | 0 | 4.61471E-05 |
| 1511 | ALPMGALPQGPMQ | 0 | 0 | 0.009667757 |
| 1512 | ALPMGALPQGPM | 0.00053414 | 0 | 0.02883729 |
| 1513 | ALPMGALPQGP | 0.000469052 | 0.000439457 | 0.030307879 |
| 1514 | ALPMGALPQ | 0.013481986 | 0.000259787 | 0.037200028 |
| 1515 | LPMGALPQ | 0.000843502 | 0.001285326 | 0.000584037 |
| 1516 | LPVGTMPEGTIV | 0 | 0.001126706 | 0 |
| 1517 | VLPVGTMPEGTIV | 0 | 0.001203309 | 0 |
| 1518 | RAITIAGVPQSVTE | 0 | 0 | 0.003596834 |
| 1519 | LPLIVNTVALPDEL | 0 | 0.000805699 | 0 |
| 1520 | DKDTHTSLGVPTLSIVA | 0 | 0 | 0.001760728 |
| 1521 | DTHTSLGVPTLSIVA | 0 | 9.18864E-05 | 0.003301047 |
| 1522 | IPLPLGTVTI | 0 | 0 | 0.000482017 |
| 1523 | IPLPLGTVTIL | 0 | 0.00018331 | 0 |
| 1524 | SLSVSTLPLPEVQ | 0 | 0.00306343 | 0 |
| 1525 | IPSMAIGAIAGRIV | 0 | 0 | 0.00058037 |
| 1526 | IPSMAIGAIAGRIVG | 0 | 0 | 0.004463352 |
| 1527 | VDNHIGISIAGLTADAR | 0 | 0 | 0.000270471 |
| 1528 | EPLAPALGEAALAALA | 0 | 0 | 0.00029176 |
| 1529 | ESVAAALSPLG | 0 | 0 | 0.000805897 |
| 1530 | VGESVAAALSPLG | 0.000637063 | 0 | 0.009618052 |
| 1531 | VGESVAAALSPLGIE | 0 | 0.000212936 | 0.016103251 |
| 1532 | VGESVAAALSPLGIEV | 0 | 0 | 0.001227792 |
| 1533 | VGESVAAALSPLGIEVD | 0 | 0 | 0.029363716 |
| 1534 | VHAVAGAVGSVT | 0 | 0 | 0.000443962 |
| 1535 | VPHPSSLSGSLPGMSR | 0.000674961 | 0 | 0.000935852 |
| 1536 | PGMGAMGGMGGGMGGGMF | 0.00052867 | 0.001267124 | 0.001600661 |
| 1537 | IPIAVGGALAGL | 0 | 0 | 0.017339172 |
| 1538 | IPIAVGGALAGLV | 0 | 0 | 0.001898549 |
| 1539 | IPIAVGGALAGLVL | 0 | 0 | 0.020067768 |
| 1540 | PIAVGGALAGLVL | 0 | 0 | 0.008813212 |
| 1541 | PIAVGGALAGLVLI | 0 | 0 | 0.012342629 |
| 1542 | LITLAVPSAVAL | 0 | 0 | 0.001307581 |
| 1543 | VPGSVASVLLQGL | 0 | 8.22237E-05 | 0 |
| 1544 | VPGSVASVLLQGLA | 0 | 0.000486654 | 0 |
| 1545 | VPGSVASVLLQGLAG | 0 | 0.002424258 | 0 |
| 1546 | DVYKIGGIGTVPVG | 0.00120806 | 0 | 0 |
| 1547 | DVYKIGGIGTVPVGR | 0.001385771 | 0 | 0 |
| 1548 | DVYKIGGIGTVPVGRV | 0.003076546 | 0 | 0 |
| 1549 | GIGTVPVGRVE | 0 | 0.014928045 | 0 |
| 1550 | IGGIGTVPVGRVE | 0 | 0.027709601 | 6.1499E-05 |
| 1551 | IGGIGTVPVGRVET | 0 | 0.000977264 | 0.000269173 |
| 1552 | IGGIGTVPVGR | 0.000132885 | 0 | 0 |
| 1553 | KIGGIGTVPVGRVE | 0 | 0.006093447 | 0 |
| 1554 | EKVSTLPAITLK | 0 | 0.000701468 | 0 |
| 1555 | LPYKEFFGGVSGLTVEQ | 0 | 0 | 0.00098539 |
| 1556 | LPYKEFFGGVSGLTVEQF | 0 | 0 | 0.003399541 |
| 1557 | AQRWAAGLGGTAPS | 0 | 0 | 0.000922887 |
| 1558 | AQRWAAGLGGTAPSYG | 0 | 0 | 0.003040882 |
| 1559 | DILFTTALPTRIA | 0 | 0.000230555 | 0.002131658 |
| 1560 | SDILFTTALPTRIA | 0 | 5.55429E-05 | 0.005874889 |
| 1561 | SDILFTTALPTRIAY | 0 | 0 | 0.00112569 |
| 1562 | SDILFTTALPTRI | 0 | 0 | 0.000177526 |
| 1563 | VISDILFTTALPTR | 0 | 0 | 0.000203099 |
| 1564 | HPPELLFSASLPALG | 0 | 0.000687186 | 0.014115772 |
| 1565 | HPPELLFSASLPALGFS | 0 | 0.000271424 | 0.013694483 |
| 1566 | HPPELLFSASLPALGFSTY | 0 | 0 | 0.002024503 |
| 1567 | RYPTSIASL | 0 | 0 | 0.003302971 |
| 1568 | RYPTSIASLAF | 0.001084395 | 0 | 0.001486474 |
| 1569 | RPWSFSASLTPVALG | 0 | 0 | 0.001682272 |
| 1570 | SEEEYSAPLPALAPS | 0 | 0 | 0.004944247 |
| 1571 | DVPYTAALTAVRPS | 0.000150348 | 0 | 0.002317368 |
| 1572 | AVRGGHVEIVR | 0 | 0 | 0.000858273 |
| 1573 | IGAVRGGHVEIVR | 5.23262E-05 | 0 | 0.011784197 |
| 1574 | LIGAVRGGHVEIVR | 0 | 0 | 0.003716633 |
| 1575 | VLIGAVRGGHVEIVR | 2.98867E-05 | 0 | 0.002372388 |
| 1576 | IGAVRGGHVE | 0 | 0 | 0.000303332 |
| 1577 | TAALTAVRPSRVA | 0 | 0 | 0.000243906 |
| 1578 | VDTAAMAGLAFTCLKR | 0 | 0.000207741 | 0.010968951 |
| 1579 | GIGLVKGGVSAVAGGVT | 0 | 0 | 0.001243022 |
| 1580 | IGLVKGGVSAVAGGVT | 0 | 0 | 0.004039655 |
| 1581 | DRTKTPIIATLASGAVA | 0 | 4.6987E-05 | 0.002278252 |
| 1582 | KTPIIATLASGAVA | 0.000571838 | 0.000990188 | 0.002156803 |
| 1583 | TPIIATLASGAVA | 0 | 0.002621272 | 0.004698811 |
| 1584 | IATLASGAVAAVM | 6.05543E-05 | 0 | 0.001407211 |
| 1585 | IATLASGAVAAVMA | 0 | 0 | 0.002506663 |
| 1586 | TPIIATLASGAVAAVMA | 0 | 0 | 0.014416362 |
| 1587 | TPIIATLASGAVAAVMAF | 0 | 0 | 0.00575873 |
| 1588 | TPIIATLASGAVAA | 0 | 0.00025658 | 0.002637308 |
| 1589 | TPIIATLASGAVAAV | 0 | 0.000232412 | 0.009271352 |
| 1590 | TPIIATLASGAVAAVM | 9.67027E-05 | 0 | 0.01377938 |
| 1591 | KPVPAAPVPSPVAPAPVP | 0 | 0 | 0.001734719 |
| 1592 | LPSLPGGPLSASL | 0 | 0 | 0.001495813 |
| 1593 | EKALAAGGVGSIVR | 0.000175025 | 0 | 0.000731704 |
| 1594 | EVEHALSSGSIA | 0 | 0 | 4.70923E-05 |
| 1595 | EVEHALSSGSIAAITVT | 0 | 0 | 0.004507047 |
| 1596 | HALSSGSIAAITVT | 0 | 0.000155434 | 0.005423277 |
| 1597 | VEHALSSGSIAAITVT | 0 | 0.000257776 | 0.008172309 |
| 1598 | EVVVAGSSLPTSSK | 0 | 0 | 0.002243833 |
| 1599 | QEVVVAGSSLPTSSK | 0 | 0 | 0.00032282 |
| 1600 | SASIGAAGVPQAG | 0 | 0 | 0.000420452 |
| 1601 | SPGLASGSVGQSP | 0 | 0 | 0 |
| 1602 | TSYVTGSTMPPTT | 0 | 0 | 0.002109261 |
| 1603 | GPLHSSLAASALSQ | 0 | 0 | 0.002722211 |
| 1604 | VGPLHSSLAASALSQ | 0 | 0 | 0.018181846 |
| 1605 | VGPLHSSLAASALSQM | 0 | 0 | 0.000528867 |
| 1606 | VGPLHSSLAASALS | 0 | 0 | 0.004122931 |
| 1607 | VSGVLAGAGLGAALRG | 0 | 0 | 0.00372165 |
| 1608 | GPVPTAIIGGVAGSIL | 0 | 0 | 0.014126844 |
| 1609 | GRRAGPVPTAIIGGVAGSIL | 0 | 0.000184929 | 0.002549324 |
| 1610 | HGSSIAGGLVK | 0 | 0 | 0.000837042 |
| 1611 | HGSSIAGGLVKGALS | 0 | 0 | 0.000469614 |
| 1612 | LPTSFGSLAPAPLVFPGP | 0 | 0.00055926 | 0 |
| 1613 | SPTLPTSFGSLAPAPLVFPGP | 0 | 0.000629377 | 0 |
| 1614 | FPRNISAGSLGSLL | 0 | 0 | 0.00080899 |
| 1615 | LASVSTVLTSKY | 0 | 0.003077917 | 0 |
| 1616 | LASVSTVLTSKYR | 0.000130726 | 0.000747797 | 0 |
| 1617 | LDKFLASVSTVLTS | 0 | 0.001114708 | 0 |
| 1618 | LDKFLASVSTVLTSKY | 0 | 0.000287669 | 0 |
| 1619 | VIVVGAGVAGLVAAK | 0 | 0 | 0.001579278 |
| 1620 | VDLSKYITTIAGVMT | 0 | 0.00139312 | 0 |
| 1621 | STRIIYGGSVTG | 0 | 1.40186E-05 | 0.000634142 |
| 1622 | TRIIYGGSVTGAT | 0 | 0 | 0.000699499 |
| 1623 | AAAISKTAVAPIER | 0 | 0.000535071 | 0 |
| 1624 | AAISKTAVAPIER | 0 | 0.0001306 | 0 |
| 1625 | AAISKTAVAPIERVK | 0 | 7.91725E-05 | 0.000108609 |
| 1626 | AGGIAAAISKTAVAPI | 0 | 0.000125546 | 0.001170277 |
| 1627 | YNYEGRGSVAGSVG | 0 | 0 | 0.001360325 |
| 1628 | AKDFLAGGIAAAISK | 0 | 0 | 0.002054091 |
| 1629 | AKDFLAGGIAAAISKT | 0.00213425 | 0 | 0.001496048 |
| 1630 | AKDFLAGGVAAAISKT | 0 | 0 | 0.002130499 |
| 1631 | KDFLAGGVAAAISK | 0 | 0 | 0.00063507 |
| 1632 | IAGVASALAMALIG | 0 | 0 | 0.000132512 |
| 1633 | DPSSGLGVTKQD | 0 | 0.002570861 | 0 |
| 1634 | DPSSGLGVTKQDLGPVPM | 0.006170414 | 1.934305804 | 0.001060798 |
| 1635 | EDPSSGLGVTKQDLGPVPM | 0.001005489 | 0.679507417 | 0.000152534 |
| 1636 | LEDPSSGLGVTKQDLGPVPM | 0.000829241 | 0.469124485 | 0 |
| 1637 | LELEDPSSGLGVTKQDLGPVPM | 0 | 0.037918672 | 0 |
| 1638 | PSSGLGVTKQ | 0.000100514 | 0.000530716 | 0 |
| 1639 | PSSGLGVTKQDLGPVP | 0.001670138 | 0.006911203 | 0.000440338 |
| 1640 | GLGVTKQDLGPVPM | 0.000323094 | 0.640657858 | 0 |
| 1641 | GVTKQDLGPVPM | 0.000365348 | 0.811091437 | 0 |
| 1642 | LGVTKQDLGPVPM | 0.000253651 | 0.490634068 | 0.000108001 |
| 1643 | PSSGLGVTKQDLGPVPM | 0.05566419 | 0.229070013 | 0.019490556 |
| 1644 | SGLGVTKQDLGPVPM | 0 | 0.10070943 | 0 |
| 1645 | SSGLGVTKQDLGPVPM | 0 | 0.096745409 | 0.000981211 |
| 1646 | VTKQDLGPVPM | 0 | 0.012144045 | 0 |
| 1647 | DSKDSTYSLSSTLTLSKA | 0 | 0.000642478 | 0.000918692 |
| 1648 | DSTYSLSSTLTLSK | 0 | 0.002502656 | 0.002811601 |
| 1649 | DSTYSLSSTLTLSKA | 0 | 0.000249231 | 0.003273688 |
| 1650 | KDSTYSLSSTLTLSKA | 0 | 0.006101034 | 0.009188177 |
| 1651 | KDSTYSLSSTLTLSK | 0 | 0.003757708 | 0.00259517 |
| 1652 | KDSTYSLSSTLTLSKAD | 0 | 0 | 0.010544181 |
| 1653 | KDSTYSLSSTLTLSKADY | 0 | 0 | 0.002085146 |
| 1654 | FPDQAYANSQPAAS | 0 | 0 | 0.015225667 |
| 1655 | NPFSETNAATTVPVT | 0 | 0 | 0.003505672 |
| 1656 | NPFSETNAATTVPVTQ | 0 | 0 | 0.018360604 |
| 1657 | NPFSETNAATTVPVTQLPG | 0 | 0 | 0.00177503 |
| 1658 | SETNAATTVPVTQ | 3.85E-05 | 0.000509599 | 0.000563745 |
| 1659 | GPGDVETGTSITVPQK | 0 | 0.003729714 | 0 |
| 1660 | GPGDVETGTSITVPQKK | 0.000447285 | 0.002178673 | 0 |
| 1661 | NPEKVNIEGGAIA | 0 | 0 | 0.006700636 |
| 1662 | NPEKVNIEGGAIALGHP | 0 | 0.000517508 | 0.003064276 |
| 1663 | NPEKVNIEGGAIALGHPLG | 0 | 0 | 0.00072263 |
| 1664 | VAVGRALYY | 0.000148377 | 0.000749624 | 0.000445817 |
| 1665 | VNVFIVSVAKPIP | 0 | 0.001141925 | 0 |
| 1666 | FPKPPSYNVATTLPSYD | 0 | 0.00029402 | 0.005286034 |
| 1667 | FPKPPSYNVATTLPSYDE | 0 | 0 | 0.005214263 |
| 1668 | FPKPPSYNVATTLPSYDEA | 0 | 0 | 0.048437933 |
| 1669 | KPPSYNVATTLPSYDEA | 0.000181572 | 0 | 0.00316941 |
| 1670 | TLPSYDEAERTK | 0.000479216 | 0 | 0 |
| 1671 | TSLPTYDEAEKAK | 0.001280518 | 0.000611532 | 5.87847E-05 |
| 1672 | YPVPPPYSVATSLPTYD | 0 | 0.000124769 | 0.00081607 |
| 1673 | YPVPPPYSVATSLPTYDEA | 0 | 0 | 0.026486194 |
| 1674 | DWRWLDGSPVTLS | 0 | 0 | 0.000638991 |
| 1675 | KFIDTTSKF | 5.03924E-05 | 0 | 0.006097492 |
| 1676 | SPMPEHYVEGSTVN | 0 | 1.39522E-05 | 0.001751123 |
| 1677 | ASEDRLGGAIAAIN | 0 | 0 | 0.005535319 |
| 1678 | EDRLGGAIAAINSIQ | 3.28933E-05 | 0 | 0.001627154 |
| 1679 | GIASIESIHSE | 0 | 0.000218123 | 0 |
| 1680 | IASIESIHSE | 0 | 0.000114059 | 0.00059027 |
| 1681 | DAGTIAGLNVL | 0.004085467 | 0 | 1.23857E-05 |
| 1682 | DAGTIAGLNVLRII | 8.34954E-05 | 0.000446336 | 4.21677E-05 |
| 1683 | DAGTIAGLNVLRIIN | 0.001767007 | 0 | 0 |
| 1684 | DAGTIAGLNVLRIINEP | 0.00062781 | 0 | 0 |
| 1685 | KDAGTIAGLNVL | 0 | 0 | 0 |
| 1686 | DPTEATAVGAVEASFK | 0.00177508 | 0 | 0.00018208 |
| 1687 | EAGSPLAGLDMDTFDSG | 0 | 0 | 0.002961938 |
| 1688 | DSKLHSISSIDVN | 0 | 0.001731751 | 0.001218342 |
| 1689 | KLHSISSIDVN | 0 | 0.00026983 | 0 |
| 1690 | LHSISSIDVN | 0 | 0.001069705 | 4.52109E-05 |
| 1691 | SKLHSISSIDVN | 0 | 0.00048474 | 0.000298192 |
| 1692 | VDSKLHSISSIDVN | 5.44199E-05 | 0.015210783 | 0.001313805 |
| 1693 | WVDSKLHSISSIDVN | 0 | 0.004057804 | 0 |
| 1694 | GQGHVHGVASSPSHD | 0 | 0 | 0.000404375 |
| 1695 | GQGHVHGVASSPSHDLA | 0 | 0 | 0.002100274 |
| 1696 | LNVLRIINEPTAAA | 0 | 0.002360211 | 0.001994595 |
| 1697 | AEKAKAAAMAAAAAE | 0 | 1.53182E-05 | 0.001718264 |
| 1698 | APDFSKLARAAAVSSGFD | 0 | 0 | 0.004886868 |
| 1699 | APDFSKLARAAAVSSGFDG | 0 | 0 | 0.00848705 |
| 1700 | APDFSKLARAAAVSSGFDGA | 0 | 0 | 0.002095228 |
| 1701 | FSKLARAAAVSSGFDG | 0 | 0 | 0.003519359 |
| 1702 | APEPSTVQILHSPA | 0 | 0.020343266 | 0.000141009 |
| 1703 | APEPSTVQILHSPAVE | 0.001430454 | 0.001931668 | 0 |
| 1704 | APEPSTVQILHSPAVEGSQ | 0 | 0 | 0.004476133 |
| 1705 | APEPSTVQILHSPAVEGSQV | 0 | 0 | 0.002041632 |
| 1706 | APEPSTVQILHSPAVEGSQVE | 0 | 0 | 0.003355712 |
| 1707 | YAPEPSTVQILHSP | 0 | 0.011480833 | 0 |
| 1708 | YAPEPSTVQILHSPA | 0 | 0.006992237 | 0 |
| 1709 | DLGPEGGTVELIQ | 0 | 0 | 0.002350128 |
| 1710 | DLGPEGGTVELIQGR | 0 | 0.00036283 | 0.009861543 |
| 1711 | GPEGGTVELIQ | 0 | 6.57829E-05 | 0.006156505 |
| 1712 | LDLGPEGGTVELIQGR | 0.000317766 | 0 | 0.016471136 |
| 1713 | LDLGPEGGTVELIQGRA | 0.001232342 | 0 | 0.041535378 |
| 1714 | LGPEGGTVE | 0 | 0 | 0.001664078 |
| 1715 | LGPEGGTVELIQGR | 0 | 0 | 0.002722778 |
| 1716 | LGPEGGTVELIQGRA | 0 | 0 | 0.003394673 |
| 1717 | GPEGGTVELIQG | 0 | 0 | 0.002135553 |
| 1718 | GPEGGTVELIQGR | 0 | 0 | 0.013632521 |
| 1719 | GPEGGTVELIQGRA | 0 | 0 | 0.027118255 |
| 1720 | GPEGGTVELIQGRAAKGN | 0 | 0 | 0.003186474 |
| 1721 | FPDQFSTGEPPALD | 7.07084E-05 | 0.003784534 | 0 |
| 1722 | FPDQFSTGEPPALDEVP | 0 | 0.002212645 | 0 |
| 1723 | FPDQFSTGEPPALDEVPE | 0 | 0.003386468 | 0 |
| 1724 | LSLNEEEIR | 0 | 0.001495738 | 0 |
| 1725 | LSLNEEEIRAN | 0.000716206 | 0.00058262 | 0.000186006 |
| 1726 | RPSSSPNPTTIQNENLK | 0 | 0.002937967 | 0 |
| 1727 | SPNPTTIQNENLK | 0 | 0.004480781 | 0 |
| 1728 | SSPNPTTIQNENLK | 0 | 0.003668548 | 0.000969354 |
| 1729 | EDEDEDVKAERLK | 0 | 0.000323377 | 7.90572E-06 |
| 1730 | PQGPSLEWLKKL | 0.003637154 | 0.002776208 | 0.002799883 |
| 1731 | DKTGTITHGTPVVN | 5.82236E-05 | 3.04942E-05 | 0.0076283 |
| 1732 | KTGTITHGTPVVN | 0 | 1.61338E-05 | 0.000590446 |
| 1733 | TGTITHGTPVVN | 0 | 0 | 0.00295915 |
| 1734 | MPATLARAAPGEAPP | 0 | 0 | 0.000989776 |
| 1735 | GPIVVHCSAGVGRTGTYIG | 4.69381E-05 | 0 | 0.00268099 |
| 1736 | EPTDTAAMARTSN | 0 | 0 | 0.00059613 |
| 1737 | AAEAKAAEAAASAYY | 0 | 0 | 0.000785662 |
| 1738 | GPDVPSTPAAEAKAAEAAASAYYN | 0 | 0 | 0.01855668 |
| 1739 | TPAAEAKAAEAAASAYY | 0 | 0.000263336 | 0.11277904 |
| 1740 | TPAAEAKAAEAAASAYYN | 0 | 0.002486559 | 0.097280718 |
| 1741 | VPSTPAAEAKAAEAAASAYYN | 0 | 0 | 0.080310029 |
| 1742 | VPSTPAAEAKAAEAAA | 0 | 0 | 0.005310837 |
| 1743 | VPSTPAAEAKAAEAAAS | 0 | 0 | 0.053773515 |
| 1744 | VPSTPAAEAKAAEAAASA | 0 | 7.79242E-05 | 0.009705043 |
| 1745 | VPSTPAAEAKAAEAAASAYY | 0 | 0.006378226 | 0.021552069 |
| 1746 | VPSTPAAEAKAAEAAASAYYNP | 0 | 0 | 0.022147922 |
| 1747 | TPAAEAKAAEAAAS | 0.000388639 | 0.000139347 | 0.009378026 |
| 1748 | TPAAEAKAAEAAASA | 0 | 0 | 0.00510543 |
| 1749 | TPAAEAKAAEAAASAYYNP | 0 | 0 | 0.023884128 |
| 1750 | TPAAEAKAAEAAASAYYNPG | 0 | 0 | 0.010335697 |
| 1751 | VPSTPAAEAKAAEAAASAYYNPG | 0 | 0.000157692 | 0.008417574 |
| 1752 | GPDVPSTPAAEAKAAEAA | 0 | 5.36304E-05 | 0.019268323 |
| 1753 | GPDVPSTPAAEAKAAEAAA | 6.17983E-05 | 0 | 0.004288452 |
| 1754 | GPDVPSTPAAEAKAAEAAASA | 0 | 0 | 0.007358605 |
| 1755 | TPAAEAKAAEAA | 0 | 0 | 0.001721687 |
| 1756 | VPSTPAAEAKAAEAA | 0 | 0 | 0.010744571 |
| 1757 | AKAAEAAASAYYN | 0 | 0 | 0.001140518 |
| 1758 | AKAAEAAASAYYNPG | 0 | 8.34474E-05 | 0.005257027 |
| 1759 | AKAAEAAASAYYNPGN | 0 | 0 | 0.009012116 |
| 1760 | AKAAEAAASAYYNPGNPH | 0 | 0 | 0.036963218 |
| 1761 | EAKAAEAAASAYYNPGNPH | 0 | 0 | 0.003829594 |
| 1762 | TPAAEAKAAEAAASAYYNPGNPH | 0 | 0 | 0.006233863 |
| 1763 | KAAEAAASAYYNPG | 0 | 0 | 0.000953322 |
| 1764 | KAAEAAASAYYNPGN | 0 | 0 | 0.007975812 |
| 1765 | KAAEAAASAYYNPGNP | 0.000334719 | 0 | 0.001883919 |
| 1766 | KAAEAAASAYYNPGNPH | 0 | 0.000102068 | 0.011078664 |
| 1767 | EPQAKGAEASAASEE | 7.14815E-05 | 3.30193E-05 | 0.011836904 |
| 1768 | EPQAKGAEASAASEEE | 0 | 0 | 0.004873274 |
| 1769 | QEPQAKGAEASAASEEE | 0 | 0 | 0.00465402 |
| 1770 | QEPQAKGAEASAASEE | 0 | 0 | 0.005008077 |
| 1771 | TPESQEPQAKGAEASAASEE | 0 | 0 | 0.001350251 |
| 1772 | VPQPRGSEAPSA | 0.000142326 | 0 | 0.002735214 |
| 1773 | AKGAEASAASEEEAGPQ | 0.000450751 | 0.001371725 | 0.000469477 |
| 1774 | ASAYYNPGNPHNV | 0 | 0 | 0.008805568 |
| 1775 | ASAYYNPGNPHNVY | 0 | 0 | 0.022990602 |
| 1776 | ASAYYNPGNPHNVYMPT | 0 | 0 | 0.003763822 |
| 1777 | SAYYNPGNPHNV | 0 | 0 | 0.001347924 |
| 1778 | RYFDPANGKF | 0 | 0.000140439 | 0.002800653 |
| 1779 | ATTAANPRESSYA | 0 | 0 | 0.000779116 |
| 1780 | DLPPASSEARNSAFG | 0 | 0 | 0.002157397 |
| 1781 | LPPASSEARNSAFG | 0 | 0.000565015 | 0.024342045 |
| 1782 | TTAANPRESSYA | 6.58446E-05 | 0 | 0.000785712 |
| 1783 | VDLPPASSEARNSAFG | 0 | 0.000962277 | 0.009644956 |
| 1784 | DLPPASSEARNSA | 0 | 0 | 0.000988611 |
| 1785 | LPPASSEARNSA | 6.06075E-06 | 0 | 0.012490646 |
| 1786 | VDLPPASSEARNSA | 0 | 1.22783E-05 | 0.000644324 |
| 1787 | INVYATTAANPRES | 3.02164E-05 | 0.006127413 | 0.000743396 |
| 1788 | INVYATTAANPRESS | 0 | 0.002864974 | 0.001256307 |
| 1789 | INVYATTAANPRESSY | 0 | 7.30158E-05 | 0.002036323 |
| 1790 | INVYATTAANPRESSYA | 0 | 0.002341757 | 0.004613685 |
| 1791 | LPDNINVYATTAANPRESSYA | 0 | 5.09013E-05 | 0.007617551 |
| 1792 | VYATTAANPRES | 0 | 0 | 0.002491094 |
| 1793 | NVYATTAANPRESS | 0 | 0.000189761 | 0.002131943 |
| 1794 | NVYATTAANPRESSY | 0 | 0 | 0.001270712 |
| 1795 | NVYATTAANPRESSYA | 0 | 0 | 0.00733706 |
| 1796 | VYATTAANPRESS | 0 | 0 | 0.022406238 |
| 1797 | VYATTAANPRESSY | 0 | 2.15306E-05 | 0.01787953 |
| 1798 | VYATTAANPRESSYA | 0 | 0 | 0.162377414 |
| 1799 | LPDNINVYATTAANPRESS | 0 | 0 | 0.00094131 |
| 1800 | LPDNINVYATTAANPRESSY | 0 | 0 | 0.000999321 |
| 1801 | NINVYATTAANPRES | 0 | 0.000265821 | 0.000403025 |
| 1802 | AARSTDVAAVVVPILF | 0.000342176 | 0.001057003 | 0 |
| 1803 | AARSTDVAAVVVPILFL | 0 | 0.000297603 | 0 |
| 1804 | ARSTDVAAVVVPIL | 0.001481337 | 0.000138279 | 0 |
| 1805 | ARSTDVAAVVVPILF | 0.003492436 | 0.004363601 | 0 |
| 1806 | RSTDVAAVVVP | 0.001480695 | 0 | 0 |
| 1807 | RSTDVAAVVVPIL | 0.005741948 | 0.003561508 | 0 |
| 1808 | ARSTDVAAVVVPILFL | 0.0001145 | 0.001380955 | 0 |
| 1809 | RSTDVAAVVVPILF | 0.005703922 | 0.005370328 | 0 |
| 1810 | RSTDVAAVVVPILFL | 0 | 0.001569998 | 0 |
| 1811 | STDVAAVVVPILF | 0.001241969 | 0.001791066 | 0 |
| 1812 | DASATQAARSTDVA | 0 | 0 | 0.000534463 |
| 1813 | GADASATQAARSTDVA | 0 | 8.49938E-05 | 0.008402305 |
| 1814 | SGADASATQAARSTDVA | 1.92049E-05 | 0 | 0.001565718 |
| 1815 | SGADASATQAARSTDVAA | 0 | 0 | 0.001407843 |
| 1816 | SGADASATQAARSTDVAAVVVPILF | 0.000849894 | 0.000703558 | 0.000102475 |
| 1817 | GADASATQAARSTD | 0 | 0 | 0.000448728 |
| 1818 | GADASATQAARSTDV | 0 | 0 | 0.000528407 |
| 1819 | DSIASVVVPIII | 0 | 0 | 0 |
| 1820 | LDSIASVVVPIII | 0 | 0.000442591 | 0 |
| 1821 | SIASVVVPIII | 0 | 0.000209931 | 0 |
| 1822 | AASSDSAQGSDVSLTA | 0 | 0 | 0.001277766 |
| 1823 | ASSDSAQGSDVSLTA | 0 | 0.000145323 | 0.003236067 |
| 1824 | DSAQGSDVSLTA | 0 | 0 | 0.031102477 |
| 1825 | SDSAQGSDVSLTA | 2.39962E-05 | 0 | 0.018773962 |
| 1826 | SSDSAQGSDVSLTA | 0 | 0 | 0.041293608 |
| 1827 | SSDSAQGSDVSLT | 0 | 0 | 0.003103696 |
| 1828 | APVEPATADATEIHDA | 0 | 0.000565566 | 0 |
| 1829 | APVEPATADATEIHDAA | 0 | 0.000882694 | 0 |
| 1830 | IPTGAEASNVL | 0 | 0 | 0.002255039 |
| 1831 | IPTGAEASNVLVG | 0 | 0 | 0.019655111 |
| 1832 | TQQLPAAAQAPDALPA | 0 | 0 | 0.003629232 |
| 1833 | LPVVINGGATSSGEQ | 0.000139584 | 0 | 0.005669864 |
| 1834 | LPVVINGGATSSGEQD | 0 | 0.000633077 | 0.010574932 |
| 1835 | LPVVINGGATSSGEQDN | 0 | 0 | 0.012838449 |
| 1836 | TPIVIQPASATETT | 8.03317E-05 | 0.000936142 | 0.005239479 |
| 1837 | TPIVIQPASATETTQ | 0 | 0 | 0.005332283 |
| 1838 | TPIVIQPASATETTQL | 0 | 0 | 0.00265154 |
| 1839 | AEAEAAAAAAEPGFDDEE | 0 | 0 | 0.000724937 |
| 1840 | FHRAASSAAQGAFQ | 0 | 2.54305E-05 | 0.006260045 |
| 1841 | FHRAASSAAQGAFQG | 0 | 0 | 0.000173023 |
| 1842 | FHRAASSAAQGAFQGN | 0 | 0 | 0.000408688 |
| 1843 | TFHRAASSAAQGAFQGN | 0 | 6.16299E-06 | 0.001731794 |
| 1844 | TFHRAASSAAQGAFQ | 0 | 0 | 0.012830836 |
| 1845 | TFHRAASSAAQGAFQG | 1.2864E-05 | 0 | 0.003217445 |
| 1846 | RTFHRAASSAAQGAF | 0 | 0 | 0.000421579 |
| 1847 | RTFHRAASSAAQGAFQ | 0 | 0 | 0.002591466 |
| 1848 | SSRTFHRAASSAAQGAF | 0 | 0.000215699 | 0.004169865 |
| 1849 | YPGDVDRSSSAGGQGSYVP | 0 | 0 | 0.001089512 |
| 1850 | APASDSKPGSSEAAPSS | 0 | 0 | 0.000486393 |
| 1851 | APASDSKPGSSEAAPSSK | 0 | 0 | 0.00138727 |
| 1852 | APASDSKPGSSEAAPSSKE | 0 | 0 | 0.001304454 |
| 1853 | APASDSKPGSSEAAPSSKETPA | 0 | 0 | 0.001144129 |
| 1854 | GPASDTASPEGHA | 0 | 0.000372058 | 0 |
| 1855 | APSATQPATAETQHIA | 0 | 0.000687805 | 0 |
| 1856 | APSATQPATAETQHIAD | 0 | 0.001132908 | 0.000201065 |
| 1857 | APSATQPATAETQHIADQ | 0 | 0.005643895 | 0.001517762 |
| 1858 | LPGGAEGAAAQPQAVLR | 0 | 0 | 0.003355834 |
| 1859 | APPVPPVKPGAGAAN | 0 | 0 | 0.018008329 |
| 1860 | APPVPPVKPGAGAANPG | 0 | 0 | 0.000679489 |
| 1861 | APPVPPVKPGAGAANPGP | 0 | 0 | 0.075069234 |
| 1862 | APPVPPVKPGAGAANPGPA | 0 | 0 | 0.052565139 |
| 1863 | APPVPPVKPGAGAANPGPAE | 0 | 1.69636E-05 | 0.317027545 |
| 1864 | APPVPPVKPGAGAANPGPAEG | 0 | 0 | 0.003659248 |
| 1865 | PPVPPVKPGAGAANPGPAE | 0 | 0 | 0.003429199 |
| 1866 | NPAVRTAAANAAAGA | 0 | 0 | 0.003055381 |
| 1867 | NPAVRTAAANAAAGAA | 0 | 0 | 0.002116016 |
| 1868 | NPAVRTAAANAAAGAAE | 0 | 0 | 0.00645489 |
| 1869 | SNPAVRTAAANAAAGA | 0 | 0 | 0.000895442 |
| 1870 | SNPAVRTAAANAAAGAA | 0 | 0 | 0.001204268 |
| 1871 | SEAPSGESRKF | 0 | 0.001653153 | 0 |
| 1872 | IEQPPTSNPAPQIVQ | 0 | 0.00929427 | 0 |
| 1873 | VPESTSTDTPPDID | 0 | 0.000811534 | 0 |
| 1874 | VPESTSTDTPPDIDLH | 0 | 0.001194459 | 0.000570488 |
| 1875 | VPESTSTDTPPDIDLHN | 0 | 0.000485648 | 0 |
| 1876 | LPESKGGSSEPGTELQ | 0 | 0 | 0.001414205 |
| 1877 | APGVEASGAGEAR | 4.2935E-06 | 0 | 0.001556128 |
| 1878 | APGVEASGAGEARA | 0 | 0 | 0.000232308 |
| 1879 | APGVEASGAGEARAS | 0 | 0 | 0.000616744 |
| 1880 | APGVEASGAGEARAST | 0 | 0 | 0.000355405 |
| 1881 | APGVEASGAGEARASTG | 0 | 0 | 0.000854462 |
| 1882 | APGVEASGAGEARASTGS | 0 | 0 | 0.000224792 |
| 1883 | APGVEASGAGEARASTGSS | 0 | 0 | 0.003162526 |
| 1884 | VEASGAGEARASTGS | 0 | 0 | 0.000489611 |
| 1885 | VEASGAGEARASTGSS | 0 | 0 | 0.000678985 |
| 1886 | AQPGEGHAAPTN | 0 | 0 | 0.003138531 |
| 1887 | AQPGEGHAAPTNVVE | 0 | 0 | 0.001289817 |
| 1888 | EPAAQPGEGHAAPTNVVE | 0 | 0 | 0.009646203 |
| 1889 | EPAAQPGEGHAAPTN | 0 | 0 | 0.020705567 |
| 1890 | EPAAQPGEGHAAPTNVV | 0 | 0 | 0.009887376 |
| 1891 | GPPGNWSPRPPRAGEARPGP | 0 | 0 | 0.001524063 |
| 1892 | PRPPRAGEARPGP | 0 | 0 | 0.018147342 |
| 1893 | RPPRAGEARPGP | 0 | 0 | 0.023632084 |
| 1894 | SPRPPRAGEARPGP | 0 | 0 | 0.228300009 |
| 1895 | WSPRPPRAGEARPGP | 0 | 9.02082E-05 | 0.000579105 |
| 1896 | RPPRAGEARPGPT | 0 | 0 | 0.002372575 |
| 1897 | RPPRAGEARPGPTA | 0 | 0 | 0.003207568 |
| 1898 | SPRPPRAGEARPGPTA | 0 | 0 | 0.081593431 |
| 1899 | SPRPPRAGEARPGPTAE | 0 | 0 | 0.005158798 |
| 1900 | SPRPPRAGEARPGPTAES | 0 | 0 | 0.004460552 |
| 1901 | SPRPPRAGEARPG | 0 | 0 | 0.000327695 |
| 1902 | SPRPPRAGEARPGPT | 0 | 0 | 0.033729031 |
| 1903 | QPRGKPGESRSASSDTIE | 0 | 0.002298031 | 0 |
| 1904 | RVIQPHGGGSNPLE | 0 | 0 | 5.43071E-05 |
| 1905 | VIQPHGGGSNPLE | 0 | 0 | 0.00304997 |
| 1906 | GDAIEPAPPSQGAEAK | 0 | 0 | 0.001595145 |
| 1907 | TGDAIEPAPPSQGAEAK | 0 | 0 | 0.004226724 |
| 1908 | TGDAIEPAPPSQGAEAKG | 0 | 0 | 0.000477908 |
| 1909 | QPSPDAAASQTQTSPAHP | 0 | 0 | 0.005885374 |
| 1910 | QPSPDAAASQTQTSPAHPS | 0 | 0 | 0.003179236 |
| 1911 | SPAEPEGGPASEGAARP | 8.66499E-06 | 0 | 0.00348005 |
| 1912 | DRKGGSYSQAASSDSAQG | 0 | 0 | 0.000525753 |
| 1913 | KGGSYSQAASSDSAQG | 0 | 0 | 0.000528334 |
| 1914 | DRQMDTEAAASEAPQ | 0 | 0.000502544 | 0 |
| 1915 | EDRQMDTEAAASEAPQ | 0 | 0.005090202 | 0.000685607 |
| 1916 | EEDRQMDTEAAASEAPQ | 0 | 0.001759859 | 0 |
| 1917 | RQMDTEAAASEAPQ | 0 | 0.000161136 | 0 |
| 1918 | EDRQMDTEAAASEAP | 4.03945E-05 | 0.000608556 | 0 |
| 1919 | DTEAAASEAPQDVT | 2.08753E-05 | 0.000704713 | 0 |
| 1920 | AGVATGSEAQSSPQF | 0 | 0 | 0.003223718 |
| 1921 | AGVATGSEAQSSPQFQ | 5.1434E-05 | 0 | 0.003775958 |
| 1922 | EAGVATGSEAQSSPQFQ | 0 | 0 | 0.00203325 |
| 1923 | GVATGSEAQSSPQFQ | 0 | 0 | 0.00252148 |
| 1924 | SSFHPAPGNAQSSSQ | 0 | 0 | 0.000669359 |
| 1925 | SSFHPAPGNAQSSSQTPK | 0 | 0 | 0.001803833 |
| 1926 | RPSLSPTSGDANPAFG | 0 | 0 | 0.005143192 |
| 1927 | DDDSSTASDSDVLIR | 0.000295533 | 0.00256611 | 0 |
| 1928 | DDSSTASDSDVLIR | 0 | 0.001592686 | 0 |
| 1929 | DSSTASDSDVLIR | 0 | 0.000722568 | 0 |
| 1930 | GNRFATASADGQIY | 0 | 0.000855383 | 0 |
| 1931 | SPDGNRFATASADGQIY | 0 | 0.002765167 | 0 |
| 1932 | IPGSTTNPEVFK | 0 | 0.006594183 | 0 |
| 1933 | YIPGSTTNPEVFK | 0 | 0.001154555 | 0.000735049 |
| 1934 | PGSTTNPEVFKL | 0 | 0.004711144 | 0 |
| 1935 | EPPLYPMAGAAGPQGDE | 0 | 0 | 0.002133333 |
| 1936 | GPSTTTETETIAK | 0 | 0.003494118 | 0 |
| 1937 | IGPSTTTETETIAK | 0 | 0.007377325 | 0.000173543 |
| 1938 | ISAGSGGQAERT | 0 | 0 | 7.10973E-05 |
| 1939 | ISAGSGGQAERTIA | 0 | 0 | 0.000942256 |
| 1940 | ISAGSGGQAERTIAG | 0 | 0 | 0.004574255 |
| 1941 | ISAGSGGQAERTIAGT | 0 | 0 | 0.004258449 |
| 1942 | DVKPSPSAAEADFD | 0.009793175 | 5.91622E-05 | 5.88156E-05 |
| 1943 | DVKPSPSAAEADFDIV | 0.012587563 | 0 | 0.001898237 |
| 1944 | DVKPSPSAAEADFDIVT | 0.021509232 | 0 | 0.002045693 |
| 1945 | KPSPSAAEADFD | 0.022018776 | 0 | 0.009284337 |
| 1946 | VDVKPSPSAAEADFDIVT | 0.030360207 | 0 | 0.002339567 |
| 1947 | VDVKPSPSAAEADFDIVTK | 0.005388421 | 0 | 0.003875957 |
| 1948 | VKPSPSAAEADFD | 0.034361182 | 0 | 0.016629087 |
| 1949 | VKPSPSAAEADFDIV | 0.011363065 | 0 | 0.002472739 |
| 1950 | VKPSPSAAEADFDIVT | 0.08410551 | 0.000456798 | 0.018913645 |
| 1951 | KPSPSAAEADFDI | 0.003608745 | 0.001670817 | 0 |
| 1952 | KPSPSAAEADFDIV | 0.039741927 | 0 | 0.004316946 |
| 1953 | KPSPSAAEADFDIVT | 0.057304075 | 0.001778018 | 0.012444604 |
| 1954 | KPSPSAAEADFDIVTK | 0.010906811 | 9.30253E-05 | 0.000368255 |
| 1955 | VKPSPSAAEADFDIVTK | 0.009113879 | 4.24939E-05 | 0.000397984 |
| 1956 | VDVKPSPSAAEADFD | 0.01531945 | 0.000809108 | 0.00343612 |
| 1957 | VDVKPSPSAAEADFDIV | 0.017205127 | 0 | 0.001479991 |
| 1958 | YTATEGQYQ | 8.95969E-05 | 0.001388044 | 0 |
| 1959 | SPGQPEAGPEGAQERPSQ | 0 | 0 | 0.000206564 |
| 1960 | GVWVFEIGSPATTN | 0 | 0 | 0.000959875 |
| 1961 | VWVFEIGSPATTN | 0 | 0 | 0.004271029 |
| 1962 | VWVFEIGSPATTNG | 0 | 6.35458E-05 | 0.002629105 |
| 1963 | VWVFEIGSPATTNGVVPA | 0.000490753 | 0 | 0.002568545 |
| 1964 | IVYDVSGAASDRN | 0 | 6.15702E-05 | 0.000520733 |
| 1965 | HGPTEGVVPGN | 0 | 0 | 0.001101142 |
| 1966 | QPTPQAVVSAAQAGLLR | 0 | 0 | 0.001822341 |
| 1967 | IEKQIATSGPPPEGGL | 0 | 0.00466355 | 9.99794E-05 |
| 1968 | AKRVIISAPSADAP | 0 | 0 | 0.004865166 |
| 1969 | GAKRVIISAPSADAP | 0 | 0 | 0.0025116 |
| 1970 | IISAPSADAPMFVMG | 0.003533411 | 0 | 0 |
| 1971 | KRILITGGAGFVGSH | 0 | 0 | 0.00730154 |
| 1972 | RILITGGAGFVGSH | 0 | 0 | 0.001627999 |
| 1973 | APEASGTPSSDAVSRLEEE | 0 | 0.000488278 | 8.46776E-05 |
| 1974 | ASGTPSSDAVSRLEEE | 0 | 0.00051869 | 0 |
| 1975 | GTPSSDAVSRLEEE | 0 | 0.000780089 | 0 |
| 1976 | TPSSDAVSRLE | 0 | 0.002002031 | 1.8025E-05 |
| 1977 | TPSSDAVSRLEE | 0 | 0.000150236 | 0 |
| 1978 | TPSSDAVSRLEEE | 0 | 0.002854359 | 9.35735E-05 |
| 1979 | NASATTTEPLPEKT | 0 | 0.000416154 | 0 |
| 1980 | HGIDPTGTY | 9.59104E-05 | 0.000100757 | 0.000820048 |
| 1981 | SDLAVEAGAPYAERL | 0.005180671 | 0.000536402 | 0.001354568 |
| 1982 | KPVQPSGPEGVPHYAEADIVN | 0.005681627 | 0.0033626 | 0.009581759 |
| 1983 | KPVQPSGPEGVPHYAEADIVNLQG | 0 | 0 | 0.002219132 |
| 1984 | RPASPATETVPAFSE | 0 | 0.000569349 | 0.000164752 |
| 1985 | INIIMDGPPAQSLG | 3.46121E-05 | 0 | 0.014127703 |
| 1986 | WINIIMDGPPAQSLG | 0 | 0 | 0.00345905 |
| 1987 | EDSAATSESLDVM | 0 | 0.002129904 | 0 |
| 1988 | EDSAATSESLDVMASQKRP | 0 | 0.000713395 | 0 |
| 1989 | EQEEAAAAAAEPGPDPS | 0.000527491 | 0.000145792 | 0.001678627 |
| 1990 | QEEAAAAAAEPGPDPS | 3.30053E-05 | 0 | 0.000881083 |
| 1991 | SSSATAQGTPEKKEEPD | 0.000262579 | 0.000764765 | 0.000608902 |
| 1992 | GPAEGSGEAARVNGR | 0 | 0 | 0.000308474 |
| 1993 | DPEEEKGSQPPPAAES | 0 | 0 | 0.000606227 |
| 1994 | DPEEEKGSQPPPAAESQ | 0 | 0 | 0.0002057 |
| 1995 | IPQEEHAANGPELL | 0.003166734 | 0 | 0 |
| 1996 | LLPWSRGEPGAAEPS | 0 | 0 | 0.002308922 |
| 1997 | LPWSRGEPGAAEP | 0 | 0 | 0.009017608 |
| 1998 | LPWSRGEPGAAEPS | 0 | 0 | 0.012680701 |
| 1999 | AAEEKAAQALSVQ | 0 | 0 | 0.003506996 |
| 2000 | AAEEKAAQALSVQDKG | 0 | 0.000111858 | 0.002998414 |
| 2001 | AEEKAAQALSVQ | 0 | 0 | 0 |
| 2002 | AEEKAAQALSVQDKG | 0 | 0 | 0.000376733 |
| 2003 | DRPGQGIAVPVG | 0 | 0 | 0.002888538 |
| 2004 | LDRPGQGIAVPVG | 0 | 0 | 0.012884846 |
| 2005 | LDRPGQGIAVPVGE | 0 | 0 | 0.004257453 |
| 2006 | LDRPGQGIAVPVGEA | 0 | 0 | 0.008353415 |
| 2007 | QLDRPGQGIAVPVG | 0 | 0 | 0.000594906 |
| 2008 | FPLPIGESVTVTRDLEG | 0 | 0.000223168 | 0 |
| 2009 | SEALALTQTW | 8.38636E-05 | 0.002765444 | 0 |
| 2010 | DLGSPEGIAVDHLGRN | 0.002649784 | 6.01438E-05 | 0 |
| 2011 | SPEGIAVDHLG | 0.003606104 | 3.06619E-05 | 0 |
| 2012 | SPEGIAVDHLGRN | 0.009240046 | 0 | 0 |
| 2013 | DGPPAQSLGVEPVDK | 0 | 0.000652151 | 0.002755757 |
| 2014 | ADEPSSEESDLEIDKE | 0 | 0.001100182 | 0 |
| 2015 | DEPSSEESDLEIDK | 3.74783E-05 | 0.002820084 | 0 |
| 2016 | DEPSSEESDLEIDKE | 0 | 0.002718752 | 0.00037677 |
| 2017 | EPSSEESDLEIDK | 0 | 0.001086754 | 0 |
| 2018 | KADEPSSEESDLEIDKE | 1.38648E-05 | 0.001287735 | 0.000126206 |
| 2019 | EPSSEESDLEIDKE | 0 | 0.001493191 | 0 |
| 2020 | KADEPSSEESDLEIDK | 0 | 0.000359863 | 0 |
| 2021 | IQKYGTNPEETAAE | 0 | 0.003959802 | 0 |
| 2022 | IQKYGTNPEETAAEE | 0 | 0.001901336 | 0 |
| 2023 | KTIQKYGTNPEETAAEE | 0 | 0.00077089 | 0 |
| 2024 | TIQKYGTNPEETAAEE | 0 | 0.00438255 | 0 |
| 2025 | TIQKYGTNPEETA | 0 | 0.002457815 | 5.16234E-05 |
| 2026 | TIQKYGTNPEETAAE | 0 | 0.002993984 | 0 |
| 2027 | KYGTNPEETAAEESWD | 0 | 0.002099838 | 0 |
| 2028 | NPEETAAEESWD | 0 | 0.01341826 | 0 |
| 2029 | PEETAAEESWD | 0 | 0.001290694 | 0 |
| 2030 | TNPEETAAEESWD | 0 | 0.035739743 | 0 |
| 2031 | TNPEETAAEESWDYV | 0 | 0.007117801 | 0 |
| 2032 | NPEETAAEESWDYV | 0 | 0.000634135 | 0 |
| 2033 | NPEETAAEESWDYVQ | 0 | 0.00150265 | 0 |
| 2034 | TNPEETAAEESWDYVQ | 0 | 0.005378493 | 0 |
| 2035 | DEGTAQEGKAAATPES | 0 | 0 | 0.012535984 |
| 2036 | DEGTAQEGKAAATPESQ | 0 | 0 | 0.001639395 |
| 2037 | EGTAQEGKAAATPES | 0 | 0 | 0.0023725 |
| 2038 | SDEGTAQEGKAAATPESQ | 0 | 0 | 0.001884781 |
| 2039 | SDEGTAQEGKAAATPES | 0 | 0.000158797 | 0.005095354 |
| 2040 | GEQPRGGGPTSSEQ | 0 | 0 | 0.000562508 |
| 2041 | SGEQPRGGGPTSSEQ | 0 | 0 | 0.001313975 |
| 2042 | SGEQPRGGGPTSSEQI | 0 | 0 | 0.001019027 |
| 2043 | SGEQPRGGGPTSSEQIM | 0 | 0 | 0.002006503 |
| 2044 | EESDLEIDKEGVIE | 0.003934834 | 0.001466035 | 0.000462162 |
| 2045 | EESDLEIDKEGVIEPD | 0.002059153 | 0 | 0 |
| 2046 | ESDLEIDKEGVIE | 0.003115885 | 0.000920437 | 0 |
| 2047 | ESDLEIDKEGVIEPD | 0.001885033 | 0.000133753 | 0 |
| 2048 | APTQESAVTPGTPD | 0.000889265 | 0.000130793 | 0.001894348 |
| 2049 | APTQESAVTPGTPDPT | 0 | 0 | 0.009012467 |
| 2050 | APTQESAVTPGTPDPTCAG | 0 | 0 | 0.010570604 |
| 2051 | EEGAVGGAAEETGRR | 0 | 0 | 0.000448928 |
| 2052 | LNWAVAAGAEDPGPA | 0.001077403 | 0.001159468 | 0.002601023 |
| 2053 | DLQRSSSEQAM | 1.82955E-05 | 0.001856864 | 0 |
| 2054 | DLQRSSSEQAMR | 9.59909E-06 | 0.005918768 | 5.06149E-05 |
| 2055 | SDLQRSSSEQAMR | 0 | 0.005136385 | 0 |
| 2056 | YSDLQRSSSEQAMR | 0 | 0.020448392 | 2.16372E-05 |
| 2057 | LQRSSSEQAMR | 0 | 0.001631669 | 0 |
| 2058 | QRSSSEQAMR | 0 | 0.000107401 | 0 |
| 2059 | SDLQRSSSEQAM | 0 | 0.002358634 | 0 |
| 2060 | YSDLQRSSSEQAM | 0 | 0.010293226 | 0.000425428 |
| 2061 | ERPGSEEPPAEP | 8.60944E-05 | 0.00117484 | 0 |
| 2062 | ERPGSEEPPAEPVS | 0 | 0.000612737 | 0.000100569 |
| 2063 | RPGSEEPPAEP | 0 | 0.000192132 | 0 |
| 2064 | RRERPGSEEPPAEPVS | 4.07751E-05 | 0.001408067 | 4.69109E-05 |
| 2065 | RRERPGSEEPPAEP | 0.00017904 | 0.001013156 | 0 |
| 2066 | SPVECDFTSPGDEGPPR | 0.000806927 | 0 | 0 |
| 2067 | VGLTPSAAQTARQHPK | 0 | 4.44149E-05 | 0.000467325 |
| 2068 | AAERPGEAAVASSPS | 0 | 0 | 0.000732724 |
| 2069 | AAERPGEAAVASSPSK | 0 | 0 | 0.000761974 |
| 2070 | AIVGTAESNSEHPLG | 0 | 0.000289492 | 0 |
| 2071 | IVGTAESNSEHPLG | 0 | 0.00351136 | 0 |
| 2072 | AQSQTDRANLG | 0 | 0.000970335 | 0 |
| 2073 | AQSQTDRANLGT | 0 | 0.000209219 | 0 |
| 2074 | AQSQTDRANLGTL | 0.000173476 | 0.00105053 | 2.77268E-05 |
| 2075 | AQSQTDRANLGTLR | 0 | 0.001222292 | 3.67672E-05 |
| 2076 | NVKAQSQTDRANLGTLR | 0 | 0.002172769 | 0 |
| 2077 | VKAQSQTDRANLGTLR | 0 | 0.004368996 | 5.0841E-05 |
| 2078 | KAQSQTDRANLGTL | 0 | 0.001614315 | 0 |
| 2079 | NVKAQSQTDRANLGTL | 0 | 0.002771007 | 0 |
| 2080 | VKAQSQTDRANLGTL | 0 | 0.007414607 | 0 |
| 2081 | VKAQSQTDRANLGT | 0 | 0.001432025 | 8.37009E-05 |
| 2082 | NVKAQSQTDRANLG | 0 | 0.00226527 | 0 |
| 2083 | NVKAQSQTDRANLGT | 0 | 0.00038787 | 0 |
| 2084 | VKAQSQTDRANLG | 0 | 0.004181616 | 0 |
| 2085 | QTRNVKAQSQTDRANLG | 0 | 0.000143093 | 9.15415E-05 |
| 2086 | RNVKAQSQTDRANLG | 0 | 0.000393699 | 0 |
| 2087 | WDLQTRNVKAQSQTDR | 0 | 0.00029432 | 0 |
| 2088 | VPDPDAEKPEDWD | 0.001705778 | 0.000122629 | 4.02757E-05 |
| 2089 | VPDPDAEKPEDWDE | 0.000881556 | 0 | 4.75365E-05 |
| 2090 | VPDPDAEKPEDWDED | 0.001236795 | 0 | 0 |
| 2091 | PNPNPNHPSS | 0 | 6.77259E-05 | 0 |
| 2092 | PNPNPNHPSSSG | 0 | 0.000715537 | 0 |
| 2093 | PNPNPNHPSSSGS | 0 | 0.000101058 | 0 |
| 2094 | PRPPNPPKPMPNPNPNHPSSSGS | 0 | 0.000327179 | 0 |
| 2095 | PRPPNPPKPMPNPNPNHPSS | 0 | 0.000411579 | 0 |
| 2096 | PRPPNPPKPMPNPNPNHPSSSG | 0 | 0.00677705 | 0.000691505 |
| 2097 | AQSQTDRVDLG | 0.000385607 | 0.000717997 | 6.62355E-05 |
| 2098 | AQSQTDRVDLGTLR | 4.16868E-05 | 0.001979586 | 0 |
| 2099 | VKAQSQTDRVDLGTLR | 0 | 0.005549485 | 0.000331436 |
| 2100 | VKAQSQTDRVDLG | 0 | 0.005464679 | 0.000171448 |
| 2101 | VKAQSQTDRVDLGT | 0 | 0.000752348 | 2.33289E-05 |
| 2102 | VKAQSQTDRVDLGTL | 5.86641E-05 | 0.00630316 | 0 |
| 2103 | APESGLAEYL | 0 | 0.001584418 | 0 |
| 2104 | APESGLAEYLFDK | 0 | 0.032589455 | 0.000755875 |
| 2105 | APESGLAEYLFDKH | 0 | 0.031604252 | 0.001042178 |
| 2106 | APESGLAEYLFDKHT | 0 | 0.009659212 | 0.00045251 |
| 2107 | GAPESGLAEYLFDK | 0.000287637 | 0.003630953 | 5.57595E-05 |
| 2108 | GAPESGLAEYLFDKH | 0 | 0.001667996 | 0.000731021 |
| 2109 | KMGAPESGLAEYLFDKH | 0 | 0.001855456 | 0.000260641 |
| 2110 | MGAPESGLAEYLFDK | 0 | 0.008799184 | 0.002407439 |
| 2111 | KMGAPESGLAEYL | 0 | 0.002807754 | 4.49084E-05 |
| 2112 | KMGAPESGLAEYLFDK | 0.000183176 | 0.011679233 | 0.001323323 |
| 2113 | RKMGAPESGLAEYLFDK | 0 | 0.001061824 | 0 |
| 2114 | APQGGLAEFNPFS | 0 | 0 | 0.002376444 |
| 2115 | LTNAPQGGLAEFNPFS | 0 | 0 | 0.00513896 |
| 2116 | LTNAPQGGLAEFNPFSE | 0 | 0 | 0.002086433 |
| 2117 | NAPQGGLAEFNPFS | 0 | 0 | 0.000714315 |
| 2118 | TNAPQGGLAEFNPFS | 0 | 0.000186 | 0.002919584 |
| 2119 | LIKMASTDTPMAR | 0.000274148 | 0.001651778 | 0.00067937 |
| 2120 | NPTEPGSDGTMSDKE | 0 | 0.000603921 | 0 |
| 2121 | INSPEGAVGRSLQPL | 0 | 0 | 0.002319853 |
| 2122 | RLLPGNATISK | 0 | 7.79238E-05 | 0 |
| 2123 | DSTGKYWTVGSDSA | 0 | 0.000525461 | 0 |
| 2124 | KYWTVGSDSAVT | 0 | 5.08045E-05 | 0 |
| 2125 | AVNSIGKSLPSEASEQ | 0 | 0 | 0.000504827 |
| 2126 | SIGKSLPSEASEQ | 0 | 0 | 0.001833656 |
| 2127 | GPSPGEGISPQSAQAPQAP | 0 | 2.49383E-05 | 0.003079276 |
| 2128 | GPSPGEGISPQSAQAPQAPG | 0 | 0.000668861 | 0.004560615 |
| 2129 | GPSPGEGISPQSAQAPQAPGD | 0 | 0 | 0.000797787 |
| 2130 | GPSPGEGISPQSAQAPQAPGDN | 0 | 0.005868985 | 0.017021693 |
| 2131 | LPILKRAVAGDASESALL | 5.26047E-05 | 0 | 0.002968063 |
| 2132 | RAVAGDASESA | 0.000491187 | 0 | 0.000499216 |
| 2133 | RAVAGDASESAL | 0.004881347 | 0 | 0.00427133 |
| 2134 | VPPNSPQGSVACPPPP | 0 | 0 | 0.005655024 |
| 2135 | VPPNSPQGSVACPPPPA | 0 | 0 | 0.002597021 |
| 2136 | LPYHPAQPGQAA | 0 | 0 | 0.001208124 |
| 2137 | LPYHPAQPGQAAK | 0 | 1.07535E-05 | 0.003702527 |
| 2138 | LPYHPAQPGQAAKK | 0 | 0 | 0.001416278 |
| 2139 | SPPGIDPTRGSQGAQSSP | 0 | 0 | 0.001133028 |
| 2140 | ELEDATETADAM | 0 | 0 | 0 |
| 2141 | ELEDATETADAMNR | 0 | 0.000129092 | 0 |
| 2142 | ELEDATETADAMNRE | 0 | 0.001613148 | 0 |
| 2143 | RELEDATETADAMNRE | 0 | 0.000266028 | 0 |
| 2144 | RELEDATETADAM | 0 | 0.002148739 | 0.00152747 |
| 2145 | RELEDATETADAMN | 0 | 0.001464007 | 0 |
| 2146 | RMVVGGAQAGDAG | 2.93042E-05 | 0 | 0.000692284 |
| 2147 | RMVVGGAQAGDAGT | 0 | 0 | 0.00210589 |
| 2148 | RMVVGGAQAGDAGTY | 0 | 0 | 0.009100121 |
| 2149 | RMVVGGAQAGDAGTYH | 0 | 0 | 0.001194659 |
| 2150 | YRMVVGGAQAGDAGTYH | 0 | 0.000347498 | 0.015197991 |
| 2151 | YRMVVGGAQAGDAG | 0 | 0.000122204 | 0.001998723 |
| 2152 | YRMVVGGAQAGDAGTY | 0 | 0 | 0.016494357 |
| 2153 | VVGGAQAGDAGTYH | 5.7383E-05 | 0 | 0.000644682 |
| 2154 | APGTPEGPEPERPSPG | 0.004365809 | 0 | 6.62391E-05 |
| 2155 | APGTPEGPEPERPSPGD | 0.001310949 | 0 | 0 |
| 2156 | TPEGPEPERPSPG | 0.000482529 | 0 | 0 |
| 2157 | GPGGPEGGDAEGPIT | 0 | 0 | 0.002677148 |
| 2158 | GPPAQGSEAQLIS | 6.41711E-05 | 0 | 0.002610866 |
| 2159 | GPPAQGSEAQLISFD | 0 | 3.79375E-05 | 0.006731929 |
| 2160 | PPFNPHGGAEPGADSAEGDVG | 0.014372114 | 0 | 0 |
| 2161 | PPFNPHGGAEPGADSAEGDVGDG | 0.001054304 | 2.87917E-05 | 0 |
| 2162 | RPPFNPHGGAEPGADSAEGDVGDG | 0.001224418 | 0 | 0 |
| 2163 | RPPFNPHGGAEPGADSAEGDVG | 0.007794607 | 7.02804E-05 | 0 |
| 2164 | DPESGEADEVP | 0 | 0.000707677 | 0 |
| 2165 | DPESGEADEVPA | 0 | 0.005004617 | 0 |
| 2166 | DPESGEADEVPAY | 0 | 0.001459493 | 0 |
| 2167 | FSSDPESGEADEVPAY | 0 | 0.005287544 | 0 |
| 2168 | SSDPESGEADEVPAY | 0 | 0.002147332 | 0 |
| 2169 | PESGEADEVPA | 0 | 0.001099992 | 0 |
| 2170 | SDPESGEADEVPA | 0 | 0.001451356 | 0 |
| 2171 | SSDPESGEADEVPA | 0 | 0.012491428 | 0 |
| 2172 | FSSDPESGEADEVP | 0 | 0.004606271 | 0 |
| 2173 | FSSDPESGEADEVPA | 0 | 0.012522937 | 5.53058E-05 |
| 2174 | SDPESGEADEVP | 0 | 0.001107662 | 0 |
| 2175 | SSDPESGEADEVP | 0 | 0.002102318 | 0 |
| 2176 | EDTDSSDAENEINEH | 0 | 0.000453915 | 0 |
| 2177 | LEITESQSADAEPPPPP | 0 | 0.001603706 | 0 |
| 2178 | IHKNPNTSEPQHL | 0 | 0.010472073 | 0 |
| 2179 | IHKNPNTSEPQHLL | 0.000145077 | 0.004381906 | 0.000893896 |
| 2180 | IHKNPNTSEPQHLLV | 0 | 0.000541579 | 0 |
| 2181 | KNPNTSEPQHL | 0 | 2.86145E-05 | 0 |
| 2182 | NPNTSEPQHL | 0 | 0.019819611 | 0 |
| 2183 | SIHKNPNTSEPQHL | 0 | 0.002920676 | 1.01331E-05 |
| 2184 | NPNTSEPQHLL | 0 | 0.002210846 | 0.001560608 |
| 2185 | SIHKNPNTSEPQHLL | 0 | 0.00225518 | 4.48794E-05 |
| 2186 | SPGESEHLLEPAEAERSQ | 0 | 2.08094E-05 | 0.017851687 |
| 2187 | SPGESEHLLEPAEAERSQR | 0 | 0 | 0.004146111 |
| 2188 | SPGESEHLLEPAEAERSQRRR | 0 | 0 | 0.009477043 |
| 2189 | TPPSEEDSAEAERL | 0 | 0.000293334 | 0 |
| 2190 | TPPSEEDSAEAERLK | 0 | 0.001393867 | 5.6953E-05 |
| 2191 | DDLGSYQGAQGNRYVG | 0 | 0 | 0 |
| 2192 | GSINSTDSERWK | 0 | 0.000440365 | 0 |
| 2193 | HTGSINSTDSERWK | 0.000460659 | 0.003087194 | 0 |
| 2194 | INSTDSERWK | 0 | 0.000702407 | 0 |
| 2195 | SINSTDSERWK | 0 | 0.000637418 | 0 |
| 2196 | TGSINSTDSERWK | 9.27411E-05 | 0.002098749 | 1.72764E-05 |
| 2197 | DRVAGESAEPEP | 0.000523676 | 0.000218467 | 0 |
| 2198 | DRVAGESAEPEPEP | 0.004261261 | 0.011129089 | 2.41391E-05 |
| 2199 | DRVAGESAEPEPEPE | 0.005078133 | 0.033049379 | 0 |
| 2200 | DRVAGESAEPEPEPEA | 0.000191179 | 0.004545716 | 0 |
| 2201 | DRVAGESAEPEPEPEAD | 0 | 0.002369769 | 0 |
| 2202 | RDRVAGESAEPEPEPEAD | 0.000455802 | 0.002140706 | 0 |
| 2203 | RDRVAGESAEPEP | 0.007669733 | 0.002056783 | 9.03697E-05 |
| 2204 | RDRVAGESAEPEPE | 0.001120951 | 0.002148664 | 2.89094E-05 |
| 2205 | RDRVAGESAEPEPEP | 0.036405196 | 0.027037135 | 8.26731E-06 |
| 2206 | RDRVAGESAEPEPEPE | 0.04313832 | 0.046613618 | 9.34066E-05 |
| 2207 | RDRVAGESAEPEPEPEA | 0.001774862 | 0.004439175 | 2.39514E-05 |
| 2208 | TRDRVAGESAEPEPEPEA | 8.66818E-05 | 0.000775687 | 3.1606E-05 |
| 2209 | RVAGESAEPEPEP | 0.002623819 | 0.001116 | 0 |
| 2210 | RVAGESAEPEPEPE | 0.003280658 | 0.002851423 | 5.59741E-05 |
| 2211 | STRDRVAGESAEPEPEPE | 0.009820413 | 0.010026374 | 0 |
| 2212 | TRDRVAGESAEPEPEPE | 0.014109308 | 0.010293906 | 0.000442827 |
| 2213 | STRDRVAGESAEPEPEP | 0.005310442 | 0.002500203 | 0.000153144 |
| 2214 | TRDRVAGESAEPEPE | 0.000314703 | 0.000248724 | 0 |
| 2215 | TRDRVAGESAEPEPEP | 0.005333838 | 0.003979382 | 0 |
| 2216 | FDRLSTEGSDQEKED | 0 | 0.000393253 | 0 |
| 2217 | FDRLSTEGSDQEKEDD | 0 | 0.001136286 | 0 |
| 2218 | GFDRLSTEGSDQEKEDD | 0 | 0.002689995 | 0 |
| 2219 | GFDRLSTEGSDQEKEDDG | 0 | 0.000210647 | 0 |
| 2220 | EPEKVETGSEPGDTEP | 3.85547E-05 | 0.002119921 | 0 |
| 2221 | EPEKVETGSEPGDTEPL | 0 | 0.002229871 | 0 |
| 2222 | EPISEPEKVETGSEPGDTEPL | 0 | 0.002982097 | 0 |
| 2223 | DEVEAAAQAAGIHD | 9.20831E-05 | 0 | 0.001558953 |
| 2224 | DEVEAAAQAAGIHDA | 0.000172307 | 0 | 0.020489528 |
| 2225 | GNDEVEAAAQAAGIHDA | 0.000313765 | 0 | 0.001424036 |
| 2226 | NDEVEAAAQAAGIHDA | 0 | 6.15064E-05 | 0.026534604 |
| 2227 | NDEVEAAAQAAGIHD | 5.48429E-05 | 0 | 0.001950834 |
| 2228 | EVEAAAQAAGIHDA | 0 | 0 | 0.001942268 |
| 2229 | SPVESTTEPPAVR | 2.17703E-05 | 0.000874545 | 0.000203783 |
| 2230 | EIQTSPETSMR | 0 | 0.000388077 | 7.69188E-05 |
| 2231 | VNRLLDSLEPPGEPGP | 0 | 0.001936743 | 0.000833471 |
| 2232 | VDPTTNSARVYNGAAD | 0 | 0 | 0.002062945 |
| 2233 | EDALSSDTSGHFR | 0 | 0.000222706 | 0 |
| 2234 | AGPEGVAPAPEGEK | 0.000830293 | 0 | 0 |
| 2235 | SDNWEAITGTGDPEH | 0 | 0 | 0 |
| 2236 | YSDNWEAITGTGDPEH | 0.00155624 | 0.000135896 | 0 |
| 2237 | ALQPGQAVPAGSHVR | 0 | 0 | 0.004606155 |
| 2238 | QALQPGQAVPAGSHVR | 0 | 0 | 0.004032765 |
| 2239 | QPGQAVPAGSHVR | 0 | 0 | 0.001062175 |
| 2240 | AMNPTNTVF | 0 | 0 | 0.001254545 |
| 2241 | TPTVTYQRGGEAVSSGGRPG | 0 | 6.26552E-05 | 0.00025899 |
| 2242 | DGTVPFWAHAGNAIPSSDQIR | 0.000338487 | 0 | 0.001126943 |
| 2243 | VPFWAHAGNAIPSSD | 0 | 0.000122645 | 0.000928676 |
| 2244 | VPFWAHAGNAIPSSDQ | 0 | 0.000559435 | 0.022971036 |
| 2245 | VPFWAHAGNAIPSSDQI | 0 | 0.000914103 | 0.006841104 |
| 2246 | VPFWAHAGNAIPSSDQIR | 0.000753596 | 0 | 0.00934674 |
| 2247 | WAHAGNAIPSSDQ | 5.12431E-05 | 0 | 0.000602932 |
| 2248 | LSDDVETDTVAPGEN | 0 | 0.000427538 | 0 |
| 2249 | SPSWQRPNQGVPSTGRIS | 0 | 0 | 0.000817551 |
| 2250 | IPGSPEPEHGSVQESQ | 0 | 0 | 0.001306168 |
| 2251 | GVSFDKTSATWL | 0 | 0.003366883 | 0.000443459 |
| 2252 | SGVSFDKTSATWL | 0 | 0.00115727 | 0 |
| 2253 | SPVNSGQPPNYE | 0 | 0.001333759 | 0 |
| 2254 | SVENKIVSLDPSEAGPPR | 0.000143907 | 0.001580767 | 0 |
| 2255 | VENKIVSLDPSEAGPP | 0 | 0.002953192 | 0.000211022 |
| 2256 | VENKIVSLDPSEAGPPR | 4.66983E-05 | 0.006382611 | 0 |
| 2257 | VDRLIALDAAEEF | 0.000829147 | 0 | 7.33862E-05 |
| 2258 | VDRLIALDAAEEFFK | 0.001864739 | 9.81322E-05 | 0 |
| 2259 | VDDRLVTMQIWDTA | 2.91319E-05 | 0.001292746 | 0.000787682 |
| 2260 | VDDRLVTMQIWDTAG | 0 | 0.004035741 | 0.000342722 |
| 2261 | ADIMIGFARGAHGDS | 0 | 0 | 0.000768596 |
| 2262 | GTADIMIGFARGAHGDS | 0 | 8.01168E-05 | 0.00221832 |
| 2263 | GTADIMIGFARGAHG | 0 | 0 | 0.000695987 |
| 2264 | TADIMIGFARGAHG | 0 | 0 | 0.000978772 |
| 2265 | ATSQVLLPSK | 0 | 0.000407129 | 0 |
| 2266 | GGKYAATSQVLLPSK | 0.001620558 | 0.025595878 | 0 |
| 2267 | GGKYAATSQVLLPSKD | 0.000958395 | 0.005559668 | 0.000424788 |
| 2268 | GGKYAATSQVLLPSKDV | 0 | 0.001004619 | 0 |
| 2269 | GKYAATSQVLLPSK | 7.38894E-05 | 0.004344459 | 0 |
| 2270 | KYAATSQVLLPSK | 0 | 0.003551561 | 0.000118994 |
| 2271 | YAATSQVLLPSK | 0 | 0.000163241 | 0 |
| 2272 | GKYAATSQVLLPSKD | 0 | 0.001291053 | 0 |
| 2273 | GGKYAATSQVLLP | 0.001216898 | 0.002981995 | 0 |
| 2274 | GGKYAATSQVLLPS | 0.006612904 | 0.013684354 | 0.000220921 |
| 2275 | RGGKYAATSQVLLPS | 0.002260757 | 0.000188612 | 0 |
| 2276 | RGGKYAATSQVLLPSK | 0.000708245 | 0.009375584 | 0 |
| 2277 | RGGKYAATSQVLLPSKD | 6.76081E-05 | 0.001837406 | 0 |
| 2278 | RGGKYAATSQVLLP | 0.000317941 | 0 | 0 |
| 2279 | APQILLSGTAHFARPA | 0 | 3.97757E-05 | 0.002895722 |
| 2280 | LSGTAHFARPAVDFEG | 0 | 0 | 0.001385861 |
| 2281 | SGTAHFARPAVDFEG | 0 | 0 | 0.004697766 |
| 2282 | ATTDTVTEVVVPFN | 0 | 0.00455276 | 0.000120041 |
| 2283 | RATTDTVTEVVVPFN | 0 | 0.003012484 | 0.000459569 |
| 2284 | SRATTDTVTEVVVPFN | 0 | 0.003220697 | 0.00214296 |
| 2285 | TDTVTEVVVPFN | 0.000316337 | 0.00183169 | 4.09391E-05 |
| 2286 | TTDTVTEVVVPFN | 0.000221649 | 0.002004545 | 0 |
| 2287 | RATTDTVTEVVVP | 0 | 0.001310408 | 0 |
| 2288 | SRATTDTVTEVVVP | 0.000527496 | 0.007091972 | 9.36379E-05 |
| 2289 | KVANIILSY | 0 | 0.005525009 | 0.001487683 |
| 2290 | TVTEVLLKY | 0 | 0.008224783 | 0.002552661 |
| 2291 | APANALPARSEAA | 0 | 0 | 0 |
| 2292 | APANALPARSEAAAVQPVIG | 0 | 0 | 0.000764175 |
| 2293 | VFAPANALPARSE | 0 | 0 | 0 |
| 2294 | VFAPANALPARSEA | 0 | 8.0599E-05 | 0.002931732 |
| 2295 | VFAPANALPARSEAA | 0 | 0.000585408 | 0.00579425 |
| 2296 | VQVFAPANALPARSE | 0 | 0 | 0.00161549 |
| 2297 | VQVFAPANALPARSEA | 0 | 0 | 0.002850797 |
| 2298 | LPARSEAAAVQPVIG | 0.000438966 | 0.001870847 | 0.100983625 |
| 2299 | NVPVYSSGDTVSGRVN | 0 | 0 | 0 |
| 2300 | VPVYSSGDTVSGRVN | 0 | 0 | 0.005271169 |
| 2301 | SPGDTVASKASAEGGS | 0 | 0 | 0.002021919 |
| 2302 | ATWTIQGAANALSG | 0 | 0 | 0.012969568 |
| 2303 | ATWTIQGAANALSGDV | 0 | 0 | 0.468680059 |
| 2304 | ATWTIQGAANALSGDVW | 6.30798E-05 | 0 | 0.212206858 |
| 2305 | ATWTIQGAANALSGDVWD | 0.000797051 | 0 | 0.031899815 |
| 2306 | ATWTIQGAANALSGDVWDID | 0 | 0 | 0.001456413 |
| 2307 | ATWTIQGAANALSGDVWDIDN | 0 | 0 | 0.005518135 |
| 2308 | TWTLQGAANALSG | 0 | 0 | 0.002897543 |
| 2309 | WTLQGAANALSG | 0 | 0 | 0.007829371 |
| 2310 | TWTLQGAANALSGDV | 0.000261866 | 0.000349563 | 0.202495003 |
| 2311 | WTLQGAANALSGDV | 0 | 0 | 0.126871964 |
| 2312 | TLQGAANALSGDV | 0 | 0 | 0.002600326 |
| 2313 | TLQGAANALSGDVW | 0 | 0 | 0.0072743 |
| 2314 | TWTLQGAANALSGDVW | 0 | 0 | 0.036056497 |
| 2315 | WTLQGAANALSGDVW | 0 | 0 | 0.053364894 |
| 2316 | IQGAANALSGDVWDID | 0.009545713 | 0 | 0 |
| 2317 | IQGAANALSGDVWDIDN | 0.011889749 | 0 | 0 |
| 2318 | IQGAANALSGDVWDIDNE | 0.000667478 | 0.000669914 | 0 |
| 2319 | WTIQGAANALSGDVWDIDNE | 0 | 0 | 0.005352862 |
| 2320 | WTIQGAANALSGDVWDIDN | 0 | 0 | 0.001855314 |
| 2321 | DPSKPGGNVEGATQSLAEQ | 0 | 0 | 0.001769876 |
| 2322 | GPETVGSEVEALN | 0 | 0.001099007 | 2.4718E-05 |
| 2323 | RPPAPESVGTEEMPED | 0.001313526 | 0.005334505 | 0 |
| 2324 | RPPAPESVGTEEMPEDG | 0.000146034 | 0.000496835 | 0 |
| 2325 | RPPAPESVGTEEMPEDGEPD | 0.000145509 | 0.000738425 | 0 |
| 2326 | INVIAQSLSTENIK | 0 | 0.000294208 | 0 |
| 2327 | AKVTLNGVPAQPLGPR | 0.000314645 | 0.00031098 | 0 |
| 2328 | KVTLNGVPAQPLGPR | 0.000146928 | 0.001534264 | 0.000126648 |
| 2329 | TVETLSGQAVVSYH | 0 | 0 | 0 |
| 2330 | TVETLSGQAVVSYHT | 0 | 0 | 0.000340005 |
| 2331 | TVETLSGQAVVSYHTV | 0 | 0 | 0.002886335 |
| 2332 | VTVETLSGQAVVSYH | 0 | 0 | 0.012171564 |
| 2333 | VTVETLSGQAVVSYHT | 0 | 0 | 0.000198844 |
| 2334 | WISVDSVTSEIK | 0 | 0.001271077 | 0.000104069 |
| 2335 | WISVDSVTSEIKL | 0 | 0.001037511 | 6.55613E-05 |
| 2336 | ETLKEPETVGAQSI | 0 | 0.000442829 | 0.000128037 |
| 2337 | DDLQPWHSFGADSVP | 0.001054697 | 0.009144323 | 0 |
| 2338 | DDLQPWHSFGADSVPA | 0.02000566 | 0.090521759 | 0 |
| 2339 | DDLQPWHSFGADSVPAN | 0.020916418 | 0.127352291 | 0.000221645 |
| 2340 | DDLQPWHSFGADSVPANT | 0.002641627 | 0.079711855 | 0 |
| 2341 | DDLQPWHSFGADSVPANTE | 0.005592883 | 0.0369932 | 0 |
| 2342 | DDLQPWHSFGADSVPANTEN | 0.00028054 | 0.004654886 | 0 |
| 2343 | LDDLQPWHSFGADSVPANTEN | 0.000370552 | 0.004210146 | 0 |
| 2344 | LQPWHSFGADSVP | 0 | 0.002962453 | 0 |
| 2345 | SLDDLQPWHSFGADSVPANTEN | 0.000119952 | 0.001378001 | 0 |
| 2346 | LQPWHSFGADSVPANT | 0 | 0.020188549 | 0 |
| 2347 | LQPWHSFGADSVPANTE | 0.000257571 | 0.004618251 | 0 |
| 2348 | DLQPWHSFGADSVPA | 0.01596489 | 0.034684284 | 0 |
| 2349 | DLQPWHSFGADSVPAN | 0.011722549 | 0.031635006 | 0 |
| 2350 | DLQPWHSFGADSVPANT | 0.000137657 | 0.016252927 | 0.000274659 |
| 2351 | DLQPWHSFGADSVPANTE | 0 | 0.007452002 | 0 |
| 2352 | DLQPWHSFGADSVPANTEN | 0.000193274 | 0.000977544 | 0 |
| 2353 | LQPWHSFGADSVPA | 0.026551433 | 0.313852258 | 0 |
| 2354 | QPWHSFGADSVPA | 0.00021265 | 0.000441178 | 0 |
| 2355 | EFSLDDLQPWHSFGADSVPANTE | 0 | 0.000625527 | 0 |
| 2356 | LDDLQPWHSFGADSVP | 0.000519209 | 0.002530527 | 0 |
| 2357 | LDDLQPWHSFGADSVPA | 0.003836463 | 0.007481854 | 0 |
| 2358 | LDDLQPWHSFGADSVPAN | 0.008348987 | 0.03207177 | 0.000139037 |
| 2359 | LDDLQPWHSFGADSVPANT | 0.001106027 | 0.031988705 | 0 |
| 2360 | LDDLQPWHSFGADSVPANTE | 0.00145239 | 0.027045081 | 0 |
| 2361 | SLDDLQPWHSFGADSVPANTE | 9.49869E-05 | 0.006479333 | 0 |
| 2362 | GEFSLDDLQPWHSFGADSVPAN | 0.000493628 | 0.00198965 | 0 |
| 2363 | LQPWHSFGADSVPAN | 0.001786959 | 0.06028965 | 0 |
| 2364 | QPWHSFGADSVPAN | 0.001752976 | 0.000266504 | 0 |
| 2365 | SLDDLQPWHSFGADSVPAN | 0.001358848 | 0.005133424 | 0 |
| 2366 | SLDDLQPWHSFGADSVP | 0 | 0.000662923 | 0 |
| 2367 | SLDDLQPWHSFGADSVPANT | 0 | 0.006046169 | 0 |
| 2368 | QPWHSFGADSVPANTE | 0.006728902 | 0 | 0.00053408 |
| 2369 | HSFGADSVPANTENEV | 0 | 0.002634404 | 0 |
| 2370 | HSFGADSVPANTENEVE | 0 | 0.001621784 | 0 |
| 2371 | HSFGADSVPANTENEVEP | 0 | 0.003475772 | 0 |
| 2372 | SFGADSVPANTENE | 0 | 0.002376933 | 0 |
| 2373 | LPVNGEFSLDDLQPW | 0.001521544 | 0 | 0 |
| 2374 | LPVNGEFSLDDLQPWH | 0.01198884 | 0 | 0 |
| 2375 | LPVNGEFSLDDLQPWHSFG | 0.003325569 | 0 | 0 |
| 2376 | DPTQPETIAVMSWMK | 0 | 0.001375261 | 0 |
| 2377 | EFLFYLPGQPEGIR | 0 | 0 | 0 |
| 2378 | EFLFYLPGQPEGIRS | 0.000398686 | 0 | 0.00066074 |
| 2379 | FLFYLPGQPEGIR | 0 | 0 | 0 |
| 2380 | FYLPGQPEGIR | 0 | 0 | 0.004014108 |
| 2381 | NPPPEEFLFYLPGQPEGIRS | 0 | 0.000282199 | 0.019697749 |
| 2382 | NPPPEEFLFYLPGQPEGIRSS | 0 | 0 | 0.02122506 |
| 2383 | NPPPEEFLFYLPGQPEGIRSSN | 0 | 0 | 0.005510236 |
| 2384 | NPPPEEFLFYLPGQPEGIR | 0 | 0 | 0.037609188 |
| 2385 | PPPEEFLFYLPGQPEGIR | 0 | 0 | 0.000143089 |
| 2386 | FLFYLPGQPEGIRS | 0 | 0 | 0.006606508 |
| 2387 | FLFYLPGQPEGIRSS | 0 | 0 | 0.000352178 |
| 2388 | GPGDPDSIRCD | 0 | 0.000722388 | 0 |
| 2389 | GPGDPDSIRCDTR | 0 | 0.000399602 | 0 |
| 2390 | GPGDPDSIRCDTRP | 6.63428E-05 | 0.004392911 | 0 |
| 2391 | GPGDPDSIRCDTRPQ | 0 | 0.003074537 | 0.000111429 |
| 2392 | ISKTGEAETITTHYL | 0 | 0.004911964 | 9.07988E-05 |
| 2393 | KTGEAETITTHYL | 0.000783418 | 0.010117321 | 0.001094301 |
| 2394 | SKTGEAETITTHYL | 0 | 0.001755204 | 0 |
| 2395 | TGEAETITTHYL | 0 | 0.004426638 | 0 |
| 2396 | IYKVPSTEAEAL | 0 | 0.002020972 | 0 |
| 2397 | IYKVPSTEAEALAS | 0 | 0.001733126 | 0 |
| 2398 | YHGHMSGGQAETLLQ | 0 | 0 | 0.001432234 |
| 2399 | YPEAQSVASDILR | 7.64237E-05 | 0.00076495 | 0.005007876 |
| 2400 | GPGDPASDEGDGLPGDRP | 0.000360744 | 0.001021074 | 0.001427705 |
| 2401 | AVSRPGRGEPH | 0 | 0 | 0.000654404 |
| 2402 | AVSRPGRGEPHFI | 0 | 0 | 0.005135619 |
| 2403 | AVSRPGRGEPHFIA | 0 | 0.000151046 | 0.016335578 |
| 2404 | AVSRPGRGEPHFIAVG | 0 | 4.43014E-05 | 0.123294565 |
| 2405 | AVSRPGRGEPHFIAVGYVD | 3.12758E-05 | 0 | 0.003953955 |
| 2406 | AVSRPGRGEPHFIAVGYVDDTQFV | 0 | 0 | 0.001569419 |
| 2407 | AVSRPSRGEPHFI | 0 | 2.34824E-05 | 0.005380002 |
| 2408 | AVSRPSRGEPHFIA | 0 | 5.40081E-06 | 0.021575753 |
| 2409 | AVSRPSRGEPHFIAVG | 6.68565E-05 | 0 | 0.087843185 |
| 2410 | SVSRPGRGEPR | 0 | 0 | 0.003739244 |
| 2411 | SVSRPGRGEPRFI | 0 | 0 | 0.049248714 |
| 2412 | SVSRPGRGEPRFIA | 0 | 0 | 0.02935664 |
| 2413 | SVSRPGRGEPRFIAV | 0 | 0 | 0.000946679 |
| 2414 | SVSRPGRGEPRFIAVG | 0 | 0 | 0.361439188 |
| 2415 | SVSRPGRGEPRFIAVGY | 1.86164E-05 | 0 | 0.005501985 |
| 2416 | SVSRPGRGEPRFIAVGYVD | 9.44405E-05 | 0 | 0.012628192 |
| 2417 | SVSRPGRGEPRFIS | 0 | 1.90603E-05 | 0.003838888 |
| 2418 | SVSRPGRGEPRFISVG | 0 | 0 | 0.051193342 |
| 2419 | STSVSRPGRGEPRFIAVGYVD | 0 | 5.73209E-05 | 0.00076097 |
| 2420 | TSVSRPGRGEPRFIA | 0 | 0 | 0.001178107 |
| 2421 | TSVSRPGRGEPRFIAVG | 0 | 0 | 0.016934371 |
| 2422 | TSVSRPGRGEPRFIAVGYVD | 0 | 0 | 0.004155826 |
| 2423 | VSRPGRGEPHFI | 0 | 0.001784339 | 0.001818291 |
| 2424 | VSRPGRGEPHFIA | 0 | 0 | 0.011042526 |
| 2425 | VSRPGRGEPRFI | 0 | 2.65996E-06 | 0.029998532 |
| 2426 | VSRPGRGEPRFIA | 0 | 0 | 0.013328493 |
| 2427 | VSRPGRGEPRFIS | 0 | 0 | 0.001141574 |
| 2428 | VSRPSRGEPHFI | 0 | 0 | 0.002389294 |
| 2429 | VSRPSRGEPHFIA | 0 | 0 | 0.011279476 |
| 2430 | AVSRPGRGEPRYIAVE | 0 | 0 | 0.000945352 |
| 2431 | RPGRGEPHFIA | 3.41062E-05 | 0 | 0.00645215 |
| 2432 | RPGRGEPRFI | 8.53038E-05 | 0 | 0.008160055 |
| 2433 | RPGRGEPRFIA | 0 | 0 | 0.02557709 |
| 2434 | RPGRGEPRFIAV | 0 | 0 | 0.00082564 |
| 2435 | RPGRGEPRFIT | 0 | 0 | 0.005770194 |
| 2436 | SRPGRGEPRFIAV | 0 | 0 | 0.002530047 |
| 2437 | SRPGRGEPRFISV | 0 | 0 | 0.004120749 |
| 2438 | SRPGRGEPRFITV | 0 | 0 | 0.0002846 |
| 2439 | SRPSRGEPHFIAV | 0 | 0 | 0.003842042 |
| 2440 | VSRPGRGEPRFIAV | 0 | 0 | 7.45614E-05 |
| 2441 | VSRPGRGEPHFIAVG | 0 | 0 | 0.050558855 |
| 2442 | VSRPGRGEPHFIAVGYVD | 0 | 0 | 0.001765836 |
| 2443 | VSRPGRGEPRFIAVG | 0 | 0 | 0.280328956 |
| 2444 | VSRPGRGEPRFIAVGYVD | 0 | 0 | 0.001374824 |
| 2445 | VSRPGRGEPRFISVG | 0 | 0 | 0.03246509 |
| 2446 | VSRPSRGEPHFIAVG | 0 | 0 | 0.048826881 |
| 2447 | SRPGRGEPHFI | 0 | 6.97614E-05 | 0.007223879 |
| 2448 | SRPGRGEPHFIA | 0 | 0 | 0.019397709 |
| 2449 | SRPGRGEPHFIAVG | 0 | 0 | 0.053093972 |
| 2450 | SRPGRGEPHFIAVGYVD | 0 | 0 | 0.0005685 |
| 2451 | SRPGRGEPRFI | 0 | 0 | 0.043835651 |
| 2452 | SRPGRGEPRFIA | 0 | 0 | 0.023303842 |
| 2453 | SRPGRGEPRFIAVG | 0 | 1.0155E-05 | 0.246651972 |
| 2454 | SRPGRGEPRFIAVGY | 0.000326514 | 6.21507E-05 | 0.000943455 |
| 2455 | SRPGRGEPRFIAVGYVD | 0.001257463 | 0 | 0.003462441 |
| 2456 | SRPGRGEPRFIAVGYVDDT | 0.00530742 | 0 | 6.83692E-05 |
| 2457 | SRPGRGEPRFISVG | 0 | 4.57028E-05 | 0.008834495 |
| 2458 | SRPGRGEPRFISVGYVD | 0.003588697 | 0 | 0 |
| 2459 | SRPGRGEPRFISVGYVDD | 0.002035736 | 0 | 0 |
| 2460 | SRPGRGEPRFISVGYVDDT | 0.022811725 | 0 | 0 |
| 2461 | SRPGRGEPRFIT | 0 | 0 | 0.007599623 |
| 2462 | SRPGRGEPRFITVG | 0 | 0 | 0.01632024 |
| 2463 | SRPSRGEPHFI | 0 | 4.04829E-05 | 0.00457934 |
| 2464 | SRPSRGEPHFIA | 6.34569E-06 | 1.83918E-05 | 0.013261428 |
| 2465 | SRPSRGEPHFIAVG | 0 | 0 | 0.024373625 |
| 2466 | VSRPGRGEPRFISVGYVDDT | 0.002783031 | 0 | 5.90465E-05 |
| 2467 | EPHFIAVGYVDDTQ | 0 | 0.00320704 | 0.065948419 |
| 2468 | EPHFIAVGYVDDTQF | 0 | 0 | 0.056002532 |
| 2469 | EPHFIAVGYVDDTQFV | 0 | 0.000100636 | 0.012577564 |
| 2470 | EPHFIAVGYVDDTQFVR | 0 | 0 | 0.011021178 |
| 2471 | EPRFIAVGYVDDTQ | 0 | 0.000353889 | 0.093517884 |
| 2472 | EPRFIAVGYVDDTQF | 0.014254642 | 0.000158193 | 0.084359225 |
| 2473 | EPRFIAVGYVDDTQFV | 0.006350278 | 0 | 0.022053341 |
| 2474 | EPRFIAVGYVDDTQFVR | 0.011114455 | 4.80245E-05 | 0.028382903 |
| 2475 | EPRFISVGYVDDTQ | 0.000380936 | 0.000146531 | 0.003662944 |
| 2476 | EPRFISVGYVDDTQF | 0.047530075 | 0 | 0.003147236 |
| 2477 | EPRFISVGYVDDTQFV | 0.025361568 | 0.000127221 | 0.000131725 |
| 2478 | EPRFISVGYVDDTQFVR | 0.033995088 | 0 | 0.000218825 |
| 2479 | EPRFISVGYVDDTQFVRF | 0.005887284 | 0 | 0 |
| 2480 | EPRFISVGYVDDTQFVRFD | 0.00331049 | 0 | 0 |
| 2481 | GRGEPRFISVGYVDDTQFVRFD | 0.00242707 | 0 | 0 |
| 2482 | PGRGEPRFISVGYVDDTQFVRFD | 0.005471886 | 0 | 0 |
| 2483 | RPGRGEPRFIAVGYVDDTQFVRFD | 0.002254339 | 0 | 0.001648568 |
| 2484 | RPGRGEPRFISVGYVDDTQFVRFD | 0.035444537 | 0 | 0 |
| 2485 | SRPGRGEPRFISVGYVDDTQFVRFD | 0.003390051 | 0 | 0 |
| 2486 | FIAVGYVDDTQF | 0.001821162 | 0 | 0.005398865 |
| 2487 | FIAVGYVDDTQFVR | 0.01976863 | 3.78394E-05 | 0 |
| 2488 | FISVGYVDDTQFV | 0.019689234 | 0 | 0 |
| 2489 | FISVGYVDDTQFVR | 0.207569609 | 0 | 0 |
| 2490 | FISVGYVDDTQFVRF | 0.00578231 | 0 | 0 |
| 2491 | FISVGYVDDTQFVRFD | 0.003306192 | 0 | 0 |
| 2492 | GEPHFIAVGYVDDTQ | 0.000524055 | 0 | 0.009484929 |
| 2493 | GEPHFIAVGYVDDTQF | 0 | 5.56749E-05 | 0.009823496 |
| 2494 | GEPHFIAVGYVDDTQFVR | 0 | 0 | 0.003349774 |
| 2495 | GEPRFIAVGYVDDTQ | 0.001566643 | 6.59103E-05 | 0.005945219 |
| 2496 | GEPRFIAVGYVDDTQF | 0.001867452 | 0 | 0.010175987 |
| 2497 | GEPRFIAVGYVDDTQFV | 0.002214351 | 0 | 0.000544009 |
| 2498 | GEPRFIAVGYVDDTQFVR | 0.000357708 | 7.50737E-05 | 0.00468879 |
| 2499 | GEPRFISVGYVDDTQF | 0.009992192 | 0 | 0 |
| 2500 | GEPRFISVGYVDDTQFV | 0.00509184 | 0 | 0.000252609 |
| 2501 | GEPRFISVGYVDDTQFVR | 0.000362025 | 0 | 0 |
| 2502 | GRGEPRFISVGYVDDTQFVR | 0.002229427 | 0 | 0 |
| 2503 | PGRGEPRFIAVGYVDDTQFVR | 0.0122064 | 0.000195775 | 0.003172534 |
| 2504 | PGRGEPRFISVGYVDDTQFVR | 0.023983647 | 0 | 0 |
| 2505 | PGRGEPRFISVGYVDDTQFVRF | 0.000245185 | 0 | 0 |
| 2506 | RPGRGEPRFISVGYVDDTQFVRF | 0.010003168 | 0 | 0 |
| 2507 | GRGEPHFIAVGYVDDTQ | 0 | 0.000182246 | 0.003682729 |
| 2508 | GRGEPHFIAVGYVDDTQF | 0 | 0 | 0.003435269 |
| 2509 | GRGEPRFIAVGYVD | 0.000966661 | 0 | 8.46135E-05 |
| 2510 | GRGEPRFIAVGYVDD | 0.00112673 | 0 | 0 |
| 2511 | GRGEPRFIAVGYVDDT | 0.012717627 | 7.17441E-05 | 0 |
| 2512 | GRGEPRFIAVGYVDDTQ | 0.000312723 | 0 | 0.007045132 |
| 2513 | GRGEPRFIAVGYVDDTQF | 0 | 0 | 0.002704504 |
| 2514 | GRGEPRFIAVGYVDDTQFV | 0.005188776 | 0 | 0.000345846 |
| 2515 | GRGEPRFISVGYVD | 0.004000836 | 0 | 0 |
| 2516 | GRGEPRFISVGYVDD | 0.003433789 | 0 | 0 |
| 2517 | GRGEPRFISVGYVDDT | 0.035266105 | 0 | 0 |
| 2518 | GRGEPRFISVGYVDDTQ | 0.002871401 | 0 | 0 |
| 2519 | GRGEPRFISVGYVDDTQF | 0.018555068 | 0 | 0 |
| 2520 | GRGEPRFISVGYVDDTQFV | 0.021321965 | 0 | 0 |
| 2521 | PGRGEPRFIAVGYVDDTQFV | 0.004762655 | 0 | 0.0001094 |
| 2522 | PGRGEPRFISVGYVDDTQFV | 0.028738768 | 0.000241873 | 9.95847E-05 |
| 2523 | RPGRGEPHFIAVGYVDDTQFV | 0 | 0 | 0.006209555 |
| 2524 | RPGRGEPHFIAVGYVDDTQFVR | 0 | 0 | 0.002692297 |
| 2525 | RPGRGEPRFIAVGYVDDTQFV | 0.022799282 | 0.000271845 | 0.032852841 |
| 2526 | RPGRGEPRFIAVGYVDDTQFVR | 0.017563712 | 0 | 0.027389966 |
| 2527 | RPGRGEPRFISVGYVDDTQFV | 0.143378746 | 0.001629412 | 0.000178471 |
| 2528 | RPGRGEPRFISVGYVDDTQFVR | 0.085636088 | 0 | 0 |
| 2529 | SRPGRGEPRFISVGYVDDTQFVR | 0.001918384 | 0 | 0 |
| 2530 | RGEPRFIAVGYVDDTQF | 0 | 0 | 0.000460322 |
| 2531 | RGEPRFISVGYVDDT | 0.001868815 | 0 | 0 |
| 2532 | RGEPRFISVGYVDDTQF | 0.001714547 | 0 | 0 |
| 2533 | PGRGEPHFIAVGYVDDTQF | 0 | 0 | 0.005060113 |
| 2534 | PGRGEPRFIAVGYVDDTQ | 0 | 0 | 0.007993153 |
| 2535 | PGRGEPRFIAVGYVDDTQF | 0.019790838 | 0 | 0.00884183 |
| 2536 | PGRGEPRFISVGYVD | 0.006182628 | 0 | 0 |
| 2537 | PGRGEPRFISVGYVDD | 0.001081147 | 0 | 0 |
| 2538 | PGRGEPRFISVGYVDDTQF | 0.040289629 | 0 | 0.00026078 |
| 2539 | RPGRGEPHFIAVGYVDDTQF | 0 | 0 | 0.056024056 |
| 2540 | RPGRGEPRFIAVGYVDDTQF | 0.059847462 | 0 | 0.114373548 |
| 2541 | RPGRGEPRFISVGYVDDTQF | 0.111550047 | 0.000118402 | 0.002454801 |
| 2542 | RPSRGEPHFIAVGYVDDTQF | 0 | 0 | 0.004120807 |
| 2543 | SRPGRGEPHFIAVGYVDDTQF | 0 | 0 | 0.000230609 |
| 2544 | SRPGRGEPRFIAVGYVDDTQF | 0.001083924 | 0 | 0.00268362 |
| 2545 | SRPGRGEPRFIAVGYVDDTQFV | 0.000278465 | 0 | 0.002292361 |
| 2546 | VSRPGRGEPRFIAVGYVDDTQFV | 0 | 0 | 0.000919242 |
| 2547 | RPGRGEPHFIAVG | 0 | 0 | 0.014126673 |
| 2548 | RPGRGEPHFIAVGYVDDTQ | 0 | 0 | 0.03804759 |
| 2549 | RPGRGEPRFIAVG | 5.06236E-05 | 0 | 0.112039646 |
| 2550 | RPGRGEPRFIAVGY | 7.04261E-05 | 0 | 0.001930286 |
| 2551 | RPGRGEPRFIAVGYVD | 0.024082508 | 7.98052E-05 | 0.006941585 |
| 2552 | RPGRGEPRFIAVGYVDD | 0.007241621 | 0 | 3.82284E-05 |
| 2553 | RPGRGEPRFIAVGYVDDT | 0.08724302 | 0.000168076 | 0.001306571 |
| 2554 | RPGRGEPRFIAVGYVDDTQ | 0.011658846 | 0 | 0.105672251 |
| 2555 | RPGRGEPRFISVG | 0.000485663 | 0 | 0.003514709 |
| 2556 | RPGRGEPRFISVGYV | 0.001397641 | 0 | 0 |
| 2557 | RPGRGEPRFISVGYVD | 0.056399084 | 0 | 3.78715E-05 |
| 2558 | RPGRGEPRFISVGYVDD | 0.037723511 | 1.83288E-05 | 0.00142037 |
| 2559 | RPGRGEPRFISVGYVDDT | 0.247345498 | 0 | 0 |
| 2560 | RPGRGEPRFISVGYVDDTQ | 0.02657317 | 0.000323643 | 0.002290102 |
| 2561 | RPGRGEPRFITVG | 0 | 0 | 0.004871399 |
| 2562 | RPSRGEPHFIAVG | 0 | 2.98203E-05 | 0.004111626 |
| 2563 | RPSRGEPHFIAVGYVDDTQ | 0 | 0 | 0.002675733 |
| 2564 | SRPGRGEPRFIAVGYVDDTQ | 2.94659E-05 | 0 | 0.001124037 |
| 2565 | SRPGRGEPRFISVGYVDDTQ | 0.002605566 | 0 | 0 |
| 2566 | EPRFITVGYVDDTL | 0 | 0.000161311 | 0.00341131 |
| 2567 | RPGRGEPRFITVGYVDDTL | 0 | 0 | 0.001010369 |
| 2568 | RPGRGEPRYIAVEYVDDT | 0.006907792 | 0.003795379 | 0.000339226 |
| 2569 | RPGRGEPRYIAVEYVDDTQ | 0 | 0.002506965 | 0 |
| 2570 | MRYFYTAMSRPGRGEP | 0 | 0 | 0.000527571 |
| 2571 | MRYFYTAMSRPGRGEPR | 0 | 0 | 0.004060259 |
| 2572 | MRYFYTAVSRPSR | 0 | 0 | 0.00072098 |
| 2573 | MRYFYTAVSRPSRGEPH | 0 | 0 | 0.051622808 |
| 2574 | SMRYFYTAVSRPGRGEPH | 0 | 0 | 0.001070793 |
| 2575 | SMRYFYTAVSRPSRGEPH | 0 | 0 | 0.029529577 |
| 2576 | RYFFTSVSRPGRGEPR | 0 | 0 | 0.002489394 |
| 2577 | RYFYTAMSRPGRG | 3.13479E-05 | 0 | 0.0028939 |
| 2578 | RYFYTAMSRPGRGEP | 0 | 1.34587E-05 | 0.009593482 |
| 2579 | RYFYTAMSRPGRGEPR | 0 | 1.31621E-05 | 0.006882798 |
| 2580 | RYFYTAVSRPGRGEPH | 0 | 0 | 0.000352544 |
| 2581 | RYFYTAVSRPSRGEPH | 0 | 0 | 0.007057045 |
| 2582 | YFYTAMSRPGRG | 0 | 0 | 0.000841529 |
| 2583 | YFYTAMSRPGRGEP | 0 | 3.74834E-05 | 0.003438134 |
| 2584 | YFYTAMSRPGRGEPR | 0 | 5.00807E-05 | 0.009009003 |
| 2585 | YFYTAVSRPSRGEPH | 0 | 0 | 0.001055952 |
| 2586 | HSLKYFHTSVSRPGRG | 0 | 0 | 0.000325409 |
| 2587 | HSLKYFHTSVSRPGRGEPR | 0 | 0 | 0.002398983 |
| 2588 | LKYFHTSVSRPGRG | 0 | 7.01834E-05 | 0.001409431 |
| 2589 | LKYFHTSVSRPGRGEP | 0 | 0 | 0.000934527 |
| 2590 | LKYFHTSVSRPGRGEPR | 0 | 0 | 0.001864939 |
| 2591 | SLKYFHTSVSRPGRGEPR | 0 | 0 | 0.002124977 |
| 2592 | GSHSMRYFTTSVSRPGRGEPR | 0 | 3.93215E-05 | 0.001844169 |
| 2593 | MRYFSTSVSRPGRG | 2.80346E-05 | 0 | 0.000462787 |
| 2594 | MRYFTTSVSRPGRG | 0 | 4.33365E-05 | 0 |
| 2595 | MRYFTTSVSRPGRGEPR | 0 | 0.000183827 | 0.015842003 |
| 2596 | SMRYFTTSVSRPGRGEPR | 0 | 4.48195E-06 | 0.003192168 |
| 2597 | SMRYFSTSVSRPGRG | 0 | 4.83209E-06 | 0.000600189 |
| 2598 | DDTQFVRFDNDAASP | 0.004756418 | 0 | 0 |
| 2599 | FVRFDNDAASP | 0.002403235 | 0 | 0 |
| 2600 | TQFVRFDNDAASP | 0.000873598 | 0 | 0 |
| 2601 | VDDTQFVRFDNDAASP | 0.035989227 | 0 | 0 |
| 2602 | VDDTQFVRFDNDAASPR | 0.020835153 | 8.72549E-05 | 0.000614063 |
| 2603 | DTQFVRFDNDAASPR | 0.004683631 | 0.00064155 | 0.003128362 |
| 2604 | FVRFDNDAASPR | 0.000414008 | 0 | 0 |
| 2605 | DDTQFVRFDSDAAS | 0.004037238 | 0 | 0 |
| 2606 | DDTQFVRFDSDAASP | 0.131975923 | 0 | 5.8574E-05 |
| 2607 | DDTQFVRFDSDAASPR | 0.183629326 | 0.001293769 | 0 |
| 2608 | DDTQFVRFDSDAASPRG | 0.01171634 | 0 | 0.013201814 |
| 2609 | DDTQFVRFDSDAASPRGEP | 0.000594908 | 0.000882644 | 0 |
| 2610 | DDTQFVRFDSDAASPRGEPR | 0.024048428 | 0.019390469 | 5.12332E-05 |
| 2611 | VDDTQFVRFDSDAASPRGEPR | 0.177238457 | 0.038055575 | 0.000104514 |
| 2612 | VDDTQFVRFDSDAASPRGEPRAP | 0.005401581 | 0 | 0 |
| 2613 | DTQFVRFDSDAASP | 0.12280174 | 0 | 0 |
| 2614 | DTQFVRFDSDAASPR | 0.280547703 | 0.001126192 | 0 |
| 2615 | DTQFVRFDSDAASPRG | 0.006543839 | 0 | 0 |
| 2616 | DTQFVRFDSDAASPRGEPR | 0.019069457 | 0.005919454 | 0 |
| 2617 | FVRFDSDAASP | 0.071053316 | 2.99705E-05 | 0 |
| 2618 | QFVRFDSDAASP | 0.000938667 | 0 | 0 |
| 2619 | TQFVRFDSDAASP | 0.041697997 | 0.00026674 | 0.000355296 |
| 2620 | VRFDSDAASP | 0.001378491 | 0 | 0 |
| 2621 | TQFVRFDSDAASPR | 0.091605853 | 0.000343724 | 0.000107447 |
| 2622 | VDDTQFVRFDSDAASPR | 0.375687887 | 0.005562498 | 0 |
| 2623 | VDDTQFVRFDSDAASPRG | 0.046843607 | 0.002544368 | 0 |
| 2624 | FVRFDSDAASPR | 0.033570814 | 0.005063933 | 0 |
| 2625 | FVRFDSDAASPRG | 0.000895915 | 0.007216533 | 0 |
| 2626 | FVRFDSDAASPRGEP | 0 | 0.036371306 | 0 |
| 2627 | FVRFDSDAASPRGEPR | 0.001104825 | 0.030577558 | 0 |
| 2628 | FVRFDSDATSPR | 0 | 0.000415259 | 0 |
| 2629 | QFVRFDSDAASPRGEPR | 0.000126397 | 0.010676337 | 0 |
| 2630 | TQFVRFDSDAASPRGEPR | 0.000874875 | 0.001168176 | 0 |
| 2631 | VRFDSDAASPR | 0.00075401 | 0 | 0 |
| 2632 | QFVRFDSDAASPR | 0.006683287 | 0.000382337 | 0 |
| 2633 | QFVRFDSDAASPRGEP | 0 | 0.000662704 | 0 |
| 2634 | VDDTQFVRFDSDAASPRGEP | 0.011824457 | 0.002977938 | 0 |
| 2635 | DDTQFVRFDSDAASQ | 0.011920757 | 0 | 0 |
| 2636 | DDTQFVRFDSDAASQK | 0.028481736 | 0 | 4.19159E-05 |
| 2637 | DTQFVRFDSDAASQ | 0.015078328 | 0.002564081 | 0 |
| 2638 | FVRFDSDAASQ | 0.003150153 | 0 | 1.8919E-05 |
| 2639 | TQFVRFDSDAASQ | 0.006218922 | 0 | 0 |
| 2640 | VDDTQFVRFDSDAASQK | 0.078615258 | 0 | 0 |
| 2641 | VDDTQFVRFDSDAASQKMEPR | 0.020610947 | 0 | 0 |
| 2642 | VDDTQFVRFDSDAASQR | 0 | 0.006310657 | 0 |
| 2643 | VDDTQFVRFDSDAASQRME | 0 | 0.004708229 | 0 |
| 2644 | VDDTQFVRFDSDAASQRMEPR | 0 | 0.002944061 | 0 |
| 2645 | VDDTQFVRFDSDAASQ | 0.07881329 | 0 | 0 |
| 2646 | VDDTQFLRFDSDAAIP | 0.002133308 | 0 | 0 |
| 2647 | VDDTQFLRFDSDAAIPR | 0.002213362 | 0 | 0 |
| 2648 | VDDTQFVRFD | 0.000470913 | 0 | 0 |
| 2649 | VDDTQFVRFDSD | 0.007333685 | 0 | 0 |
| 2650 | VDDTQFVRFDSDA | 0.002263069 | 0 | 0 |
| 2651 | VDDTQFVRFDSDAA | 0.002599243 | 6.10683E-05 | 0 |
| 2652 | VDDTQFVRFDSDAAS | 0.026875421 | 6.27991E-05 | 0 |
| 2653 | VDDTQFVRFDSDAASP | 0.503045459 | 0.000870518 | 5.87468E-05 |
| 2654 | YVDDTQFVRFDSDAASP | 0.00906235 | 0.000153593 | 0 |
| 2655 | YVDDTQFVRFDSDAASPR | 0.015341714 | 0 | 0.011795253 |
| 2656 | IAVGYVDDTQFVR | 0.015141135 | 0 | 0 |
| 2657 | IAVGYVDDTQFVRFD | 0.002538478 | 0 | 0.000217508 |
| 2658 | ISVGYVDDTQFVR | 0.08528777 | 0.000246257 | 0.000228698 |
| 2659 | ISVGYVDDTQFVRF | 0.006248755 | 0.000161279 | 0 |
| 2660 | ISVGYVDDTQFVRFD | 0.018794794 | 0 | 0 |
| 2661 | ISVGYVDDTQFVRFDSD | 0.002096607 | 0 | 0 |
| 2662 | ISVGYVDDTQFVRFDSDAASPR | 0.006089702 | 0 | 0 |
| 2663 | DSDAASQRMEPR | 0 | 0.000293417 | 0 |
| 2664 | DTQFVRFDSDAASQKMEPR | 0.002393535 | 0 | 0 |
| 2665 | FDSDAASQRMEPR | 7.80757E-05 | 0.003748467 | 6.02678E-06 |
| 2666 | FVRFDSDAASQRMEPR | 0 | 0.009133696 | 0.000179114 |
| 2667 | FVRFDSDAASQRM | 7.49998E-05 | 8.01439E-05 | 0 |
| 2668 | FVRFDSDAASQRME | 0 | 0.002938745 | 0 |
| 2669 | FVRFDSDAASQRMEP | 0 | 0.003432694 | 0 |
| 2670 | DTQFVRFDSDAASQK | 0.02806876 | 3.20464E-05 | 0.000143177 |
| 2671 | DTQFVRFDSDAASQKMEP | 0.003064725 | 0 | 0 |
| 2672 | FVRFDSDAASQK | 0.003572699 | 0 | 0 |
| 2673 | FVRFDSDAASQR | 0 | 0.003431798 | 0 |
| 2674 | TQFVRFDSDAASQK | 0.005694817 | 2.40818E-05 | 4.01213E-05 |
| 2675 | FDSDAASQRMEPRAP | 0 | 0.001289762 | 0 |
| 2676 | FDSDAASQRMEPRAPW | 0.000408168 | 0.008033014 | 0.00011939 |
| 2677 | FDSDAASQRMEPRAPWIE | 0 | 0.003516938 | 0 |
| 2678 | FVRFDSDATSPRKEPR | 0 | 0.001202262 | 0 |
| 2679 | FLRFDSDAAIPRMEP | 0 | 0.004336495 | 0 |
| 2680 | FLRFDSDAAIPRMEPR | 0 | 0.041073203 | 0 |
| 2681 | FLRFDSDAAIPRMEPREP | 0 | 0.000147702 | 0 |
| 2682 | QFLRFDSDAAIPRMEPR | 0 | 0.013891654 | 0 |
| 2683 | GSHSMRYFY | 0 | 0.000712492 | 0 |
| 2684 | DGIDDFVSGVPRAAR | 0 | 0 | 0.001242221 |
| 2685 | GIDDFVSGVPRAAR | 0 | 0 | 0.002123407 |
| 2686 | GIDDFVSGVPRAART | 0.000157551 | 0 | 0.003893307 |
| 2687 | GIDDFVSGVPRAARTLG | 0 | 5.33872E-05 | 0.003667835 |
| 2688 | NQDRLVTLEEFLASTQ | 0 | 0.000682647 | 0 |
| 2689 | QDRLVTLEEFLAS | 0 | 0.000113219 | 0 |
| 2690 | QDRLVTLEEFLAST | 0 | 0.000523727 | 0 |
| 2691 | QDRLVTLEEFLASTQ | 0.001896551 | 0.005120238 | 0.000124576 |
| 2692 | AAVDTSSEITTKD | 0 | 0.000288774 | 0 |
| 2693 | DLRSWTAVDTAAQ | 0.008853554 | 0 | 0 |
| 2694 | DLRSWTAVDTAAQIS | 0.026106622 | 9.05841E-05 | 0 |
| 2695 | LRSWTAVDTAAQ | 0.000109962 | 0 | 0 |
| 2696 | EDLRSWTAVDTAAQ | 0.007096928 | 0.001299028 | 4.65501E-05 |
| 2697 | LRSWTAVDTAAQIS | 0.001922091 | 0 | 0 |
| 2698 | WTAVDTAAQISEQ | 0 | 0.001353567 | 0 |
| 2699 | WTAVDTAAQISEQK | 0.0004709 | 0.008816189 | 0.003667138 |
| 2700 | DLLSINSAAELTYLK | 0 | 0.000218836 | 0.000388821 |
| 2701 | LLSINSAAELTYLK | 0 | 0.001126879 | 0 |
| 2702 | KPVDPTVEGGAQVQQVVN | 0 | 0 | 0.001813468 |
| 2703 | APSEAEPAADLIDMGP | 0.002865991 | 0 | 0 |
| 2704 | APSEAEPAADLIDMGPD | 0.015456707 | 0 | 3.3429E-05 |
| 2705 | APSEAEPAADLIDMGPDPA | 0.009295712 | 0 | 0.000497144 |
| 2706 | MEAPAQLLF | 0 | 0.002413142 | 0 |
| 2707 | RQTATQLLK | 0 | 4.1815E-05 | 0 |
| 2708 | DIHEGTSEVAQPQ | 0 | 0 | 0.000297683 |
| 2709 | GRVSIRDSPANLSFTVT | 0 | 0 | 0.00054042 |
| 2710 | DVNTATEVEPKDGEI | 0 | 0.001415628 | 0 |
| 2711 | GSGDVNTATEVEPKDG | 0 | 0.000350509 | 0 |
| 2712 | GQPREPQVY | 0 | 1.12755E-05 | 0.001700183 |
| 2713 | QPREPQVYTLPPSREE | 0 | 0.0004487 | 0 |
| 2714 | QPREPQVYTLPPSREEM | 0 | 0.001487091 | 0 |
| 2715 | QPREPQVYTLPPSREEMT | 0 | 0.004350978 | 0 |
| 2716 | IPTKQTQIFTTYSDNQPG | 0 | 0.021946028 | 8.0766E-05 |
| 2717 | IPTKQTQIFTTYSDNQPGVL | 0 | 0.001374758 | 0 |
| 2718 | IPTKQTQTFTTYSDNQP | 0 | 0.009491121 | 7.82131E-05 |
| 2719 | IPTKQTQTFTTYSDNQPG | 0 | 0.313374088 | 0 |
| 2720 | IPTKQTQTFTTYSDNQPGV | 0 | 0.02245411 | 0 |
| 2721 | IPTKQTQTFTTYSDNQPGVL | 0 | 0.055611436 | 0.000727379 |
| 2722 | KQTQTFTTYSDNQPG | 0 | 0.018112701 | 9.28833E-05 |
| 2723 | KQTQTFTTYSDNQPGV | 0 | 0.000978439 | 0 |
| 2724 | KQTQTFTTYSDNQPGVL | 0 | 0.001985742 | 0 |
| 2725 | QTQTFTTYSDNQPG | 0 | 0.012380333 | 0 |
| 2726 | AEIDARNDSF | 0 | 0.002499241 | 0 |
| 2727 | AERQLQVQW | 7.05922E-05 | 0.001145955 | 0 |
| 2728 | DGDGGEEDVQDDEEREL | 0.000587337 | 0.001962421 | 0 |
| 2729 | DGDGGEEDVQDDEERELQ | 0.000108587 | 0.002300414 | 0 |
| 2730 | DGGEEDVQDDEEREL | 0.003206215 | 0.006905411 | 0 |
| 2731 | DVQDDEEREL | 0.000223042 | 0.000377349 | 0.00010157 |
| 2732 | EEDVQDDEEREL | 0.000214537 | 0.002021645 | 0 |
| 2733 | GDGGEEDVQDDEEREL | 0.001999865 | 0.003076724 | 0 |
| 2734 | GEEDVQDDEEREL | 0.005490313 | 0.01628731 | 0 |
| 2735 | GGEEDVQDDEEREL | 0.002277613 | 0.00635922 | 0 |
| 2736 | DGGEEDVQDDEERELQ | 0.001245415 | 0.007653247 | 0 |
| 2737 | DGGEEDVQDDEERELQMDPA | 0.000195142 | 0.004552087 | 0 |
| 2738 | DVQDDEERELQ | 0.000370934 | 0.001764092 | 0 |
| 2739 | EDVQDDEERELQ | 1.86126E-05 | 0.000282592 | 0 |
| 2740 | EEDVQDDEERELQ | 0.000216209 | 0.002611815 | 0 |
| 2741 | GDGGEEDVQDDEERELQMDPA | 0.000291702 | 0.002306026 | 0 |
| 2742 | GEEDVQDDEERELQ | 0.004625592 | 0.02745072 | 0.000131977 |
| 2743 | GGEEDVQDDEERELQ | 0.001956081 | 0.006709994 | 0 |
| 2744 | VQDDEERELQ | 0 | 0.000740591 | 0.000158098 |
| 2745 | GDGGEEDVQDDEERELQ | 0.001768492 | 0.004268081 | 0 |
| 2746 | EEDVQDDEERELQMDP | 0 | 0.001390244 | 0 |
| 2747 | GEEDVQDDEERELQMDP | 0.000783981 | 0.013992326 | 0 |
| 2748 | GEEDVQDDEERELQMDPA | 0.000831033 | 0.01404961 | 0 |
| 2749 | GGEEDVQDDEERELQMDPA | 0.000798873 | 0.007764951 | 0 |
| 2750 | GEEDVQDDEERELQM | 0 | 0.000409696 | 0 |
| 2751 | GEEDVQDDEERELQMD | 0 | 0 | 0 |
| 2752 | EERTLEVEIEPGVR | 0 | 0.005002211 | 9.66814E-05 |
| 2753 | LVNEERTLEVEIEPGVR | 0.000311511 | 0.006075749 | 8.84714E-05 |
| 2754 | NEERTLEVEIEPGVR | 0 | 0.000406107 | 0 |
| 2755 | VNEERTLEVEIEPGVR | 0 | 0.002470958 | 0 |
| 2756 | VNEERTLEVEIEPG | 0 | 0.000748195 | 0 |
| 2757 | IDIEPTDKVERIK | 0 | 0.000922953 | 0 |
| 2758 | IDIEPTDKVERIKER | 0 | 0.000913928 | 8.82618E-05 |
| 2759 | KEIEIDIEPTDK | 0.001065509 | 0 | 0.000138435 |
| 2760 | TGKEIEIDIEPTDK | 0.0003243 | 0 | 0 |
| 2761 | NVELNAGQIFTIL | 0 | 0 | 0.001060244 |
| 2762 | DPQSGALYISK | 0 | 0 | 0.007607029 |
| 2763 | LDPQSGALYISK | 0 | 6.53373E-05 | 0.005649656 |
| 2764 | TPDTGGVFIAGRVN | 0.000142143 | 0 | 0.002247328 |
| 2765 | DVEAQAATCNHT | 0 | 0 | 0.000713294 |
| 2766 | DVEAQAATCNHTV | 0.000242464 | 0 | 0.001006499 |
| 2767 | NIETEAAAVDK | 0 | 0.001286909 | 0 |
| 2768 | TNLNIETEAAAVDKLN | 0 | 0.00056304 | 0 |
| 2769 | RMNPNSPSITY | 2.22972E-05 | 0 | 0.002320842 |
| 2770 | EIESETTSEEQIQEEK | 0 | 0.000477833 | 0 |
| 2771 | IESETTSEEQIQEEK | 0 | 0.000429461 | 0 |
| 2772 | RLYLNGDGTGK | 0 | 0.000195381 | 0 |
| 2773 | YVNTDSGPELR | 0 | 0.000366543 | 0 |
| 2774 | IQPEGGTIIVTQN | 9.39379E-05 | 0.000666747 | 0.075730617 |
| 2775 | IQPEGGTIIVTQNR | 0 | 0 | 0.018553299 |
| 2776 | IQPEGGTIIVTQNRN | 0 | 0 | 0.063225926 |
| 2777 | IQPEGGTIIVTQNRNR | 0 | 0 | 0.00178719 |
| 2778 | IQPEGGTIIVTQNRNRE | 0 | 0 | 0.001292217 |
| 2779 | QPEGGTIIVTQNRN | 0 | 0 | 0.000707725 |
| 2780 | TIQPEGGTIIVTQNRN | 0 | 0 | 0.00039017 |
| 2781 | VTIQPEGGTIIVTQNRN | 0 | 0 | 0.005061134 |
| 2782 | VTIQPEGGTIIVTQN | 0 | 0.000199229 | 0.010372753 |
| 2783 | VTIQPEGGTIIVTQNR | 0 | 0 | 0.000683144 |
| 2784 | TPVSTIIMPNE | 0 | 0 | 0 |
| 2785 | SPGVVISDDEPGYD | 0.000159216 | 0.006499197 | 0 |
| 2786 | SPGVVISDDEPGYDL | 0.000188536 | 0.003027734 | 0 |
| 2787 | SPGVVISDDEPGYDLD | 0 | 0.002664264 | 0 |
| 2788 | IRRSSMSVEAETTTT | 0 | 0.00038878 | 0 |
| 2789 | DDFRWALSQSNPSA | 0 | 0 | 0.001776159 |
| 2790 | DDFRWALSQSNPSALR | 0.000218131 | 0 | 0.003092736 |
| 2791 | SPEKYVTSAPMPEPQ | 6.13917E-05 | 0.000956418 | 0.000103084 |
| 2792 | SPEKYVTSAPMPEPQAP | 0 | 0.00143286 | 0.000233008 |
| 2793 | SPEKYVTSAPMPEPQAPG | 0 | 0.031198896 | 0 |
| 2794 | EEGTPKESEPQAAAEPA | 3.02436E-05 | 0 | 0.005887515 |
| 2795 | EEGTPKESEPQAAAEPAE | 0 | 0 | 0.00189494 |
| 2796 | EGTPKESEPQAAAEPA | 0 | 0 | 0.001720516 |
| 2797 | NRSEIETQTEGSTE | 0 | 0.00021843 | 8.48644E-05 |
| 2798 | NRSEIETQTEGSTEK | 0 | 0.000314449 | 0 |
| 2799 | RSEIETQTEGSTE | 0 | 0.000379543 | 6.83092E-05 |
| 2800 | SNRSEIETQTEGSTEKE | 0 | 0.0004373 | 0 |
| 2801 | RSEIETQTEGSTEK | 0 | 0.000186746 | 0 |
| 2802 | GVIEPDTDAPQEM | 0 | 0.002230296 | 0 |
| 2803 | GVIEPDTDAPQEMG | 0.000647296 | 0.004532068 | 0.002104233 |
| 2804 | GVIEPDTDAPQEMGDE | 0 | 0.003131423 | 0 |
| 2805 | VIEPDTDAPQEM | 0 | 0.022019068 | 0 |
| 2806 | VIEPDTDAPQEMG | 0 | 0.06328987 | 0 |
| 2807 | VIEPDTDAPQEMGDE | 0 | 0.053019464 | 0.002761756 |
| 2808 | VIEPDTDAPQEMGDEN | 0 | 0.00264066 | 0 |
| 2809 | RETTDTDTADQVIA | 0 | 0.001729946 | 0 |
| 2810 | TPQTDTSDDLDRPS | 0 | 0.000170357 | 0 |
| 2811 | YTPQTDTSDDLDRPS | 0 | 0.000174472 | 0 |
| 2812 | SQLIMQAEAEAASVR | 0 | 0 | 0.000914479 |
| 2813 | NPDSNSANCLEEKG | 0 | 0.000966378 | 0 |
| 2814 | LPEPETASRCNPAASD | 0 | 6.56777E-05 | 0.004118022 |
| 2815 | LPEPETASRCNPAASDHD | 0 | 0 | 0.000819557 |
| 2816 | AELTGVTVESPEEPQR | 0 | 0.000392422 | 0 |
| 2817 | IVKQITSISIEPGVE | 6.25226E-05 | 0.003242677 | 0 |
| 2818 | EGPSSLDPSQEGP | 0.00590134 | 0.026905948 | 0 |
| 2819 | EGPSSLDPSQEGPT | 0.000833801 | 0.015144194 | 0 |
| 2820 | GPSSLDPSQEGP | 0.000148533 | 0.027519074 | 0 |
| 2821 | MPESEGPSSLDPSQEGPT | 0 | 0.010696907 | 0 |
| 2822 | SEGPSSLDPSQEGPT | 0 | 0 | 0 |
| 2823 | MPESEGPSSLDPSQEGP | 0 | 0.002412816 | 0 |
| 2824 | SEGPSSLDPSQEGP | 0.00021902 | 0.006437481 | 0 |
| 2825 | GPSSLDPSQEGPT | 0 | 0.008417584 | 0 |
| 2826 | GPSSLDPSQEGPTG | 0 | 0 | 0 |
| 2827 | EPAEGPEEAAAGRSHVVQ | 0 | 0 | 0.001722852 |
| 2828 | GPEEAAAGRSHVVQ | 0 | 0 | 0 |
| 2829 | IKGKINSITVDN | 5.39216E-05 | 0.001631831 | 0 |
| 2830 | KGKINSITVDN | 2.1584E-05 | 0.001592845 | 0.000412795 |
| 2831 | QIKGKINSITVDN | 0 | 0.001519463 | 6.99891E-05 |
| 2832 | RVPFHRVDTISVN | 0 | 0.000209568 | 0 |
| 2833 | VPFHRVDTISVN | 0 | 0.007347741 | 0 |
| 2834 | VPFHRVDTISVNGSVQ | 0 | 0.014249013 | 9.73829E-05 |
| 2835 | DFTLTISRLEPEDFAVY | 0.000595672 | 0 | 0 |
| 2836 | FTLTISRLEPEDF | 0.014073954 | 0 | 3.27088E-05 |
| 2837 | FTLTISRLEPEDFA | 0.007147252 | 0 | 0 |
| 2838 | FTLTISRLEPEDFAV | 0.012217856 | 0 | 5.11174E-05 |
| 2839 | FTLTISRLEPEDFAVY | 0.014392908 | 0 | 0.000346901 |
| 2840 | TISRLEPEDF | 0.001234041 | 7.4487E-06 | 9.52492E-05 |
| 2841 | TLTISRLEPEDF | 0.006353159 | 0 | 0 |
| 2842 | TISRLEPEDFAV | 0.001270976 | 0.00034713 | 0 |
| 2843 | TLTISRLEPEDFAV | 0.011233227 | 0 | 5.11218E-05 |
| 2844 | TLTISRLEPEDFAVY | 0.002298328 | 0 | 0 |
| 2845 | FTLKISRVEAEDVGVY | 0 | 0.00097315 | 0 |
| 2846 | TLKISRVEAEDVGVY | 0 | 0.005972342 | 0.000253939 |
| 2847 | ISRVEAGDEADYY | 0.002223182 | 0 | 0 |
| 2848 | TLTISRVEAGDEADY | 0 | 0.008829649 | 0 |
| 2849 | VDATEESDLAQQYGVR | 0 | 0 | 0 |
| 2850 | GALQIESSEETDQGKY | 0 | 0.000674874 | 0.004978917 |
| 2851 | AGGHKLGLGLEFQA | 0.00026899 | 0.002288802 | 0.000188294 |
| 2852 | GKNVNAGGHKLGLGLEFQA | 0 | 0.000516439 | 0 |
| 2853 | AGGHKVGLALELEA | 8.34689E-05 | 0.000678254 | 0 |
| 2854 | AHSILTVSEEEWN | 0 | 0 | 0 |
| 2855 | AHSILTVSEEEWNTG | 0 | 0.012695099 | 0 |
| 2856 | APGRYFAHSILTVSEEEWNTG | 0.00451139 | 0.004353433 | 0 |
| 2857 | FAHSILTVSEEEWNTG | 0.005426355 | 0.001208313 | 0 |
| 2858 | APGRYFAHSILTVSEEEWN | 0 | 0.000642115 | 0 |
| 2859 | FAHSILTVSEEEWN | 0.001167668 | 0.000428036 | 0 |
| 2860 | GIVTIGEEK | 0 | 0.000430835 | 0 |
| 2861 | GIVTIGEEKK | 1.85946E-05 | 0.000168203 | 0 |
| 2862 | GIVTIGEEKKI | 0 | 0.003007403 | 0 |
| 2863 | QFGIVTIGEEKKI | 0 | 0.006008145 | 0 |
| 2864 | AIATGKMLSLAEQQL | 0 | 0.002405084 | 0 |
| 2865 | AIATGKMLSLAEQQLVD | 0 | 0.000818194 | 0 |
| 2866 | ATGKMLSLAEQQL | 0.004261447 | 0.005173958 | 0 |
| 2867 | GKMLSLAEQQL | 0 | 0.002191189 | 0 |
| 2868 | IATGKMLSLAEQQL | 0.016236075 | 0.045687179 | 0 |
| 2869 | KMLSLAEQQL | 0 | 0.001040379 | 0 |
| 2870 | TGKMLSLAEQQL | 0 | 0.004561504 | 5.14219E-05 |
| 2871 | IATGKMLSLAEQQLV | 0.000265174 | 0.00281101 | 0 |
| 2872 | ATGKMLSLAEQQLVD | 0.008866318 | 0.00303856 | 0 |
| 2873 | IATGKMLSLAEQQLVD | 0.017857758 | 0.022629548 | 0.004054794 |
| 2874 | KMLSLAEQQLVD | 0 | 0.000740409 | 0 |
| 2875 | TGKMLSLAEQQLVD | 0 | 0.004225358 | 0 |
| 2876 | IAIATGKMLSLAEQ | 0 | 0.000606209 | 0 |
| 2877 | IAIATGKMLSLAEQQL | 0 | 0.000642871 | 0 |
| 2878 | ESAGARVVPVR | 0 | 0 | 0.004343141 |
| 2879 | KYLESAGARVVPVR | 0 | 0 | 0.014649978 |
| 2880 | LESAGARVVPVR | 0 | 0 | 0.003566319 |
| 2881 | SAGARVVPVR | 0 | 0 | 0.001914846 |
| 2882 | VKYLESAGARVVPVR | 0 | 0 | 0.000435972 |
| 2883 | YLESAGARVVPVR | 0 | 0 | 0.035157747 |
| 2884 | YLESAGARVVPVRLD | 0 | 0.000627582 | 0.003009744 |
| 2885 | SPGPRLLAVAGALGLLASGLQ | 0 | 0 | 0.000744669 |
| 2886 | PLLVGTGVALVTGATV | 0 | 0 | 0.000127895 |
| 2887 | VPLLVGTGVALVTG | 0 | 0 | 0 |
| 2888 | VPLLVGTGVALVTGAT | 0 | 0 | 0.003912074 |
| 2889 | KLVSLSAQNLVD | 0.000212706 | 0.001504318 | 0 |
| 2890 | LKTGKLVSLSAQNLVD | 0 | 0.000922203 | 0 |
| 2891 | TGKLVSLSAQNLVD | 0 | 0.001769835 | 0 |
| 2892 | TGKLVSLSAQNL | 0 | 0.001687284 | 0 |
| 2893 | LPIGATVNMD | 0 | 8.27239E-05 | 0.001225048 |
| 2894 | LPIGATVNMDG | 0 | 0 | 0.002030242 |
| 2895 | LPIGATVNMDGAA | 0 | 4.46256E-05 | 0.010929258 |
| 2896 | LPIGATVNMDGAALFQ | 0.009708322 | 0 | 4.85098E-05 |
| 2897 | RFILPIGATVNMDG | 0 | 0 | 0.000719151 |
| 2898 | LPQLVGVSTPLQGGSN | 0 | 0 | 0.000592725 |
| 2899 | DVAVVAGGLGRQL | 0 | 0 | 0.002154755 |
| 2900 | DVAVVAGGLGRQLL | 0 | 0 | 0.012500338 |
| 2901 | VAVVAGGLGRQLL | 0 | 0 | 0.001795417 |
| 2902 | VYVLAGAVAR | 0 | 0 | 0.001079095 |
| 2903 | DQLSTHTDGIPH | 0 | 0.008051234 | 0 |
| 2904 | DQLSTHTDGIPHL | 0 | 0.014908882 | 0 |
| 2905 | DQLSTHTDGIPHLV | 5.46664E-05 | 0.033506721 | 5.6601E-05 |
| 2906 | GDQLSTHTDGIPHLV | 0 | 0.011811553 | 0.001167481 |
| 2907 | QGDQLSTHTDGIPHLV | 0 | 0.001236331 | 0 |
| 2908 | LSTHTDGIPHL | 0 | 0.000110986 | 0 |
| 2909 | GDQLSTHTDGIPH | 0 | 0.032055661 | 0 |
| 2910 | GDQLSTHTDGIPHL | 0 | 0.007886061 | 0.002445637 |
| 2911 | LTQGDQLSTHTDGIPH | 0 | 0.007299491 | 4.35503E-05 |
| 2912 | QGDQLSTHTDGIPH | 7.30768E-05 | 0.007798265 | 0 |
| 2913 | TQGDQLSTHTDGIPH | 0 | 0.010786806 | 0 |
| 2914 | STHTDGIPHLVL | 0 | 0.001889691 | 0 |
| 2915 | ENSPAGRAQPVP | 0 | 4.00475E-06 | 0.002304509 |
| 2916 | SPAGRAQPVP | 0 | 0 | 0.006035658 |
| 2917 | DSQETAVAIASR | 0 | 0.000497349 | 0 |
| 2918 | DSQETAVAIASRLG | 0 | 0 | 0.005831927 |
| 2919 | GDSQETAVAIASR | 7.57691E-05 | 0.000513726 | 0 |
| 2920 | ITGDSQETAVAIASR | 0 | 0.002753762 | 0.001497479 |
| 2921 | TGDSQETAVAIASR | 0 | 0.002699094 | 0.000494529 |
| 2922 | IPDNVDAALALPAH | 0 | 0 | 0.010802083 |
| 2923 | EPTVTISPSRTEALN | 0.000361759 | 0 | 0.009547579 |
| 2924 | EPTVTISPSRTEALNHHN | 0 | 0 | 0.000839332 |
| 2925 | RRVEPTVTISPSRT | 0 | 0.000671403 | 0 |
| 2926 | RVEPTVTISPSRT | 0 | 3.93915E-05 | 0 |
| 2927 | VEPTVTISPSRT | 0 | 0.001110545 | 0 |
| 2928 | VEPTVTISPSRTE | 0 | 0.000465745 | 0 |
| 2929 | VEPTVTISPSRTEA | 0 | 0.003240894 | 0 |
| 2930 | VEPTVTISPSRTEALN | 0 | 0 | 0.002811997 |
| 2931 | GALESAIAIATGK | 5.35588E-05 | 0 | 0.00597293 |
| 2932 | GALESAIAIATGKM | 0 | 6.17737E-05 | 0.035179532 |
| 2933 | GALESAIAIATGKML | 0 | 0 | 0.003236847 |
| 2934 | TGALESAIAIATGKML | 0 | 0 | 0.031766907 |
| 2935 | TGALESAIAIATGKMLS | 0 | 0 | 0 |
| 2936 | STTGALESAIAIATGKM | 0 | 0.002093935 | 0.001482117 |
| 2937 | TGALESAIAIATG | 0 | 0.000120362 | 0.009669806 |
| 2938 | TGALESAIAIATGK | 0 | 0.000193021 | 0.02962025 |
| 2939 | TGALESAIAIATGKM | 0.00043662 | 0.003667539 | 0.293019794 |
| 2940 | TTGALESAIAIATGKM | 0 | 0.000462983 | 0.013220426 |
| 2941 | TTGALESAIAIATGK | 0 | 0 | 0.00147161 |
| 2942 | TTGALESAIAIATGKML | 0 | 0 | 0.005872595 |
| 2943 | TVAVAAGRSWVAIPR | 0 | 0 | 0.020202158 |
| 2944 | TVAVAAGRSWVAIPREQ | 0 | 0 | 0.001017025 |
| 2945 | TQKSLSLSPGK | 0.000427446 | 0 | 0.000569212 |
| 2946 | APDGLAVDWIHSN | 0.001521714 | 0 | 0 |
| 2947 | DIQAPDGLAVDWIHSN | 0.019849241 | 0 | 0 |
| 2948 | KPETVPVDFQ | 0 | 0.000289915 | 9.83438E-05 |
| 2949 | GPFRSNSISLDNL | 0 | 0.000173426 | 0 |
| 2950 | GPFRSNSISLDNLK | 0 | 0 | 0 |
| 2951 | GPFRSNSISLDNLKPS | 0.000915163 | 0.005301368 | 0.000716558 |
| 2952 | DPSGTYHAWKANAIGRG | 5.49572E-05 | 0.002096193 | 0 |
| 2953 | SASYKADTVAKVQ | 0.000625984 | 0 | 0.00030215 |
| 2954 | DPTGTYHGDSDLQLDR | 0.007479009 | 0.001102214 | 0 |
| 2955 | DPTGTYHGDSDLQLDRI | 0.001714855 | 0 | 0 |
| 2956 | DPTGTYHGDSDLQLER | 0.000683577 | 0 | 3.47842E-05 |
| 2957 | IDPTGTYHGDSDLQLDRIS | 0.00011012 | 0.001422255 | 0 |
| 2958 | IDPTGTYHGDSD | 0.000328247 | 0 | 0 |
| 2959 | IDPTGTYHGDSDLQLD | 0.000935589 | 0.001727716 | 0 |
| 2960 | IDPTGTYHGDSDLQLDR | 0.004111017 | 0.004074135 | 0 |
| 2961 | IDPTGTYHGDSDLQLE | 0 | 0.000361963 | 0 |
| 2962 | RIIGLKPEGVPRID | 0.001168091 | 0 | 0 |
| 2963 | QPHSTIKTEAVAS | 0 | 2.45369E-05 | 0 |
| 2964 | TPPPGVHSQTLSRF | 0 | 0.003844173 | 0.000106228 |
| 2965 | TPPPGVHSQTLSRFF | 0 | 0.000859523 | 0 |
| 2966 | TPPPGVHSQTLSRFFDK | 0 | 0.010036145 | 0 |
| 2967 | DIDSGQGIRN | 0 | 0 | 0.00014786 |
| 2968 | LHQDIDSGQGIRN | 0 | 0 | 0.003628616 |
| 2969 | HWDTTQSLKQ | 0 | 0.000588873 | 0 |
| 2970 | LLLWHWDTTQSLKQ | 0.010743865 | 0.000140146 | 0.001461335 |
| 2971 | LLWHWDTTQSLKQ | 0.017029725 | 0.009165094 | 0.001493059 |
| 2972 | LWHWDTTQSLKQ | 0.002610844 | 0.017710631 | 0.001614383 |
| 2973 | WHWDTTQSLKQ | 6.51407E-05 | 0.016471484 | 0 |
| 2974 | LWHWDTTQSLK | 0.00096659 | 0 | 0.000645262 |
| 2975 | LWHWDTTQSLKQLE | 0 | 0.003304329 | 0 |
| 2976 | WHWDTTQSLKQLE | 0 | 0.001193684 | 0 |
| 2977 | LLLWHWDTTQSLK | 0.006594416 | 0 | 0.001218806 |
| 2978 | LLWHWDTTQSL | 0.00424709 | 0 | 0 |
| 2979 | LLWHWDTTQSLK | 0.011413836 | 0 | 0.002156353 |
| 2980 | FVKTLTGKTITLEVEPSD | 0 | 0.004164769 | 0 |
| 2981 | FVKTLTGKTITLEVEPSDT | 0 | 0.039658694 | 0 |
| 2982 | FVKTLTGKTITLEVEPSDTI | 0 | 0.003074174 | 0 |
| 2983 | FVKTLTGKTITLEVEPSDTIEN | 0 | 0.003561089 | 0 |
| 2984 | GKTITLEVEPSD | 0.002148194 | 0.002300334 | 4.73523E-05 |
| 2985 | KTITLEVEPSD | 0 | 0.003822528 | 0 |
| 2986 | KTLTGKTITLEVEPSD | 0.000297331 | 0.121658463 | 0 |
| 2987 | LTGKTITLEVEPSD | 0.003358324 | 0.051417329 | 0.000405194 |
| 2988 | TGKTITLEVEPSD | 0.018809728 | 0.034216669 | 0 |
| 2989 | TLTGKTITLEVEPSD | 0.00425315 | 0.073087771 | 0 |
| 2990 | VKTLTGKTITLEVEPSD | 0.001136023 | 0.156574025 | 0 |
| 2991 | KTLTGKTITLEVEP | 0 | 0.008594913 | 6.03973E-05 |
| 2992 | KTLTGKTITLEVEPS | 0 | 0.163224432 | 0 |
| 2993 | KTLTGKTITLEVEPSDT | 0.001214634 | 0.442415585 | 3.87138E-05 |
| 2994 | KTLTGKTITLEVEPSDTI | 0 | 0.036259219 | 0 |
| 2995 | TLTGKTITLEVEP | 0 | 0.003296756 | 0 |
| 2996 | VKTLTGKTITLEVEPSDTI | 0.000268389 | 0.047207693 | 0 |
| 2997 | TLTGKTITLEVEPS | 0 | 0.178179949 | 0 |
| 2998 | TLTGKTITLEVEPSDT | 0.00846316 | 0.162296495 | 0 |
| 2999 | VKTLTGKTITLEVEPSDT | 0.004058459 | 0.829375913 | 0 |
| 3000 | GKTITLEVEPSDT | 0.000662762 | 0.018832003 | 0 |
| 3001 | GKTITLEVEPSDTI | 0.004406566 | 0.007115778 | 0 |
| 3002 | GKTITLEVEPSDTIEN | 0.001133723 | 0.000380217 | 0 |
| 3003 | KTITLEVEPSDT | 0.004270985 | 0.015140011 | 0.000233902 |
| 3004 | KTLTGKTITLEVEPSDTIEN | 0.000114235 | 0.008229546 | 0 |
| 3005 | LTGKTITLEVEPSDTIEN | 0.001464047 | 0.006511077 | 0 |
| 3006 | TGKTITLEVEPSDTIEN | 0.007767128 | 0.002599284 | 0.000289824 |
| 3007 | TLTGKTITLEVEPSDTIEN | 0.002835648 | 0.006364916 | 0 |
| 3008 | VKTLTGKTITLEVEPSDTIEN | 0.001341703 | 0.020387217 | 0 |
| 3009 | TGKTITLEVEPSDT | 0.04047695 | 0.135740677 | 0 |
| 3010 | TGKTITLEVEPSDTI | 0.008810284 | 0.061399551 | 0 |
| 3011 | LTGKTITLEVEPS | 0 | 0.018155619 | 0 |
| 3012 | LTGKTITLEVEPSDT | 0.014060891 | 0.168137958 | 0 |
| 3013 | LTGKTITLEVEPSDTI | 0 | 0.179729659 | 0 |
| 3014 | TGKTITLEVEPS | 0.000120116 | 0.00908882 | 6.32788E-05 |
| 3015 | TLTGKTITLEVEPSDTI | 0.000303775 | 0.061910427 | 0 |
| 3016 | KTITLEVEPSDTI | 0 | 0.01133876 | 0.000156089 |
| 3017 | KTITLEVEPSDTIE | 0.003921361 | 0.000579644 | 0.002020808 |
| 3018 | KTLTGKTITLEVEPSDTIE | 0.002217846 | 0.009330671 | 0 |
| 3019 | LTGKTITLEVEPSDTIE | 0.020613603 | 0.011998055 | 0 |
| 3020 | TGKTITLEVEPSDTIE | 0.059247973 | 0.00390535 | 0.000167578 |
| 3021 | TLTGKTITLEVEPSDTIE | 0.015212645 | 0.010871444 | 0 |
| 3022 | VKTLTGKTITLEVEPSDTIE | 0.000799435 | 0.010138663 | 0 |
| 3023 | VKTLTGKTITLEVEP | 0 | 0.01138974 | 0.000610311 |
| 3024 | VKTLTGKTITLEVEPS | 0 | 0.069184613 | 3.80993E-05 |
| 3025 | IFVKTLTG | 0 | 0.000740702 | 0 |
| 3026 | IFVKTLTGKTI | 0 | 0.001946485 | 0 |
| 3027 | IFVKTLTGKTIT | 0 | 0.029350162 | 0 |
| 3028 | IFVKTLTGKTITL | 0 | 0.031774728 | 0 |
| 3029 | IFVKTLTGKTITLE | 0 | 0.020179636 | 0 |
| 3030 | QIFVKTLTGK | 0 | 0.001749214 | 0 |
| 3031 | QIFVKTLTGKTITL | 0 | 0.001126274 | 0 |
| 3032 | WSQWLGGSSWPGYVRPLPP | 0 | 0 | 0.000358612 |
| 3033 | EIIVQNGKVIGVK | 0 | 0 | 0.000693918 |
| 3034 | EIIVQNGKVIGVKSEG | 0 | 0 | 0.002671073 |
| 3035 | DPYGVAVGGTVGHC | 0 | 0 | 0.001777837 |
| 3036 | DPYGVAVGGTVGHCL | 0 | 0.000131772 | 0.078400667 |
| 3037 | DPYGVAVGGTVGHCLCTG | 0 | 6.70574E-05 | 0.004486977 |
| 3038 | EDPYGVAVGGTVGHCL | 0 | 0 | 0.001372155 |
| 3039 | VGISSAVPFLIL | 0 | 0 | 0.000276447 |
| 3040 | EVTHQGLSSPVT | 0 | 0 | 0.007692469 |
| 3041 | EVTHQGLSSPVTK | 7.81492E-06 | 0 | 0.000692605 |
| 3042 | KHKVYACEVTHQGLSSP | 0 | 0 | 0.000494813 |
| 3043 | AAEEPNAAVPDEIPP | 0.003561122 | 0.001583633 | 0.000107556 |
| 3044 | AAEEPNAAVPDEIPPL | 0.009551862 | 0.006980999 | 0.004366665 |
| 3045 | AESGRGQVMSSLAE | 6.62321E-06 | 0 | 0.000624294 |
| 3046 | NAESGRGQVMSSLAE | 0 | 9.6225E-05 | 0.002393808 |
| 3047 | AIETIVAAMSNLVPPV | 0 | 0 | 0.000953467 |
| 3048 | IETIVAAMSNLVPP | 0 | 0 | 0.001435773 |
| 3049 | IETIVAAMSNLVPPV | 0 | 0 | 0.009157269 |
| 3050 | IVAAMSNLVPPV | 0 | 0 | 0.000265755 |
| 3051 | TIVAAMSNLVPPV | 0 | 0 | 0.001339166 |
| 3052 | GRGQVMSSLAELEDDF | 9.46602E-05 | 0.000898614 | 0.000541273 |
| 3053 | RGQVMSSLAELEDDF | 0.000112648 | 0.000141082 | 0.000404369 |
| 3054 | RGQVMSSLAELEDDFK | 0 | 0.00057559 | 0.000517855 |
| 3055 | GSGSHTLPALLENLK | 0 | 0.00026484 | 0 |
| 3056 | HLVSTTKTEVIPP | 0 | 0.001701319 | 0 |
| 3057 | HLVSTTKTEVIPPLIE | 0.000110351 | 0.004008108 | 0 |
| 3058 | LVSTTKTEVIP | 0 | 0.00350134 | 0 |
| 3059 | LVSTTKTEVIPP | 0 | 0.02579094 | 0 |
| 3060 | TLHLVSTTKTEVIPPLIE | 0.002118595 | 0.007119357 | 0 |
| 3061 | VSTTKTEVIP | 0 | 0.001331092 | 0 |
| 3062 | LHLVSTTKTEVIP | 0 | 0.000900615 | 0 |
| 3063 | LHLVSTTKTEVIPP | 0.000130257 | 0.01767678 | 0 |
| 3064 | LHLVSTTKTEVIPPL | 3.91809E-05 | 0.00459162 | 0 |
| 3065 | TLHLVSTTKTEVIPPL | 0.000253085 | 0.001882807 | 0 |
| 3066 | LVSTTKTEVIPPL | 0 | 0.004578482 | 0 |
| 3067 | LVSTTKTEVIPPLI | 0 | 0.000580365 | 0 |
| 3068 | LVSTTKTEVIPPLIE | 0 | 0.010283478 | 0 |
| 3069 | VSTTKTEVIPP | 0 | 0.003521293 | 0 |
| 3070 | NTLHLVSTTKTEVIPP | 0.001784242 | 0.008393182 | 0 |
| 3071 | TLHLVSTTKTEVIPP | 0.007143662 | 0.017340498 | 3.89769E-05 |
| 3072 | VSTTKTEVIPPLIE | 0 | 0.012034122 | 0 |
| 3073 | VSTTKTEVIPPLIEN | 0 | 0.000566054 | 0 |
| 3074 | MLPSILNQL | 0 | 0 | 0.005536845 |
| 3075 | TEMLPSILNQL | 0 | 0.002700388 | 0 |
| 3076 | FAGSRSQLVQ | 0 | 0 | 0.000312306 |
| 3077 | KKLLFAGSRSQLVQ | 0 | 0 | 0.002409897 |
| 3078 | DLGTDMVPAISL | 0 | 0.023887553 | 0 |
| 3079 | IDLGTDMVPAISL | 0 | 0.003415096 | 1.61698E-05 |
| 3080 | IDLGTDMVPAIS | 0 | 0.003178458 | 0 |
| 3081 | VPAQLVGGVGQDGVAE | 0 | 0.002947891 | 0.00801102 |
| 3082 | LPVFHPVTGEIIP | 0.00164932 | 7.20176E-05 | 0 |
| 3083 | LPVFHPVTGEIIPP | 0.003699319 | 0 | 0 |
| 3084 | LPVFHPVTGEIIPPL | 0.004314499 | 0.002823918 | 0 |
| 3085 | LPVFHPVTGEIIPPLHT | 0.00259828 | 0.000880683 | 0 |
| 3086 | ESEVVAGIPRTD | 0 | 0 | 0 |
| 3087 | IESEVVAGIPRTD | 0 | 0 | 0.03360842 |
| 3088 | IESEVVAGIPRTDQ | 0 | 0 | 0.002211957 |
| 3089 | IESEVVAGIPR | 0 | 0.001143976 | 0.012767933 |
| 3090 | DNPGKVEHTEELSSITE | 0 | 0.001487657 | 0 |
| 3091 | NPGKVEHTEELSSITE | 1.63919E-05 | 0.003427438 | 0 |
| 3092 | HTEELSSITEVVT | 0 | 0.006066676 | 0.000767087 |
| 3093 | ALANIAVDKANLE | 0.00602337 | 5.45175E-05 | 0 |
| 3094 | ALANIAVDKANLEI | 0.004622682 | 0.000158165 | 0 |
| 3095 | ALANIAVDKANLEIM | 0.011351786 | 0.000374515 | 0 |
| 3096 | ALANIAVDKANLEIMT | 0.024000619 | 0.001304129 | 0 |
| 3097 | GALANIAVDKANLEIMT | 0.00475303 | 0.000111781 | 0 |
| 3098 | LANIAVDKANLE | 0.000803107 | 0 | 7.16652E-05 |
| 3099 | LANIAVDKANLEI | 0.023131211 | 0.000837022 | 0 |
| 3100 | LANIAVDKANLEIM | 0.06555969 | 0.001259042 | 0 |
| 3101 | ANIAVDKANLEIM | 0.012975908 | 0.000209143 | 0.000326649 |
| 3102 | ANIAVDKANLEIMT | 0.008929428 | 0 | 0.00027444 |
| 3103 | LANIAVDKANLEIMT | 0.06280314 | 0.001077925 | 2.30688E-05 |
| 3104 | LANIAVDKANLEIMTK | 0.007883273 | 0 | 0 |
| 3105 | NIAVDKANLEIM | 0.003360695 | 0.000354353 | 0 |
| 3106 | NIAVDKANLEIMT | 0.000269732 | 0.000128415 | 0 |
| 3107 | GALANIAVDK | 0 | 0 | 0.00063249 |
| 3108 | GALANIAVDKANLEIM | 0.001966715 | 3.45006E-05 | 0 |
| 3109 | AQGALANIAVD | 0 | 0 | 0.001004636 |
| 3110 | AQGALANIAVDK | 1.54578E-05 | 0 | 0.042362645 |
| 3111 | AQGALANIAVDKA | 0.002434412 | 0.000121332 | 0.154940367 |
| 3112 | AQGALANIAVDKAN | 0.006570676 | 0.000826697 | 0.045267 |
| 3113 | AQGALANIAVDKANLE | 0.00574597 | 0.001788946 | 0.001783812 |
| 3114 | ASFEAQGALANIAVDKANLE | 0 | 0 | 0.035462276 |
| 3115 | EAQGALANIAVDKANLE | 0 | 0 | 0.001082417 |
| 3116 | EFGRFASFEAQGALANIAVDKANLE | 0 | 0 | 0.003852827 |
| 3117 | RFASFEAQGALANIAVDKANLE | 0 | 0 | 0.003432256 |
| 3118 | SFEAQGALANIAVDKANLE | 0 | 0 | 0.002837877 |
| 3119 | QGALANIAVDK | 3.22268E-05 | 0.000306942 | 0.021368775 |
| 3120 | QGALANIAVDKA | 0.000204109 | 0 | 0.034198471 |
| 3121 | QGALANIAVDKAN | 0.000915519 | 0 | 0.002682031 |
| 3122 | QGALANIAVDKANLE | 0.000289716 | 0.002301605 | 0 |
| 3123 | EAQGALANIA | 0 | 0 | 0.000678933 |
| 3124 | EAQGALANIAVD | 0 | 4.29245E-05 | 0.040440115 |
| 3125 | EAQGALANIAVDK | 0 | 0 | 0.481749046 |
| 3126 | EAQGALANIAVDKA | 0.000539283 | 0.000689152 | 0.493928889 |
| 3127 | EAQGALANIAVDKAN | 0.001131281 | 0 | 0.105873809 |
| 3128 | EAQGGLANIAI | 0 | 0 | 0.000347532 |
| 3129 | FEAQGALANIAVDKAN | 0.005526726 | 0.002486737 | 0.04287528 |
| 3130 | SFEAQGALANIAVDKAN | 0 | 0 | 0.095391342 |
| 3131 | FEAQGALANIAVD | 0 | 0 | 0.009424956 |
| 3132 | FEAQGALANIAVDK | 0 | 0.000395135 | 0.172676704 |
| 3133 | FEAQGALANIAVDKA | 0.00048482 | 5.28377E-05 | 0.190680637 |
| 3134 | SFEAQGALANIAVDKA | 0.000105863 | 0 | 0.708829307 |
| 3135 | ASFEAQGALAN | 0.000648377 | 6.3762E-05 | 0.034992023 |
| 3136 | ASFEAQGALANIA | 0.003117946 | 0 | 0.033514835 |
| 3137 | ASFEAQGALANIAV | 0 | 0 | 0.002079129 |
| 3138 | ASFEAQGALANIAVD | 0 | 0.000157525 | 0.03992191 |
| 3139 | ASFEAQGALANIAVDK | 0.006469488 | 0.000762053 | 1.10392635 |
| 3140 | ASFEAQGALANIAVDKA | 0.000247955 | 0 | 1.739736664 |
| 3141 | ASFEAQGALANIAVDKAN | 0 | 0 | 0.499194633 |
| 3142 | EFGRFASFEAQGALANIAVDKAN | 0 | 0 | 0.014823626 |
| 3143 | GRFASFEAQGALANIAVDKAN | 0 | 0 | 0.008828611 |
| 3144 | RFASFEAQGALANIAVDKAN | 0 | 0 | 0.011160696 |
| 3145 | SFEAQGALAN | 0 | 0 | 0.004975177 |
| 3146 | SFEAQGALANIAVD | 0 | 0 | 0.025590148 |
| 3147 | SFEAQGALANIAVDK | 0.000482218 | 0.001224494 | 0.398341257 |
| 3148 | FASFEAQGALANIA | 0.010645499 | 0 | 0 |
| 3149 | FASFEAQGALANIAVDKA | 0 | 0 | 0.00582244 |
| 3150 | GRFASFEAQGALANIAVDKA | 0 | 0 | 0.004291801 |
| 3151 | GRFASFEAQGALAN | 0.008583812 | 0 | 0.002475607 |
| 3152 | GRFASFEAQGALANIA | 0.002336688 | 8.65565E-05 | 0 |
| 3153 | GRFASFEAQGALANIAVD | 0 | 0 | 0.000363294 |
| 3154 | GRFASFEAQGALANIAVDK | 0 | 0 | 0.002692161 |
| 3155 | RFASFEAQGALAN | 0.010654745 | 0.000201433 | 0.000822054 |
| 3156 | RFASFEAQGALANIA | 0.015373394 | 0 | 7.94605E-05 |
| 3157 | RFASFEAQGALANIAVD | 0 | 0 | 0 |
| 3158 | RFASFEAQGALANIAVDK | 0 | 0 | 0.002973445 |
| 3159 | EFGRFASFEAQGAL | 0 | 0.003469215 | 0.000276858 |
| 3160 | EFGRFASFEAQGALA | 0 | 0.003066815 | 0 |
| 3161 | EFGRFASFEAQGALAN | 0.001951635 | 0.000297926 | 0 |
| 3162 | EFGRFASFEAQGALANIAVDK | 0 | 0 | 0.005388897 |
| 3163 | EFGRFASFEAQGALANIAVDKA | 0 | 0 | 0.003745714 |
| 3164 | FGRFASFEAQGAL | 0 | 0.004094188 | 0 |
| 3165 | FGRFASFEAQGALA | 0 | 0.002122612 | 0 |
| 3166 | AQGGLANIAILN | 0 | 0 | 0.003507311 |
| 3167 | AQGGLANIAILNN | 0 | 0 | 0.003543458 |
| 3168 | EAQGGLANIAILNN | 0 | 0 | 0.00971964 |
| 3169 | FEAQGGLANIAILNN | 0 | 7.84557E-05 | 0.003169484 |
| 3170 | FSFEAQGGLANIAILNN | 0.000138344 | 3.95753E-05 | 0.007578045 |
| 3171 | SFEAQGGLANIAILNN | 0.00028053 | 0 | 0.007764866 |
| 3172 | EAQGGLANIAILN | 0 | 0 | 0.009587336 |
| 3173 | FEAQGGLANIAILN | 0.000797959 | 0 | 0.00329632 |
| 3174 | FSFEAQGGLANIAILN | 0 | 0 | 0.009515845 |
| 3175 | SFEAQGGLANIAILN | 0 | 7.2291E-05 | 0.026560552 |
| 3176 | SFEAQGGLANIAIL | 0 | 0 | 0.000793601 |
| 3177 | FSFEAQGGLANIA | 0 | 0 | 0.002797036 |
| 3178 | FSFEAQGGLANIAI | 0 | 0 | 0.002649768 |
| 3179 | EEFGRFASF | 0 | 0.004019749 | 0 |
| 3180 | LEEFGRFASFEAQ | 0.000592382 | 0.002158239 | 0 |
| 3181 | LEEFGRFASFEAQG | 0.000194149 | 0.003505176 | 0 |
| 3182 | ETVWRLEEFGRF | 0 | 0.000257757 | 0 |
| 3183 | ETVWRLEEFGRFA | 0 | 0.000702392 | 0 |
| 3184 | ETVWRLEEFGRFAS | 0 | 0.000239624 | 0 |
| 3185 | SPPEGDPESAVTELQ | 0 | 0.002972954 | 0 |
| 3186 | EENVPSSVTDVALPAL | 0 | 0.001153616 | 0 |
| 3187 | VEENVPSSVTDVALPAL | 6.28858E-05 | 0.002044611 | 7.0669E-05 |
| 3188 | VPSSVTDVALPAL | 0 | 0.00237108 | 0 |
| 3189 | VPSSVTDVALPALL | 0 | 0.000945833 | 0 |
| 3190 | IFDDVSSGVSQLASK | 0 | 0 | 0.001558519 |
| 3191 | GIAAVTDIPLGEI | 0.000700747 | 0.000354406 | 0 |
| 3192 | GIAAVTDIPLGEII | 0 | 0.00121641 | 0 |
| 3193 | GIAAVTDIPLGEIIS | 0 | 0.003372798 | 0 |
| 3194 | KGIAAVTDIPLGEIIS | 0 | 0.001593453 | 0 |
| 3195 | KGIAAVTDIPLGEII | 0 | 0.000767961 | 0 |
| 3196 | GWEEGVAQMSVGQR | 0 | 0.00038047 | 0.005469004 |
| 3197 | RGWEEGVAQMSVGQR | 0 | 0.000795817 | 0.002921807 |
| 3198 | VIRGWEEGVAQMSVGQR | 0 | 0 | 0.052780344 |
| 3199 | RGWEEGVAQMSVG | 0 | 0 | 0.009917251 |
| 3200 | RGWEEGVAQMSVGQ | 1.67812E-05 | 0 | 0.008056826 |
| 3201 | VIRGWEEGVAQMSVGQ | 0.000167546 | 0.000102054 | 0.043553058 |
| 3202 | VIRGWEEGVAQMS | 0 | 0 | 0.00095941 |
| 3203 | VIRGWEEGVAQMSVG | 9.28766E-05 | 0.000376946 | 0.058029405 |
| 3204 | LAKTAFDEAIAE | 7.29892E-05 | 0.000746965 | 0 |
| 3205 | LAKTAFDEAIAELDT | 0.001789017 | 0 | 0.000349885 |
| 3206 | SLAKTAFDEAIAELDT | 0.000720648 | 1.63933E-05 | 0 |
| 3207 | DPTPDPNTAYASYPGASVD | 0 | 0.000138682 | 0.008945504 |
| 3208 | DPTPDPNTAYASYPGASVDN | 0 | 0 | 0.07453351 |
| 3209 | DPTPDPNTAYASYPGASVDNY | 0 | 0 | 0.032298617 |
| 3210 | DPTPDPNTAYASYPGASVDNYQ | 0 | 0 | 0.015333884 |
| 3211 | PNTAYASYPGASVDN | 0 | 0.002695525 | 0.002147816 |
| 3212 | TPDPNTAYASYPGASVDN | 0 | 5.19159E-05 | 0.011549505 |
| 3213 | TPDPNTAYASYPGASVDNY | 0 | 0 | 0.000986835 |
| 3214 | DSPSKSGSEAQTTKD | 0 | 0 | 0.000726392 |
| 3215 | DSPSKSGSEAQTTKDVP | 0 | 0 | 0.016563174 |
| 3216 | DSPSKSGSEAQTTKDVPN | 0 | 0 | 0.023373218 |
| 3217 | SPSKSGSEAQTTKDVP | 0 | 0 | 0.026196071 |
| 3218 | SPSKSGSEAQTTKDVPN | 0 | 0.000825625 | 0.018834133 |
| 3219 | SPSKSGSEAQTTKDVPNK | 0 | 0.000759615 | 4.74427E-06 |
| 3220 | EHSYTSSAEAEASR | 0 | 4.84535E-05 | 0.001295155 |
| 3221 | HSYTSSAEAEASR | 0 | 0.000189586 | 0.004103758 |
| 3222 | IVEHSYTSSAEAEASR | 0 | 0.000924282 | 9.58115E-05 |
| 3223 | VEHSYTSSAEAEASR | 0 | 9.09166E-05 | 0.000857325 |
| 3224 | IVEHSYTSSAEAEA | 0 | 0.000398679 | 0 |
| 3225 | EPGKGEQASSSESDPEGP | 0 | 0.001343796 | 0 |
| 3226 | KPNMVTPGHACPIK | 0 | 0 | 0.001976828 |
| 3227 | ATATATARADQEGA | 0 | 0.000504632 | 0 |
| 3228 | ATATATARADQEGAR | 0 | 0.002865712 | 0 |
| 3229 | PPGAPHTEEEGAED | 0 | 0.000907545 | 0 |
| 3230 | LPSARTDEQALL | 0.000187198 | 0.008174926 | 0.0013508 |
| 3231 | LPSARTDEQALLS | 6.88759E-05 | 0.003840148 | 0 |
| 3232 | SLPSARTDEQALLSSIL | 0 | 0.000325032 | 0 |
| 3233 | SLPSARTDEQALL | 0 | 0.0049015 | 0 |
| 3234 | PSARTDEQALLSSI | 0 | 0.002082737 | 0 |
| 3235 | EEQLAAAKAQQEQ | 0 | 0.000111322 | 0.001913475 |
| 3236 | EEQLAAAKAQQEQE | 0 | 0.000278424 | 0.008062096 |
| 3237 | EEQLAAAKAQQEQEL | 0 | 0 | 0.006129391 |
| 3238 | WEEQLAAAKAQQEQE | 1.65498E-05 | 0 | 0.00165287 |
| 3239 | VSNDVGPGRSEEVF | 0 | 0 | 0.001495653 |
| 3240 | VSNDVGPGRSEEVFLQ | 0 | 0 | 0.004208242 |
| 3241 | GPSLKGEEEPLVAS | 0 | 0 | 0 |
| 3242 | SGPSLKGEEEPLVAS | 0.001296428 | 0.000213362 | 0.000208838 |
| 3243 | SGPSLKGEEEPLVASE | 0.003880246 | 0 | 0 |
| 3244 | SGPSLKGEEEPLV | 0.006061163 | 0 | 0 |
| 3245 | SGPSLKGEEEPLVA | 0.00534801 | 0 | 6.44629E-05 |
| 3246 | VPGMHTEDNPG | 0 | 0.005989351 | 0 |
| 3247 | VPGMHTEDNPGKVE | 0 | 0.003135106 | 0 |
| 3248 | VPGMHTEDNPGKVEHTEE | 0 | 0 | 0.000448614 |
| 3249 | DEVIGQVLSTLKSEDVPYT | 0 | 0.000710795 | 0 |
| 3250 | GNDEVIGQVLSTLKSEDVPYT | 0 | 0.000619838 | 0 |
| 3251 | IGQVLSTLKSEDVP | 0 | 0.012508754 | 0 |
| 3252 | IGQVLSTLKSEDVPY | 0 | 0.006155752 | 0 |
| 3253 | IGQVLSTLKSEDVPYT | 0 | 0.028281167 | 0 |
| 3254 | VIGQVLSTLKSEDVPYT | 0 | 0.001562195 | 0.000103206 |
| 3255 | DIYETLKSNNMK | 0 | 0.002363001 | 0.000378518 |
| 3256 | LDIYETLKSNNMK | 0 | 0.000749134 | 3.44341E-05 |
| 3257 | YPYDGIHPDDLSFK | 0.00251452 | 0 | 0 |
| 3258 | ESPDNIEKGMISSSKTDD | 0 | 0 | 0.004362053 |
| 3259 | SPDNIEKGMISSSKTD | 0 | 0 | 0.000181125 |
| 3260 | SPDNIEKGMISSSKTDD | 0 | 0 | 0.026155995 |
| 3261 | IEKGMISSSKTDDL | 1.98624E-05 | 0.00379829 | 0 |
| 3262 | IEKGMISSSKTDDLT | 4.58951E-05 | 0.003752068 | 0 |
| 3263 | DPFNPFELTNHAVLL | 0 | 0.002870307 | 0 |
| 3264 | DPFNPFELTNHAVLLVG | 0 | 0.001834835 | 0.00104634 |
| 3265 | DPFNPFELTNHAVLLVGYG | 0 | 0.000911285 | 0 |
| 3266 | NVNHGVLVVGYG | 0 | 0 | 0 |
| 3267 | QNVNHGVLVVGYG | 0 | 0.000252443 | 0.008399393 |
| 3268 | QNVNHGVLVVGYGDLN | 0 | 0 | 0.002156959 |
| 3269 | TQNVNHGVLVVGYG | 0 | 0.000176284 | 0.008160862 |
| 3270 | GEHGLIIRV | 0 | 0.003395392 | 0.000765526 |
| 3271 | AELISAIKRTLARL | 0 | 0 | 0.000614928 |
| 3272 | NEVVAGIKEY | 0 | 0.001214306 | 0.00016106 |
| 3273 | APGAESAVASFVTQL | 0 | 0 | 0.000436913 |
| 3274 | ATVASEKESVM | 0.001793509 | 0.00224307 | 0 |
| 3275 | DATVASEKESVM | 0.002168242 | 0.011454348 | 0 |
| 3276 | GDATVASEKESVM | 0.001356185 | 0.002642654 | 6.67622E-05 |
| 3277 | GPAGDATVASEKESVM | 0.041027161 | 0.11038999 | 0.000278318 |
| 3278 | GPAGDATVASEKE | 0 | 0.014624851 | 0 |
| 3279 | GPAGDATVASEKES | 4.45346E-05 | 0.057712432 | 0 |
| 3280 | GPAGDATVASEKESV | 0 | 0.003582219 | 0 |
| 3281 | RGPAGDATVASEKES | 3.43441E-05 | 0.000578722 | 0 |
| 3282 | LPASPDAGVPSDDTLR | 0 | 0 | 0.001269245 |
| 3283 | DTDSSVASEVR | 5.49089E-06 | 0 | 0.002047461 |
| 3284 | GPQTPLAPEEETQARLLPA | 0 | 0.000274782 | 0 |
| 3285 | QTPIAAEIEHFIH | 0.002544469 | 0.000579394 | 0 |
| 3286 | TPIAAEIEHFI | 0.028816821 | 9.94917E-05 | 0 |
| 3287 | TPIAAEIEHFIH | 0.177117543 | 0.024862098 | 0 |
| 3288 | TPIAAEIEHFIHL | 0.001632469 | 8.52601E-05 | 0 |
| 3289 | TPIAAEIEHFIHLI | 0.010481192 | 0.000441301 | 0 |
| 3290 | TPIAAEIEHFIHLIT | 0.016551474 | 0.001296045 | 2.79766E-05 |
| 3291 | INIHSETSVPDH | 0.000139159 | 0.002142581 | 0 |
| 3292 | VINIHSETSVPDH | 0.000353645 | 0.002774588 | 0.002533413 |
| 3293 | QVHPDTGISSK | 0.000309513 | 0.000388613 | 0.000163003 |
| 3294 | KPIHQGPDAAVTGHIR | 0 | 0 | 0.002210092 |
| 3295 | NPSSDVQADGALG | 0.011254841 | 0 | 0.001535114 |
| 3296 | NPSSDVQADGALGSRSPPG | 0.0015889 | 0 | 0 |
| 3297 | NPSSDVQADGALGSRSPPGVG | 0 | 0 | 0.000446569 |
| 3298 | ARFDPQGGLAGIAAIK | 0 | 0 | 0.030836128 |
| 3299 | ARFDPQGGLAGIAAIKA | 0 | 0 | 0.002142827 |
| 3300 | DPQGGLAGIAAIK | 0 | 0 | 0.04537368 |
| 3301 | FDPQGGLAGIAAIK | 0 | 0 | 0.024589741 |
| 3302 | RFDPQGGLAGIAAIK | 0 | 0 | 0.00463848 |
| 3303 | DPQGGLAGIAAIKA | 0 | 0.000153326 | 0.002211611 |
| 3304 | DPQGGLAGIAAIKAH | 0 | 0 | 0.00099307 |
| 3305 | FDPQGGLAGIAAIKAH | 0 | 0 | 0 |
| 3306 | EEIAMATVTALR | 0 | 0 | 0.008642152 |
| 3307 | EEIAMATVTALRR | 0 | 0 | 0.000958118 |
| 3308 | EEIAMATVTALRRTVPP | 0 | 0 | 0.000856621 |
| 3309 | SHEEIAMATVTALRRTVPP | 0 | 0 | 0.001672463 |
| 3310 | SHEEIAMATVTALR | 0 | 0.000203617 | 0.004682851 |
| 3311 | SHEEIAMATVTALRR | 0 | 0 | 0.001434509 |
| 3312 | TPEEIAMATVTALR | 0 | 0.00022601 | 0.004298685 |
| 3313 | TPEEIAMATVTALRR | 0 | 0 | 0.002637448 |
| 3314 | TPEEIAMATVTALRRTVPP | 0 | 0 | 0.005725648 |
| 3315 | DDQDEVLGGMTPLKK | 0 | 6.83145E-05 | 0.001075918 |
| 3316 | EVLESSRPGTTVG | 0.000627671 | 2.56031E-05 | 0.00116667 |
| 3317 | FEVLESSRPGTTVG | 0 | 0 | 0.010694386 |
| 3318 | FEVLESSRPGTTVGVV | 0 | 6.97787E-05 | 0.002425283 |
| 3319 | NFEVLESSRPGTTVGVV | 0 | 0 | 0.003042631 |
| 3320 | NFEVLESSRPGTTVG | 0 | 0 | 0.007398326 |
| 3321 | VLENAEGARTTPSVVA | 0 | 0 | 0.001338384 |
| 3322 | GPHLVQSDGTVPFWA | 0 | 0.000663919 | 7.94657E-05 |
| 3323 | AGEEGEAGDEELPLPPG | 0.001574636 | 0 | 0 |
| 3324 | EEGEAGDEELPLPPG | 0.032246757 | 0.001348497 | 7.76025E-05 |
| 3325 | GEEGEAGDEELPLPPG | 0.014131484 | 0 | 0 |
| 3326 | EEGEAGDEELPLPPGDVG | 0.008255784 | 0.000777375 | 0 |
| 3327 | GEEGEAGDEELPLPPGDVG | 0.006059116 | 0.000196715 | 0 |
| 3328 | LPPGQESESAESAK | 0 | 0.000978623 | 0 |
| 3329 | DLVAFSSDEELT | 0 | 0.000631145 | 0 |
| 3330 | VAFSSDEELT | 0.000357062 | 0.002454137 | 0 |
| 3331 | LVAFSSDEELTM | 0.000210619 | 0.000674176 | 0 |
| 3332 | LATFSTDQELRFV | 0.000317006 | 0.002971174 | 0.00013564 |
| 3333 | NPTDEDVPWPGFI | 1.07929E-05 | 0.000644061 | 0 |
| 3334 | APASNREADTVELAELGP | 0.000553177 | 0.003479168 | 0 |
| 3335 | APASNREADTVELAELGPL | 0 | 0.000871429 | 0.000128456 |
| 3336 | EEMPLNVADLI | 0 | 0.001851374 | 0 |
| 3337 | FITIQVAQM | 0 | 9.0371E-05 | 0 |
| 3338 | GVVDSEDLPLNIS | 0 | 0.000436718 | 0 |
| 3339 | IRGVVDSEDLPLNIS | 0 | 0.002011348 | 0 |
| 3340 | IRGVVDSEDLPLN | 0 | 0.002774154 | 0.000165101 |
| 3341 | QEIPVNVRF | 0 | 0.001953415 | 0 |
| 3342 | FPRELASLSPNLVSS | 0 | 0.001411506 | 0 |
| 3343 | FPRELASLSPNLVSSF | 0 | 0.002470008 | 0 |
| 3344 | HIPDVITYL | 0 | 0 | 0.00607426 |
| 3345 | IPELDMTEVVAPFM | 0.001931808 | 0.004382276 | 0 |
| 3346 | IPELDMTEVVAPFMA | 0.000443949 | 0.000382563 | 0 |
| 3347 | IPELDMTEVVAPFMAN | 0 | 0 | 0 |
| 3348 | LDMTEVVAPFM | 0.000660487 | 0.001183387 | 0 |
| 3349 | KIPELDMTEVVAPFM | 0.000441497 | 0.000168809 | 0 |
| 3350 | KIPELDMTEVVAPFMA | 0.00112053 | 4.75257E-05 | 8.43242E-05 |
| 3351 | RIADLISSF | 0 | 0.000332416 | 0.000588506 |
| 3352 | LPSTATSVNIPDLLPG | 0 | 0.001375368 | 0.000518997 |
| 3353 | IPIPSTGSVEMAVA | 0 | 0 | 0.006563113 |
| 3354 | APREVLTGNDEVIG | 0 | 0.008127837 | 0 |
| 3355 | APREVLTGNDEVIGQ | 7.94949E-05 | 0.003930727 | 0.000291239 |
| 3356 | APREVLTGNDEVIGQV | 0 | 0.032390793 | 0 |
| 3357 | EEENLFEIITADEVH | 0 | 0.000253848 | 0.000554372 |
| 3358 | EENLFEIITADEVH | 0.00014144 | 0.000948316 | 0 |
| 3359 | LFEIITADEVHY | 0 | 0.000944433 | 0 |
| 3360 | LFEIITADEVHYF | 0 | 0.001053783 | 0 |
| 3361 | NLFEIITADEVHY | 0 | 4.31586E-05 | 0 |
| 3362 | FEVVTPEEQ | 0.005278515 | 0 | 1.89646E-05 |
| 3363 | FEVVTPEEQN | 0.001345681 | 0 | 1.77843E-05 |
| 3364 | KPPSSGTSSEFEVVTPEEQN | 0.023892736 | 0.00825609 | 0 |
| 3365 | KPPSSGTSSEFEVVTPEEQNSPE | 0.009636729 | 0.000683248 | 0 |
| 3366 | TSSEFEVVTPEEQ | 0.012315732 | 0.000569426 | 0 |
| 3367 | KPPSSGTSSEFEVVTPE | 4.42189E-05 | 0.070531587 | 0 |
| 3368 | KPPSSGTSSEFEVVTPEE | 0 | 0.0438439 | 0 |
| 3369 | KPPSSGTSSEFEVVTPEEQ | 0.052584906 | 0.048346247 | 0 |
| 3370 | EVVTTEENIPD | 8.58E-05 | 0.001317378 | 0 |
| 3371 | EVVTTEENIPDVVP | 0 | 0.00310883 | 0 |
| 3372 | EVVTTEENIPDVVPG | 0 | 0.012600164 | 0 |
| 3373 | ITEVVTTEENIPDVV | 0 | 0.003612521 | 0 |
| 3374 | ITEVVTTEENIPDVVP | 0 | 0.015609326 | 0 |
| 3375 | ITEVVTTEENIPDVVPG | 0 | 0.046046497 | 3.73134E-05 |
| 3376 | ITEVVTTEENIPDVVPGSHLTPIE | 0 | 0.000609931 | 0 |
| 3377 | SITEVVTTEENIPDVVPGSHLTPIE | 0 | 0.000816714 | 0 |
| 3378 | TEVVTTEENIPDVVP | 0 | 0.010586596 | 0.00026726 |
| 3379 | VVTTEENIPD | 0 | 0.004328798 | 0 |
| 3380 | VVTTEENIPDVV | 0 | 0.002860619 | 0 |
| 3381 | VVTTEENIPDVVP | 0 | 0.008135452 | 0 |
| 3382 | VVTTEENIPDVVPG | 0 | 0.008947066 | 0 |
| 3383 | TEVVTTEENIPDV | 0 | 0.001369817 | 0 |
| 3384 | TEVVTTEENIPDVV | 0 | 0.002135081 | 0.000113015 |
| 3385 | TEVVTTEENIPDVVPG | 0 | 0.007113323 | 0 |
| 3386 | VVTTEENIPDV | 0 | 0.000769644 | 0 |
| 3387 | ITEVVTTEENIPD | 0.000282275 | 0.008912197 | 6.42322E-05 |
| 3388 | ITEVVTTEENIPDV | 0 | 0.003725666 | 0 |
| 3389 | SITEVVTTEENIPDVVP | 0 | 0.002081423 | 0 |
| 3390 | SITEVVTTEENIPDVVPG | 0 | 0.003700842 | 0.000422032 |
| 3391 | SITEVVTTEENIPD | 0 | 0.001835205 | 0.000211309 |
| 3392 | DHWQILPGDPFDN | 0 | 0 | 0.000896642 |
| 3393 | FELLPGESNKIP | 0 | 0 | 0.00311279 |
| 3394 | FELLPGESNKIPR | 0 | 0.000468033 | 0.001101568 |
| 3395 | FELLPGESNKIPRL | 0 | 0 | 0.000204959 |
| 3396 | FELLPGESNKIPRLR | 0 | 0 | 0.002487852 |
| 3397 | GPMFELLPGESNKIPRLR | 0 | 0.000502608 | 0.010516597 |
| 3398 | GPMFELLPGESNKIPR | 0 | 0.00133715 | 0.002258175 |
| 3399 | GPMFELLPGESNKIPRL | 0 | 0.000140118 | 0.001637941 |
| 3400 | FNVLTTNTDGKIE | 0 | 0.001038567 | 0 |
| 3401 | VFEVVATNGDTHLG | 0 | 0.0003835 | 0.000716059 |
| 3402 | VFEVVATNGDTHLGGE | 0 | 0.004863664 | 0 |
| 3403 | VVATNGDTHLGGE | 0 | 0.000260823 | 1.9686E-05 |
| 3404 | APPASSLYSSPVNSSAP | 0 | 0 | 0.001684906 |
| 3405 | ARIITGQEEGAYG | 0.004315675 | 6.97471E-05 | 0.001473587 |
| 3406 | ARIITGQEEGAYGWI | 0.00256176 | 0 | 0.000256186 |
| 3407 | GARIITGQEEGAYGWI | 0.002413506 | 0 | 0 |
| 3408 | RIITGQEEGAYG | 0.001614567 | 0.00013754 | 0.000164449 |
| 3409 | GARIITGQEEGAYG | 0.004530221 | 0.003470143 | 0.000147674 |
| 3410 | QGARIITGQEEGAYG | 0 | 0.000818523 | 0.000260387 |
| 3411 | DFQGARIITG | 0 | 0.000226578 | 0.005716024 |
| 3412 | DFQGARIITGQE | 0 | 0.000156179 | 0.029492936 |
| 3413 | DFQGARIITGQEE | 0.003525643 | 3.47337E-05 | 0.066553253 |
| 3414 | DFQGARIITGQEEG | 0 | 0.000222283 | 0.034120403 |
| 3415 | DFQGARIITGQEEGA | 0 | 0.000251832 | 0.002093912 |
| 3416 | YPFDFQGARII | 0 | 0 | 0.001187206 |
| 3417 | YPFDFQGARIIT | 0.00025041 | 5.42484E-05 | 0.001052048 |
| 3418 | YPFDFQGARIITG | 0 | 0 | 0.019582661 |
| 3419 | YPFDFQGARIITGQE | 6.1665E-05 | 0 | 0.045960317 |
| 3420 | YPFDFQGARIITGQEE | 0.000125645 | 4.6912E-05 | 0.09509066 |
| 3421 | YPFDFQGARIITGQEEG | 0 | 9.07567E-05 | 0.041166257 |
| 3422 | EDRSAGSVESPSVS | 0 | 0.000559081 | 0 |
| 3423 | EDRSAGSVESPSVSS | 0 | 0.000560875 | 0 |
| 3424 | IGGAASLDPGALGRL | 0 | 0.001199009 | 0 |
| 3425 | LIGGAASLDPGAL | 0 | 0 | 0.006688389 |
| 3426 | GAASLDASCLGRLG | 0 | 0.000523767 | 0 |
| 3427 | VSGAASLDASCLGRLG | 0 | 0.001747496 | 0 |
| 3428 | GVLFHSSPALQPAA | 0 | 0.001606536 | 0.001478587 |
| 3429 | LLSGVLFHSSPALQPAAD | 0 | 0 | 0.000913789 |
| 3430 | LPDSSKLLSGVLFHSSPALQPAAD | 0 | 0 | 0.001904637 |
| 3431 | SGVLFHSSPALQPAAD | 0 | 0 | 0.001304694 |
| 3432 | PDSSKLLSGVLFHSSPAL | 0 | 0 | 0.000762975 |
| 3433 | DAAFAGCSQDPEFH | 0.000747444 | 0 | 3.73134E-05 |
| 3434 | DEDAAFAGCSQDPEFHK | 0.001371141 | 0 | 0 |
| 3435 | DGSDEDAAFAGCSQDPEFHK | 0.001104442 | 0 | 0 |
| 3436 | EDAAFAGCSQDPEFH | 0.001984404 | 0 | 0 |
| 3437 | EDAAFAGCSQDPEFHK | 0.006613574 | 0 | 0 |
| 3438 | EQQSPQSGKVIPDR | 0 | 4.93622E-05 | 0.000663585 |
| 3439 | SPQSGKVIPDR | 0 | 2.40481E-05 | 0.000442198 |
| 3440 | ATVLSIPELL | 0 | 0.001175451 | 0 |
| 3441 | ILATVLSIPELL | 0 | 0.000938335 | 0 |
| 3442 | IWILATVLSIPELL | 0 | 0.001250334 | 0 |
| 3443 | TVLSIPELL | 0 | 0.000197571 | 0 |
| 3444 | TVLSIPELLY | 0 | 0.001040728 | 0 |
| 3445 | ILATVLSIPEL | 0 | 0.000480681 | 0 |
| 3446 | IWILATVLSIPEL | 0 | 0.001435648 | 0 |
| 3447 | IHIIGGVIA | 0 | 0 | 0.000945071 |
| 3448 | IHIIGGVIAVG | 0 | 0 | 0.004144211 |
| 3449 | SIHIIGGVIAVG | 0 | 0 | 0.000739247 |
| 3450 | SIHIIGGVIA | 0 | 0 | 0.000254575 |
| 3451 | FIIVVPGMISRIL | 0 | 0 | 0.000727455 |
| 3452 | IIVVPGMISRIL | 0 | 0 | 0.017148682 |
| 3453 | KPLPGIIMGGILP | 0 | 0 | 0.000362024 |
| 3454 | KPLPGIIMGGILPFG | 0 | 0 | 0.026807365 |
| 3455 | PVVMAGIIAI | 0 | 0 | 0.000346618 |
| 3456 | LHLSVADLLFV | 0 | 0.000427704 | 0 |
| 3457 | RLHLSVADLLFV | 0 | 0.0001528 | 0 |
| 3458 | YRLHLSVADLLFV | 0 | 0.000157438 | 0 |
| 3459 | IMLPGVLRY | 0 | 0.009664098 | 0.002154072 |
| 3460 | YLLPAIVHI | 0 | 0 | 0.011717875 |
| 3461 | DNEIKVAKAEAAGHR | 4.82517E-05 | 3.73952E-05 | 0.006798345 |
| 3462 | DNEIKVAKAEAAGHRD | 0 | 0 | 0.001937071 |
| 3463 | DNEIKVAKAEAAGHRDT | 0 | 0 | 0.000210867 |
| 3464 | EIKVAKAEAAGHR | 0 | 0 | 0.000374533 |
| 3465 | MDNEIKVAKAEAAGHR | 0 | 1.56375E-05 | 0.00079347 |
| 3466 | EEAWVIGSVVARAEGSPR | 0 | 0 | 0.001866852 |
| 3467 | SSGLYSLSSVVTVPS | 0 | 0 | 0.00577511 |
| 3468 | SSGLYSLSSVVTVPSS | 0 | 0.00022301 | 0.002323988 |
| 3469 | HYSPIYLSF | 7.86076E-05 | 0 | 0.000285016 |
| 3470 | VPPVQVSPLIKLGRY | 0 | 0 | 0.000736108 |
| 3471 | VPPVQVSPLIKLGRYSALFLG | 0.000651012 | 0 | 0 |
| 3472 | IPPSFASIFLTKSTK | 0.001536834 | 0 | 0.000340294 |
| 3473 | IPPSFASIFLTKSTKL | 0 | 0.0090548 | 1.79919E-05 |
| 3474 | IPPSFASIFLTKSTKLT | 0 | 0.028550236 | 0 |
| 3475 | SIFLTKSTKL | 0 | 0.00019208 | 0 |
| 3476 | DLSALVRHAEPEQN | 0.000699995 | 0.00017874 | 0.002867544 |
| 3477 | SALVRHAEPEQN | 2.0638E-05 | 0 | 0.010532381 |
| 3478 | SALVRHAEPEQNWE | 3.76115E-05 | 0 | 0.007335453 |
| 3479 | SALVRHAEPEQNWEA | 0.000163805 | 0 | 0.01532346 |
| 3480 | EELPTLLHF | 0 | 0.000607137 | 0 |
| 3481 | VINSEELSSIL | 0 | 0.000551023 | 0 |
| 3482 | VINSEELSSILK | 0 | 0.004824546 | 0 |
| 3483 | YPEEISSMVLTKMK | 0.000379979 | 0.000948446 | 0 |
| 3484 | YPEEVSSMVLTKM | 0.001233496 | 0.00164314 | 0 |
| 3485 | YPEEVSSMVLTKMK | 0.011067248 | 0.018417743 | 0 |
| 3486 | YPEEVSSMVLTKMKE | 0.004178291 | 0.005099101 | 2.05567E-05 |
| 3487 | YPEEVSSMVLTKMKEIA | 0.003250335 | 0.002880788 | 0 |
| 3488 | KKEDVGTVVGIDLG | 0 | 0.000624149 | 0 |
| 3489 | VPRKTVSSGELATVVR | 0 | 0 | 0.002474167 |
| 3490 | VPRKTVSSGELATVVRR | 0 | 0 | 0.001121175 |
| 3491 | IVEGEKTVESEQITPQ | 0 | 0.000523803 | 0 |
| 3492 | DNKGIDSDASYPY | 0 | 0.004569862 | 0.000143157 |
| 3493 | DNKGIDSDASYPYK | 0.003369435 | 0.198421465 | 3.55249E-05 |
| 3494 | DNKGIDSDASYPYKA | 0.002046735 | 0.023823534 | 0 |
| 3495 | DNKGIDSDASYPYKAM | 6.48917E-05 | 0.002338976 | 0 |
| 3496 | IDNKGIDSDASYPYKAM | 5.53222E-05 | 0.010836289 | 0.001032803 |
| 3497 | IDNKGIDSDASYPYKAMD | 0.000250692 | 0.006406241 | 0.000495086 |
| 3498 | KGIDSDASYPY | 0 | 0.001143916 | 0 |
| 3499 | NKGIDSDASYPYK | 3.44014E-06 | 0.034220376 | 0 |
| 3500 | GIDSDASYPYK | 0 | 0.104189867 | 0 |
| 3501 | GIDSDASYPYKA | 0 | 0.007510773 | 0 |
| 3502 | IDNKGIDSDASYPYKA | 0.000725789 | 0.056273764 | 0.000192578 |
| 3503 | IDSDASYPYK | 0 | 0.003436903 | 0 |
| 3504 | IIDNKGIDSDASYPYKA | 0 | 0.002049372 | 6.32991E-05 |
| 3505 | KGIDSDASYPYKA | 7.72065E-05 | 0.020808247 | 0.001250952 |
| 3506 | NKGIDSDASYPYKA | 1.59265E-05 | 0.004388618 | 0 |
| 3507 | KGIDSDASYPYK | 2.66562E-05 | 0.095693682 | 0 |
| 3508 | KGIDSDASYPYKAM | 0 | 0.000107239 | 0 |
| 3509 | IDNKGIDSD | 0 | 0.000439622 | 0 |
| 3510 | IDNKGIDSDASYPY | 0 | 0.020178929 | 0 |
| 3511 | IDNKGIDSDASYPYK | 0.003960889 | 0.856382398 | 0.000365092 |
| 3512 | IIDNKGIDSDASYPYK | 0.000482734 | 0.05129744 | 0 |
| 3513 | KVDKVIQAQTAFSANPA | 0 | 0 | 0.004860804 |
| 3514 | DRSDHLIQTDTVN | 0 | 0.000226627 | 0 |
| 3515 | SDHLIQTDTVN | 1.72814E-05 | 0.002386391 | 3.59393E-05 |
| 3516 | SDHLIQTDTVNLH | 0 | 0.000831148 | 0.000109805 |
| 3517 | SDHLIQTDTVNLHRKPKP | 0 | 0.000259161 | 0 |
| 3518 | KFWEVISDEHGIDPT | 0 | 0.002440443 | 8.94225E-05 |
| 3519 | VISDEHGIDPT | 0 | 0.001203547 | 0.000315706 |
| 3520 | DPDGSWAQIAEK | 0 | 0 | 0.018998286 |
| 3521 | DPDGSWAQIAEKR | 0 | 0 | 0.026754704 |
| 3522 | DPDGSWAQIAEKRA | 0 | 4.76872E-05 | 0.002792664 |
| 3523 | IQDPDGSWAQIAEKR | 0 | 0 | 0.00477112 |
| 3524 | QDPDGSWAQIAEKR | 8.18921E-05 | 0.001083145 | 0.003393678 |
| 3525 | WIQDPDGSWAQIAEKR | 0 | 0 | 0.003752245 |
| 3526 | AALSVSQENPEMEGPE | 0.008289869 | 0 | 0 |
| 3527 | ALSVSQENPEMEGPE | 0.004655142 | 0.000368125 | 2.4178E-05 |
| 3528 | SVSQENPEMEGPE | 0.002180269 | 9.14292E-05 | 0 |
| 3529 | ETTEEEANMETKPTGAQ | 0.011766136 | 0.015776783 | 0 |
| 3530 | TTEEEANMETKPTGAQ | 0 | 0.001234362 | 0 |
| 3531 | ATVIQTLSEDQRFR | 0 | 0.001197574 | 0 |
| 3532 | IGHSFGGATVIQTLS | 0 | 0.000117946 | 0.004283652 |
| 3533 | IGHSFGGATVIQTLSE | 0 | 0 | 0.0099228 |
| 3534 | IGHSFGGATVIQTLSED | 0 | 0.000140016 | 0.015571764 |
| 3535 | GLPLHVATLLTGLL | 0 | 0.000264975 | 0 |
| 3536 | GLPLHVATLLTGLLE | 0 | 0.000579886 | 0 |
| 3537 | LPLHVATLLTGLL | 0 | 0.002771157 | 0 |
| 3538 | LPLHVATLLTGLLE | 0 | 0.008626871 | 0 |
| 3539 | PLHVATLLTGLLECLG | 0 | 0.00130536 | 0 |
| 3540 | IAVVAGVVQTIL | 0 | 0 | 0.02016902 |
| 3541 | LSAVVASIIESIG | 0 | 0 | 0.000229512 |
| 3542 | LSAVVASIIESIGDY | 1.09044E-05 | 9.09007E-05 | 0.000249162 |
| 3543 | IPDPDFTYLGGILNPIPD | 0 | 0 | 0.000840756 |
| 3544 | DRPVGGRGFGGAG | 0 | 0 | 0.003535013 |
| 3545 | DRPVGGRGFGGAGE | 0 | 0 | 0.002152647 |
| 3546 | DRPVGGRGFGGAGELG | 0 | 0 | 0.003049308 |
| 3547 | EPGREQVVEDRPVGGRGFGGAGELG | 0 | 0 | 0.000909768 |
| 3548 | VEDRPVGGRGFGGAGELG | 0 | 0 | 0.000698209 |
| 3549 | VEDRPVGGRGFGGAG | 0 | 4.47081E-05 | 0.014102764 |
| 3550 | VEDRPVGGRGFGGAGE | 0 | 5.97319E-05 | 0.006966244 |
| 3551 | VEDRPVGGRGFGGAGEL | 0 | 5.756E-05 | 0.007711337 |
| 3552 | LMDTRTATAELGWT | 0 | 0.000969039 | 0 |
| 3553 | LMDTRTATAELGWTA | 0 | 0.000889198 | 0.001609034 |
| 3554 | TLMDTRTATAELGWT | 0 | 0.000257357 | 0 |
| 3555 | LPYTPGSDVAGVIE | 0 | 0.007540227 | 0.00138967 |
| 3556 | LPYTPGSDVAGVIEA | 0 | 0 | 0 |
| 3557 | LPYTPGSDVAGVIEAV | 0 | 0.000176418 | 0 |
| 3558 | LPYTPGSDVAGVIEAVG | 0 | 0.000357967 | 0 |
| 3559 | QPYPSGADVPFGPP | 0.000204133 | 0 | 0.03791809 |
| 3560 | QPYPSGADVPFGPPL | 0 | 0 | 0.012614278 |
| 3561 | AASSLQSGVPSRFSG | 0 | 0 | 0.000640757 |
| 3562 | AASSLQSGVPSRFSGSG | 0 | 4.80636E-05 | 0.007382896 |
| 3563 | ASSLQSGVPSRFSG | 0 | 0 | 0.004730555 |
| 3564 | GATSLQSGVPSRFSG | 0 | 0 | 0.000145773 |
| 3565 | ASSLESGVPSRFSGSG | 0.000405958 | 0.000502955 | 0.00080269 |
| 3566 | ASSLQSGVPSRFSGSG | 0 | 0.000213036 | 0.008121669 |
| 3567 | DASSLESGVPSRFSGSG | 0 | 0.000712637 | 0.015749247 |
| 3568 | DASSLESGVPSRFS | 0 | 0.000102282 | 0.001738398 |
| 3569 | DASSLESGVPSRFSG | 0 | 4.57068E-05 | 0.002350045 |
| 3570 | DASNLESGVPSRF | 0 | 0.000187837 | 0.00383878 |
| 3571 | DASNLESGVPSRFS | 0 | 0.000159772 | 0.007507234 |
| 3572 | DASNLESGVPSRFSG | 0 | 0 | 0.002190226 |
| 3573 | IINSSITTK | 1.02919E-05 | 0.000627211 | 0 |
| 3574 | IPPPGEQLAVGGSLVQPAVAPS | 0 | 0 | 0.007388069 |
| 3575 | FSWRTQIDSPLN | 0 | 0.000723414 | 0 |
| 3576 | SPFFSWRTQIDSPLN | 0 | 0.006094882 | 0.00017853 |
| 3577 | SPFFSWRTQIDSPLNG | 0 | 0.000530793 | 0 |
| 3578 | SPFFSWRTQIDSPLNGK | 0 | 0.004069895 | 0.000523722 |
| 3579 | SPFFSWRTQIDSPLNGKV | 0 | 0.005993896 | 0 |
| 3580 | SPFFSWRTQIDSPL | 0.000556216 | 0.002573757 | 0 |
| 3581 | SPSFSWRTQIDSPLS | 0 | 0.002452413 | 0.000133384 |
| 3582 | SPSFSWRTQIDSPLSGK | 0 | 0.007437278 | 0 |
| 3583 | SPSFSWRTQIDSPLSGKV | 0 | 0.008979015 | 0 |
| 3584 | DALAQAAGLVNAGRIG | 0 | 0 | 0.001111232 |
| 3585 | GGGVVQPGR | 0 | 0 | 0.000309854 |
| 3586 | ESGGGVVQPGRS | 0 | 0 | 0.007930048 |
| 3587 | ESGGGVVQPGRSL | 0 | 0 | 0.007142589 |
| 3588 | ESGGGVVQPGRSLR | 0 | 0 | 0.002242908 |
| 3589 | GGGVVQPGRS | 0 | 0 | 0.000530175 |
| 3590 | LVESGGGVVQPGRSLR | 0 | 0 | 0.061117772 |
| 3591 | SGGGVVQPGRS | 0 | 0 | 0.001366806 |
| 3592 | VESGGGVVQPGRSLR | 0 | 0 | 0.002737391 |
| 3593 | VQLVESGGGVVQPGRSLR | 0 | 0 | 0.002211286 |
| 3594 | SGGGVVQPGRSLR | 0 | 2.57889E-06 | 0.000612961 |
| 3595 | GGGVVQPGRSL | 0 | 0 | 0.001414815 |
| 3596 | LVESGGGVVQPGRSL | 0 | 0 | 0.109142895 |
| 3597 | VESGGGVVQPGRSL | 0 | 0 | 0.006125028 |
| 3598 | LVESGGGVVQPGR | 2.93512E-05 | 0 | 0.008268391 |
| 3599 | LVESGGGVVQPGRS | 0 | 0.001437856 | 0.076774356 |
| 3600 | SGGGVVQPGR | 0 | 0 | 0.000888458 |
| 3601 | VESGGGVVQPGR | 0 | 0 | 0.00072771 |
| 3602 | VESGGGVVQPGRS | 0 | 0.000114929 | 0.004130876 |
| 3603 | LLESGGGLVQPGGSLR | 0 | 0 | 0.001441167 |
| 3604 | LVESGGGLVQPGGSL | 0 | 0.000278411 | 0.090162122 |
| 3605 | LVESGGGLVQPGGSLR | 0 | 0 | 0.022580448 |
| 3606 | VESGGGLVQPGGSL | 0 | 0 | 0.002717061 |
| 3607 | VQLVESGGGLVQPGGSLR | 0 | 0 | 0.000832737 |
| 3608 | VESGGGLVQPGGSLR | 0 | 0 | 0.003925933 |
| 3609 | IIEKASHSGMINPSR | 0 | 0 | 0.000122218 |
| 3610 | DPSEINPSMPQRT | 0 | 0 | 0.000250454 |
| 3611 | APKRLIYGATSLQSGVPS | 0 | 0 | 0.002322589 |
| 3612 | QVEHTSLDSPVT | 0 | 0.000188148 | 0 |
| 3613 | VEHTSLDSPVT | 0 | 0.000493244 | 0 |
| 3614 | DIQHAGVQYIL | 0 | 2.37712E-05 | 0.003264213 |
| 3615 | DIQHAGVQYILD | 0 | 0 | 0.003852993 |
| 3616 | DIQHAGVQYILDS | 0 | 7.3688E-05 | 0.047822147 |
| 3617 | DIQHAGVQYILDSV | 0 | 0 | 0.007493293 |
| 3618 | DIQHAGVQYILDSVI | 0 | 2.70451E-05 | 0.000832135 |
| 3619 | DIQHAGVQYILDSVIS | 5.24216E-05 | 0 | 0.001659872 |
| 3620 | GIKNDIQHAGVQYILDS | 0 | 0.000360211 | 0.002736393 |
| 3621 | IKNDIQHAGVQYILDS | 0 | 0.001449321 | 0.019364212 |
| 3622 | NDIQHAGVQYILDS | 0 | 0 | 0.003503411 |
| 3623 | GKFVIFGATSLQNTGA | 0 | 0 | 0.003227021 |
| 3624 | KFVIFGATSLQNTG | 0 | 2.78266E-05 | 0.005721369 |
| 3625 | KFVIFGATSLQNTGA | 0 | 0 | 0.002694777 |
| 3626 | ALSRSYGTPELDEDD | 0 | 0 | 0.00216519 |
| 3627 | GPGNTLAHAFAPGTG | 0 | 0 | 0.001084492 |
| 3628 | DLHAFAGSAAWD | 0 | 0 | 0.001362857 |
| 3629 | DLHAFAGSAAWDD | 0 | 0 | 0.003262428 |
| 3630 | DLHAFAGSAAWDDSA | 0 | 0 | 0.005499943 |
| 3631 | DLHAFAGSAAWDDSAR | 0 | 0.001023772 | 0.013018991 |
| 3632 | SDLHAFAGSAAWDDSAR | 0 | 0.000955867 | 0.013033792 |
| 3633 | SDLHAFAGSAAWD | 0 | 0 | 0.004445065 |
| 3634 | SDLHAFAGSAAWDD | 0 | 0 | 0.007327637 |
| 3635 | SDLHAFAGSAAWDDSA | 0 | 0 | 0.018529152 |
| 3636 | ENTYSSGSSFPVPK | 0 | 0 | 0.003083402 |
| 3637 | ENTYSSGSSFPVPKS | 0 | 0 | 0.003813955 |
| 3638 | SENTYSSGSSFPVPKS | 0 | 0.000347585 | 0.014800637 |
| 3639 | SENTYSSGSSFPVPK | 0 | 0 | 0.008130229 |
| 3640 | YETEFHPGTTSFGMS | 0 | 0 | 0.000790074 |
| 3641 | DFHAFSAGPGLFSYIR | 0 | 0 | 0.001754973 |
| 3642 | DFHAFSAGPGLFSYIRH | 0 | 0 | 0.00035579 |
| 3643 | GPGLFSYIRHWDQN | 0 | 0 | 0.000896916 |
| 3644 | KLTFTAGGAIEFGQR | 0 | 0 | 0.002280302 |
| 3645 | LPFAAAGALQGW | 0 | 0 | 0.001721316 |
| 3646 | LPFAAAGALQGWSL | 0 | 0 | 0.004140703 |
| 3647 | TLPFAAAGALQGWSLG | 0 | 5.34957E-05 | 0.008866915 |
| 3648 | TLPFAAAGALQGW | 0 | 0 | 0 |
| 3649 | PFAAAGALQGWSL | 0 | 0 | 0.007615167 |
| 3650 | LPDGPAGSWEQLIQ | 0.004306813 | 1.73611E-05 | 0 |
| 3651 | LPDGPAGSWEQLIQER | 0.017525624 | 0 | 0.000354991 |
| 3652 | LPDGPAGSWEQLIQERR | 0.005101755 | 0 | 0 |
| 3653 | AEVLGLILRY | 0 | 0.000886348 | 0 |
| 3654 | EVLGLILRY | 0 | 0.001153526 | 0.000105762 |
| 3655 | AGALAASAPVLAVAG | 0 | 0 | 0.001483844 |
| 3656 | YPHLVAGALAASAP | 0 | 0 | 0.006483671 |
| 3657 | YPHLVAGALAASAPV | 0 | 0 | 0.000418641 |
| 3658 | YPHLVAGALAASAPVL | 0 | 0 | 0.002122446 |
| 3659 | FGHTVGGAAGVVSR | 0 | 0 | 7.52067E-05 |
| 3660 | ATPGSLLPVVII | 0 | 0.000445426 | 0 |
| 3661 | ATPGSLLPVVIIA | 0 | 0.003216706 | 0 |
| 3662 | GATPGSLLPVVIIA | 0 | 0.003540871 | 0 |
| 3663 | GATPGSLLPVVII | 0 | 0.000480968 | 0 |
| 3664 | LDVTPLSLGIETAGGV | 0 | 0.000995423 | 0 |
| 3665 | LGIETAGGVMTVLIK | 0 | 0.00017554 | 0.016174518 |
| 3666 | LGIETAGGVMTVLIKR | 0 | 0 | 0.011038153 |
| 3667 | SLGIETAGGVMTVLIKR | 0 | 0 | 0.005624603 |
| 3668 | TPLSLGIETAGGVMTVLIKR | 0 | 6.50695E-05 | 0.002473833 |
| 3669 | SLGIETAGGVMTVLIK | 0 | 0 | 0.001810892 |
| 3670 | TPLSLGIETAGGVMTVLIK | 0 | 0 | 0.000730731 |
| 3671 | KGPVSVGVDARHP | 0 | 0.001142248 | 8.99567E-05 |
| 3672 | KGPVSVGVDARHPS | 0 | 0.00017269 | 0 |
| 3673 | APDEETLIALLAHAK | 0 | 8.21747E-05 | 0 |
| 3674 | ASTPVFGGILSLINEHR | 0 | 0 | 0.001813188 |
| 3675 | STPVFGGILSLINEHR | 0 | 0 | 0.011207641 |
| 3676 | TPVFGGILSLIN | 0 | 0 | 0.001026157 |
| 3677 | TPVFGGILSLINE | 0 | 0 | 0.000557317 |
| 3678 | TPVFGGILSLINEH | 0 | 3.07738E-05 | 0.003777276 |
| 3679 | TPVFGGILSLINEHR | 0 | 0 | 0.018045972 |
| 3680 | STPVFGGILSLINEHRI | 0 | 0 | 0.000670796 |
| 3681 | TPVFGGILSLINEHRI | 0.000152593 | 0 | 0.000348143 |
| 3682 | TPVFGGILSLINEHRIL | 0.000201177 | 0 | 0.000341584 |
| 3683 | YTAVVPLVY | 0.046029721 | 0.000519258 | 0 |
| 3684 | DPSETKPWAVYAGLLGG | 0 | 0 | 0.00085943 |
| 3685 | KPWAVYAGLLGGVI | 0 | 0 | 0.004652222 |
| 3686 | MPQLNTTVWPTMITPML | 0 | 0.000174905 | 0.000526817 |
| 3687 | EPSSQPTIPIV | 0 | 0.001805933 | 0 |
| 3688 | EPSSQPTIPIVG | 0 | 0.052804247 | 0 |
| 3689 | EPSSQPTIPIVGIVAGLAVL | 0 | 0 | 0.00036956 |
| 3690 | EPSSQSTVPIV | 0 | 0.014529119 | 0 |
| 3691 | EPSSQSTVPIVG | 0 | 0.217233784 | 0.000359396 |
| 3692 | EPSSQSTVPIVGIV | 0 | 0.017109231 | 0 |
| 3693 | EPSSQSTVPIVGIVA | 0.000692029 | 0.016991759 | 0 |
| 3694 | EPSSQSTVPIVGIVAG | 0.0076654 | 0.016095984 | 0 |
| 3695 | EPSSQSTVPIVGIVAGLA | 0.00096844 | 0.001468571 | 0 |
| 3696 | EPSSQSTVPIVGIVAGLAV | 8.43946E-05 | 0.000281143 | 0.00285629 |
| 3697 | QPTIPIMGIVA | 0.000669195 | 0 | 0 |
| 3698 | QPTIPIMGIVAG | 0.001880127 | 0 | 0 |
| 3699 | QPTIPIVGILAG | 0.004808566 | 4.33594E-05 | 0 |
| 3700 | IPIMGIVAGLAV | 0.00275912 | 0 | 0 |
| 3701 | IPIMGIVAGLAVL | 0.015261288 | 0 | 0 |
| 3702 | IPIVGILAG | 0.000626137 | 0 | 0 |
| 3703 | IPIVGIVAGLA | 0 | 0 | 0.001776677 |
| 3704 | IPIVGIVAGLAV | 0 | 0 | 0.036460648 |
| 3705 | IPIVGIVAGLAVL | 0 | 0.000155511 | 0.02276554 |
| 3706 | IPIVGIVAGLAVLA | 0 | 0 | 0.005334037 |
| 3707 | IPIVGIVAGLAVLAV | 0.000103294 | 0 | 0.015718837 |
| 3708 | VPIVGIVAGLAV | 0.003693032 | 8.38298E-05 | 0.008357625 |
| 3709 | VPIVGIVAGLAVL | 0.018981529 | 0.000229278 | 0.001841357 |
| 3710 | VPIVGIVAGLAVLA | 0 | 0 | 0.001007468 |
| 3711 | VPIVGIVAGLAVLAV | 0.002724271 | 0.000834255 | 0.008404939 |
| 3712 | PIVGILAGL | 0.000824008 | 0 | 0 |
| 3713 | QPTIPIMGIVAGLAVL | 0.000163486 | 0 | 0 |
| 3714 | IVAGLAVLAV | 0 | 0 | 0.000572893 |
| 3715 | PIVGIVAGLAVLAVL | 0 | 0 | 0.006033969 |
| 3716 | PIVGIVAGLAVLAVV | 0 | 0.000179446 | 0.000995291 |
| 3717 | LPKPLTLRWEPSSQPTIPIVG | 0 | 0.00056466 | 0 |
| 3718 | LRWEPSSQPTIPIV | 0 | 0.006881819 | 0 |
| 3719 | LRWEPSSQPTIPIVG | 0 | 0.097825446 | 0 |
| 3720 | LRWEPSSQSTVPIVG | 0 | 0.081411129 | 3.6701E-05 |
| 3721 | LTLRWEPSSQPTIPIVG | 0 | 0.000457155 | 0 |
| 3722 | RWEPSSQPTIPIV | 0 | 0.002747834 | 0 |
| 3723 | RWEPSSQSTVPIV | 0 | 0.005145465 | 0.000694248 |
| 3724 | WEPSSQPTIPIV | 0 | 0.01131904 | 0 |
| 3725 | WEPSSQSTVPIV | 0 | 0.006999237 | 0 |
| 3726 | LRWEPSSQPTIPIVGI | 0 | 0.000526251 | 0 |
| 3727 | LRWEPSSQPTIPIVGIL | 0 | 0.00460545 | 0 |
| 3728 | LRWEPSSQPTIPIVGILA | 0 | 0.000775213 | 0 |
| 3729 | LRWEPSSQPTIPIVGILAG | 0 | 0.002162495 | 0 |
| 3730 | LRWEPSSQPTIPIVGILAGL | 0 | 0.001643126 | 0 |
| 3731 | LRWEPSSQPTIPIVGIV | 0 | 0.000425209 | 0 |
| 3732 | LRWEPSSQSTVPIVGIV | 0 | 0.00142159 | 0.000505297 |
| 3733 | LRWEPSSQSTVPIVGIVAG | 0.002255053 | 0.009196984 | 0 |
| 3734 | RWEPSSQPTIPIVG | 0 | 0.10350293 | 0 |
| 3735 | RWEPSSQSTVPIVG | 0 | 0.096301936 | 0 |
| 3736 | WEPSSQPTIPIVG | 0 | 0.157427822 | 0.000102696 |
| 3737 | WEPSSQSTVPIVG | 0 | 0.171587622 | 0 |
| 3738 | RWEPSSQPTIPIVGIL | 0 | 0.000496155 | 0 |
| 3739 | VPIVGIIAGLVLLG | 0 | 0 | 0.000187083 |
| 3740 | VPIVGIIAGLVLLGA | 0 | 0 | 0.001190701 |
| 3741 | KPASQPTIPIV | 5.6526E-05 | 0.000823788 | 0 |
| 3742 | KPASQPTIPIVG | 0 | 0.00908275 | 0 |
| 3743 | LRWKPASQPTIPIVG | 0 | 0.003756366 | 0 |
| 3744 | WKPASQPTIPIVG | 4.79338E-05 | 0.029379646 | 0 |
| 3745 | LRWELSSQPTIPIVG | 0 | 0.011091203 | 0 |
| 3746 | RWELSSQPTIPIVG | 0.000391244 | 0.005869461 | 0 |
| 3747 | WELSSQPTIPIVG | 0 | 0.008459422 | 0.00043908 |
| 3748 | QPPEASIAVVSIPRQLPG | 0 | 0.000159261 | 0 |
| 3749 | RVVSVLTVL | 0 | 0 | 0.005405597 |
| 3750 | RVVSVLTVLHQDWLN | 0 | 0.00020785 | 0 |
| 3751 | TYRVVSVLTVLHQDW | 0 | 6.91096E-05 | 0 |
| 3752 | VVSVLTVLHQDW | 0 | 0.000206024 | 0.000543018 |
| 3753 | VVSVLTVLHQDWL | 0 | 0.00421156 | 0 |
| 3754 | VVSVLTVLHQDWLN | 0 | 0.003817903 | 0.000123415 |
| 3755 | LRAALVPLVAG | 0 | 0 | 0.003021503 |
| 3756 | VLRAALVPLVAG | 0 | 0 | 0.008186846 |
| 3757 | DEELNKLLGKVTIAQGGVLPNIQ | 0 | 0 | 0.002569747 |
| 3758 | GKVTIAQGGVLPNIQ | 0 | 0 | 0.010822429 |
| 3759 | KVTIAQGGVLPNIQ | 0 | 0 | 0.002980184 |
| 3760 | VTIAQGGVLPNIQ | 0 | 0 | 0.00448369 |
| 3761 | VTIAQGGVLPNIQAVLLPK | 0 | 0.000220472 | 0.00115225 |
| 3762 | FSIPEGALVAVVG | 0 | 0 | 0.003368351 |
| 3763 | FSIPEGALVAVVGQV | 0 | 0 | 0.002198728 |
| 3764 | HEINGLLVGF | 0 | 0.000116771 | 0 |
| 3765 | GLSERSQAVGAVLSLLL | 0.001008542 | 0 | 6.91996E-05 |
| 3766 | LPPDALVGLITFGRMVQVHE | 0 | 0 | 0.000196149 |
| 3767 | APNRDQWSMTPPMVN | 0 | 0.000928923 | 0 |
| 3768 | EPIYATIGAGVVN | 0 | 0.001724482 | 0.01008094 |
| 3769 | QPVYATIGSGIVN | 0 | 0 | 0.000473752 |
| 3770 | GITTSIVEHS | 0 | 0.00149776 | 0 |
| 3771 | GITTSIVEHSYT | 0 | 0.001088226 | 0.001243002 |
| 3772 | VTGITTSIVEHSYT | 0 | 0.003403017 | 0.030553759 |
| 3773 | VTAVGSAVVNKVPLT | 0 | 0 | 0.006196043 |
| 3774 | DFITMNPSVVQRAFG | 0.00020222 | 0.000185094 | 0.001817658 |
| 3775 | DFITMNPSVVQRAFGGF | 0 | 0.000167006 | 0.009330914 |
| 3776 | FITMNPSVVQRAFG | 0 | 0.000282764 | 0.002485587 |
| 3777 | FITMNPSVVQRAFGGF | 0 | 0 | 0.008005485 |
| 3778 | TIIDRSFRY | 0 | 0.003904995 | 0.002572047 |
| 3779 | SPAGWDVPVGALIQRGFIRPK | 0 | 0 | 0.002032547 |
| 3780 | DAFGTGIVEKL | 0 | 0.000192787 | 0 |
| 3781 | EVVDTYGSSILSIL | 0 | 0 | 0.003088708 |
| 3782 | VVDTYGSSILSIL | 0 | 0 | 0.000286297 |
| 3783 | LPLPGLLFGAPALLGL | 0 | 0 | 0.002098254 |
| 3784 | LPLPGLLFGAPALLGLA | 0 | 0 | 0.000774718 |
| 3785 | YPEQLFGAAGVMAIEHA | 0 | 0 | 0.000434489 |
| 3786 | AFNEPGSSLVSRTR | 0 | 0 | 0.001887477 |
| 3787 | FNEPGSSLVSRTR | 0 | 0 | 0.006061056 |
| 3788 | EPGSSLVSRTRVK | 0 | 0 | 0.000590882 |
| 3789 | DEFKNTGSGLLG | 0 | 0.000934883 | 0 |
| 3790 | HPATWSAGGIISRIK | 0 | 0 | 0.004128589 |
| 3791 | HPATWSAGGIISRIKS | 0 | 0 | 0.002488603 |
| 3792 | DDIAYSEDNPTPG | 0.002373745 | 0 | 0.007206769 |
| 3793 | DDIAYSEDNPTPGIV | 0.016122653 | 0 | 0.069951844 |
| 3794 | DDIAYSEDNPTPGIVI | 0.00169797 | 0 | 0.001328328 |
| 3795 | DDIAYSEDNPTPGIVIN | 0.002710177 | 0.000149763 | 0.010303202 |
| 3796 | YDDIAYSEDNPTPGIVIN | 0.001254203 | 0 | 0.007847915 |
| 3797 | IAYSEDNPTPGIV | 0.005561718 | 0 | 0.017162317 |
| 3798 | IAYSEDNPTPGIVI | 0.000136783 | 0 | 0.004286557 |
| 3799 | IAYSEDNPTPGIVIN | 0.001093396 | 0 | 0.003972047 |
| 3800 | YDDIAYSEDNPTP | 0.0081411 | 0 | 0.007979064 |
| 3801 | YDDIAYSEDNPTPG | 0.018581823 | 0 | 0.007565305 |
| 3802 | YDDIAYSEDNPTPGIV | 0.021000957 | 0 | 0.089573651 |
| 3803 | YDDIAYSEDNPTPGIVI | 0.001087183 | 0 | 0.011824576 |
| 3804 | DEQAASAISELVS | 0 | 0.003974134 | 0.002144869 |
| 3805 | LNLALNSASAIGCH | 0 | 0.000180735 | 0.000696292 |
| 3806 | LNLALNSASAIGCHVVN | 0 | 0 | 0.00130866 |
| 3807 | NLALNSASAIGCHVVN | 0 | 0 | 0.006205083 |
| 3808 | AAVAIKAMAK | 7.26628E-05 | 0.000151509 | 0 |
| 3809 | AVAIKAMAK | 0 | 0.000192824 | 3.36295E-05 |
| 3810 | ITVLSAMTEEAAVAIKAMAK | 0.000348147 | 7.33158E-05 | 0.000527061 |
| 3811 | VLSAMTEEAAVAIK | 0 | 0.000655942 | 0 |
| 3812 | QPLGPSSLAVHTILPQ | 0 | 0.001268865 | 0 |
| 3813 | GVSLKTLHPDLG | 0 | 0.002829188 | 0 |
| 3814 | KVAVKTLKPGTMS | 0 | 0.000718885 | 0 |
| 3815 | STKVAVKTLKPGTMS | 0 | 0.000875147 | 0 |
| 3816 | TKVAVKTLKPGTMS | 0 | 0.001194086 | 0 |
| 3817 | STKVAVKTLKPGTM | 0 | 0.000325139 | 0 |
| 3818 | LPWLAAATVRSVTH | 0 | 0 | 0.001018729 |
| 3819 | ALKNPPINTK | 8.98453E-06 | 0.000948168 | 0 |
| 3820 | ETVRNGGLIFAG | 0 | 0 | 0.002765341 |
| 3821 | ETVRNGGLIFAGL | 0 | 3.58017E-05 | 0.012796806 |
| 3822 | ETVRNGGLIFAGLA | 0 | 0 | 0.020328936 |
| 3823 | ETVRNGGLIFAGLAF | 0 | 0 | 0.000788906 |
| 3824 | YETVRNGGL | 0 | 0.000795214 | 3.17495E-05 |
| 3825 | YETVRNGGLI | 0 | 0.001206212 | 0 |
| 3826 | YETVRNGGLIFAGLA | 0 | 0 | 0.002104608 |
| 3827 | YYDYETVRNGGL | 0 | 0.003421143 | 0 |
| 3828 | YYDYETVRNGGLI | 0 | 0.005371551 | 0 |
| 3829 | KESPLLFKF | 0 | 0.000367671 | 0 |
| 3830 | VPMRQAAVTSTSLT | 0 | 0 | 0.001535026 |
| 3831 | VPMRQAAVTSTSLTI | 0 | 0 | 0.001298651 |
| 3832 | VPMRQAAVTSTSLTIK | 0 | 1.22283E-05 | 0.003388066 |
| 3833 | APGVSIHSATSLDSR | 0 | 0 | 0.000405931 |
| 3834 | APGVSIHSATSLDSRVQ | 0 | 0 | 0.000727173 |
| 3835 | APSKEASTEAGRAA | 0 | 0 | 0.00209621 |
| 3836 | EPPAEGGHPGAAQ | 0 | 0 | 0.000413717 |
| 3837 | QPEPPAEGGHPGAAQ | 0 | 8.94799E-06 | 0.006145145 |
| 3838 | QPEPPAEGGHPGAAQR | 0 | 0 | 0.001240641 |
| 3839 | QPEPPAEGGHPGAAQRG | 0 | 0 | 0.00513745 |
| 3840 | QPEPPAEGGHPGAAQRGQ | 0 | 0.000164669 | 0.001216327 |
| 3841 | QPEPPAEGGHPGAAQRGQG | 0 | 0 | 0.003162435 |
| 3842 | QPEPPAEGGHPGAAQRGQGD | 0 | 0 | 0.00073429 |
| 3843 | QPEPPAEGGHPGAAQRGQGDL | 0 | 0 | 0.004509688 |
| 3844 | IPEVKVHSGRGAGAES | 0 | 0 | 0.018472442 |
| 3845 | IPEVKVHSGRGAGAESS | 0 | 0 | 0.009882335 |
| 3846 | IPEVKVHSGRGAGAESSHPV | 0 | 0 | 0.000802701 |
| 3847 | DPLPAHSGALPFPSPGP | 0 | 0 | 0.011399189 |
| 3848 | DPLPAHSGALPFPSPGPP | 0 | 0 | 0.023451244 |
| 3849 | DPLPAHSGALPFPSPGPPQ | 0 | 0 | 0.007274949 |
| 3850 | DPLPAHSGALPFPSPGPPQPP | 0 | 0 | 0.003754829 |
| 3851 | DPLPAHSGALPFPSPGPPQPPHPP | 0 | 0 | 0.018928655 |
| 3852 | DVWGKGTTVTVS | 0 | 2.77094E-05 | 0.013806608 |
| 3853 | DVWGKGTTVTVSS | 0.000128794 | 0 | 0.002708244 |
| 3854 | MDVWGKGTTVTVSS | 0.000196276 | 0.001133577 | 0.009525706 |
| 3855 | YMDVWGKGTTVTVSS | 0 | 0.000591956 | 0.008142145 |
| 3856 | YMDVWGKGTTVTVS | 0 | 0 | 0.014630423 |
| 3857 | GPRPAAIAVAG | 3.74462E-05 | 0 | 0.013729328 |
| 3858 | GPRPAAIAVAGFS | 2.70182E-05 | 0 | 0.018885299 |
| 3859 | GPRPAAIAVAGFSN | 0.00019334 | 0 | 0.00087639 |
| 3860 | GPRPAAIAVAGFSNWTS | 0 | 0 | 0.006935535 |
| 3861 | GPRPAAIAVAGFSNWTSN | 0 | 0 | 0.000871404 |
| 3862 | GPRPAAIAVAGFSNWTSNF | 0.005517265 | 0.000116091 | 0.004749884 |
| 3863 | GPRPAAIAVAGFSNWTSNFI | 0 | 0 | 0.001117834 |
| 3864 | QGPRPAAIAVAG | 0 | 0 | 0.011490858 |
| 3865 | QGPRPAAIAVAGFS | 0 | 0 | 0.009285487 |
| 3866 | VPRLWPRAGAVA | 0 | 0 | 0.001408086 |
| 3867 | VPRLWPRAGAVAER | 0 | 0 | 0.000796848 |
| 3868 | VPRLWPRAGAVAERL | 0 | 0 | 0.000871755 |
| 3869 | VPRLWPRAGAVAERLW | 0 | 0.000311513 | 0.004883648 |
| 3870 | VPRLWPRAGAVAERLWS | 0 | 0 | 0.000491421 |
| 3871 | HPTTLAVGICSPAS | 0 | 0 | 0.003529891 |
| 3872 | HPTTLAVGICSPASR | 3.35105E-05 | 0 | 0.005928069 |
| 3873 | HPTTLAVGICSPASRS | 8.10632E-05 | 0 | 0.01165515 |
| 3874 | RYRPGTVAL | 0.000388224 | 0.000148037 | 0.002465741 |
| 3875 | YRPGTVALR | 0.000717207 | 0.000724107 | 0.001483663 |
| 3876 | GPTTYKVTSTLTIK | 6.59611E-05 | 0.049222261 | 0 |
| 3877 | GPTTYKVTSTLTIKE | 0 | 0.01526514 | 0 |
| 3878 | GPTTYKVTSTLTIKES | 0 | 0.003988521 | 0 |
| 3879 | GPTTYKVTSTLTIKESD | 0 | 0.004792729 | 0 |
| 3880 | SGPTTYKVTSTLTIK | 0 | 0.00699057 | 0.000303218 |
| 3881 | KVTSTLTIKESDWL | 0 | 0.005732435 | 0 |
| 3882 | IDKKSMIASTAITVH | 0 | 0.00055949 | 0.010010873 |
| 3883 | IDKKSMIASTAITVHY | 0 | 0.000370135 | 0.013852987 |
| 3884 | FNVRIATTALAIY | 0 | 0 | 0.001007821 |
| 3885 | NVRIATTALAIY | 0 | 0.001395724 | 0.002126857 |
| 3886 | NVRIATTALAIYH | 7.3503E-05 | 0.008050735 | 0.003397457 |
| 3887 | NVRIATTALAIYHV | 8.90102E-05 | 0.000257847 | 8.10758E-05 |
| 3888 | RVKLPSGSK | 6.45672E-06 | 0.000349415 | 0 |
| 3889 | RVKLPSGSKK | 0 | 0.000384757 | 0 |
| 3890 | AKDVKFGAD | 4.53081E-06 | 0.0002853 | 0.00013081 |
| 3891 | ELKWGTDEAQFIYIL | 0 | 0.000631835 | 0 |
| 3892 | GELKWGTDEAQFIYIL | 0 | 0.001334584 | 0.000463016 |
| 3893 | ELKYSTNEGETWK | 2.30101E-05 | 0.00517615 | 0 |
| 3894 | ELKYSTNEGETWKT | 0 | 0.000894473 | 0 |
| 3895 | NELKYSTNEGETWKT | 0 | 0.004749951 | 0.000153666 |
| 3896 | GGSAEDSVGSSSVTVVLL | 0 | 0 | 0.000658708 |
| 3897 | SGGSAEDSVGSSSVTVVLL | 0 | 0.00019365 | 0.000555077 |
| 3898 | HDIKYGSGSGQQSVTG | 9.73662E-06 | 0.000127285 | 0.000946645 |
| 3899 | DIHYGSGSLSG | 4.37887E-05 | 0 | 0.008540525 |
| 3900 | DIHYGSGSLSGYL | 0 | 0 | 0.006763964 |
| 3901 | FDIHYGSGSLSGYL | 0 | 0 | 0.011010704 |
| 3902 | IHYGSGSLSG | 0 | 0 | 0.002480202 |
| 3903 | SFDIHYGSGSLSGYL | 0 | 0 | 0.019662755 |
| 3904 | TSFDIHYGSGSLSGYL | 0 | 0 | 0.094123836 |
| 3905 | SFDIHYGSGSLSGY | 0 | 0 | 0.002506575 |
| 3906 | TSFDIHYGSGSLSGY | 0 | 0 | 0.006585173 |
| 3907 | FDIHYGSGSLSG | 0 | 0 | 0.006823017 |
| 3908 | GTSFDIHYGSGSLSG | 0 | 0 | 0.024334407 |
| 3909 | SFDIHYGSGSLSG | 0 | 0 | 0.046841572 |
| 3910 | TSFDIHYGSGSLSG | 0 | 0 | 0.171955964 |
| 3911 | GTSFDIHYGSGSLS | 0 | 0 | 0.028103736 |
| 3912 | TSFDIHYGSGSLS | 0 | 0 | 0.021677554 |
| 3913 | SPFPSSSFSPGGLAPEISP | 0.01049955 | 0 | 0 |
| 3914 | SPFPSSSFSPGGLAPEISPL | 0.012555471 | 0 | 0 |
| 3915 | SPFPSSSFSPGGLAPEISPLE | 0.015197643 | 0 | 0 |
| 3916 | LPAPGVPAWAREIS | 0.000363897 | 0 | 0.001716104 |
| 3917 | FIVWMRTAALPTFR | 0 | 0.001154914 | 0 |
| 3918 | RVKGPGISKF | 0 | 0.001428465 | 2.21805E-05 |
| 3919 | ALIKAAGVNVEPFWP | 0 | 0 | 0.002279897 |
| 3920 | INALIKAAGVNVEP | 0 | 0 | 0.001761796 |
| 3921 | INALIKAAGVNVEPFW | 0 | 0 | 0 |
| 3922 | DEDGVVRGASIP | 0 | 0 | 0 |
| 3923 | DEDGVVRGASIPFQ | 0.000770622 | 0 | 0.034597591 |
| 3924 | DEDGVVRGASIPFQF | 0.001019029 | 0 | 0.008935542 |
| 3925 | DEDGVVRGASIPFQFR | 0 | 0.00047905 | 0.027064945 |
| 3926 | DEDGVVRGASIPFQFRP | 0 | 2.52469E-05 | 0.021692301 |
| 3927 | DEDGVVRGASIPFQFRPE | 0 | 0.002273309 | 0.019726608 |
| 3928 | EDGVVRGASIP | 1.70818E-05 | 0 | 0.006953761 |
| 3929 | GVVRGASIP | 0 | 0 | 0.000578883 |
| 3930 | VDEDGVVRGASIPFQFRPE | 0.001592196 | 0.000250004 | 0.015456473 |
| 3931 | VDEDGVVRGASIPFQFRPEN | 0 | 0 | 0.000657385 |
| 3932 | EDGVVRGASIPFQ | 0.000762251 | 0 | 0.02013473 |
| 3933 | EDGVVRGASIPFQF | 0 | 0 | 0.008548578 |
| 3934 | VDEDGVVRGASIPFQF | 0.00041735 | 4.08217E-05 | 0.003858445 |
| 3935 | VDEDGVVRGASIPFQFR | 0.000254496 | 0 | 0.016483565 |
| 3936 | VDEDGVVRGASIPFQFRP | 0.001028124 | 0 | 0.008833054 |
| 3937 | VDEDGVVRGASIP | 0.000726487 | 0 | 0.006866759 |
| 3938 | VDEDGVVRGASIPF | 0 | 0.000283926 | 0.004050535 |
| 3939 | VDEDGVVRGASIPFQ | 0.003129705 | 0 | 0.025583022 |
| 3940 | DGVVRGASIPFQFR | 0 | 0 | 0.000216921 |
| 3941 | EDGVVRGASIPFQFR | 0 | 0 | 0.011200116 |
| 3942 | EDGVVRGASIPFQFRP | 0 | 0 | 0.007688024 |
| 3943 | EDGVVRGASIPFQFRPE | 0 | 0 | 0.003046242 |
| 3944 | GVVRGASIPFQ | 0.000121757 | 0 | 0.014724703 |
| 3945 | GVVRGASIPFQF | 0 | 0 | 0.007390945 |
| 3946 | GVVRGASIPFQFR | 0 | 0 | 0.010709955 |
| 3947 | GVVRGASIPFQFRP | 0 | 0 | 0.007469818 |
| 3948 | GVVRGASIPFQFRPE | 0 | 0 | 0.00317593 |
| 3949 | IPGTVVRTSTIPEELG | 0 | 0.000808246 | 0 |
| 3950 | EGPMIHSGSVIAAGIS | 4.97331E-05 | 0 | 0.005646889 |
| 3951 | EGPMIHSGSVVG | 0 | 0 | 0.026043384 |
| 3952 | GPMIHSGSVIAAGIS | 0 | 0 | 0.002320894 |
| 3953 | RKTVVHGGSLIPH | 0 | 0 | 0.000147122 |
| 3954 | APVYVVRSGGLVKTRS | 0 | 0 | 0.00032808 |
| 3955 | YDLGKGAAVIRSR | 0 | 0 | 0.000450269 |
| 3956 | YDLGKGAAVIRSREP | 0 | 0 | 0.005721393 |
| 3957 | DQESGAVIHPATQ | 0 | 0 | 0.00019831 |
| 3958 | LDQESGAVIHPATQ | 9.94054E-05 | 0 | 0.001368455 |
| 3959 | LDQESGAVIHPATQT | 0 | 3.43567E-05 | 0.002463993 |
| 3960 | LDQESGAVIHPAT | 0 | 0 | 0.002048258 |
| 3961 | KPGDLESAPVLRTSGEG | 0 | 0 | 0.010865621 |
| 3962 | NEDNGIIKAF | 0 | 0.006398674 | 0 |
| 3963 | EPTYHGIVTENDNTVL | 0 | 0.001450175 | 0 |
| 3964 | YHGIVTENDNTVL | 0 | 0.001520999 | 0 |
| 3965 | YHGIVTENDNTVLL | 0 | 0.001808485 | 0 |
| 3966 | LEPTYHGIVTENDNTV | 0.002755369 | 0 | 0 |
| 3967 | ALPHVHTVSYGDDED | 0 | 0.001158814 | 0 |
| 3968 | LPHVHTVSYGDDED | 0 | 0.00377118 | 0.003484194 |
| 3969 | LPHVHTVSYGDDEDS | 0 | 0.006263033 | 2.85791E-05 |
| 3970 | LPHVHTVSYGDDEDSLS | 0 | 0.002323751 | 0 |
| 3971 | LHGYAFSQDENGIVS | 0 | 0.004077114 | 0.001108192 |
| 3972 | LPKDDEYYQFCYVDE | 0.005853421 | 0 | 0 |
| 3973 | LPKDDEYYQFCYVDED | 0.010241003 | 0 | 0 |
| 3974 | LPKDDEYYQFCYVDEDG | 0.008223498 | 0.000191276 | 0 |
| 3975 | LPKDDEYYQFCYVDEDGVV | 0.011185988 | 0 | 0 |
| 3976 | LPKDDEYYQFCYVDEDGVVR | 0.002105495 | 0 | 0 |
| 3977 | ADMDQFTASISETPVDVR | 0.000775295 | 0 | 0.00016156 |
| 3978 | DQFTASISETPVDVR | 0 | 0.007691632 | 0.000265536 |
| 3979 | VLTNSPVELR | 0 | 0.000187971 | 0.000142128 |
| 3980 | VLTNSPVELREPN | 0 | 0.000525756 | 0.000499851 |
| 3981 | VPPEVTVLTNSPVELREPN | 0.003587042 | 0.128616465 | 0 |
| 3982 | VPPEVTVLTNSPVELREPNV | 0.000118463 | 0.001667831 | 0 |
| 3983 | VPPEVTVLTNSPVELREPNVL | 0.000368134 | 0.003470739 | 0 |
| 3984 | VPPEVTVLTNSPVE | 0 | 0.005701426 | 0 |
| 3985 | VPPEVTVLTNSPVEL | 0 | 0.0020323 | 0.000399377 |
| 3986 | VPPEVTVLTNSPVELR | 0.000849383 | 0.077746999 | 0 |
| 3987 | VPPEVTVLTNSPVELREP | 5.76552E-05 | 0.010936057 | 0 |
| 3988 | LPNPSVDVKGIGDELYNPE | 0.011941282 | 0.002877972 | 0 |
| 3989 | PSVDVKGIGDELYNPE | 0.015389802 | 0.006052879 | 0.000170817 |
| 3990 | VPVIADGGIQNVGHI | 0 | 0.000354917 | 0.00379696 |
| 3991 | DGEDWAGGLPWG | 0 | 0 | 0.002356472 |
| 3992 | DGEDWAGGLPWGGRS | 0 | 0 | 0.00076972 |
| 3993 | DGEDWAGGLPWGGRSP | 0 | 0 | 0.01003568 |
| 3994 | DGEDWAGGLPWGGRSPG | 0 | 0 | 0.145487397 |
| 3995 | DGEDWAGGLPWGGRSPGG | 0.000118604 | 0 | 0.02199881 |
| 3996 | EDWAGGLPWG | 0 | 0 | 0.000375934 |
| 3997 | GEDWAGGLPWGGRSP | 0.001543595 | 0 | 0.004420901 |
| 3998 | EDWAGGLPWGGRSP | 0 | 0 | 0.00661867 |
| 3999 | EDWAGGLPWGGRSPG | 0.001301031 | 0 | 0.048597598 |
| 4000 | GEDWAGGLPWGGRSPG | 0 | 0 | 0.057600162 |
| 4001 | DWAGGLPWGGRSP | 0 | 0 | 0.000730067 |
| 4002 | DWAGGLPWGGRSPG | 0 | 0 | 0.022409899 |
| 4003 | DWAGGLPWGGRSPGG | 0 | 0 | 0.001227445 |
| 4004 | EDWAGGLPWGGRSPGG | 0 | 0 | 0.024399854 |
| 4005 | GEDWAGGLPWGGRSPGG | 0 | 0 | 0.003952622 |
| 4006 | WAGGLPWGGRSP | 0 | 0 | 0.011018987 |
| 4007 | WAGGLPWGGRSPG | 0 | 0 | 0.035891609 |
| 4008 | WAGGLPWGGRSPGG | 0 | 0 | 0.009979705 |
| 4009 | AESALQLLYT | 0 | 8.38775E-05 | 0 |
| 4010 | VPPVEVVYSQEPGAQPD | 0.001665552 | 0.00414575 | 0 |
| 4011 | VPPVEVVYSQEPGAQPDL | 0 | 0.006622748 | 0 |
| 4012 | VPPVEVVYSQEPGAQPDLA | 0.000477028 | 0.003227362 | 0 |
| 4013 | TGVDFTGTTVGFAR | 0 | 0 | 0.001055431 |
| 4014 | APPSYEETVAVNSYYPT | 0 | 0.001158322 | 0 |
| 4015 | APPSYEETVAVNSYYPTPP | 0 | 0.020981068 | 0 |
| 4016 | APSAPPSYEETVAVNSYYPTPP | 0 | 0.004734165 | 0 |
| 4017 | DRTESRSTEPELIQ | 0.000117556 | 0.000425357 | 0.000175026 |
| 4018 | RTESRSTEPELIQ | 0 | 0.015429957 | 0 |
| 4019 | TESRSTEPELIQ | 0 | 0.000429259 | 0 |
| 4020 | VDRTESRSTEPELIQ | 0 | 0.02010954 | 5.14328E-05 |
| 4021 | VDRTESRSTEPELIQVK | 0 | 0.002420059 | 0 |
| 4022 | RTESRSTEPELIQVK | 0 | 0.000674255 | 0 |
| 4023 | LPSKSTDADLAR | 0 | 0.002568607 | 0 |
| 4024 | LPSYEEALSLPSKTP | 0 | 0 | 0.001725145 |
| 4025 | LPSYEEALSLPSKTPE | 0 | 0 | 0.026897587 |
| 4026 | LPSYEEALSLPSKTPEG | 0 | 3.88925E-05 | 0.144001656 |
| 4027 | LPSYEEALSLPSKTPEGGPA | 0 | 0 | 0.001568626 |
| 4028 | LPSYEEALSLPSKTPEGGPAPPP | 0 | 0 | 0.09329085 |
| 4029 | YEEALSLPSKTPEG | 7.17952E-05 | 0 | 0.004204677 |
| 4030 | YEEALSLPSKTPEGGPAPPP | 0 | 0 | 0.002380452 |
| 4031 | VVLPSYEEALSLPSK | 0.004524795 | 0 | 0 |
| 4032 | VVLPSYEEALSLPSKTPE | 0.009025584 | 0 | 0 |
| 4033 | VVLPSYEEALSLPSKTPEG | 0 | 0 | 0.000819289 |
| 4034 | GPPPSGAYLVAGNAQMSH | 0 | 0 | 0.005211741 |
| 4035 | LENIYPDSQIPRHY | 0.002986369 | 0.000112407 | 0 |
| 4036 | NIYPDSQIPRHY | 0.000542179 | 0 | 0 |
| 4037 | VDATAETDLAKRFDVS | 0 | 0.000727525 | 0 |
| 4038 | IETTPESRYL | 0 | 8.68282E-05 | 0 |
| 4039 | IKEEVVGLTETSSQPK | 0.001542627 | 0 | 0 |
| 4040 | NPKQAATQSELHY | 0 | 0.006240158 | 4.73904E-05 |
| 4041 | NPKQAATQSELHYA | 0 | 0.005834896 | 0 |
| 4042 | NPKQAATQSELHYAN | 0 | 0.002614286 | 0 |
| 4043 | QNPKQAATQSELHY | 0 | 0 | 0 |
| 4044 | VPGSQAQLKEVWEELDGLDPN | 0.002327403 | 0 | 0 |
| 4045 | AASVTFSLLVSGSQD | 0 | 0.001357899 | 0 |
| 4046 | ASVTFSLLVSGSQD | 0 | 0.001596255 | 0 |
| 4047 | AAVVVPSGEEQRYT | 0.008944118 | 0.002204049 | 0 |
| 4048 | AAVVVPSGQEQRYT | 0.004216705 | 0 | 0 |
| 4049 | ASVVVPSGQEQR | 1.39756E-05 | 0.000251327 | 0 |
| 4050 | GDGTFQKWASVVVPSGQEQRYT | 0 | 0.000467458 | 0 |
| 4051 | GTFQKWAAVVVPSGEEQRYT | 0 | 0 | 0.001037194 |
| 4052 | RPAGDGTFQKWAAVVVPSGEEQRYT | 0.000134787 | 0.000100109 | 0.012104044 |
| 4053 | RPAGDGTFQKWASVVVPSGQEQRYT | 0 | 0.003022886 | 0 |
| 4054 | TFQKWAAVVVPSGEEQRYT | 0 | 0 | 0.000819321 |
| 4055 | VVVPSGEEQ | 0.001594633 | 0 | 0 |
| 4056 | VVVPSGEEQR | 0.000289686 | 0 | 0.000193407 |
| 4057 | VVVPSGQEQ | 0.000435445 | 0.0001986 | 0 |
| 4058 | AVVVPSGEEQRY | 0 | 0.004681553 | 0 |
| 4059 | AVVVPSGEEQRYT | 0.000942774 | 0.009203941 | 0 |
| 4060 | GDRTFQKWAAVVVPSGEEQRYT | 0 | 0 | 0.0006188 |
| 4061 | RPAGDRTFQKWAAVVVPSGEEQRYT | 0 | 0 | 0.003301329 |
| 4062 | SVVVPSGQEQRY | 0.000501573 | 0.000157089 | 0 |
| 4063 | SVVVPSGQEQRYT | 0 | 0.001173385 | 0 |
| 4064 | VPSGEEQRYT | 0 | 0.000470096 | 0 |
| 4065 | VVVPSGEEQRYT | 0.001565421 | 0.002864618 | 3.41679E-05 |
| 4066 | AGDGTFQKWAAVVVPSGEEQ | 0 | 0 | 0.001442626 |
| 4067 | AGDGTFQKWASVVVPSG | 0.000116534 | 0.00502861 | 0 |
| 4068 | DGTFQKWAAVVVP | 0.004671014 | 0 | 0 |
| 4069 | DGTFQKWAAVVVPS | 0.004757322 | 0.003635765 | 0 |
| 4070 | DGTFQKWAAVVVPSG | 0.037761397 | 0.0069076 | 0.010893682 |
| 4071 | DGTFQKWASVVVP | 0 | 0.003766737 | 0 |
| 4072 | DGTFQKWASVVVPS | 0 | 0.006513053 | 0 |
| 4073 | DGTFQKWASVVVPSG | 3.31174E-05 | 0.068642552 | 7.45428E-05 |
| 4074 | FQKWAAVVVP | 0.054470597 | 0 | 0 |
| 4075 | FQKWASVVVP | 0.000388168 | 0.022501607 | 0 |
| 4076 | GDGTFQKWAAVVVPSG | 0.033045401 | 0.015339765 | 0.012159325 |
| 4077 | GDGTFQKWASVVVPSG | 0.00069696 | 0.061169145 | 7.15108E-05 |
| 4078 | GTFQKWAAVVVP | 0.010353355 | 0.001128932 | 0 |
| 4079 | GTFQKWASVVVP | 0 | 0.009258881 | 0 |
| 4080 | KWASVVVP | 0 | 0.001153172 | 0 |
| 4081 | PAGDGTFQKWAAVVVPSGEEQ | 0.004909353 | 0 | 0.006506395 |
| 4082 | PAGDGTFQKWAAVVVPSGEEQR | 0 | 0.000681153 | 0.005427367 |
| 4083 | PAGDGTFQKWASVVVPSGQEQ | 0 | 0.00209624 | 0.000746867 |
| 4084 | QKWAAVVVP | 0.003861238 | 0 | 0 |
| 4085 | QKWASVVVP | 0 | 0.000592178 | 0 |
| 4086 | RPAGDGTFQKWAAVVVPSGEEQR | 0.000181137 | 0.001110692 | 0.058951393 |
| 4087 | RPAGDGTFQKWAAVVVPSGEEQRY | 0 | 0 | 0.004547605 |
| 4088 | RPAGDGTFQKWAAVVVPSGQEQR | 0.017304341 | 0 | 0.010655868 |
| 4089 | RPAGDGTFQKWASVVVPSGQEQR | 0.000952127 | 0.008776155 | 0 |
| 4090 | TFQKWAAVVVP | 0.091059543 | 0 | 0 |
| 4091 | TFQKWASVVVP | 0.001396404 | 0.008548327 | 0 |
| 4092 | GTFQKWAAVVVPS | 0.037760043 | 0.006688803 | 0.001107175 |
| 4093 | GTFQKWAAVVVPSG | 0.059395579 | 0.022830094 | 0.003131849 |
| 4094 | GTFQKWAAVVVPSGEE | 0.026954442 | 0.000695773 | 0.003726049 |
| 4095 | GTFQKWAAVVVPSGEEQ | 0.010317253 | 0.000441624 | 0.002055528 |
| 4096 | GTFQKWAAVVVPSGQE | 0.013925303 | 0 | 0.002330489 |
| 4097 | GTFQKWAAVVVPSGQEQ | 0.003289125 | 0 | 0.000461822 |
| 4098 | GTFQKWASVVVPS | 0 | 0.017927958 | 0 |
| 4099 | GTFQKWASVVVPSG | 0 | 0.169463953 | 0 |
| 4100 | GTFQKWASVVVPSGQE | 0 | 0.056219553 | 0.0027538 |
| 4101 | GTFQKWASVVVPSGQEQ | 0 | 0.027742565 | 0.000215271 |
| 4102 | TFQKWAAVVVPS | 0.058189637 | 0 | 0 |
| 4103 | TFQKWASVVVPS | 0 | 0.003619668 | 0 |
| 4104 | DGTFQKWAAVVVPSGEE | 0.01321943 | 0.000197737 | 0.013643699 |
| 4105 | DGTFQKWAAVVVPSGEEQ | 0.005626202 | 0 | 0.040565335 |
| 4106 | DGTFQKWAAVVVPSGEEQR | 0 | 0 | 0.041251885 |
| 4107 | DGTFQKWAAVVVPSGEEQRY | 0 | 0 | 0.001775913 |
| 4108 | DGTFQKWAAVVVPSGQE | 0.004524423 | 0.00017095 | 0.002546253 |
| 4109 | DGTFQKWAAVVVPSGQEQ | 0 | 0 | 0.002491868 |
| 4110 | DGTFQKWAAVVVPSGQEQR | 0 | 0 | 0.002411168 |
| 4111 | DGTFQKWASVVVPSGQ | 0 | 0.00914986 | 0 |
| 4112 | DGTFQKWASVVVPSGQE | 0 | 0.016812702 | 0.001837468 |
| 4113 | DGTFQKWASVVVPSGQEQ | 0 | 0.013619737 | 0.0015848 |
| 4114 | DGTFQKWASVVVPSGQEQR | 0.000812593 | 0.001489568 | 0 |
| 4115 | FQKWAAVVVPS | 0.032997309 | 3.64403E-05 | 0 |
| 4116 | FQKWAAVVVPSG | 0.242332408 | 0 | 0.017132975 |
| 4117 | FQKWAAVVVPSGE | 0.005190098 | 0 | 0.012885645 |
| 4118 | FQKWAAVVVPSGQ | 0.001896489 | 0.000129291 | 0.004852508 |
| 4119 | FQKWASVVVPS | 0.000129029 | 0.00679034 | 0 |
| 4120 | FQKWASVVVPSG | 0.002071206 | 0.416334343 | 0 |
| 4121 | FQKWASVVVPSGQ | 0 | 0.046944166 | 5.80576E-05 |
| 4122 | GDGTFQKWAAVVVPSGEEQRY | 0 | 0 | 0.032564548 |
| 4123 | GTFQKWAAVVVPSGE | 0 | 0.00053213 | 0 |
| 4124 | GTFQKWASVVVPSGQ | 0 | 0.025221833 | 0.000189173 |
| 4125 | QKWAAVVVPS | 0.00425332 | 0 | 0 |
| 4126 | TFQKWAAVVVPSGE | 0.008501577 | 0.000385002 | 0.005528464 |
| 4127 | TFQKWAAVVVPSGQ | 0.00279678 | 0.000870813 | 0.001264061 |
| 4128 | TFQKWASVVVPSGQ | 0 | 0.034994339 | 0 |
| 4129 | PAGDGTFQKWAAVVVPSG | 0.018954132 | 0 | 0 |
| 4130 | PAGDGTFQKWAAVVVPSGEE | 0.004462742 | 0 | 0.001219595 |
| 4131 | PAGDGTFQKWASVVVPSG | 0 | 0.008771616 | 0.000424895 |
| 4132 | PAGDGTFQKWASVVVPSGQ | 0 | 0.000718482 | 0 |
| 4133 | PAGDGTFQKWASVVVPSGQE | 0 | 0.001315883 | 0 |
| 4134 | QKWAAVVVPSG | 0.040787959 | 0 | 0.000576459 |
| 4135 | QKWASVVVPSG | 0.000153838 | 0.030056646 | 0 |
| 4136 | RPAGDGTFQKWAAVVVPSGEE | 0.035893359 | 0.008865926 | 0.038329967 |
| 4137 | RPAGDGTFQKWAAVVVPSGQE | 0.014984035 | 0 | 0.01539579 |
| 4138 | RPAGDGTFQKWASVVVPSGQE | 0 | 0.03733036 | 0 |
| 4139 | TFQKWAAVVVPSG | 0.363376854 | 0.001644653 | 0.009584701 |
| 4140 | TFQKWASVVVPSG | 0.002678896 | 0.211564481 | 0 |
| 4141 | GDGTFQKWAAVVVPS | 0.00367078 | 0.001813037 | 0 |
| 4142 | GDGTFQKWAAVVVPSGE | 0 | 0.000703389 | 0.002334307 |
| 4143 | GDGTFQKWAAVVVPSGEE | 0.009209383 | 0 | 0.011601232 |
| 4144 | GDGTFQKWAAVVVPSGEEQ | 0.004099849 | 0 | 0.050995274 |
| 4145 | GDGTFQKWAAVVVPSGQEQ | 0.000418026 | 0 | 0.002637586 |
| 4146 | GDGTFQKWASVVVP | 0.000153577 | 0.000939344 | 0 |
| 4147 | GDGTFQKWASVVVPS | 0 | 0.002978706 | 0 |
| 4148 | GDGTFQKWASVVVPSGQ | 0 | 0.011027583 | 0 |
| 4149 | GDGTFQKWASVVVPSGQE | 0 | 0.01351069 | 0 |
| 4150 | GDGTFQKWASVVVPSGQEQ | 0 | 0.009795541 | 0 |
| 4151 | RPAGDGTFQKWAAVVVPSGEEQ | 0.031632015 | 0.008804709 | 0.110489601 |
| 4152 | RPAGDGTFQKWAAVVVPSGQEQ | 0.010044407 | 0 | 0.021101873 |
| 4153 | RPAGDGTFQKWASVVVPSGQEQ | 0 | 0.027847421 | 0.002095007 |
| 4154 | TRPAGDGTFQKWAAVVVPSGEEQ | 0.000602718 | 0 | 0.002561832 |
| 4155 | RTFQKWAAVVVPSGEE | 0.010958523 | 0.000388409 | 0.002878222 |
| 4156 | TFQKWAAVVVPSGEE | 0.310911795 | 0 | 0.0533657 |
| 4157 | TFQKWAAVVVPSGQE | 0.065729232 | 0 | 0.012574762 |
| 4158 | TFQKWASVVVPSGQE | 0 | 0.144154619 | 0.000337505 |
| 4159 | QKWAAVVVPSGEEQ | 0.001586698 | 0.000312084 | 0.012843012 |
| 4160 | QKWAAVVVPSGQEQ | 0 | 0 | 0.001698779 |
| 4161 | QKWASVVVPSGQ | 0 | 0.000824927 | 0 |
| 4162 | QKWASVVVPSGQEQ | 0 | 0.004241412 | 5.27197E-05 |
| 4163 | RTFQKWAAVVVPSGEEQ | 0.003236525 | 0.000446647 | 0.002265734 |
| 4164 | TFQKWAAVVVPSGEEQ | 0.050017836 | 0 | 0.082388436 |
| 4165 | TFQKWAAVVVPSGQEQ | 0.00751832 | 0 | 0.015865456 |
| 4166 | TFQKWASVVVPSGQEQ | 0 | 0.038130762 | 0.002080467 |
| 4167 | FQKWAAVVVPSGEE | 0.269238625 | 0.000565097 | 0.193862025 |
| 4168 | FQKWAAVVVPSGEEQ | 0.022183896 | 0.000522864 | 0.195032015 |
| 4169 | FQKWAAVVVPSGEEQR | 0.001204811 | 0 | 0.075478388 |
| 4170 | FQKWAAVVVPSGQE | 0.043828081 | 4.50885E-05 | 0.048910043 |
| 4171 | FQKWAAVVVPSGQEQ | 0.003140613 | 0 | 0.039042866 |
| 4172 | FQKWAAVVVPSGQEQR | 0 | 0 | 0.012644145 |
| 4173 | FQKWASVVVPSGQE | 0.000793085 | 0.270142365 | 0.003385228 |
| 4174 | FQKWASVVVPSGQEQ | 0 | 0.038719419 | 0.002662537 |
| 4175 | FQKWASVVVPSGQEQR | 0 | 0.012268654 | 0.001412179 |
| 4176 | GDGTFQKWAAVVVPSGEEQR | 0 | 0 | 0.033589178 |
| 4177 | GDGTFQKWAAVVVPSGQEQR | 8.05356E-05 | 0 | 0.002707098 |
| 4178 | GTFQKWAAVVVPSGEEQR | 0.000164937 | 0 | 0.004963261 |
| 4179 | GTFQKWASVVVPSGQEQR | 0 | 0.007340235 | 0 |
| 4180 | KWAAVVVPSG | 0.103778285 | 0 | 3.61409E-05 |
| 4181 | KWAAVVVPSGE | 0.003255635 | 0 | 1.54985E-05 |
| 4182 | KWAAVVVPSGEE | 0.055663049 | 0 | 0.00045951 |
| 4183 | KWAAVVVPSGQE | 0.015463855 | 0 | 0.000281638 |
| 4184 | KWASVVVPSG | 0.003553056 | 0.181066733 | 0.002813528 |
| 4185 | KWASVVVPSGQ | 0.000183914 | 0.01001304 | 0 |
| 4186 | KWASVVVPSGQE | 9.69741E-05 | 0.058195004 | 0.000387407 |
| 4187 | QKWAAVVVPSGEE | 0.009006589 | 0 | 0.003053826 |
| 4188 | QKWAAVVVPSGQE | 0 | 0 | 0.001567122 |
| 4189 | QKWASVVVPSGQE | 0 | 0.004316284 | 0 |
| 4190 | TFQKWAAVVVPSGEEQR | 0.00216298 | 0 | 0.047077115 |
| 4191 | TFQKWAAVVVPSGQEQR | 0 | 0 | 0.007360452 |
| 4192 | TFQKWASVVVPSGQEQR | 0 | 0.00974992 | 0.004404831 |
| 4193 | TRPAGDGTFQKWAAVVVPSGEEQR | 0 | 0 | 0.002028663 |
| 4194 | WASVVVPSG | 0 | 0.001176301 | 0 |
| 4195 | KWAAVVVPSGEEQ | 0.005349298 | 0 | 0.000642978 |
| 4196 | KWAAVVVPSGQEQ | 0.00145238 | 0 | 0.004112405 |
| 4197 | KWASVVVPSGQEQ | 0 | 0.016469371 | 0.004069668 |
| 4198 | KWASVVVPSGQEQR | 0 | 0.001819369 | 0.000158386 |
| 4199 | QKWAAVVVPSGEEQR | 0 | 0.000255424 | 0.007062214 |
| 4200 | WAAVVVPSGEE | 0.004756509 | 0 | 0 |
| 4201 | WASVVVPSGQEQ | 0 | 0.001384145 | 9.04713E-05 |
| 4202 | DRTFQKWAAVVVP | 0.009034025 | 0 | 0 |
| 4203 | DRTFQKWAAVVVPS | 0.00631024 | 0.00087371 | 0 |
| 4204 | DRTFQKWAAVVVPSG | 0.052775384 | 0.00801379 | 0.002142276 |
| 4205 | DRTFQKWAAVVVPSGEE | 0.049223955 | 0.000153114 | 0.018212691 |
| 4206 | DRTFQKWAAVVVPSGEEQ | 0.010029132 | 0.000825393 | 0.033430783 |
| 4207 | DRTFQKWAAVVVPSGEEQR | 0.000573412 | 0 | 0.013868523 |
| 4208 | GDRTFQKWAAVVVPSGEEQR | 0 | 0 | 0.014988926 |
| 4209 | RPAGDRTFQKWAAVVVPSGEEQR | 0.000270528 | 0.000415956 | 0.017638726 |
| 4210 | RTFQKWAAVVVP | 0.003811225 | 0 | 0.000221144 |
| 4211 | GDRTFQKWAAVVVP | 0.010772739 | 0.003505306 | 0.000274678 |
| 4212 | GDRTFQKWAAVVVPS | 0.006356061 | 0.017113602 | 0.000243654 |
| 4213 | GDRTFQKWAAVVVPSG | 0.046469986 | 0.054903792 | 0.005671788 |
| 4214 | GDRTFQKWAAVVVPSGE | 0.000412734 | 0.006032867 | 0.001253644 |
| 4215 | GDRTFQKWAAVVVPSGEE | 0.027867051 | 0.004869236 | 0.015499146 |
| 4216 | GDRTFQKWAAVVVPSGEEQ | 0.01069471 | 0.002244147 | 0.029596497 |
| 4217 | PAGDRTFQKWAAVVVPSGEEQ | 0.000438404 | 0 | 0.002827201 |
| 4218 | RPAGDRTFQKWAAVVVPSGEEQ | 0.01337402 | 0.010156938 | 0.065242975 |
| 4219 | PAGDRTFQKWAAVVVPSG | 0.002726226 | 0.000227965 | 0 |
| 4220 | RPAGDRTFQKWAAVVVPSG | 0.033127072 | 0.086538803 | 0.017229046 |
| 4221 | RPAGDRTFQKWAAVVVPSGE | 0 | 0.014559191 | 0.001299808 |
| 4222 | RPAGDRTFQKWAAVVVPSGEE | 0.014132029 | 0.017691388 | 0.030349592 |
| 4223 | RTFQKWAAVVVPSG | 0.00530227 | 0.000655658 | 0 |
| 4224 | RPAGDRTFQKWAAVVVP | 0.007525567 | 0.004919353 | 7.51555E-05 |
| 4225 | RPAGDRTFQKWAAVVVPS | 0.007464013 | 0.012953379 | 0.000366204 |
| 4226 | RPAGDGTFQKW | 0 | 0.001490778 | 0 |
| 4227 | RPAGDGTFQKWAAVVVP | 0.008639953 | 0.00035588 | 0.000176585 |
| 4228 | RPAGDGTFQKWAAVVVPS | 0.011226529 | 0.00088595 | 0 |
| 4229 | RPAGDGTFQKWAAVVVPSG | 0.091467863 | 0.062857209 | 0.019073433 |
| 4230 | RPAGDGTFQKWAAVVVPSGE | 0.000844764 | 0.011750304 | 0.001902618 |
| 4231 | RPAGDGTFQKWAAVVVPSGQ | 0.0004812 | 0 | 0.00257343 |
| 4232 | RPAGDGTFQKWASVVVP | 0 | 0.007294349 | 0 |
| 4233 | RPAGDGTFQKWASVVVPS | 0 | 0.004252161 | 0 |
| 4234 | RPAGDGTFQKWASVVVPSG | 0 | 0.132642004 | 0 |
| 4235 | RPAGDGTFQKWASVVVPSGQ | 0 | 0.038634675 | 0 |
| 4236 | EIPVVIAASEDRLGG | 0.000191962 | 0 | 0 |
| 4237 | IPVVIAASEDRLGG | 0.003758992 | 0 | 0 |
| 4238 | SPPVMVAGGRVF | 0 | 0 | 0.002624781 |
| 4239 | APGLIIATGSVGKN | 0 | 0 | 0.074825175 |
| 4240 | APGLIIATGSVGKNL | 0 | 0.001613177 | 0.108180816 |
| 4241 | APGLIIATGSVGKNLA | 0 | 4.65866E-05 | 0.017404603 |
| 4242 | APGLIIATGSVGKNLAS | 0 | 2.641E-05 | 0.030837728 |
| 4243 | APGLIIATGSVGKNLASK | 0 | 0 | 0.034557849 |
| 4244 | GLIIATGSVGKNL | 0 | 0 | 0.000565265 |
| 4245 | DPWSGGGVTKDIT | 0 | 0.000231482 | 0.002332257 |
| 4246 | DPWSGGGVTKDITD | 0 | 0 | 0.007829916 |
| 4247 | DPWSGGGVTKDITDT | 0 | 0 | 0.006382454 |
| 4248 | LDPWSGGGVTKDITDT | 0 | 0 | 0.008526164 |
| 4249 | LDPWSGGGVTKDITD | 0 | 0 | 0.006823541 |
| 4250 | DPWAGGGIRRN | 6.66923E-05 | 0 | 0.003342119 |
| 4251 | DILVVATGQPEMVK | 0 | 0 | 0.000592354 |
| 4252 | VVIVAGSNGWYNYRH | 0 | 0 | 0.002132188 |
| 4253 | VVIVAGSNGWYNYRHQ | 0.000147249 | 0 | 0.004460489 |
| 4254 | EDGVITASEDRTIR | 0.000135635 | 0.000603096 | 1.83527E-05 |
| 4255 | IPKEDGVITASEDRTIR | 0.000137277 | 0.003290077 | 0.001803955 |
| 4256 | IPKEDGVITASEDRT | 0 | 0.000143799 | 0 |
| 4257 | TPETLLGSTEEKPLPLG | 0 | 0.000726401 | 0 |
| 4258 | ARAKVIAAEGEMNASR | 1.11636E-05 | 2.48102E-05 | 0.001277318 |
| 4259 | ASEAEVEQLLSERAP | 0.002649828 | 6.23741E-05 | 0 |
| 4260 | SEAEVEQLLSERAP | 0.001831678 | 0 | 0 |
| 4261 | VSLLAASEAEVEQL | 0 | 0.008316604 | 0.000959823 |
| 4262 | VSLLAASEAEVEQLL | 6.66238E-05 | 0.00715479 | 0 |
| 4263 | VVTTQGEVEEIEQ | 0 | 0.001307608 | 0 |
| 4264 | VVTTQGEVEEIEQH | 0 | 0.001417459 | 0 |
| 4265 | LQDMINEVDADGN | 0.011082938 | 0 | 0 |
| 4266 | NPTEAELQDMINEVDADGN | 0.00182154 | 0 | 0 |
| 4267 | LRTDQLDGETD | 0 | 0.001101725 | 0 |
| 4268 | LRTDQLDGETDWK | 0 | 0.00055423 | 6.08595E-05 |
| 4269 | EFTEAESNMNDL | 0 | 0.003346131 | 0 |
| 4270 | IDDPNSNLEEAINE | 0 | 0.000880352 | 0 |
| 4271 | KPSGPQPQADLQALLQ | 0.001078473 | 0.000168547 | 0 |
| 4272 | EGEEVDYDVSPSDP | 0.00352069 | 0 | 0 |
| 4273 | EGEEVDYDVSPSDPK | 0.003284979 | 0 | 0 |
| 4274 | ELHFEGEEVDYDVSPS | 0.004969773 | 0.000166145 | 0 |
| 4275 | RELHFEGEEVDYDVSPS | 0.013346101 | 0.00078056 | 0.000605312 |
| 4276 | TRELHFEGEEVDYDVSPS | 0.001923343 | 0.000250683 | 0 |
| 4277 | GDLEYQMSTTARAKR | 0 | 0.000733404 | 0.002040569 |
| 4278 | VPPSQGDLEYQMSTTARAKR | 0 | 0 | 0.00020161 |
| 4279 | VPPSQGDLEYQMSTTARAK | 0 | 3.11764E-05 | 0.002295818 |
| 4280 | AEGVIVGHW | 0 | 0.000142205 | 0 |
| 4281 | NETLIVSKF | 0 | 0.001130869 | 1.72983E-05 |
| 4282 | GDPMILSCLMEHL | 0 | 0.000250813 | 0 |
| 4283 | AKKDEPKSGEEALIIP | 0 | 0.011763763 | 0 |
| 4284 | AKKDEPKSGEEALIIPP | 0 | 0.022368986 | 0.001034755 |
| 4285 | AKKDEPKSGEEALIIPPD | 0 | 0.02872452 | 6.5881E-05 |
| 4286 | AKKDEPKSGEEALIIPPDA | 0 | 0.000806246 | 0 |
| 4287 | DEPKSGEEALIIP | 0 | 0.051754755 | 0 |
| 4288 | KDEPKSGEEALIIP | 0.000174111 | 0.043119839 | 0 |
| 4289 | KKDEPKSGEEALIIP | 0 | 0.043494222 | 1.8191E-05 |
| 4290 | EAKKDEPKSGEEALIIP | 0 | 0.003838055 | 0 |
| 4291 | EAKKDEPKSGEEALIIPP | 0 | 0.005483711 | 4.73094E-05 |
| 4292 | DEPKSGEEALIIPP | 0.000231457 | 0.106283003 | 0 |
| 4293 | DEPKSGEEALIIPPD | 0 | 0.018967634 | 0 |
| 4294 | EAKKDEPKSGEEALIIPPD | 0 | 0.003500869 | 0 |
| 4295 | EPKSGEEALIIPP | 0 | 0.000420403 | 0.000314596 |
| 4296 | KDEPKSGEEALIIPPD | 0 | 0.021106608 | 0.000157977 |
| 4297 | KKDEPKSGEEALIIPPD | 0 | 0.029021144 | 0 |
| 4298 | KDEPKSGEEALIIPP | 0 | 0.065399107 | 9.38539E-05 |
| 4299 | KKDEPKSGEEALIIPP | 0 | 0.070565185 | 0.001098665 |
| 4300 | GPNEAMVVSGFCRSPPV | 0 | 0 | 0.000545778 |
| 4301 | DDNTVIIGSANINDRS | 0 | 0 | 0.000732386 |
| 4302 | ETKKFRFEEPVVLPDL | 0.002136969 | 0 | 0.000592897 |
| 4303 | ETKKFRFEEPVVLPDLD | 0.002329508 | 0 | 0 |
| 4304 | DNMIASGQLDESIR | 0 | 0.00033313 | 0.003401508 |
| 4305 | LDNMIASGQLDESIR | 6.27599E-05 | 0.000313782 | 0.017101031 |
| 4306 | LDNMIASGQLDESIRE | 0 | 0 | 0.000371621 |
| 4307 | LDNMIASGQLDESIREN | 0 | 0 | 0.004658185 |
| 4308 | GNQAASQLEEAGRA | 0 | 0.000372157 | 0 |
| 4309 | KTASNVEEAFIN | 0 | 0.001215026 | 0 |
| 4310 | SAKTASNVEEAFIN | 0 | 0.003143071 | 0 |
| 4311 | SAKTASNVEEAFINT | 0 | 0.001425358 | 0.000785981 |
| 4312 | SAKTASNVEEAFINTA | 4.27654E-05 | 0.000923364 | 0 |
| 4313 | TSAKTASNVEEAFINTA | 0 | 0.000529333 | 0 |
| 4314 | TSAKTASNVEEAFIN | 0 | 0.000227279 | 0 |
| 4315 | ALDLGFGDAVHVYDGPG | 0 | 0 | 0.005689059 |
| 4316 | DLGFGDAVHVYDGPG | 0.000330164 | 0 | 0.003115097 |
| 4317 | TALDLGFGDAVHVYDGPG | 0 | 0 | 0.001234309 |
| 4318 | DLGFGDAVHVYDGPGPPE | 0 | 0 | 0.000827487 |
| 4319 | DEFSEAIKAF | 0 | 0.002485224 | 0 |
| 4320 | NEFPEPIKL | 0 | 0.012127522 | 0.000182885 |
| 4321 | TEVELYNEFPEPIK | 0.000224126 | 0 | 0 |
| 4322 | GPEEFAEGLVIGVR | 0 | 0 | 0.000736975 |
| 4323 | EPVVLPAEARRGP | 0 | 0 | 0.005294033 |
| 4324 | EPVVLPAEARRGPA | 0 | 0 | 0.00052661 |
| 4325 | PPSEYTWFKDGIVMPTNPK | 0 | 0 | 0.003708021 |
| 4326 | SPPSEYTWFKDGIVMPTNPK | 0 | 0 | 0.15441331 |
| 4327 | SPPSEYTWFKDGIVMPTNPKS | 0 | 0 | 0.045878989 |
| 4328 | SPPSEYTWFKDGIVMPTNPKST | 0 | 0 | 0.018689987 |
| 4329 | YTWFKDGIVMPTNPK | 0 | 0 | 0.00438633 |
| 4330 | SPPSEYTWFKDGIVMPT | 0 | 0.000214282 | 0.00403947 |
| 4331 | SPPSEYTWFKDGIVMPTN | 0 | 0.001523102 | 0.0670058 |
| 4332 | SPPSEYTWFKDGIVMPTNP | 9.19673E-05 | 0 | 0.026294987 |
| 4333 | FDGGVVMGSDSR | 0 | 0 | 0.003209506 |
| 4334 | AEEEEVAEVEEEEADDD | 0.00016204 | 0 | 0 |
| 4335 | EEEEVAEVEEEEA | 0.001370557 | 0 | 0 |
| 4336 | EEEEVAEVEEEEADD | 0.000530845 | 0 | 0 |
| 4337 | EEEEVAEVEEEEADDD | 0.001418071 | 0 | 0 |
| 4338 | EEVAEVEEEEA | 0.00012634 | 9.31476E-05 | 0 |
| 4339 | EEVAEVEEEEAD | 0.000686538 | 0.000168059 | 0 |
| 4340 | EEVAEVEEEEADD | 0.000111969 | 0 | 0 |
| 4341 | EEVAEVEEEEADDD | 0.001359916 | 7.6331E-05 | 0 |
| 4342 | EVAEVEEEEAD | 0.000535731 | 0.000316516 | 0 |
| 4343 | EVAEVEEEEADD | 0.00079159 | 0 | 0 |
| 4344 | EVAEVEEEEADDD | 0.001614145 | 0.001988597 | 0 |
| 4345 | EVAEVEEEEADDDE | 0.000399123 | 0.002056188 | 0 |
| 4346 | VAEVEEEEADDD | 0.000617685 | 0.001137388 | 0 |
| 4347 | VAEVEEEEADDDE | 0 | 0.000903255 | 0 |
| 4348 | DKVVEVAEEEEVAE | 0.001115488 | 0 | 0 |
| 4349 | EDKVVEVAEEEEVAE | 0.003212059 | 0.00039156 | 0 |
| 4350 | GSEDKVVEVAEEEEVAE | 0.006738257 | 5.9102E-05 | 0 |
| 4351 | GSEDKVVEVAEEEEVAEVE | 0.001884582 | 0 | 0.000164259 |
| 4352 | KVVEVAEEEEVAE | 0.004900996 | 0 | 0 |
| 4353 | SEDKVVEVAEEEEVAE | 0.002888288 | 0 | 0 |
| 4354 | VVEVAEEEEVAE | 0.001573628 | 0 | 0 |
| 4355 | KVVEVAEEEEVAEV | 0.000829511 | 0.000524061 | 0 |
| 4356 | KVVEVAEEEEVAEVE | 0.008938566 | 0.000599842 | 4.13194E-05 |
| 4357 | KVVEVAEEEEVAEVEE | 0.002763097 | 0.000115598 | 0 |
| 4358 | KVVEVAEEEEVAEVEEE | 0.001375778 | 8.20444E-05 | 0 |
| 4359 | VVEVAEEEEVAEVE | 0.001913753 | 0 | 0.000124323 |
| 4360 | VAEEEEVAEVEE | 0.000581157 | 3.01889E-05 | 0 |
| 4361 | GSEDKVVEVAEEEEVA | 0.004240899 | 0 | 0 |
| 4362 | SEDKVVEVAEEEEVA | 0.002080354 | 0 | 0 |
| 4363 | VPEDDNISNDSNDFT | 0.00253405 | 0 | 0 |
| 4364 | APPEVPESGDLDDDAEAIL | 0 | 0.002549274 | 0 |
| 4365 | KVYELSNVQEDSQP | 0.045754326 | 0.007383381 | 0 |
| 4366 | KVYELSNVQEDSQPM | 0.009860889 | 0.000293676 | 0.000142216 |
| 4367 | LPGNNRKVYELSNVQEDSQPM | 0.002072624 | 0.000290186 | 0 |
| 4368 | LSNVQEDSQP | 0.001258608 | 0 | 1.87131E-05 |
| 4369 | RKVYELSNVQEDSQPM | 0.000823689 | 0.00036003 | 0 |
| 4370 | VYELSNVQEDSQP | 0.022548761 | 0.001166077 | 0 |
| 4371 | VYELSNVQEDSQPM | 0.002648024 | 0 | 0.000709412 |
| 4372 | LPGNNRKVYELSNVQEDSQP | 0.008812508 | 0.002870235 | 0.000369876 |
| 4373 | RKVYELSNVQEDSQP | 0.010917499 | 0.004042104 | 0.000377349 |
| 4374 | LPPELRRQRSGEVENNSDN | 0 | 0 | 0.000890337 |
| 4375 | GSGFLTEMEQDYQL | 0 | 0.006652888 | 0 |
| 4376 | GSGFLTEMEQDYQLV | 0 | 0.002502828 | 0 |
| 4377 | SGSGFLTEMEQDYQLV | 0.000109239 | 0.004064058 | 0 |
| 4378 | SGSGFLTEMEQDYQLVD | 0 | 0.00163555 | 0 |
| 4379 | SGSGFLTEMEQDYQL | 0 | 0.001541811 | 0 |
| 4380 | VTEIDQDKY | 0.001511057 | 0 | 0 |
| 4381 | TRFFETCDLDNDKYI | 0 | 0.000920262 | 0 |
| 4382 | TTRFFETCDLDNDKYIA | 0 | 0.005229139 | 0 |
| 4383 | ASFDVNDQDPDPQPR | 0 | 0.002723875 | 0 |
| 4384 | ASFDVNDQDPDPQPRY | 0 | 0.001343005 | 0 |
| 4385 | DPGASFDVNDQDPDPQPRY | 0 | 0.001551952 | 0 |
| 4386 | DPGASFDVNDQDPDPQPRYT | 0 | 0.005388083 | 0 |
| 4387 | SFDVNDQDPDPQPR | 0 | 0.000728175 | 0 |
| 4388 | GASFDVNDQDPDPQPR | 0 | 0.001311536 | 0 |
| 4389 | DPGASFDVNDQDPDPQP | 0 | 0.004063115 | 0 |
| 4390 | DPGASFDVNDQDPDPQPR | 0 | 0.01213977 | 0 |
| 4391 | GKTIQVDNTDAEGRLI | 0 | 0.00023316 | 0 |
| 4392 | IQVDNTDAEGRL | 0 | 0.002905214 | 0 |
| 4393 | IQVDNTDAEGRLI | 4.08851E-05 | 0.013278334 | 0.000678081 |
| 4394 | TIQVDNTDAEGRL | 0 | 0.00049111 | 0 |
| 4395 | KGAQAGTQVEEAEAEA | 0 | 0.000294094 | 8.24592E-06 |
| 4396 | SDQVPDTESETRIL | 0 | 0.000691469 | 0 |
| 4397 | ESKTHFRAVDPDGDGHV | 0.001112159 | 1.37301E-05 | 0 |
| 4398 | AEQVREENLPDE | 0.001701428 | 0.0072466 | 0 |
| 4399 | AEQVREENLPDEN | 0 | 0.000352952 | 0 |
| 4400 | DEFEEAEQVREENLPDEN | 0.004552583 | 0.036647492 | 0 |
| 4401 | EAEQVREENLPDEN | 0.000435788 | 0.000674861 | 0 |
| 4402 | EDEFEEAEQVREENLPDEN | 0.00023947 | 0.009410469 | 0 |
| 4403 | EEAEQVREENLPDEN | 0.00062389 | 0.003243215 | 0 |
| 4404 | EFEEAEQVREENLPDEN | 0.012712925 | 0.036303477 | 0 |
| 4405 | FEEAEQVREENLPDEN | 0.001526312 | 0.010250323 | 0 |
| 4406 | DEFEEAEQVREENLPDE | 0.008562384 | 0.045492577 | 0 |
| 4407 | EAEQVREENLPDE | 0.001792414 | 0.002827348 | 0 |
| 4408 | EEAEQVREENLPDE | 0.001281406 | 0.006552483 | 0 |
| 4409 | EFEEAEQVREENLPDE | 0.048537164 | 0.063857842 | 0 |
| 4410 | FEEAEQVREENLPDE | 0.032537544 | 0.116434312 | 7.15913E-05 |
| 4411 | EFEEAEQVREENLPD | 0.001160367 | 0.001006241 | 0 |
| 4412 | FEEAEQVREENLPD | 0.009365128 | 0.004865704 | 0 |
| 4413 | EEPNNIHDEDCATMNKGG | 0 | 0.0017886 | 0 |
| 4414 | EPEEPNNIHDEDCATMNKGG | 0.001242244 | 0.049422523 | 0 |
| 4415 | PEEPNNIHDEDCATMNKGG | 0 | 0.002275521 | 0 |
| 4416 | EPEEPNNIHDEDCATMNKG | 0.000273128 | 0.003189757 | 0 |
| 4417 | DENPQQLKL | 0 | 0.003172764 | 9.08831E-05 |
| 4418 | DDDESDDTLEENRG | 0 | 0 | 0 |
| 4419 | DDESDDTLEENRG | 0 | 0.000938375 | 0 |
| 4420 | LTPDTLDEEKAR | 0 | 0.000715642 | 0 |
| 4421 | TPDTLDEEKAR | 0 | 0.000559532 | 0 |
| 4422 | DEDGDEVEEEAEEPY | 0.000960555 | 0 | 0 |
| 4423 | DEDGDEVEEEAEEPYE | 0.002225236 | 0.000280463 | 0 |
| 4424 | DGDEVEEEAEEPY | 0 | 0 | 0 |
| 4425 | EDGDEVEEEAEEPY | 0.00138472 | 0.000289842 | 0 |
| 4426 | VEEEAEEPY | 0 | 0 | 0 |
| 4427 | DGDEVEEEAEEPYE | 0.004445486 | 0 | 0 |
| 4428 | EDGDEVEEEAEEPYE | 0.002735321 | 0.000100957 | 0 |
| 4429 | GDEVEEEAEEPYE | 0.009305797 | 0.000543791 | 0 |
| 4430 | VEEEAEEPYE | 0.001963071 | 4.66706E-05 | 0 |
| 4431 | GDEVEEEAEEPYEE | 0.001054682 | 0 | 0 |
| 4432 | VEEEAEEPYEE | 0.000448034 | 0 | 0 |
| 4433 | EEEGLEDIDEEGDE | 0.000203459 | 7.01734E-05 | 0 |
| 4434 | SDDEEESEEAKRL | 0 | 0.000114302 | 0 |
| 4435 | SDDEEESEEAKRLR | 0 | 0.000832529 | 0 |
| 4436 | ALGFYPAEITLTWQR | 0 | 0 | 0.001818726 |
| 4437 | ALSFYPAEITLTWQR | 0 | 0 | 0.005111591 |
| 4438 | ALSFYPAEITLTWQRD | 0 | 0 | 0.006966121 |
| 4439 | ALSFYPAEITLTWQRDG | 0 | 0.000135558 | 0.002012476 |
| 4440 | SFYPAEITLTWQR | 0 | 0 | 0.003008892 |
| 4441 | WALGFYPAEITLTWQRDG | 0 | 0 | 0.003407897 |
| 4442 | WALGFYPAEITLTWQRDGEDQ | 0 | 0 | 0.0004796 |
| 4443 | WALSFYPAEITLTWQRDG | 0 | 0 | 0.008972911 |
| 4444 | GFYPAEITLTWQRD | 0 | 0 | 0.002069143 |
| 4445 | SFYPAEITLTWQRD | 0 | 0.000424834 | 0.009179566 |
| 4446 | SFYPAEITLTWQRDG | 0 | 0.003033879 | 0.005623112 |
| 4447 | WALSFYPAEITLTWQR | 0 | 8.21306E-05 | 0.007045304 |
| 4448 | WALSFYPAEITLTWQRD | 0 | 0 | 0.0184588 |
| 4449 | DGEDQTQDTELVE | 0 | 0.005558026 | 0 |
| 4450 | DGEDQTQDTELVETRP | 3.68453E-05 | 0.005928257 | 0 |
| 4451 | DGEDQTQDTELVETRPA | 0 | 0.031259078 | 0 |
| 4452 | DGEDQTQDTELVETRPAG | 0 | 0.002729884 | 0 |
| 4453 | DGEDQTQDTELVETRPAGDG | 0 | 0.001293089 | 0 |
| 4454 | DGEDQTQDTELVETRPAGDR | 0 | 0.001938292 | 0 |
| 4455 | DQTQDTELVETRPA | 0 | 0.010640504 | 0 |
| 4456 | EDQTQDTELVETRPA | 0 | 0.064205071 | 0.002054168 |
| 4457 | GEDQTQDTELVETRPA | 0 | 0.010801379 | 0 |
| 4458 | EDQTQDTELVETRP | 0.001541184 | 0.00385325 | 0 |
| 4459 | EDQTQDTELVETRPAGDG | 0 | 5.10101E-05 | 0 |
| 4460 | RDGEDQTQDTELVETRP | 0 | 0.004040531 | 0 |
| 4461 | RDGEDQTQDTELVETRPA | 0 | 0.005464807 | 0 |
| 4462 | QRDGEDQTQDTELVE | 0 | 0.008973663 | 0 |
| 4463 | QRDGEDQTQDTELVETRPA | 0 | 0.001843594 | 0 |
| 4464 | RDGEDQTQDTELVE | 0.000152891 | 0.017709595 | 0 |
| 4465 | RDGEEQTQDTELVE | 0.003064899 | 0.002568517 | 2.75549E-05 |
| 4466 | WQRDGEDQTQDTELVE | 0 | 0.000729681 | 0 |
| 4467 | EITLTWQRDGEDQTQDT | 0.003130593 | 0 | 0 |
| 4468 | EITLTWQRDGEDQTQDTE | 0.003480388 | 0.000145812 | 0 |
| 4469 | ITLTWQRDGEDQT | 0.005214981 | 0.000259644 | 0.000291156 |
| 4470 | ITLTWQRDGEDQTQ | 0.014067286 | 0 | 0 |
| 4471 | ITLTWQRDGEDQTQD | 0.053485613 | 0 | 0.000364122 |
| 4472 | ITLTWQRDGEDQTQDT | 0.124946088 | 0 | 0 |
| 4473 | LTWQRDGEDQT | 0.002528936 | 0 | 0 |
| 4474 | YPAEITLTWQRDGEDQTQDTE | 0.004231687 | 0 | 0 |
| 4475 | YPAEITLTWQRDGEDQTQDT | 0.003305979 | 0 | 0 |
| 4476 | ITLTWQRDGEDQTQDTE | 0.043448177 | 0 | 0 |
| 4477 | ITLTWQRDGEDQTQDTEL | 0.004442963 | 0 | 0 |
| 4478 | LTWQRDGEDQTQ | 0.008353411 | 0 | 0 |
| 4479 | LTWQRDGEDQTQD | 0.020732268 | 0 | 0 |
| 4480 | LTWQRDGEDQTQDT | 0.06199181 | 0 | 0 |
| 4481 | LTWQRDGEDQTQDTE | 0.04067609 | 3.51334E-05 | 4.9292E-05 |
| 4482 | LTWQRDGEDQTQDTEL | 0.011434797 | 0 | 0 |
| 4483 | LTWQRDGEDQTQDTELVE | 0.007771779 | 0.000279604 | 0 |
| 4484 | TWQRDGEDQTQD | 0.000371215 | 0 | 0 |
| 4485 | TWQRDGEDQTQDT | 0.000939823 | 0 | 0.000155496 |
| 4486 | TWQRDGEDQTQDTE | 0.001045679 | 1.21596E-05 | 0 |
| 4487 | WQRDGEDQTQDVELVE | 0 | 0.002125785 | 0 |
| 4488 | VWGFYPAEVTITWRKN | 0 | 0 | 9.17998E-05 |
| 4489 | YVWGFYPAEVTITWRKN | 0.000255541 | 0 | 0.002056562 |
| 4490 | QDTELVETRPAGDRTFQ | 4.56778E-05 | 0.000437647 | 0 |
| 4491 | SPGDQVMEGKSATL | 0 | 0 | 0.001602933 |
| 4492 | SPGDQVMEGKSATLT | 0 | 0.000262865 | 0.004190099 |
| 4493 | APPVRHLIATQLLSNLE | 0 | 0.004542569 | 0 |
| 4494 | APPVRHLIATQLLSNLED | 0 | 0.002981222 | 0 |
| 4495 | APPVRHLIATQLLSNLEDI | 0 | 0.00093954 | 0 |
| 4496 | APPVRHLIATQLLSNLEDIM | 0 | 0.001375025 | 0 |
| 4497 | ATQLLSNLEDIM | 0 | 0.008702473 | 0 |
| 4498 | ATQLLSNLEDIMR | 0.000496164 | 0.017486106 | 0.000187332 |
| 4499 | ATQLLSNLEDIMRIL | 0 | 0.005446635 | 0.000167631 |
| 4500 | ATQLLSNLEDIMRILA | 0 | 0.000793523 | 0 |
| 4501 | IATQLLSNLEDIMRILA | 0 | 0.001098878 | 0 |
| 4502 | QLLSNLEDIMR | 0.000220107 | 0.000624357 | 0 |
| 4503 | TQLLSNLEDIMR | 0 | 0.001034648 | 0 |
| 4504 | LLSNLEDIMR | 0 | 0.001292152 | 0 |
| 4505 | LLSNLEDIMRIL | 0 | 0.001264196 | 8.58664E-06 |
| 4506 | QLLSNLEDIMRIL | 0 | 0 | 0 |
| 4507 | TQLLSNLEDIMRIL | 0 | 0.000542039 | 2.58203E-05 |
| 4508 | IATQLLSNLEDIM | 0 | 0.002699863 | 4.62225E-05 |
| 4509 | IATQLLSNLEDIMR | 0.001165531 | 0.007164754 | 0.000123935 |
| 4510 | IATQLLSNLEDIMRIL | 0 | 0.000676033 | 0 |
| 4511 | ILLAELEQLK | 0 | 0.000160234 | 0.000512082 |
| 4512 | IGATLTDVDDLIFK | 0.001241118 | 0.000512654 | 0 |
| 4513 | PPGLEYLSQIDQIL | 0 | 0.002508683 | 0 |
| 4514 | GEIQEIVKTGLW | 0 | 0.000892313 | 0 |
| 4515 | GLQEYKSLQSELDEIN | 0.002647578 | 0.001611069 | 0 |
| 4516 | VPVESDLQEIIK | 0 | 0.002511158 | 0 |
| 4517 | VPVESDLQEIIKE | 0 | 0.000216042 | 0 |
| 4518 | DLKDMTSEQLDDIL | 3.63799E-05 | 0.055761493 | 0 |
| 4519 | DLKDMTSEQLDDILK | 9.13361E-05 | 0.156867073 | 0.000418623 |
| 4520 | DLKDMTSEQLDDILKY | 0 | 0.000434461 | 0 |
| 4521 | DLKDMTSEQLDDILKYH | 0 | 0.004988229 | 0.000192245 |
| 4522 | GSDLKDMTSEQLDDILKYH | 0 | 0.001010434 | 0.002530614 |
| 4523 | SDLKDMTSEQLDDILKYH | 0 | 0.002863859 | 0 |
| 4524 | QELDDILKF | 0 | 0.001977895 | 3.07033E-05 |
| 4525 | DMTSEQLDDILK | 0 | 0.015386132 | 0.000162803 |
| 4526 | GSDLKDMTSEQLDDILK | 0 | 0.007222185 | 0 |
| 4527 | LKDMTSEQLDDILK | 0 | 0.010555917 | 7.11968E-05 |
| 4528 | SDLKDMTSEQLDDILK | 0.000798963 | 0.036569756 | 0.00070561 |
| 4529 | GSDLKDMTSEQLDDIL | 0 | 0.004320042 | 0.000114185 |
| 4530 | SDLKDMTSEQLDDIL | 0 | 0.0185457 | 0 |
| 4531 | EEIQDEVDELL | 0.000725497 | 0.000640105 | 0 |
| 4532 | IEDLQDQLEDMM | 0 | 0.000742383 | 0 |
| 4533 | IEDLQDQLEDMME | 0 | 0.001113275 | 0 |
| 4534 | IEDLQDQLEDMMEDAN | 0 | 0.000447376 | 0 |
| 4535 | VAEEIQDEVDELLQ | 0.001333779 | 0.000903215 | 0 |
| 4536 | VAEEIQDEVDELLQKE | 0.000700338 | 0.000595483 | 0 |
| 4537 | VAEEIQDEVDELLQKEQ | 0 | 0.001914066 | 0.000143835 |
| 4538 | VPAVAEEIQDEVDELLQKEQ | 0 | 0.00086825 | 0 |
| 4539 | GHDDDVIDIEDDLDDVIE | 0 | 0.000162277 | 0 |
| 4540 | VIDIEDDLDDVIE | 0.000156691 | 0.002502736 | 0 |
| 4541 | VIDIEDDLDDVIEE | 0 | 0.000312581 | 0 |
| 4542 | EELNDLIQRF | 0 | 0.002708337 | 0 |
| 4543 | LEARIAQLEEELEEEQ | 0.000232071 | 0.000167362 | 0 |
| 4544 | VEEMKTQLEELEDELQ | 0 | 0 | 0 |
| 4545 | IEKQYDADLEQIL | 0.00602521 | 0 | 0 |
| 4546 | IEKQYDADLEQILIQ | 0.001141127 | 0 | 0 |
| 4547 | RDFNSELLRL | 0.000249861 | 0.004263073 | 0 |
| 4548 | DDMREMEEERL | 0.006931608 | 0.002211819 | 0 |
| 4549 | DDMREMEEERLR | 0.014572372 | 0.012550523 | 0 |
| 4550 | DDMREMEEERLRMR | 0.002059857 | 0.000149026 | 0 |
| 4551 | DMREMEEERL | 0.000135495 | 0.001326921 | 0 |
| 4552 | DPKNEEDDMREMEEERLRMR | 0.000199794 | 0.002056758 | 0 |
| 4553 | EDDMREMEEERLRMR | 0.000868389 | 0.001032102 | 0 |
| 4554 | EEDDMREMEEERLRMR | 0.018673985 | 0.032017713 | 0 |
| 4555 | EEDDMREMEEERLRM | 0.009923008 | 0.004916328 | 0 |
| 4556 | EEDDMREMEEERLRMRE | 0.004999074 | 0.01599657 | 0 |
| 4557 | EEDDMREMEEERLRMREH | 0.000349909 | 0.003067808 | 0 |
| 4558 | DMREMEEERLR | 0.00384152 | 0.003968906 | 0 |
| 4559 | DPKNEEDDMREMEEERLR | 0.017898787 | 0.054387319 | 0 |
| 4560 | EDDMREMEEERLR | 0.004567112 | 0.015085207 | 0 |
| 4561 | EEDDMREMEEERLR | 0.087667023 | 0.266850189 | 0.000333922 |
| 4562 | NEEDDMREMEEERLR | 0.011491665 | 0.034320031 | 0 |
| 4563 | DPKNEEDDMREMEEERL | 0.005107056 | 0.010385291 | 0 |
| 4564 | EDDMREMEEERL | 0.001597688 | 0.003574945 | 0 |
| 4565 | EEDDMREMEEERL | 0.044755841 | 0.065235645 | 0 |
| 4566 | NEEDDMREMEEERL | 0.00339646 | 0.005160754 | 0 |
| 4567 | DIRDIQDENQ | 0.000935178 | 0.001807704 | 0.000268656 |
| 4568 | DIRDIQDENQL | 0 | 0.004100919 | 0 |
| 4569 | YRDIRDIQDENQL | 0.000116205 | 0.000441529 | 0 |
| 4570 | DMVEMEEERLR | 0 | 0.000230929 | 0.000100994 |
| 4571 | EEDDMVEMEEERLR | 0.007321871 | 0.008340663 | 0.000314589 |
| 4572 | KLIEVDDERKL | 0 | 0 | 0.000923446 |
| 4573 | DPNFVKDLEEDLY | 0.00041165 | 0.000951301 | 0 |
| 4574 | DPNFVKDLEEDLYE | 0.002260459 | 0.003454423 | 0 |
| 4575 | DPNFVKDLEEDLYELF | 0 | 0.00030342 | 0 |
| 4576 | SDPNFVKDLEEDLYELF | 3.25039E-05 | 0.000622666 | 0 |
| 4577 | SDPNFVKDLEEDLYE | 0.000238603 | 0.002569194 | 0 |
| 4578 | AGEYGAEALER | 0.000315614 | 0 | 0.000506648 |
| 4579 | AHAGEYGAEALER | 0.000231695 | 0.000109619 | 0.000434371 |
| 4580 | AHAGEYGAEALERMF | 0.000602553 | 0 | 0.000410832 |
| 4581 | GEYGAEALER | 0.000232417 | 0 | 0.000645184 |
| 4582 | VGAHAGEYGAE | 0.000311036 | 0.000681606 | 0.001074596 |
| 4583 | VGAHAGEYGAEA | 0.000371453 | 0.000116578 | 0.000739262 |
| 4584 | VGAHAGEYGAEAL | 0.000138322 | 0 | 0.000374861 |
| 4585 | VGAHAGEYGAEALE | 0.00058908 | 0 | 0.00075334 |
| 4586 | VGAHAGEYGAEALER | 0.001726177 | 0.00049909 | 0.002156836 |
| 4587 | FIIKSELPLVIG | 0.00231395 | 0.011400461 | 0 |
| 4588 | FIIKSELPLVIGT | 0 | 0.000601899 | 0 |
| 4589 | FIIKSELPLVIGTF | 0 | 0.000503955 | 0 |
| 4590 | IIKSELPLVIG | 0.001716589 | 0.065457655 | 0 |
| 4591 | LFIIKSELPLVIGTF | 0 | 0.000424115 | 0 |
| 4592 | IIKSELPLVIGT | 0 | 0.004221822 | 0 |
| 4593 | IIKSELPLVIGTF | 0 | 0.001075534 | 0 |
| 4594 | LFIIKSELPLVIG | 0.000127058 | 0.002899681 | 0 |
| 4595 | RVHPQVTVY | 0 | 0 | 0.004435469 |
| 4596 | HPQVTVYPAKTQPL | 1.81708E-05 | 0 | 0.001912425 |
| 4597 | HPQVTVYPAKTQPLQ | 0.000161676 | 0 | 0.0190207 |
| 4598 | HPQVTVYPAKTQPLQH | 0.000114275 | 0 | 0.001509498 |
| 4599 | HPQVTVYPAKTQPLQHH | 0 | 0 | 0.002347955 |
| 4600 | HPQVTVYPAKTQPLQHHN | 0 | 0 | 0.004550672 |
| 4601 | FLEVLTEGLERVL | 0 | 0.000407684 | 6.64636E-06 |
| 4602 | RRTETVQKL | 0 | 0.001900802 | 0 |
| 4603 | KKDQDKTDTLEHELR | 0 | 0.000374326 | 0.000100515 |
| 4604 | IPRLRTDLFP | 0 | 0.006612701 | 0 |
| 4605 | IPRLRTDLFPKTR | 0 | 0.007572224 | 0 |
| 4606 | IPRLRTDLFPKTRI | 0 | 0.007738324 | 0 |
| 4607 | KIPRLRTDLFPKTRI | 0 | 0.042383172 | 0 |
| 4608 | KIPRLRTDLFPKTRIQ | 0 | 0.010195286 | 0 |
| 4609 | KIPRLRTDLFPKTRIQDLN | 0 | 0.000529908 | 0 |
| 4610 | KIPRLRTDLFP | 0 | 0.01439804 | 0.003765194 |
| 4611 | KIPRLRTDLFPKTR | 0 | 0.002684544 | 0 |
| 4612 | NKIPRLRTDLFPKTRI | 0 | 0.002468304 | 0 |
| 4613 | NKIPRLRTDLFP | 0 | 0.000221191 | 0 |
| 4614 | NPIAKIHSDLAEER | 0 | 5.92554E-05 | 0 |
| 4615 | NPIAKIHSDLAEERG | 0.000871595 | 0.001043012 | 0 |
| 4616 | NPIAKIHSDLAEERGL | 0 | 0.00035033 | 0 |
| 4617 | YLKVKGNVF | 0.006731439 | 0 | 0 |
| 4618 | IHPEIDNPEYSPD | 0.002588155 | 0 | 0 |
| 4619 | IHPEIDNPEYSPDP | 0.003330631 | 0 | 7.76834E-05 |
| 4620 | IHPEIDNPEYSPDPS | 0.01106699 | 0 | 0.000165451 |
| 4621 | WIHPEIDNPEYSPDPS | 0.008915944 | 0.000201231 | 0 |
| 4622 | IRKEVKGDLENAF | 0.000122306 | 0.000324675 | 0 |
| 4623 | IRKEVKGDLENAFL | 0.000517785 | 0.001526276 | 4.36062E-05 |
| 4624 | SIRKEVKGDLENAFL | 0.000103786 | 0.000150646 | 0 |
| 4625 | TDEFQLHTNVNDGTEFGGSIYQK | 0.000652486 | 0 | 0 |
| 4626 | ADLAGNSEVILPVPAFN | 0 | 0.002531532 | 0 |
| 4627 | HIADLAGNSEVILPVPA | 0.001199693 | 0 | 0 |
| 4628 | SEVILPVPAFNVIN | 0 | 0.00036467 | 0 |
| 4629 | LPVPAFNVINGGSHA | 0 | 0 | 0.000121431 |
| 4630 | LPVPAFNVINGGSHAG | 0 | 0 | 0.004528851 |
| 4631 | LPVPAFNVINGGSHAGN | 0 | 0 | 0.002157038 |
| 4632 | LPVPAFNVINGGSHAGNK | 0 | 0 | 0.007253561 |
| 4633 | LPVPAFNVINGGSHAGNKL | 0 | 0 | 0.005973903 |
| 4634 | LPVPAFNVINGGSHAGNKLA | 0 | 0 | 0.003873519 |
| 4635 | PVPAFNVINGGSHAGN | 9.29469E-05 | 0 | 0.00063715 |
| 4636 | PVPAFNVINGGSHAGNK | 0 | 0 | 0.001980387 |
| 4637 | PVPAFNVINGGSHAGNKL | 0.000104263 | 0.000385204 | 0.00116235 |
| 4638 | DVSNVATDTERLE | 0 | 0.000886954 | 0 |
| 4639 | GQLVFLATEGDHLQ | 0.003217351 | 0.001160621 | 0 |
| 4640 | FLLGKYISIGSEAEKNK | 0 | 0.003391344 | 0 |
| 4641 | GKYISIGSEAEKNK | 0 | 0.000286328 | 0.00146258 |
| 4642 | KYISIGSEAEK | 0 | 0.00015088 | 0 |
| 4643 | KYISIGSEAEKN | 0 | 0 | 0 |
| 4644 | KYISIGSEAEKNK | 0 | 0.001383367 | 0.002579431 |
| 4645 | LGKYISIGSEAEKNK | 5.10607E-05 | 0.003680767 | 0 |
| 4646 | LLGKYISIGSEAEKNK | 0.000163186 | 0.001778032 | 5.46198E-05 |
| 4647 | LGKYISIGSEAEK | 0 | 0 | 0 |
| 4648 | LGKYISIGSEAEKN | 0.000216541 | 0.002067902 | 0.000372991 |
| 4649 | IAQVATISANGDKE | 0.000158567 | 0.00274873 | 0 |
| 4650 | IAQVATISANGDKEI | 0 | 0.0011329 | 0 |
| 4651 | IAQVATISANGDKEIG | 0 | 0.009256047 | 0 |
| 4652 | TPEEIAQVATISANGDKEIG | 0 | 0.002471363 | 0 |
| 4653 | TPEEIAQVATISAN | 0 | 0.00104631 | 0.000205558 |
| 4654 | RYLTVAAVF | 0 | 0 | 0.000697393 |
| 4655 | DEEMDVGTDEEEETAK | 0 | 0.000113336 | 0 |
| 4656 | DEEMDVGTDEEEETAKE | 0 | 0.000291835 | 0 |
| 4657 | DVGTDEEEETAK | 0 | 0.000501952 | 0 |
| 4658 | EEMDVGTDEEEETAK | 0 | 0.004786249 | 0 |
| 4659 | EMDVGTDEEEETAK | 0 | 0.011505626 | 1.83221E-05 |
| 4660 | EMDVGTDEEEETA | 0 | 0.000458184 | 0 |
| 4661 | DVGTDEEEETAKE | 0 | 0.000370522 | 0 |
| 4662 | EEMDVGTDEEEETAKE | 0 | 0.004187187 | 0.00063243 |
| 4663 | EMDVGTDEEEETAKE | 0 | 0.00256289 | 0 |
| 4664 | EKPKIEDVGSDEEDDSG | 0.000246388 | 0.002749626 | 0 |
| 4665 | KPKIEDVGSDEEDDSG | 4.83869E-05 | 0.00061436 | 6.63035E-05 |
| 4666 | KPKIEDVGSDEEDDSGKD | 0 | 0.000233142 | 0 |
| 4667 | VGTDEEEETAKESTAEKDEL | 0.001117274 | 0.00105907 | 0.001209951 |
| 4668 | DKPEIEDVGSDEEEE | 0.000516861 | 0.000865601 | 0 |
| 4669 | DKPEIEDVGSDEEEEK | 0.000509767 | 0.004181547 | 0 |
| 4670 | DKPEIEDVGSDEEEEKK | 0.000249461 | 0.003569342 | 0 |
| 4671 | EDKPEIEDVGSDEEEEKK | 0.000162881 | 0.000758195 | 0 |
| 4672 | KPEIEDVGSDEEEE | 0 | 0.000320569 | 0 |
| 4673 | VPREAGSQKDENL | 3.69009E-05 | 0.046982689 | 0 |
| 4674 | VPREAGSQKDENLA | 0 | 0.07429222 | 0 |
| 4675 | VPREAGSQKDENLAL | 0 | 0.004832737 | 0.000402385 |
| 4676 | VPREAGSQKDENLALY | 0 | 0.005520215 | 6.46584E-05 |
| 4677 | ESPSRGLIQEMSG | 0 | 0 | 0.000877861 |
| 4678 | ESPSRGLIQEMSGDA | 0 | 0.000112449 | 0.002159248 |
| 4679 | GLIQEMSGDASVCPDK | 0 | 0.000392326 | 0.021530023 |
| 4680 | IQEMSGDASVCPDK | 0 | 0 | 0.002108226 |
| 4681 | LIQEMSGDASVCPDK | 9.3033E-05 | 0.000484369 | 0.127154191 |
| 4682 | SPSRGLIQEMSGDASVCPDK | 0 | 0 | 0.107326622 |
| 4683 | SPSRGLIQEMSGDASVCP | 0 | 8.46648E-05 | 0.003769864 |
| 4684 | SPSRGLIQEMSGDASVCPD | 0 | 0 | 0.02182617 |
| 4685 | GKVNVDEVGGEA | 0.002681799 | 0.001025198 | 0.00753108 |
| 4686 | GKVNVDEVGGEAL | 0 | 0 | 0.00047349 |
| 4687 | KVNVDEVGGEA | 0.001542307 | 0.001697719 | 0.006261017 |
| 4688 | LWGKVNVDEVGGEAL | 0.001626654 | 0 | 0.001200997 |
| 4689 | LWGKVNVDEVGGEALGRL | 0.002893179 | 0 | 0.001784962 |
| 4690 | VNVDEVGGEA | 0.004002773 | 0.002262719 | 0.006170513 |
| 4691 | WGKVNVDEVGGEA | 0.019042721 | 0.00153167 | 0.032879283 |
| 4692 | LWGKVNVDEVGGE | 0.000920065 | 0.000138973 | 0.000899721 |
| 4693 | LWGKVNVDEVGGEA | 0.059216343 | 0.002531759 | 0.047764984 |
| 4694 | NVDEVGGEALGR | 0.000351675 | 0 | 0.000982079 |
| 4695 | VNVDEVGGEALGR | 0.000525594 | 0 | 0.000495799 |
| 4696 | VNVDEVGGEALGRL | 0.000381216 | 0.000612758 | 0.001319924 |
| 4697 | EEDEERALEEQLL | 0.000608107 | 0.003076653 | 3.16362E-05 |
| 4698 | EEDEERALEEQLLK | 0.000740493 | 0.015748626 | 0.000667386 |
| 4699 | EEEDEERALEEQLL | 0 | 0.0025757 | 0 |
| 4700 | EPTAEQLAQIAAENEEDEH | 0.002014351 | 0.000564474 | 0 |
| 4701 | LAQIAAENEEDEHS | 0.001904219 | 0 | 0 |
| 4702 | LAQIAAENEEDEHSVN | 0.004658609 | 1.17059E-05 | 0 |
| 4703 | DSIAIFSPTHGMVIRKPY | 0 | 0.00151032 | 0 |
| 4704 | IAIFSPTHGMVIR | 0 | 0.001634177 | 0 |
| 4705 | IAIFSPTHGMVIRKP | 0.000101197 | 0.004649662 | 0 |
| 4706 | IAIFSPTHGMVIRKPY | 0 | 0.008308406 | 9.10894E-05 |
| 4707 | MAPRTLLL | 0 | 0 | 0.002225353 |
| 4708 | VMAPRTLLL | 0.007571169 | 0.007936475 | 0.010142798 |
| 4709 | VMAPRTLVL | 9.14225E-05 | 0 | 0.005259948 |
| 4710 | VPGKSIVENIIN | 0 | 0.007922834 | 0 |
| 4711 | VEGTARGIVVYTGDR | 0.000325608 | 0.000217946 | 0.010519675 |
| 4712 | SSYYRGAHGIIVVYD | 0 | 3.70311E-05 | 0.022800684 |
| 4713 | SSDAQSDDVPDITS | 0 | 0.000519666 | 8.27715E-05 |
| 4714 | AIEITTNSQEAAAK | 0 | 0.000173277 | 0 |
| 4715 | DITSDSENSNFR | 0.001203333 | 0.027427053 | 0 |
| 4716 | DITSDSENSNFRN | 1.2698E-05 | 0.001343011 | 0 |
| 4717 | DITSDSENSNFRNE | 5.13419E-05 | 0.002577276 | 0 |
| 4718 | KDITSDSENSNFR | 3.36325E-05 | 0.001521073 | 0 |
| 4719 | IILKDITSDSEN | 0 | 0.002159476 | 0 |
| 4720 | IILKDITSDSENSN | 0.000368017 | 0.001370019 | 0.000588834 |
| 4721 | IILKDITSDSENSNFR | 0.000231356 | 0.004591196 | 0 |
| 4722 | ILKDITSDSEN | 0 | 0.002399536 | 0 |
| 4723 | ILKDITSDSENSN | 0.000358713 | 0.001578973 | 0 |
| 4724 | ILKDITSDSENSNFR | 0.000750405 | 0.00972317 | 0 |
| 4725 | SFHVVIGSQAEEGQYS | 0 | 0 | 0.002829758 |
| 4726 | DLGQHGLEED | 0.012320874 | 0.044700329 | 0 |
| 4727 | DLGQHGLEEDF | 0.001131696 | 0.004392624 | 0 |
| 4728 | DLGQHGLEEDFM | 0.562263078 | 0.500594956 | 0 |
| 4729 | DLGQHGLEEDFML | 0.596052468 | 0.671499972 | 1.93031E-05 |
| 4730 | DRNLPSDSQDLGQHGLEEDFML | 0.224371258 | 0.314877008 | 0 |
| 4731 | DSQDLGQHGLEEDFML | 0.058493785 | 0.056849516 | 0 |
| 4732 | LDRNLPSDSQDLGQHGLEEDFML | 0.068617474 | 0.163019007 | 0 |
| 4733 | LGQHGLEED | 5.13932E-05 | 0.00011059 | 0 |
| 4734 | LPSDSQDLGQHGLEEDFML | 0.87759429 | 1.015327669 | 0 |
| 4735 | PSDSQDLGQHGLEEDFML | 0.927007736 | 0.814907281 | 0 |
| 4736 | QDLGQHGLEEDFML | 0.789412318 | 1.130307427 | 0.000116923 |
| 4737 | RNLPSDSQDLGQHGLEEDFML | 0.054398024 | 0.078098209 | 0 |
| 4738 | RSLDRNLPSDSQDLGQHGLEEDFML | 0.358439291 | 0.643584285 | 0 |
| 4739 | SDSQDLGQHGLEEDFML | 0.099210604 | 0.303149497 | 0 |
| 4740 | SLDRNLPSDSQDLGQHGLEEDFML | 0.942236879 | 1.870663886 | 0 |
| 4741 | SQDLGQHGLEEDFML | 0.235163316 | 0.326074608 | 0 |
| 4742 | GQHGLEEDFML | 0.014337321 | 0.005293759 | 0 |
| 4743 | LGQHGLEEDFML | 0.019526791 | 0.039763237 | 0 |
| 4744 | QHGLEEDFML | 0.002321974 | 0.007459989 | 0 |
| 4745 | DRNLPSDSQDLGQHGLEED | 0.006034376 | 0.019554329 | 0 |
| 4746 | DRNLPSDSQDLGQHGLEEDFM | 0.156817775 | 0.215369864 | 0 |
| 4747 | DSQDLGQHGLEED | 0.001327525 | 0.001348433 | 0 |
| 4748 | LDRNLPSDSQDLGQHGLEEDFM | 0.014169884 | 0.036322667 | 0 |
| 4749 | LPSDSQDLGQHGLEED | 0.048095382 | 0.092462391 | 0 |
| 4750 | LRSLDRNLPSDSQDLGQHGLEEDFM | 0.153441441 | 0.42052835 | 0 |
| 4751 | NLPSDSQDLGQHGLEED | 0.003177514 | 0.014244406 | 0 |
| 4752 | PSDSQDLGQHGLEED | 0.004861462 | 0.011425924 | 5.81437E-05 |
| 4753 | QDLGQHGLE | 0.000979538 | 0.002664548 | 0 |
| 4754 | QDLGQHGLEED | 0.088366427 | 0.214854044 | 0 |
| 4755 | RNLPSDSQDLGQHGLEED | 0.001027204 | 0.000592202 | 0 |
| 4756 | RSLDRNLPSDSQDLGQHGLEEDFM | 0.042789775 | 0.060361061 | 0 |
| 4757 | SDSQDLGQHGLEED | 0.002981648 | 0.017901252 | 0.00060096 |
| 4758 | SLDRNLPSDSQDLGQHGLEEDFM | 0.203913273 | 0.305371695 | 0 |
| 4759 | SQDLGQHGLEED | 0.006539509 | 0.011949713 | 0.000146028 |
| 4760 | DSQDLGQHGLEEDFM | 0.061806846 | 0.056279945 | 0.000368589 |
| 4761 | GQHGLEEDFM | 0.016182909 | 0.004865554 | 0 |
| 4762 | LGQHGLEEDFM | 0.060859909 | 0.030677995 | 0 |
| 4763 | LPSDSQDLGQHGLEEDFM | 0.605046375 | 0.471382363 | 0 |
| 4764 | PSDSQDLGQHGLEEDFM | 0.157913196 | 0.173938673 | 0 |
| 4765 | QDLGQHGLEEDFM | 0.810522285 | 0.967422794 | 9.59695E-05 |
| 4766 | QHGLEEDFM | 0.009051882 | 0.000783856 | 0 |
| 4767 | RNLPSDSQDLGQHGLEEDFM | 0.009840901 | 0.02547815 | 0 |
| 4768 | SDSQDLGQHGLEEDFM | 0.052540028 | 0.073123191 | 0 |
| 4769 | SQDLGQHGLEEDFM | 0.239215911 | 0.213360413 | 0 |
| 4770 | LPSDSQDLGQHGLEEDF | 0.001249651 | 0.003996318 | 0.000117419 |
| 4771 | QDLGQHGLEEDF | 0.005251384 | 0.008420489 | 0 |
| 4772 | SLDRNLPSDSQDLGQHGLEEDF | 0.003704422 | 0.006945827 | 0 |
| 4773 | LDRNLPSDSQDLGQHGLEED | 0.001436253 | 0.010352205 | 0 |
| 4774 | LPSDSQDLGQH | 0.000128441 | 0.000318107 | 0 |
| 4775 | LPSDSQDLGQHGLE | 0.000668506 | 0.001476048 | 0 |
| 4776 | LRSLDRNLPSDSQDLGQHGLEED | 0.026319872 | 0.093714568 | 0 |
| 4777 | LRSLDRNLPSDSQDLGQHGLEEDF | 0 | 0.003670774 | 0 |
| 4778 | RSLDRNLPSDSQDLGQHGLEED | 0.005561885 | 0.013759885 | 0 |
| 4779 | SLDRNLPSDSQDLGQHGLEED | 0.030855673 | 0.119717054 | 0 |
| 4780 | LRSLDRNLPSD | 0.001125072 | 0.005100315 | 0 |
| 4781 | LRSLDRNLPSDSQD | 0.000276396 | 0.00118307 | 0 |
| 4782 | LRSLDRNLPSDSQDLG | 0.000500188 | 0.0023031 | 0 |
| 4783 | LRSLDRNLPSDSQDLGQ | 0 | 0.000540308 | 0 |
| 4784 | SLDRNLPSD | 0.000997419 | 0.004962383 | 0 |
| 4785 | RSLDRNLPSDSQDLG | 0 | 0.000487952 | 0 |
| 4786 | SLDRNLPSDSQD | 0.000269619 | 0.002106811 | 0.000116055 |
| 4787 | GPITANSEQIARLR | 0 | 0.000765859 | 0 |
| 4788 | LKKLGSEGEDLIR | 0 | 0.000110547 | 0 |
| 4789 | FREKLQAANAEDIKSG | 8.87493E-05 | 0 | 0.002477744 |
| 4790 | FREKLQAANAEDIKSGK | 0 | 0 | 0.000303747 |
| 4791 | REKLQAANAEDIKSG | 0 | 0 | 0.001761546 |
| 4792 | LESAETEEVVDIF | 0 | 0 | 0 |
| 4793 | AIHTDTDNLG | 0 | 0.002476163 | 0.001426785 |
| 4794 | EAIHTDTDNLG | 0 | 0.001233675 | 7.43932E-06 |
| 4795 | FVEAIHTDTDNLG | 0.000422382 | 0.01239788 | 0 |
| 4796 | FVEAIHTDTDNLGIRIP | 0 | 0 | 0 |
| 4797 | VEAIHTDTDNLG | 0 | 0.006837714 | 0.000905828 |
| 4798 | VEAIHTDTDNLGIR | 0 | 0.00269268 | 0 |
| 4799 | EPVPGIKAEPDE | 0.001041335 | 2.97368E-05 | 3.06831E-05 |
| 4800 | ASGDMRTNGDNYL | 0 | 0.007779962 | 0 |
| 4801 | ASGDMRTNGDNYLYEI | 0 | 0.000819531 | 0 |
| 4802 | GDMRTNGDNYL | 3.24802E-05 | 0.00540994 | 0 |
| 4803 | RASGDMRTNGDNYL | 0.000865262 | 0.000847177 | 0 |
| 4804 | FPPNNKGEIKTEGQELIT | 0 | 0.021836358 | 0 |
| 4805 | FPPNNKGEIKTEGQELITVIK | 0 | 0.008455901 | 0 |
| 4806 | GEIKTEGQELIT | 0 | 0.009223511 | 0 |
| 4807 | KGEIKTEGQELIT | 0.001604457 | 0.031169659 | 4.39624E-05 |
| 4808 | NKGEIKTEGQELIT | 0 | 0.043019971 | 0.000109934 |
| 4809 | PPNNKGEIKTEGQELIT | 0 | 0.005428017 | 0 |
| 4810 | GEIKTEGQELITVIK | 0 | 0.004554298 | 0 |
| 4811 | KGEIKTEGQELITVIK | 0 | 0.018909652 | 0 |
| 4812 | NKGEIKTEGQELITVIK | 0 | 0.021555565 | 0 |
| 4813 | PPNNKGEIKTEGQELITVIK | 0 | 0.001073067 | 0 |
| 4814 | KGEIKTEGQELI | 0 | 0.000621652 | 0 |
| 4815 | KGEIKTEGQELITV | 0 | 0.00093323 | 0 |
| 4816 | KGEIKTEGQELITVI | 0 | 0.007670449 | 0 |
| 4817 | NKGEIKTEGQELITVI | 0 | 0.005885478 | 0 |
| 4818 | NKGEIKTEGQELI | 0 | 0.000385594 | 0.000211598 |
| 4819 | NKGEIKTEGQELITV | 0 | 0.003102367 | 0 |
| 4820 | DQTDMHGDSEYNIM | 0.000259763 | 0.00165637 | 0 |
| 4821 | LDQTDMHGDSEYNIM | 0 | 0.000664243 | 0 |
| 4822 | APTSFGYDKP | 7.22924E-05 | 0 | 0 |
| 4823 | APTSFGYDKPHVL | 0.009791835 | 0 | 0 |
| 4824 | ITVIKAPTSFGYDKP | 0.003125896 | 0 | 0 |
| 4825 | LPFDHSRVI | 0 | 0 | 0.00078448 |
| 4826 | GQELITVIKAPTSFG | 0.001647589 | 0 | 0 |
| 4827 | DMTSEEVMSL | 0 | 0.001112645 | 0 |
| 4828 | DMTSEEVMSLM | 0 | 0.002537237 | 0 |
| 4829 | DMTSEEVMSLMS | 0 | 0.000888274 | 0 |
| 4830 | HLGDMTSEEVMSLMS | 0 | 0.011417603 | 0 |
| 4831 | HLGDMTSEEVMSLMSS | 0 | 0.00301539 | 0 |
| 4832 | HLGDMTSEEVMSLMSSL | 0 | 0.000232671 | 0 |
| 4833 | LGDMTSEEVMSLMS | 0 | 0.001889436 | 0 |
| 4834 | LGDMTSEEVMSLM | 0 | 0 | 0 |
| 4835 | HLGDMTSEEVMSL | 0 | 0.010495317 | 0.001394739 |
| 4836 | HLGDMTSEEVMSLM | 0 | 0.011110014 | 0.001267232 |
| 4837 | LGDMTSEEVMSL | 0 | 0.001225581 | 0 |
| 4838 | AEESDNVDSADAE | 0 | 0.000903543 | 0 |
| 4839 | ESDNVDSADAEEDDSDV | 6.86192E-05 | 0.003217364 | 0.000194175 |
| 4840 | NVDSADAEEDDSDV | 0 | 0.001384956 | 0 |
| 4841 | SDNVDSADAEEDDSDV | 0.000397877 | 0.002868045 | 0 |
| 4842 | GEDVETSKKW | 0 | 0.004518079 | 0 |
| 4843 | DPDDVITDIE | 0 | 0.000659802 | 0 |
| 4844 | DPDDVITDIEMVD | 0 | 6.59048E-05 | 0 |
| 4845 | FDPDDVITDIE | 0 | 0.000487514 | 0 |
| 4846 | QGKWDVFSENLLADVKG | 0 | 0.00028926 | 0 |
| 4847 | RTQNVLGEK | 0 | 0.000137044 | 0 |
| 4848 | IIADVRAAREQISIG | 0 | 0 | 0.005764074 |
| 4849 | IIADVRAAREQISIGG | 0 | 0 | 0.008054847 |
| 4850 | LADVKGARAA | 0 | 0 | 0.000226162 |
| 4851 | LLADVKGARAA | 0 | 0 | 0.000317831 |
| 4852 | LLADVKGARAALN | 0 | 0 | 0.000371918 |
| 4853 | SENLLADVKGARAAL | 0.000149979 | 0 | 0 |
| 4854 | IKTELISVSEVHPSR | 0 | 0.00011522 | 0.002825466 |
| 4855 | TELISVSEVHPSR | 0 | 0 | 0.026525922 |
| 4856 | KLLGELHTL | 0 | 0 | 0.000566522 |
| 4857 | VVGQLKADEDPIM | 0.000244636 | 0 | 0 |
| 4858 | EGDDVQTAAEEVLADG | 0 | 0.000878213 | 0 |
| 4859 | FIVPLTDLRIP | 0.028788623 | 1.19788E-05 | 0 |
| 4860 | TPDFIVPLTDLRIP | 0.019517191 | 0 | 0 |
| 4861 | TPDFIVPLTDLRIPS | 0.006833089 | 0 | 0 |
| 4862 | LPIYSEEIVEMYKG | 0 | 0.000862057 | 0 |
| 4863 | AGVDPSTGKVQLTRDII | 0 | 0 | 0.007778245 |
| 4864 | DPSTGKVQLTRD | 0 | 3.3271E-05 | 0.001071856 |
| 4865 | DPSTGKVQLTRDI | 0 | 1.24195E-05 | 0.000782282 |
| 4866 | DPSTGKVQLTRDII | 7.89568E-05 | 0 | 0.010656856 |
| 4867 | GVDPSTGKVQLTRDII | 0 | 0 | 0.050535926 |
| 4868 | VDPSTGKVQLTRDII | 0 | 0 | 0.020905866 |
| 4869 | PSTGKVQLTRDII | 0 | 0 | 0.000867937 |
| 4870 | GVDPSTGKVQLTR | 0 | 0.000198625 | 0.00094829 |
| 4871 | GVDPSTGKVQLTRDIIK | 0 | 0 | 0.005489894 |
| 4872 | VDPSTGKVQLTR | 0 | 0 | 0.000354487 |
| 4873 | VDPSTGKVQLTRD | 0 | 0 | 0.000223337 |
| 4874 | VDPSTGKVQLTRDI | 0 | 0 | 0.001031886 |
| 4875 | EEAGARVQQNVPS | 0 | 0 | 0.00212866 |
| 4876 | EEAGARVQQNVPSG | 0 | 0 | 0.001911289 |
| 4877 | EESGARINI | 0 | 0.00159839 | 0 |
| 4878 | RIREESGARINISEGN | 0.000182208 | 0 | 0.001201665 |
| 4879 | VPTDFSSAKIEVS | 0 | 0 | 0.006589591 |
| 4880 | VPTDFSSAKIEVSQ | 0 | 0.00049139 | 0.020537496 |
| 4881 | VPTDFSSAKIEVSQL | 0 | 0.00013986 | 0.008166328 |
| 4882 | VPTDFSSAKIEVSQLL | 0 | 0 | 0.023426199 |
| 4883 | VPTDFSSAKIEVSQLLK | 0 | 0 | 0.005675738 |
| 4884 | GPSKVDINTEDLEDG | 0 | 0.024258673 | 0 |
| 4885 | GPPKLDIRKEEKQIMIDIF | 0.015687048 | 0 | 0 |
| 4886 | GPPKLDIRKEEKQIMIDIFH | 0.010534965 | 0 | 0 |
| 4887 | GPPKLDIRKEEKQIMIDIFHP | 0.021437195 | 0 | 0 |
| 4888 | DIPQAAKVNMTELDK | 0 | 0.000517533 | 0.000760949 |
| 4889 | DEPEWVKTEREQFVEFR | 0 | 0.000350882 | 0 |
| 4890 | DSEWIRSQEA | 0.000652004 | 0.000486881 | 7.07518E-05 |
| 4891 | DSEWIRSQEAG | 5.83532E-05 | 0.002785449 | 0 |
| 4892 | DSEWIRSQEAGGR | 0 | 0.000499124 | 0 |
| 4893 | DSEWIRSQEAGGRV | 0 | 0.003188053 | 0 |
| 4894 | DSEWIRSQEAGGRVV | 0 | 0.022504932 | 0 |
| 4895 | DSEWIRSQEAGGRVVP | 0.000100799 | 0.006311168 | 0 |
| 4896 | DSEWIRSQEAGGRVVPD | 0.000360471 | 0.005723512 | 0.001034113 |
| 4897 | DWRDSEWIRSQEAGGRVVPD | 0 | 0.000341221 | 0.018083051 |
| 4898 | DWRDSEWIRSQEAGGRVVPDS | 0 | 0 | 0.023021826 |
| 4899 | RDSEWIRSQEAGGRVVPD | 0 | 0.002400909 | 0 |
| 4900 | WRDSEWIRSQEAGGRVVPD | 0 | 0 | 0.006309401 |
| 4901 | WIRSQEAGGRVVPD | 0 | 4.65092E-05 | 0.004979395 |
| 4902 | IRSQEAGGRVVPD | 1.04407E-05 | 0 | 0.03084941 |
| 4903 | IRSQEAGGRVVPDS | 1.0288E-05 | 0.000340949 | 0.041378178 |
| 4904 | WIRSQEAGGRVVPDS | 0 | 0 | 0.00987678 |
| 4905 | WRDSEWIRSQEAGGRVVPDS | 0 | 0 | 0.004236579 |
| 4906 | RSQEAGGRVVPDS | 0 | 0 | 0.000859685 |
| 4907 | DWRDSEWIRSQEAGGRVV | 0 | 0.00074747 | 0 |
| 4908 | RDSEWIRSQEAGGRV | 0 | 0.001185913 | 0 |
| 4909 | RDSEWIRSQEAGGRVV | 0 | 0.009537028 | 0 |
| 4910 | SHDGNTDEPEWVK | 0 | 8.32483E-05 | 0 |
| 4911 | YSHDGNTDEPEWVK | 0 | 0.001066229 | 0 |
| 4912 | YSHDGNTDEPEWVKTER | 0 | 0.000406847 | 0 |
| 4913 | EPQFHNGESEESH | 0 | 0 | 0.000522728 |
| 4914 | EPQFHNGESEESHEQ | 0 | 0 | 0.001066581 |
| 4915 | AQITQRKWEAARVA | 0 | 0 | 0.005101584 |
| 4916 | AQITQRKWEAARVAEQLR | 0 | 0 | 0.000314371 |
| 4917 | ITQRKWEAARVA | 0.000378099 | 0 | 0.055684544 |
| 4918 | KWEAAHVA | 0 | 0 | 0.0036735 |
| 4919 | KWEAARVA | 0 | 0 | 0.106721037 |
| 4920 | QITQRKWEAARVA | 0 | 0.000615717 | 0.024499991 |
| 4921 | QRKWEAARVA | 2.28943E-05 | 0 | 0.621084484 |
| 4922 | RKWEAAHVA | 5.52739E-05 | 0 | 0.003421737 |
| 4923 | RKWEAARVA | 0 | 0 | 0.093647972 |
| 4924 | TQRKWEAARVA | 2.31629E-05 | 0 | 0.088816399 |
| 4925 | QITQRKWEAARVAE | 0 | 0 | 0 |
| 4926 | QITQRKWEAARVAEQ | 0 | 0 | 0.003676645 |
| 4927 | QITQRKWEAARVAEQD | 0.004898779 | 0 | 0.006175139 |
| 4928 | QITQRKWEAARVAEQDR | 0.00094898 | 0.00012017 | 0 |
| 4929 | ITQRKWEAARVAE | 5.24959E-05 | 0 | 0.00224919 |
| 4930 | ITQRKWEAARVAEQ | 0.002223781 | 0 | 0.015170402 |
| 4931 | ITQRKWEAARVAEQD | 0.009702752 | 0 | 6.24176E-05 |
| 4932 | ITQRKWEAARVAEQDR | 0.004590339 | 0 | 0 |
| 4933 | ITQRKWEAARVAEQDRA | 0.001159073 | 0 | 0 |
| 4934 | QRKWEAARVAE | 0 | 0 | 0.055138175 |
| 4935 | RKWEAAHVAE | 8.32589E-05 | 2.11368E-05 | 0.004275104 |
| 4936 | RKWEAARVAE | 0 | 2.71522E-05 | 0.011279375 |
| 4937 | TQRKWEAARVAE | 0 | 0 | 0.004816357 |
| 4938 | TQRKWEAARVAEQ | 0.000669365 | 0 | 0.0264239 |
| 4939 | HKWEAAHVAEQ | 0 | 0 | 0.000577113 |
| 4940 | HKWEAAHVAEQLR | 0 | 0 | 0.005456875 |
| 4941 | ITQRKWEAARVAEQLR | 0 | 0 | 0.027591125 |
| 4942 | ITQRKWEAARVAEQLRA | 0 | 0 | 0.007336072 |
| 4943 | KWEAAHVAE | 0 | 2.22206E-06 | 0.002558049 |
| 4944 | KWEAAHVAEQ | 0.000215283 | 0 | 0.005365792 |
| 4945 | KWEAARVAE | 6.38972E-05 | 0.000175576 | 0.008649745 |
| 4946 | KWEAARVAEQ | 0.000212038 | 1.33933E-05 | 0.049178887 |
| 4947 | QITQRKWEAARVAEQLRA | 0 | 0 | 0.00240084 |
| 4948 | QRKWEAARVAEQLR | 0 | 6.22102E-05 | 0.358832102 |
| 4949 | RKWEAAHVAEQ | 0.000851602 | 0 | 0.005679671 |
| 4950 | RKWEAARVAEQ | 0.000308295 | 0 | 0.067451652 |
| 4951 | RKWEAARVAEQL | 0 | 0 | 0.072719112 |
| 4952 | RKWEAARVAEQLR | 0 | 0 | 0.135311316 |
| 4953 | TQRKWEAARVAEQLR | 0 | 0 | 0.063802369 |
| 4954 | QRKWEAARVAEQ | 0.000972079 | 4.07244E-06 | 0.203052471 |
| 4955 | QRKWEAARVAEQD | 0.002735101 | 0 | 5.64509E-05 |
| 4956 | TQRKWEAARVAEQDR | 0.00078655 | 0 | 0 |
| 4957 | KRKWEAAHVAEQQ | 0 | 0 | 0.006036468 |
| 4958 | KRKWEAAHVAEQQR | 0 | 0 | 0.010473108 |
| 4959 | KWEAAHVAEQQ | 0 | 0 | 0.006393429 |
| 4960 | QITKRKWEAAHVAEQQR | 0 | 0 | 0.002970846 |
| 4961 | RKWEAAHVAEQQ | 0 | 2.53789E-05 | 0.014675142 |
| 4962 | TKRKWEAAHVAEQQR | 0 | 0 | 0.002887997 |
| 4963 | WEAAHVAEQQ | 0 | 1.34636E-05 | 0.0031034 |
| 4964 | RKWEAAHVAEQQR | 0 | 0 | 0.011402603 |
| 4965 | KWEAAHVAEQQR | 0 | 4.95721E-05 | 0.011376877 |
| 4966 | KWEAAHVAEQQRA | 0 | 0 | 0.000168517 |
| 4967 | RKWEAAHVAEQQRA | 0 | 0 | 0.000892671 |
| 4968 | TKRKWEAAHVAEQQRA | 0 | 0 | 0.000194686 |
| 4969 | WEAAHVAEQQR | 0 | 8.41075E-05 | 0.00271942 |
| 4970 | ITQRKWEAARVAEQL | 0 | 0 | 0.005675561 |
| 4971 | KWEAAHVAEQL | 0 | 3.30774E-05 | 0.004452085 |
| 4972 | KWEAARVAEQL | 0 | 0 | 0.007037017 |
| 4973 | QITQRKWEAARVAEQL | 0 | 0 | 0.004027356 |
| 4974 | QITQRKWEAARVAEQLR | 0 | 0 | 0.014831509 |
| 4975 | QRKWEAARVAEQL | 0 | 5.11944E-06 | 0.078344421 |
| 4976 | TQRKWEAARVAEQL | 7.54026E-05 | 0 | 0.008652801 |
| 4977 | WEAAHVAEQ | 2.39371E-05 | 0 | 0.003580324 |
| 4978 | WEAAHVAEQL | 0 | 0 | 0.010333224 |
| 4979 | WEAARVAEQ | 0 | 0 | 0.007705968 |
| 4980 | WEAARVAEQL | 0 | 0.000309166 | 0.002389188 |
| 4981 | KWEAAHVAEQLR | 0 | 0 | 0.001097028 |
| 4982 | KWEAARVAEQLR | 0 | 0 | 0.085784684 |
| 4983 | KWEAARVAEQLRA | 0 | 0.000936255 | 0.005974162 |
| 4984 | QRKWEAARVAEQLRA | 0 | 0 | 0.041748669 |
| 4985 | TQRKWEAARVAEQLRA | 0 | 0.000305464 | 0.00810495 |
| 4986 | WEAAHVAEQLR | 0 | 3.57763E-05 | 0.028530104 |
| 4987 | WEAARVAEQLR | 0 | 0 | 0.01712444 |
| 4988 | WEAAHVAEQLRA | 0 | 0 | 0.001840112 |
| 4989 | WEAARVAEQLRA | 0 | 0 | 0.001562023 |
| 4990 | ITKRKWEAAHVA | 2.6305E-05 | 0 | 0.002824427 |
| 4991 | ITKRKWEAAHVAE | 0.000107649 | 0 | 0.001986535 |
| 4992 | ITKRKWEAAHVAEQ | 0.001151825 | 0 | 0.007174878 |
| 4993 | ITKRKWEAAHVAEQQ | 0 | 0 | 0.009408208 |
| 4994 | ITKRKWEAAHVAEQQR | 0 | 0 | 0.015152509 |
| 4995 | ITKRKWEAAHVAEQQRA | 0 | 0 | 0.001619173 |
| 4996 | KHKWEAAHVA | 0 | 0 | 0.002224315 |
| 4997 | KRKWEAAHVA | 3.60954E-05 | 0 | 0.003913595 |
| 4998 | TKHKWEAAHVA | 0 | 0 | 0.0020398 |
| 4999 | TKRKWEAAHVA | 0 | 0 | 0.000456318 |
| 5000 | TKRKWEAAHVAEQQ | 0 | 0 | 0.000866596 |
| 5001 | KHKWEAAHVAE | 0 | 0 | 0.002056648 |
| 5002 | KHKWEAAHVAEQ | 0 | 0 | 0.000829434 |
| 5003 | KHKWEAAHVAEQL | 0 | 0 | 0.016563557 |
| 5004 | KHKWEAAHVAEQLR | 0 | 0 | 0.016805757 |
| 5005 | KRKWEAAHVAE | 0 | 0 | 0.000855991 |
| 5006 | KRKWEAAHVAEQ | 6.15077E-06 | 0 | 0.000551087 |
| 5007 | TKHKWEAAHVAEQLR | 0 | 0 | 0.009604059 |
| 5008 | TTKHKWEAAHVAEQLR | 0 | 0 | 0.002663445 |
| 5009 | TKHKWEAAHVAEQ | 0 | 0 | 0.000725035 |
| 5010 | TKHKWEAAHVAEQL | 8.80078E-06 | 1.62508E-05 | 0.004751922 |
| 5011 | TKRKWEAAHVAEQ | 2.52236E-06 | 0 | 0.000574391 |
| 5012 | TTKHKWEAAHVAEQ | 0 | 0 | 0.00026677 |
| 5013 | QITKRKWEAAHVA | 0 | 0 | 0.002886413 |
| 5014 | QITKRKWEAAHVAEQ | 0 | 0 | 0.000835644 |
| 5015 | QTTKHKWEAAHVA | 0 | 0 | 0.000588223 |
| 5016 | ITQRKWEAAREAEQ | 0 | 0 | 0.000213476 |
| 5017 | ITQRKWEAAREAEQLR | 0 | 0 | 0.000987681 |
| 5018 | QITQRKWEAAREAEQLR | 4.40647E-05 | 0 | 0.000902744 |
| 5019 | QRKWEAAREAEQ | 4.67687E-05 | 0 | 0.000347521 |
| 5020 | QRKWEAAREAEQLR | 0 | 0 | 0.003084748 |
| 5021 | RKWEAAREAEQLR | 0 | 0 | 0.000812176 |
| 5022 | TQRKWEAAREAEQLR | 0 | 0 | 0.00111781 |
| 5023 | DDWDEDAPAKIPDE | 0.003699309 | 0 | 0 |
| 5024 | DDWDEDAPAKIPDEE | 0.000224701 | 0 | 0 |
| 5025 | DWDEDAPAKIPDE | 0.001477029 | 0 | 0 |
| 5026 | KPDDWDEDAPAKIPDEE | 0.005587611 | 0 | 0.000107021 |
| 5027 | KPDDWDEDAPAKIPDE | 0.018936389 | 0.000366203 | 0.000704869 |
| 5028 | VKPDDWDEDAPAKIPDE | 0.001984849 | 0 | 0 |
| 5029 | HDGIVTAEELESY | 0.000597875 | 0.001779808 | 0 |
| 5030 | HDGIVTAEELESYM | 0.000125052 | 0.001441512 | 0 |
| 5031 | HDGIVTAEELESYMDP | 0.002341207 | 0.010549513 | 0 |
| 5032 | HDGIVTAEELESYMDPM | 0.006321023 | 0.001684572 | 0 |
| 5033 | NHDGIVTAEELESYMDP | 0 | 0.001259184 | 0.001079096 |
| 5034 | ILTGDQLLFE | 0 | 0.000513731 | 0 |
| 5035 | NPVISGEQIVQQ | 0.003725675 | 0 | 0.000828626 |
| 5036 | SPEDLIFEGAKVT | 0 | 0 | 0.002472574 |
| 5037 | ARSQVVFSAEELIYPDRR | 0 | 0.000352473 | 0 |
| 5038 | RSQVVFSAEELIYPDRR | 0.000298677 | 0.001591619 | 0 |
| 5039 | SQVVFSAEELIYPDRR | 0.004089965 | 0.003549912 | 0 |
| 5040 | AHQPRTADEIPMEPG | 0.000258964 | 0.001817886 | 0 |
| 5041 | IYAHQPRTADEIPMEPG | 0 | 0.003900498 | 0 |
| 5042 | QPRTADEIPMEPG | 3.51381E-05 | 0.01073185 | 0.000934415 |
| 5043 | YAHQPRTADEIPMEPG | 0 | 0.006937473 | 0 |
| 5044 | ATEEVSLDSPEREP | 0.00360064 | 0.001039342 | 0 |
| 5045 | ARPADEIAVDRDVPWGVD | 0 | 0.000615089 | 0 |
| 5046 | DEIAVDRDVPWG | 0.008775222 | 0.000155209 | 0 |
| 5047 | RPADEIAVDRDVPWG | 0.038880412 | 0.01033311 | 0 |
| 5048 | RPADEIAVDRDVPWGVD | 0.001734354 | 0.004705138 | 0 |
| 5049 | RPADEIAVDRDVPW | 0.000500668 | 0.000733597 | 0 |
| 5050 | EEYTDDIPLLK | 0 | 0.001719483 | 2.38248E-05 |
| 5051 | LTSEEYTDDIPLLK | 0 | 0.001935245 | 0 |
| 5052 | SEEYTDDIPLLK | 0 | 0.004636477 | 0 |
| 5053 | YTDDIPLLK | 0 | 0.000628536 | 0 |
| 5054 | ITGFSDDVPMVIA | 0.000112286 | 0.000340766 | 0 |
| 5055 | SYGDEIGLDAAALPGQP | 0.004398624 | 0 | 0.000185163 |
| 5056 | ALTFSYGRALQASAL | 0 | 0 | 0.006114408 |
| 5057 | EPFHQGHHSVDTAAMAG | 4.98246E-06 | 0.001053754 | 0 |
| 5058 | EPFHQGHHSVDTAAMAGL | 0 | 0.000602745 | 0.000187799 |
| 5059 | HQGHHSVDTAAM | 0 | 3.61314E-05 | 0 |
| 5060 | HQGHHSVDTAAMAG | 0 | 0.000492387 | 0 |
| 5061 | HQGHHSVDTAAMAGL | 0 | 0.000237642 | 0 |
| 5062 | APGLSPDTIMECAM | 0.000754772 | 0.00331096 | 0 |
| 5063 | APGLSPDTIMECAMG | 0.00080181 | 0.010118209 | 0 |
| 5064 | APGLSPDTIMECAMGD | 0.000569552 | 0.011126928 | 0 |
| 5065 | APGLSPDTIMECAMGDR | 0.005295855 | 0.090713603 | 0 |
| 5066 | APGLSPDTIMECAMGDRG | 0 | 0.009734462 | 0 |
| 5067 | GLSPDTIMECAMGDR | 0 | 0.000175329 | 0 |
| 5068 | FLGPLPANPVKVG | 0 | 0 | 0.001507818 |
| 5069 | IVKYNASSVTPESLR | 0 | 0.000563866 | 0 |
| 5070 | DNTYQATIGIDFLSKT | 0 | 0.000868289 | 0 |
| 5071 | EPYNATLSVHQL | 0 | 0.000685457 | 0 |
| 5072 | SDSFEAAVPSNSHIVS | 8.68862E-05 | 0.00056085 | 0.002700527 |
| 5073 | DPKMNGALPSDA | 0 | 5.41123E-05 | 0.009043524 |
| 5074 | DPKMNGALPSDAVG | 0 | 0 | 0.000619989 |
| 5075 | DPKMNGALPSDAVGYR | 0 | 0.000396184 | 0.002456469 |
| 5076 | QDPKMNGALPSDAVGYR | 5.88212E-05 | 0 | 0.008780249 |
| 5077 | EEAHLNTSF | 0 | 0.003092644 | 0 |
| 5078 | HGCQGGLPSQAFE | 0 | 0 | 0.004755447 |
| 5079 | HGCQGGLPSQAFEY | 0 | 0 | 0.002095251 |
| 5080 | HGCQGGLPSQAFEYI | 0 | 0 | 0.006142059 |
| 5081 | NHGCQGGLPSQAFEYI | 9.51684E-05 | 0.002615847 | 0.017896744 |
| 5082 | NHGCQGGLPSQAFEYIL | 0 | 0 | 0.001683634 |
| 5083 | NNHGCQGGLPSQAFEYIL | 0 | 0 | 0.001153451 |
| 5084 | NHGCQGGLPSQA | 8.85895E-05 | 0 | 0.001123837 |
| 5085 | NHGCQGGLPSQAFE | 0 | 9.8964E-05 | 0.023243818 |
| 5086 | NHGCQGGLPSQAFEY | 0.000146873 | 0.000157807 | 0.006421463 |
| 5087 | NNHGCQGGLPSQAFEY | 0 | 0 | 0.001462219 |
| 5088 | NNHGCQGGLPSQAFEYI | 0 | 0 | 0.012130839 |
| 5089 | NNHGCQGGLPSQA | 0 | 0 | 0.001202231 |
| 5090 | NNHGCQGGLPSQAFE | 0 | 3.08764E-05 | 0.020441274 |
| 5091 | LPSQAFEYILYNKG | 0.008875544 | 0.007578044 | 0.00555907 |
| 5092 | LPSQAFEYILYNKGI | 0.006223578 | 0.011259139 | 0.00732682 |
| 5093 | LPSQAFEYILYNKGIM | 0.003709606 | 0.012760991 | 0.008940051 |
| 5094 | LPSQAFEYILYNKGIMG | 0.000101881 | 0.001201663 | 0.018015818 |
| 5095 | APINAFIGGLAAQEVMK | 0 | 2.5943E-05 | 0.000487097 |
| 5096 | GGHMTTLSGEEIS | 0 | 0.000334476 | 0 |
| 5097 | AEDPLGAIHL | 0 | 0.001923386 | 0 |
| 5098 | EPVWRSEQAIGAIA | 0 | 0.000988582 | 0.000134533 |
| 5099 | RDEGISALW | 0 | 0.00040461 | 0 |
| 5100 | VTGEADVEFATHEDAVAAMSK | 0.00079887 | 0.000638606 | 0 |
| 5101 | DLDQQTLPGVAHQVVE | 0 | 0 | 0.001530775 |
| 5102 | DQQTLPGVAHQVVE | 0.000265605 | 0 | 0.008662024 |
| 5103 | EPANDIKEDAIAPRT | 0.000393747 | 9.37503E-06 | 0 |
| 5104 | EEKILRYALSNSIGPV | 0 | 9.17191E-05 | 0.007646476 |
| 5105 | QEEKILRYALSNSIGPV | 0 | 0 | 0.000452476 |
| 5106 | GPVSKYSQAVPAVT | 0 | 0 | 0.006501586 |
| 5107 | GPVSKYSQAVPAVTEGPIP | 0 | 0 | 0.00186273 |
| 5108 | KYSQAVPAVT | 0 | 0 | 0.000361347 |
| 5109 | SPDSIILYGQSIGTVPT | 0.000343471 | 4.91897E-05 | 0.000808195 |
| 5110 | AGVEGSLIVEKIM | 0 | 0.002069235 | 5.00316E-05 |
| 5111 | IVLDRLIEL | 0 | 0 | 0.000423106 |
| 5112 | DEREATDTPIVIQ | 0 | 0.00324632 | 0 |
| 5113 | DEREATDTPIVIQPA | 0 | 0.000753305 | 0 |
| 5114 | EREATDTPIVIQ | 0 | 0.001398075 | 0.000353213 |
| 5115 | SDEREATDTPIVIQPA | 0 | 0.003393865 | 0.000991052 |
| 5116 | SDEREATDTPIVIQ | 0 | 0.002093828 | 0.000541184 |
| 5117 | EREATDTPIVIQPA | 0 | 0.00013593 | 0 |
| 5118 | ENISRQTSETADNDN | 0 | 0.000257372 | 0 |
| 5119 | ENISRQTSETADNDNAS | 0 | 0.001347679 | 0 |
| 5120 | ISRQTSETADNDN | 0 | 0.000864937 | 0 |
| 5121 | NISRQTSETADNDN | 0 | 0.000333835 | 0 |
| 5122 | ISRQTSETADNDNAS | 0 | 0.002309778 | 0 |
| 5123 | NISRQTSETADNDNAS | 0 | 0.001169313 | 0 |
| 5124 | LVNKESSETPDQF | 0 | 0.016527463 | 0.00046951 |
| 5125 | LVNKESSETPDQFM | 0 | 0.006182438 | 0 |
| 5126 | LVNKESSETPDQFMT | 0 | 0.004862709 | 0 |
| 5127 | NKESSETPDQF | 0 | 0.000226104 | 0 |
| 5128 | VNKESSETPDQF | 0 | 0.004373017 | 0 |
| 5129 | VNKESSETPDQFM | 0 | 0.002442691 | 0 |
| 5130 | VNKESSETPDQFMT | 0 | 0.00212175 | 0 |
| 5131 | VHLVNKESSETPDQF | 0 | 0.001224897 | 0 |
| 5132 | QGTDSPEDFVIQ | 0 | 0.004285282 | 0 |
| 5133 | EETPVVLQL | 0 | 0.000581083 | 0 |
| 5134 | KVTSVVVDVVPR | 0 | 6.39141E-05 | 0 |
| 5135 | VPAVVIDMSGLREKDD | 0.00309929 | 0.000266826 | 0 |
| 5136 | DGEALSTLVLNRLK | 0 | 0.003820954 | 0 |
| 5137 | DGEALSTLVLNRLKVG | 0 | 0.000206044 | 0 |
| 5138 | GEALSTLVLNRL | 0 | 0.000412523 | 0 |
| 5139 | GEALSTLVLNRLK | 0 | 0.005604327 | 0 |
| 5140 | VDGEALSTLVLNRLK | 0 | 0.001424812 | 0 |
| 5141 | ILMEHIHKL | 0 | 2.96394E-05 | 0.003912755 |
| 5142 | APMYYRGAQA | 0 | 0 | 0.001536513 |
| 5143 | APMYYRGAQAA | 0 | 3.40494E-06 | 0.012884129 |
| 5144 | APMYYRGAQAAIVVYD | 0 | 0 | 0.043721811 |
| 5145 | APMYYRGAQAAIVVYDI | 0 | 0 | 0.002202593 |
| 5146 | YRGAQAAIVVYD | 0 | 0 | 0.026008729 |
| 5147 | YYRGAQAAIVVYD | 0 | 0.000146934 | 0.00933042 |
| 5148 | GKPTEGALIALAMK | 0.000226719 | 0 | 0.002190991 |
| 5149 | DGPKFLKSGDAAIVD | 0 | 0.000389204 | 0.007309027 |
| 5150 | DGPKFLKSGDAAIVDMVPG | 0 | 0 | 0.002349562 |
| 5151 | TPGEGSLIIRFSSPFDIA | 0 | 0 | 0.004330222 |
| 5152 | TPGEGSLIIRFSSPFDIAD | 0 | 0 | 0.003672167 |
| 5153 | TPGEGSLIIRFSSPFDIADT | 0.000109733 | 0 | 0.011612584 |
| 5154 | EPWLHSMYHGAAFQLTQG | 0 | 0 | 0.006566453 |
| 5155 | EPWLHSMYHGAAFQLTQGDQ | 0.000135535 | 0 | 0.021040303 |
| 5156 | LHSMYHGAAFQLTQ | 0 | 0.000836974 | 0.000505947 |
| 5157 | LHSMYHGAAFQLTQG | 0 | 0 | 0.003484108 |
| 5158 | LHSMYHGAAFQLTQGDQ | 0 | 0 | 0.002256769 |
| 5159 | DGFQPSRSIIF | 0 | 0.007024447 | 0.002211245 |
| 5160 | DGFQPSRSIIFA | 0 | 0.001624715 | 0.00012592 |
| 5161 | DGFQPSRSIIFASW | 0 | 0.0069581 | 0 |
| 5162 | DGFQPSRSIIFASWS | 0 | 0 | 0.021534961 |
| 5163 | DGFQPSRSIIFASWSA | 0 | 0 | 0.020003637 |
| 5164 | DGFQPSRSIIFASWSAG | 0 | 0 | 0.157072521 |
| 5165 | DGFQPSRSIIFASWSAGD | 0 | 0 | 0.005400584 |
| 5166 | DGFQPSRSIIFASWSAGDFG | 0 | 0 | 0.008795322 |
| 5167 | GFQPSRSIIFASWS | 0 | 0 | 0.007285747 |
| 5168 | GFQPSRSIIFASWSA | 0 | 0 | 0.002494203 |
| 5169 | GFQPSRSIIFASWSAG | 0 | 0 | 0.030778172 |
| 5170 | GFQPSRSIIFASWSAGD | 0 | 0 | 0.000612747 |
| 5171 | FQPSRSIIFASW | 0 | 0.000601697 | 0 |
| 5172 | FQPSRSIIFASWS | 0 | 0 | 0.002098152 |
| 5173 | FQPSRSIIFASWSA | 0 | 0 | 0.005935097 |
| 5174 | FQPSRSIIFASWSAG | 0 | 0 | 0.046347715 |
| 5175 | FQPSRSIIFASWSAGDFG | 0 | 0 | 0.001817431 |
| 5176 | QPSRSIIFASWSAG | 0.003518047 | 0.000241211 | 0.015932044 |
| 5177 | QPSRSIIFASWSAGDFG | 0.000177895 | 0.000125587 | 0.022754485 |
| 5178 | QPSRSIIFASWSAGDFGS | 0 | 0.000322338 | 0.003227506 |
| 5179 | KDGFQPSRSIIF | 0 | 0.001904669 | 0 |
| 5180 | KDGFQPSRSIIFASW | 2.78623E-05 | 0.001098495 | 0 |
| 5181 | LKDGFQPSRSIIFASW | 0 | 0.002973912 | 7.39151E-05 |
| 5182 | DPDAARAIVD | 0 | 0 | 0.002491344 |
| 5183 | ITAAQAGHAIITN | 0 | 0 | 0.001465637 |
| 5184 | TALITAAQAGHAI | 0 | 0 | 0.001882414 |
| 5185 | DPKISTSLPVLDLID | 0 | 0.000351129 | 0 |
| 5186 | LPRLTPPVL | 0 | 0 | 0.000830945 |
| 5187 | EPALAFWPGHIAPGVT | 0 | 0 | 0.002203361 |
| 5188 | EPALAFWPGHIAPGVTH | 0 | 0.000212857 | 0.005723694 |
| 5189 | EPALAFWPGHIAPGVTHE | 0 | 0 | 0.003973192 |
| 5190 | DPWEPFYVAGGKVP | 0 | 0 | 0.005600993 |
| 5191 | DPWEPFYVAGGKVPTFDE | 0 | 0 | 0.030026698 |
| 5192 | DPWEPFYVAGGKVPTFDER | 0.001969684 | 0 | 0.01495764 |
| 5193 | ENKHFAAASGKVSPT | 0 | 0 | 0.008120386 |
| 5194 | HFAAASGKVSPT | 0 | 0 | 0.002225745 |
| 5195 | KHFAAASGKVSPT | 0 | 0 | 0.001702063 |
| 5196 | NKHFAAASGKVSPT | 0 | 0 | 0.001200326 |
| 5197 | HFAAASGKVSPTA | 0 | 0 | 0.008006055 |
| 5198 | HFAAASGKVSPTASR | 0 | 0 | 0.007417922 |
| 5199 | KHFAAASGKVSPTASR | 0 | 0 | 0.003956706 |
| 5200 | KHFAAASGKVSPTA | 0 | 0 | 0.006052426 |
| 5201 | LAAARLAAA | 0.00191328 | 0 | 0 |
| 5202 | LPSPRLPTTDGAHPQPISPIP | 0 | 0.003094436 | 0.001506221 |
| 5203 | LPSPRLPTTDGAHPQPISPIPG | 0 | 0.003280366 | 0.001882336 |
| 5204 | SPTSGGGKVAPAQP | 0 | 0 | 0.002294302 |
| 5205 | SPTSGGGKVAPAQPS | 0.000343369 | 0 | 0.010993047 |
| 5206 | SPTSGGGKVAPAQPSEE | 0 | 0 | 0.005045811 |
| 5207 | SPTSGGGKVAPAQPSEEGPG | 0 | 0 | 0.001486803 |
| 5208 | IDFGEAARAD | 0 | 0 | 0.000873946 |
| 5209 | IDFGEAARADD | 0 | 0 | 0.00800023 |
| 5210 | IDFGEAARADDA | 0 | 0 | 0.001274949 |
| 5211 | IDFGEAARADDAR | 9.35164E-05 | 0 | 0.009729285 |
| 5212 | LGGPEAAKSDETA | 0 | 0 | 0.000510825 |
| 5213 | DRIEMWSYAAKVAPAD | 0 | 0.000366493 | 0.000814013 |
| 5214 | DRIEMWSYAAKVAPADGFS | 0 | 0 | 0.000642898 |
| 5215 | IEMWSYAAKVAPA | 0 | 0.000410459 | 0.002052898 |
| 5216 | IEMWSYAAKVAPAD | 0 | 0 | 0.032633795 |
| 5217 | IEMWSYAAKVAPADG | 0 | 0.000223201 | 0.01238433 |
| 5218 | IEMWSYAAKVAPADGFS | 6.55471E-05 | 0.000483648 | 0.016446988 |
| 5219 | WSYAAKVAPAD | 0 | 0.000127342 | 0.004603403 |
| 5220 | WSYAAKVAPADG | 0 | 0.00017577 | 0.002704319 |
| 5221 | WSYAAKVAPADGFS | 0 | 0 | 0.004561742 |
| 5222 | EPPTPLTVYAGAGSRVG | 0 | 0.001081955 | 0.002704887 |
| 5223 | DYTLKVSQAGKTL | 0 | 0 | 0.001024315 |
| 5224 | SPEDYTLKVSQAGKTL | 0 | 0 | 0.057736798 |
| 5225 | EDLNFVTDTAKALAD | 0 | 0.00076047 | 0 |
| 5226 | EDLNFVTDTAKALADV | 0.000175552 | 0.001352679 | 0.00051949 |
| 5227 | LAQAARGVAALTSDPA | 0.000201799 | 0 | 0.002123296 |
| 5228 | TPLLRPLASVSAQSFG | 0 | 0 | 0.003914815 |
| 5229 | TPLLRPLASVSAQSFGA | 0 | 0 | 0.001202702 |
| 5230 | TPLLRPLASVSAQSFGAL | 0 | 0 | 0.002789168 |
| 5231 | DPAFVASAYIPESLG | 0 | 0 | 0.000315582 |
| 5232 | DPAFVASAYIPESLGSL | 2.5366E-05 | 0 | 0.00010713 |
| 5233 | EPHFVGSAYVPESVG | 0 | 0.000497239 | 0.001589689 |
| 5234 | GGFMTTAFQYIIDN | 0 | 0.00319041 | 0.000497254 |
| 5235 | GGFMTTAFQYIIDNK | 0 | 0.00180915 | 0.00082341 |
| 5236 | NGGFMTTAFQYIIDNK | 0 | 0.000788423 | 0 |
| 5237 | AAVHRGVISNSGGPV | 0 | 0 | 4.00144E-05 |
| 5238 | KSTLITDGSTPIN | 0.002503996 | 0.001197173 | 0.000149229 |
| 5239 | NPANPAILSEASAPIPHDG | 0 | 0.003442401 | 0 |
| 5240 | YTSIHHGVVE | 0 | 3.53848E-06 | 0.001219052 |
| 5241 | GVVTQGASRL | 0 | 0.000111688 | 0 |
| 5242 | GVVTQGASRLA | 0 | 0.000304019 | 0 |
| 5243 | GVVTQGASRLAS | 0 | 0.000221596 | 0 |
| 5244 | GVVTQGASRLASH | 0 | 0.000652124 | 0.001071522 |
| 5245 | GVVTQGASRLASHE | 0 | 0.000547878 | 0 |
| 5246 | VTGVVTQGASRLASHE | 0 | 0.001295081 | 0.000638756 |
| 5247 | VTGVVTQGASRLA | 0 | 0.000866679 | 0 |
| 5248 | VTGVVTQGASRLAS | 0 | 0.000251398 | 0 |
| 5249 | VTGVVTQGASRLASH | 1.32487E-05 | 0.00173779 | 8.08903E-05 |
| 5250 | ITDSAGHILY | 0.014972724 | 0 | 7.39591E-05 |
| 5251 | SEAGSHIIQRM | 0 | 0.002136857 | 0 |
| 5252 | ISALLADPTRRF | 0.002432081 | 0 | 0.001544186 |
| 5253 | ISALLADPTRRFI | 6.07975E-05 | 0 | 0.001077956 |
| 5254 | ISALLADPTRRFIYV | 0.000514123 | 0 | 0.001097935 |
| 5255 | VISALLADPTRRF | 0 | 0 | 0.001305757 |
| 5256 | VAGLVAAKVLSDAGHK | 0 | 0 | 0.003606968 |
| 5257 | APPGVETDLID | 0 | 0.001339224 | 0 |
| 5258 | APPGVETDLIDVG | 0 | 0.038320921 | 5.15035E-05 |
| 5259 | APPGVETDLIDVGF | 0 | 0.003327859 | 0 |
| 5260 | APPGVETDLIDVGFT | 0 | 0.026836283 | 0 |
| 5261 | APPGVETDLIDVGFTD | 0 | 0.001352991 | 0 |
| 5262 | APPGVETDLIDVGFTDD | 0 | 0.001454851 | 0 |
| 5263 | APPGVETDLIDVGFTDDVK | 0 | 0.004463265 | 0.000165788 |
| 5264 | SPIETDVVPDTR | 0 | 0.006093889 | 0 |
| 5265 | SPIETDVVPDTRL | 0 | 0.00291454 | 0 |
| 5266 | SSPIETDVVPDTRL | 0 | 0.000160065 | 0 |
| 5267 | LEADIIGDTSGHFQ | 0.002174482 | 0 | 6.20369E-05 |
| 5268 | VPIVEPEILPDGDHDLK | 0.002230902 | 0.003306373 | 6.24938E-05 |
| 5269 | DAYERDLEADIIG | 0.004243754 | 0.003087706 | 0 |
| 5270 | DAYERDLEADIIGDT | 0 | 0.020631233 | 0 |
| 5271 | KDAYERDLEADIIGDT | 4.90323E-05 | 0.035079067 | 5.83474E-05 |
| 5272 | YKDAYERDLEADIIGDT | 0.000171363 | 0.001294475 | 0 |
| 5273 | KDAYERDLEADIIG | 0.006219128 | 0.004858349 | 0.00010012 |
| 5274 | KDAYERDLEADIIGD | 0 | 0.001173879 | 0 |
| 5275 | YKDAYERDLEADIIG | 0.005037235 | 0.00028252 | 0 |
| 5276 | DPLGYEDVIPEADREG | 0.001009093 | 0.006745292 | 0 |
| 5277 | DVIPEADREGVIR | 0.008487891 | 4.27411E-05 | 0.000117181 |
| 5278 | EDVIPEADREGVIR | 0.005088802 | 0.000732362 | 0.000486399 |
| 5279 | VIPEADREGVIR | 0.000824625 | 0 | 0 |
| 5280 | EAAWTLLEPELK | 0 | 0.003321428 | 0 |
| 5281 | QEAAWTLLEPELK | 0 | 0.002342736 | 0 |
| 5282 | WTLLEPELK | 0 | 0.000903278 | 0 |
| 5283 | LPVSEVASILQADL | 4.25501E-05 | 0.000408463 | 0 |
| 5284 | LPVSEVASILQADLQ | 4.15084E-05 | 0.000427018 | 0 |
| 5285 | EIIHKALIDRN | 0 | 0 | 0.006045211 |
| 5286 | EIIHKALIDRNIQ | 0 | 0.001361294 | 0.003852729 |
| 5287 | FREIIHKALIDRNIQ | 0 | 0 | 0.001635757 |
| 5288 | FREIIHKALIDRN | 0 | 0 | 0.002556236 |
| 5289 | DLEKDIRSDTSGHFE | 3.53242E-05 | 0.006669654 | 0 |
| 5290 | WEKWYIPDPTGKFN | 0.004157121 | 0.000551476 | 0.001863832 |
| 5291 | WEKWYIPDPTGKFNLI | 0.000608482 | 0 | 0.000251684 |
| 5292 | DLEKDIISDTSGDFR | 0 | 0.0052965 | 0 |
| 5293 | DLEKDIISDTSGDFRK | 0 | 0.000115534 | 0 |
| 5294 | KDIISDTSGDFR | 0 | 0.001819515 | 0 |
| 5295 | LEKDIISDTSGDFR | 3.56335E-05 | 0.011349821 | 0.000397269 |
| 5296 | DPSGQWRDTRVYGVFS | 0 | 0.000313548 | 0 |
| 5297 | LPDPSGQWRDTRVYGVFS | 0 | 0.003080453 | 0.000201071 |
| 5298 | LPDPSGQWRDTRVYGVFSNP | 0 | 0.003605235 | 0 |
| 5299 | GDDISSETSGDFR | 0.000120623 | 0.002317189 | 0 |
| 5300 | LGDDISSETSGDFR | 0 | 0.004037635 | 0 |
| 5301 | LGDDISSETSGDFRK | 0 | 0.000603965 | 0 |
| 5302 | AAAFAGLSREEALR | 0 | 0 | 0.001740998 |
| 5303 | DAAAAFAGLSREEALR | 0 | 0 | 0.000864736 |
| 5304 | DAAAAFAGLSREE | 0 | 0 | 0.0025669 |
| 5305 | DAAAAFAGLSREEA | 0 | 0.001467936 | 0.004914844 |
| 5306 | SDAAAAFAGLSREEA | 0.001325965 | 0 | 0.004902051 |
| 5307 | TWSDAAAAFAGLSREEA | 0 | 0 | 0.001360359 |
| 5308 | WSDAAAAFAGLSREEA | 0 | 0.005996236 | 0.013711948 |
| 5309 | SDAAAAFAGLSR | 0 | 0 | 0.000252912 |
| 5310 | WSDAAAAFAGLSR | 0 | 0.000183144 | 0.011206774 |
| 5311 | WSDAAAAFAGLSRE | 0 | 0 | 0.003066074 |
| 5312 | WSDAAAAFAGLSREE | 0 | 0 | 0.009159206 |
| 5313 | APPPYSSISAESA | 0.000604707 | 0.003722723 | 0.000306379 |
| 5314 | APPPYSSISAESAAY | 0.002550644 | 0.005343369 | 0 |
| 5315 | APPPYSSISAESAAYF | 0.004129053 | 0.010564085 | 0 |
| 5316 | APPPYSSISAESAAYFDYK | 0.035981729 | 0 | 0 |
| 5317 | GVNYASITRN | 0 | 0 | 0.005067034 |
| 5318 | GVNYASITRNQ | 0 | 0 | 0.001249931 |
| 5319 | GVNYASITRNQH | 0 | 2.89445E-05 | 0.005434108 |
| 5320 | NVDGVNYASITRNQH | 4.68649E-05 | 0.000309448 | 0.048618211 |
| 5321 | NVDGVNYASITRNQHIPQ | 0 | 7.83904E-05 | 0.046834633 |
| 5322 | VDGVNYASITRNQH | 0 | 0.000754197 | 0.012233724 |
| 5323 | VDGVNYASITRNQ | 0 | 0 | 0.003041041 |
| 5324 | VDGVNYASITRNQHIPQ | 0 | 0 | 0.005925367 |
| 5325 | NVDGVNYASITRN | 0 | 0 | 0.000534476 |
| 5326 | NVDGVNYASITRNQ | 0 | 0.000111579 | 0.009555934 |
| 5327 | VDGVNYASITRN | 0 | 0.000820614 | 0.003840493 |
| 5328 | DPAHSYTSVMVDG | 7.24144E-05 | 0.000411255 | 0 |
| 5329 | DPAHSYTSVMVDGEL | 0.000212725 | 0.001623642 | 0 |
| 5330 | DPAHSYTSVMVDGELY | 0.001350489 | 0.006478455 | 0 |
| 5331 | LPFSPLVI | 0 | 0 | 0.001777999 |
| 5332 | SPATFAGLVLVGLG | 0 | 0 | 0.000155013 |
| 5333 | AGLLLLGLAASSVR | 0 | 0 | 0.001390839 |
| 5334 | LGLAASSVRSNL | 0 | 0 | 0.002459159 |
| 5335 | LVGGASLKPEFVDIIN | 0 | 0.00286594 | 0 |
| 5336 | FRWQLESLKSQEF | 0 | 0.000804192 | 0 |
| 5337 | FRWQLESLKSQEFVE | 0 | 0.003959628 | 0.00046419 |
| 5338 | LEALKTELEDTL | 0 | 0.000991279 | 0 |
| 5339 | NVEPIHADIL | 0.001775998 | 0.000128841 | 0.00039906 |
| 5340 | YLQMNSLRAEDT | 0.00080417 | 6.24379E-05 | 0 |
| 5341 | YLQMNSLRAEDTA | 0.001927135 | 8.34289E-05 | 0.000171336 |
| 5342 | YLQMNSLRAEDTAV | 0.001889229 | 0.002396462 | 0.000698223 |
| 5343 | RVWSHQTLKSDVL | 0 | 0.001238485 | 0 |
| 5344 | YSSVKAEML | 0.000186488 | 0.001429135 | 3.86001E-05 |
| 5345 | YSSVKAEMLH | 7.6545E-05 | 0.000792006 | 0 |
| 5346 | GGLRSQLEAIFLR | 0 | 0.000131515 | 0 |
| 5347 | SQVEALFSY | 0 | 0.001468077 | 9.30461E-06 |
| 5348 | LPFNTVQGDIDAIF | 0 | 0 | 0 |
| 5349 | GGSFTVRTDTGEPMG | 0 | 0 | 0 |
| 5350 | GSFTVRTDTGEPM | 0 | 0.002831897 | 0 |
| 5351 | GSFTVRTDTGEPMG | 0 | 0.0072947 | 0 |
| 5352 | SFTVRTDTGEPM | 0 | 0.000962652 | 0 |
| 5353 | GSFTVRTDTGEPMGRG | 4.35425E-05 | 0.004917263 | 0 |
| 5354 | SFTVRTDTGEPMG | 0 | 0.004923185 | 0 |
| 5355 | SFTVRTDTGEPMGRG | 0 | 0.003327302 | 0 |
| 5356 | YSTLHTQSAEPPPPP | 0 | 0.000313394 | 0 |
| 5357 | IIEALHTDSPDDL | 0 | 0.000772397 | 0.000907763 |
| 5358 | VPREMKSNTSDPEIK | 0 | 0.0041788 | 0 |
| 5359 | EEEVDADAADAAAA | 0.002131146 | 0 | 0 |
| 5360 | EEEVDADAADAAAAE | 0.000760136 | 0 | 0.000138097 |
| 5361 | EVDADAADAAAAEEE | 0.000101794 | 0 | 0.000167126 |
| 5362 | VPEAASAEEQKEME | 0 | 0.000888366 | 0 |
| 5363 | VPEAASAEEQKEMED | 0 | 0.000145506 | 0 |
| 5364 | DIEQSIKSETSGSFE | 7.75015E-05 | 0.000515078 | 0 |
| 5365 | IEQSIKSETSGSFE | 0 | 0.001180578 | 0 |
| 5366 | AEAEQTLRF | 2.18443E-05 | 0.000480288 | 0 |
| 5367 | SEAEQTLRF | 0 | 0.000729838 | 0 |
| 5368 | EGGPDSDQAIRDR | 7.4162E-05 | 0.001615996 | 0 |
| 5369 | EGGPDSDQAIRDRF | 0 | 0.000700131 | 0 |
| 5370 | EGGPDSDQAIRDRFS | 3.54717E-05 | 0.003997268 | 0.000234156 |
| 5371 | GGPDSDQAIRDR | 6.31675E-05 | 0.001852708 | 0 |
| 5372 | GPDSDQAIRDR | 0 | 0.006235297 | 0 |
| 5373 | GGPDSDQAIRDRF | 0.00013144 | 0.0014866 | 6.68101E-05 |
| 5374 | GGPDSDQAIRDRFS | 0 | 0.001957034 | 0 |
| 5375 | GGPDSDQAIRDRFSR | 0 | 0.000763365 | 0.000368624 |
| 5376 | GPDSDQAIRDRF | 0 | 0.004015153 | 0 |
| 5377 | GPDSDQAIRDRFS | 0 | 0.004938062 | 0 |
| 5378 | GPDSDQAIRDRFSR | 4.91911E-05 | 0.001524109 | 7.38774E-05 |
| 5379 | FHPSSESEQGLTRPH | 0 | 0.000283396 | 5.6245E-05 |
| 5380 | FKLFHPSSESEQGLTRPH | 0 | 0.005301759 | 0 |
| 5381 | HPSSESEQGL | 0.000127262 | 0.000325685 | 0 |
| 5382 | HPSSESEQGLT | 0 | 0.000572915 | 0.00077911 |
| 5383 | HPSSESEQGLTRP | 0 | 0.000370226 | 0 |
| 5384 | HPSSESEQGLTRPH | 0 | 0.005180349 | 0 |
| 5385 | KLFHPSSESEQGLTRPH | 3.01054E-05 | 0.003209843 | 0 |
| 5386 | LFHPSSESEQGLTRPH | 0 | 0.011270837 | 0.000264891 |
| 5387 | LFHPSSESEQGL | 0 | 0 | 0 |
| 5388 | LFHPSSESEQGLT | 0 | 0.002679134 | 0 |
| 5389 | LFHPSSESEQGLTRP | 0 | 0.000559873 | 0 |
| 5390 | SSLSSSEGEEPVEYK | 7.17322E-05 | 0.000477342 | 0 |
| 5391 | APAPVPGGEAERVR | 0 | 0.000962478 | 0.004064289 |
| 5392 | APVPGGEAERVR | 0 | 0 | 0.001429215 |
| 5393 | ARFDSDVEVYRAVTPLGPP | 0 | 0.00025367 | 0.001468758 |
| 5394 | ARFDSDVEVYRAVTPLGPPD | 8.64027E-05 | 0.00011118 | 0.00631066 |
| 5395 | DSDVEVYRAVTPLGPP | 0 | 0 | 0.072317588 |
| 5396 | DVEVYRAVTPLGPP | 0 | 0 | 0.130843148 |
| 5397 | EVYRAVTPLGPP | 0.001373128 | 0 | 0.000854941 |
| 5398 | FDSDVEVYRAVTPLGPP | 0.000135833 | 0 | 0.009248985 |
| 5399 | RFDSDVEVYRAVTPLGPP | 0 | 0.000105326 | 0.002662302 |
| 5400 | SDVEVYRAVTPLGPP | 0 | 0 | 0.117663633 |
| 5401 | VEVYRAVTPLGPP | 0 | 0 | 0.037107079 |
| 5402 | VYRAVTPLGPP | 0.000129711 | 0 | 0.011156275 |
| 5403 | YARFDSDVEVYRAVTPLGPPD | 0 | 0 | 0.001557662 |
| 5404 | RFDSDVEVYRAVTPLGPPD | 0 | 0 | 0.006285824 |
| 5405 | DSDVEVYRAVTPLGPPD | 0 | 0.00182459 | 0.193652227 |
| 5406 | DSDVEVYRAVTPLGPPDA | 0 | 0 | 0.026730692 |
| 5407 | DSDVEVYRAVTPLGPPDAE | 0 | 0.000296575 | 0.012310623 |
| 5408 | DVEVYRAVTPLGPPD | 0 | 5.65494E-05 | 0.561132192 |
| 5409 | EVYRAVTPLGPPD | 0 | 0.00013001 | 0.015224125 |
| 5410 | FDSDVEVYRAVTPLGPPDAE | 0 | 0 | 0.002908633 |
| 5411 | SDVEVYRAVTPLGPPD | 0 | 0.00029171 | 0.345335325 |
| 5412 | VEVYRAVTPLGPPD | 0 | 0 | 0.134051628 |
| 5413 | VYRAVTPLGPPD | 0 | 0 | 0.057270954 |
| 5414 | SDVEVYRAVTPLGPPDA | 0 | 8.03893E-05 | 0.025569548 |
| 5415 | FDSDVEVYRAVTPLGPPD | 0 | 0 | 0.026898349 |
| 5416 | FDSDVEVYRAVTPLGPPDA | 0 | 6.21893E-05 | 0.004776846 |
| 5417 | DVEVYRAVTPLGPPDA | 0 | 0 | 0.023028701 |
| 5418 | DVEVYRAVTPLGPPDAE | 0 | 0 | 0.009499699 |
| 5419 | SDVEVYRAVTPLGPPDAE | 0 | 0 | 0.017727367 |
| 5420 | VEVYRAVTPLGPPDA | 0 | 0 | 0.012631452 |
| 5421 | VYRAVTPLGPPDA | 0 | 0 | 0.002431842 |
| 5422 | VEVYRAVTPLGPPDAE | 0 | 3.22777E-05 | 0.002282425 |
| 5423 | IPPAGQTEDAEPIS | 0.000182541 | 0.003470093 | 0 |
| 5424 | IPPAGQTEDAEPISEPE | 0 | 0.000577465 | 0 |
| 5425 | ALQSGNSQESVTEQ | 0 | 0.000309397 | 0.013952011 |
| 5426 | ALQSGNSQESVTEQD | 0 | 0 | 0.001853214 |
| 5427 | KVDNALQSGNSQESVTEQD | 0 | 0 | 0.013508186 |
| 5428 | KVDNALQSGNSQESVTEQDSKD | 0 | 0 | 0.001612346 |
| 5429 | KVDNALQSGNSQESVTEQDSKDS | 0 | 7.30408E-05 | 0.001223545 |
| 5430 | VDNALQSGNSQESVTEQD | 0 | 0 | 0.09091648 |
| 5431 | NALQSGNSQESVTEQ | 0 | 2.82024E-05 | 0.006168149 |
| 5432 | VDNALQSGNSQESVTEQ | 0 | 0 | 0.800625231 |
| 5433 | VDNALQSGNSQESVTEQDS | 0 | 0 | 0.010979891 |
| 5434 | VDNALQSGNSQESVTEQDSK | 0 | 0 | 0.002228873 |
| 5435 | VDNALQSGNSQESVTEQDSKD | 0 | 0 | 0.007557551 |
| 5436 | VDNALQSGNSQESVTEQDSKDS | 0 | 7.6129E-05 | 0.005303762 |
| 5437 | VDNALQSGNSQESVTEQDSKDST | 0 | 0.000189709 | 0.003181448 |
| 5438 | SGNSQESVTEQDSK | 0 | 0.000148609 | 0 |
| 5439 | SGNSQESVTEQDSKD | 0 | 0.000410864 | 0 |
| 5440 | SGNSQESVTEQDSKDS | 0 | 0.000929857 | 0 |
| 5441 | SGNSQESVTEQDSKDST | 0 | 0.002062604 | 0 |
| 5442 | DNALQSGNSQESVTE | 0 | 0 | 0.001260583 |
| 5443 | KVDNALQSGNSQESVTE | 0 | 0.000100087 | 0.006110115 |
| 5444 | KVDNALQSGNSQESVTEQ | 0 | 0 | 0.016736351 |
| 5445 | NALQSGNSQESVTE | 8.80432E-06 | 1.71729E-05 | 0.004756747 |
| 5446 | VDNALQSGNSQESVTE | 1.82401E-05 | 0 | 0.390139206 |
| 5447 | WKVDNALQSGNSQESVTEQ | 0 | 0 | 0.001729864 |
| 5448 | KVDNALQSGNSQESVT | 0 | 0 | 0.009889499 |
| 5449 | VDNALQSGNSQESVT | 0 | 0 | 0.292187682 |
| 5450 | WKVDNALQSGNSQESVT | 0 | 0.000222041 | 0.002602976 |
| 5451 | EDTVTDEEGKFR | 0 | 0.001682837 | 1.7433E-05 |
| 5452 | GEDTVTDEEGKFR | 0.000131495 | 0.000795547 | 0 |
| 5453 | YGEDTVTDEEGKFR | 0 | 0.00219463 | 0 |
| 5454 | DRLEETVQAK | 0.000155349 | 0.000223317 | 2.61936E-05 |
| 5455 | RLEETVQAK | 0.000714392 | 0.00129763 | 0.001455684 |
| 5456 | RVQEAVESMVK | 0 | 0.000943955 | 8.3897E-05 |
| 5457 | KVQWKVDNALQSGN | 0.001684667 | 0 | 0.00103983 |
| 5458 | KVQWKVDNALQSGNS | 0.000845732 | 0 | 0.000234542 |
| 5459 | ERESQDTVAENDDGGF | 0 | 0.004288902 | 0 |
| 5460 | ERESQDTVAENDDGGFS | 0 | 0.002514999 | 0 |
| 5461 | GEETVGEEDIR | 0 | 0.000183076 | 0 |
| 5462 | MQGEETVGEEDIR | 1.91218E-05 | 0.000485587 | 0.000207869 |
| 5463 | GPAGAAKDVTEESVTEDD | 0 | 0.0007399 | 0 |
| 5464 | DTDLFVGGVPEDQAA | 0 | 0 | 0.00186578 |
| 5465 | LPTIPEEEAK | 0 | 0.000721154 | 0 |
| 5466 | IPGINEAVAAVTEEEH | 0 | 0 | 0.001598253 |
| 5467 | ELNEPLSNEERNL | 0 | 0.002133147 | 0.000466465 |
| 5468 | LNEPLSNEERNL | 0 | 0.002274981 | 0 |
| 5469 | AGGDPQEAKPQEAAVAPE | 0 | 0 | 0.009008848 |
| 5470 | DPQEAKPQEAAVAPE | 0 | 0 | 0.002151288 |
| 5471 | ISEGNETVEDIAAR | 0 | 0.000775294 | 0 |
| 5472 | SIVTYTCDPDPEEGVN | 0 | 0.00110756 | 0 |
| 5473 | DPENTTWADIE | 0 | 0.002656715 | 0 |
| 5474 | DPENTTWADIEA | 0 | 0.001169945 | 0 |
| 5475 | DPENTTWADIEAM | 0 | 0 | 0 |
| 5476 | VSDPENTTWADIEAMV | 0 | 0.000305261 | 0 |
| 5477 | SDPENTTWADIE | 0 | 0.00054923 | 0 |
| 5478 | GNKQDSAFSNLENAL | 0 | 0.002146376 | 0 |
| 5479 | NKQDSAFSNLENAL | 0.000307683 | 0.004061909 | 0.000239119 |
| 5480 | KQDSAFSNLENALDLAP | 0 | 0.001442906 | 0 |
| 5481 | KQDSAFSNLENALDLAPS | 0 | 0.000888445 | 0.001516094 |
| 5482 | LDQDFHMEEALDWPGVY | 0 | 0.000326545 | 0 |
| 5483 | RLQEALNLF | 0 | 0 | 0.002478654 |
| 5484 | DWDHSWTEQSVD | 0 | 0 | 0.019240113 |
| 5485 | DWDHSWTEQSVDY | 0 | 0 | 0.002975806 |
| 5486 | DWDHSWTEQSVDYR | 0 | 0 | 0.028507724 |
| 5487 | DWDHSWTEQSVDYRH | 0 | 0 | 0.003825746 |
| 5488 | TDWDHSWTEQSVDYRH | 0 | 0 | 0.008807485 |
| 5489 | TDWDHSWTEQSVDY | 0 | 0 | 0.007662721 |
| 5490 | TDWDHSWTEQSVDYR | 0 | 0 | 0.080808977 |
| 5491 | RTDWDHSWTEQSVD | 0.000158692 | 5.2314E-05 | 0.006576572 |
| 5492 | RTDWDHSWTEQSVDYR | 0 | 0 | 0.002302093 |
| 5493 | TDWDHSWTEQSVD | 0 | 0 | 0.038000351 |
| 5494 | YRTDWDHSWTEQSVDYR | 0 | 0 | 0.000567076 |
| 5495 | YRTDWDHSWTEQSVD | 0 | 0 | 0.008286751 |
| 5496 | EHLVQYRTDWDHSWT | 0 | 0.00114133 | 0 |
| 5497 | EHLVQYRTDWDHSWTEQ | 0 | 0.007742346 | 0 |
| 5498 | HLVQYRTDWDHSWT | 0 | 0.008798705 | 0.000152606 |
| 5499 | HLVQYRTDWDHSWTE | 0.000264567 | 0.011208106 | 0 |
| 5500 | HLVQYRTDWDHSWTEQ | 0 | 0.016615536 | 0 |
| 5501 | LVQYRTDWDHSWTE | 0 | 0.014370069 | 0.000208125 |
| 5502 | LVQYRTDWDHSWTEQ | 0 | 0.010469353 | 0.000434873 |
| 5503 | KENPLQFKF | 3.38149E-05 | 0.000842386 | 0 |
| 5504 | DPLVPEIARIYKTD | 0 | 0.00418851 | 0.000584237 |
| 5505 | DPLVPEIARIYKTDR | 0 | 0 | 0.001826572 |
| 5506 | DPLVPEIARIYKTDRD | 4.07469E-05 | 0 | 0.004255551 |
| 5507 | DPNPDDPLVPEIARIYKTDRD | 0 | 0 | 0.007715663 |
| 5508 | DPNPDDPLVPEIARIYKTDRDK | 0 | 0 | 0.000304497 |
| 5509 | DPNPDDPLVPEIARIYKTDR | 0 | 0 | 0.006690112 |
| 5510 | IADMGHLKY | 0.009616043 | 0.001271016 | 0.000232485 |
| 5511 | GSYDLNSNDPDPMPHPD | 0 | 0.003092432 | 0 |
| 5512 | SPEGSYDLNSNDPDPMPHPD | 0 | 0.000848279 | 0 |
| 5513 | SYDLNSNDPDPMPHPD | 0 | 0.002438226 | 0 |
| 5514 | AKEEKEESDDEAAVEEE | 0 | 0.000946081 | 0 |
| 5515 | AKEEKEESDDEAAVEEEE | 0 | 0.001231704 | 0 |
| 5516 | EEKEESDDEAAVEE | 0 | 0.005282437 | 0 |
| 5517 | EEKEESDDEAAVEEE | 0 | 0.002264151 | 0 |
| 5518 | KEEKEESDDEAAVEEE | 0 | 0.001093267 | 0 |
| 5519 | KEEKEESDDEAAVEE | 3.67315E-05 | 0.001163501 | 0 |
| 5520 | KEEKEESDDEAAVEEEE | 0 | 0.001451544 | 0 |
| 5521 | EEKEESDDEAAVEEEE | 0.000109968 | 0.002294534 | 0 |
| 5522 | EEKEESDDEAAVEEEEEEKKP | 0 | 0.000587959 | 0 |
| 5523 | GPRQDATLDEEED | 0 | 0.000430534 | 0 |
| 5524 | GPRQDATLDEEEDM | 0.00113236 | 0.000141612 | 0 |
| 5525 | LSGGQSEEEASIN | 1.14003E-05 | 0.003311771 | 0 |
| 5526 | LSGGQSEEEASINL | 0 | 0.000843005 | 0 |
| 5527 | LSGGQSEEEASINLN | 0 | 0.002128079 | 0.00039704 |
| 5528 | SEEEASINLNAINK | 0 | 0.000387284 | 0 |
| 5529 | SSSTQASLEIDSL | 0 | 0.002711141 | 0 |
| 5530 | SSTQASLEIDSL | 0 | 0.002049441 | 0 |
| 5531 | FPVAGADVQTLRER | 0 | 0 | 0.003450503 |
| 5532 | VFPVAGADVQTLRER | 0 | 0 | 0.007057278 |
| 5533 | VFPVAGADVQTLR | 0 | 5.41367E-05 | 0.002172669 |
| 5534 | VFPVAGADVQTLRE | 0 | 0 | 0.002631907 |
| 5535 | NTPLHWAVAAGNVN | 0 | 0 | 0.002955204 |
| 5536 | TPLHWAVAAGNVN | 0 | 0 | 0.009834015 |
| 5537 | TPLHWAVAAGNVNAVDK | 0 | 0.000241139 | 0.001549824 |
| 5538 | WAVAAGNVNAVDK | 0 | 0 | 0.000279779 |
| 5539 | NVDPVQHTY | 0.00064657 | 0 | 0 |
| 5540 | SSVDVQSVEPVDQRT | 5.20932E-05 | 0.000289996 | 0.0004157 |
| 5541 | SVDVQSVEPVDQRT | 4.16107E-05 | 0.000697353 | 0.000230601 |
| 5542 | DDGAEETEEEVVA | 0 | 0.012208291 | 0 |
| 5543 | DDGAEETEEEVVAENP | 0 | 0.000988468 | 0 |
| 5544 | GAEETEEEVVA | 0 | 0.002177365 | 0 |
| 5545 | GAEETEEEVVAEN | 0 | 0.00038736 | 0 |
| 5546 | GAEETEEEVVAENP | 0 | 0 | 0 |
| 5547 | FGEEAEEEA | 0.000902687 | 0.000122221 | 0.000729643 |
| 5548 | GPGAKSQESQEADEQL | 0 | 0.0003431 | 0 |
| 5549 | LPVLVDSDEEIMT | 0.000148895 | 0.000372569 | 0 |
| 5550 | DDFDLGDAVVDGENDDPRPP | 0 | 0.00257773 | 0 |
| 5551 | DFDLGDAVVDGENDDPRPP | 0 | 0.016568161 | 0 |
| 5552 | LGDAVVDGENDDPRPP | 0 | 0 | 0 |
| 5553 | DFDLGDAVVDGENDDPRPPNPP | 0 | 0.00519975 | 0 |
| 5554 | EETIIQTDEDVPGP | 0 | 0.000241134 | 0 |
| 5555 | RKESEETIIQTDEDVPGP | 0 | 0.000570031 | 0.000243091 |
| 5556 | APGDPEAALEDNLARIR | 0.003587497 | 0 | 0.000346334 |
| 5557 | DVDGTVEEDLGKS | 0.000216497 | 0 | 0 |
| 5558 | EVDVDGTVEEDLGKS | 0.001362528 | 0 | 0 |
| 5559 | VDVDGTVEEDLGKS | 0.003553681 | 0.001357714 | 3.73005E-05 |
| 5560 | APRSMQGAIM | 0 | 0.003042838 | 1.49892E-05 |
| 5561 | EAPRSMQGAIM | 0 | 0.011093038 | 1.21132E-05 |
| 5562 | YSEAPRSMQGAIM | 3.75436E-05 | 0.001426346 | 0 |
| 5563 | ARSIESTLDDLFR | 0.000348639 | 0.004047521 | 0.000925521 |
| 5564 | ETARSIESTLDDLFR | 0 | 0.000485328 | 0 |
| 5565 | RSIESTLDDLFR | 0 | 0.000778878 | 0.000156871 |
| 5566 | SIESTLDDLFR | 0 | 0.009555565 | 0 |
| 5567 | TARSIESTLDDLFR | 0.000890215 | 0.004229058 | 0.000221622 |
| 5568 | SIESTLDDLFRN | 0 | 0.001658521 | 0 |
| 5569 | SIESTLDDLFRNSD | 0 | 0.001416476 | 0 |
| 5570 | SIESTLDDLFRNSDV | 0 | 0 | 0 |
| 5571 | DASVEEEGVRR | 0 | 7.9764E-05 | 0 |
| 5572 | GGPMDASVEEEGVRR | 0 | 0.001007884 | 0 |
| 5573 | GGPMDASVEEEGVRRA | 0 | 0.001151143 | 1.25337E-05 |
| 5574 | GPMDASVEEEGVRR | 0 | 0.046876715 | 2.8376E-05 |
| 5575 | GPMDASVEEEGVRRA | 0 | 0.010188789 | 0 |
| 5576 | GGPMDASVEEEGVR | 0 | 0.002244467 | 0 |
| 5577 | GPMDASVEEEGVR | 0 | 0.036346963 | 0 |
| 5578 | RPIDTTMDEEQ | 0 | 0.001153647 | 0 |
| 5579 | RPIDTTMDEEQVE | 0 | 0.000333398 | 0 |
| 5580 | EAVAYGAAVQAAIL | 0 | 0 | 0.000139033 |
| 5581 | NPDEAVAYGAAVQAAIL | 0 | 0 | 0.019166479 |
| 5582 | NPDEAVAYGAAVQAAILS | 0 | 0 | 0.003755296 |
| 5583 | NPDEAVAYGAAVQAAILSG | 0.000245794 | 0.000292999 | 0.010591848 |
| 5584 | NPDEAVAYGAAVQAGVL | 0 | 0 | 0.002143243 |
| 5585 | PDEAVAYGAAVQAGVL | 0.009639437 | 0 | 0.000876021 |
| 5586 | PDEAVAYGAAVQ | 0 | 4.60185E-05 | 0.00276746 |
| 5587 | NPDEAVAYGAAV | 0 | 0 | 0.002387092 |
| 5588 | NPDEAVAYGAAVQ | 0 | 0.000174436 | 0.049446083 |
| 5589 | NPDEAVAYGAAVQAA | 0 | 0 | 0.002080148 |
| 5590 | ELNKSINPDEAVAYG | 0.002435949 | 0 | 0 |
| 5591 | GKELNKSINPDEAVAYG | 0.000597982 | 0 | 0 |
| 5592 | KSINPDEAVAYG | 0.01728236 | 0 | 0.000391188 |
| 5593 | LNKSINPDEAVAYG | 0.029112721 | 0 | 0.000124819 |
| 5594 | NKSINPDEAVAYG | 0.001571999 | 0 | 0 |
| 5595 | LNKSINPDEAVAYGA | 0.002887599 | 0.000108753 | 0 |
| 5596 | KSINPDEAVAYGA | 0.000206032 | 0.000213885 | 0 |
| 5597 | KSINPDEAVAYGAA | 0.004651083 | 0 | 0.000604396 |
| 5598 | LNKSINPDEAVAYGAA | 0.024343948 | 0 | 0.000440156 |
| 5599 | EPSRGINPDEAVAYG | 0.006182932 | 0 | 0.000229586 |
| 5600 | EPSRGINPDEAVAYGAA | 0.002315993 | 0 | 0 |
| 5601 | SPAVPFSGTIQGGL | 0.000287288 | 0 | 0.033744214 |
| 5602 | SPAVPFSGTIQGGLQ | 0 | 0 | 0.037945928 |
| 5603 | SPAVPFSGTIQGGLQD | 0 | 0 | 0.005840869 |
| 5604 | SPAVPFSGTIQGGLQDG | 0 | 8.45793E-05 | 0.008136243 |
| 5605 | GAAVQAAILSGDK | 0 | 0 | 0.002889457 |
| 5606 | GAAVQAAILSGDKS | 0 | 0 | 0.002060731 |
| 5607 | GIEGGLLASIR | 0 | 0 | 0.00060262 |
| 5608 | GPGLEGGVVGKSADFV | 0 | 0 | 0.002661528 |
| 5609 | FNLDTENAMTFQE | 0 | 0.000408819 | 0 |
| 5610 | DVQGTDASLDEELDR | 0 | 0.002379696 | 0.000141741 |
| 5611 | FPAVSALVSRTADA | 0 | 0 | 0 |
| 5612 | PAVSALVSRTADA | 0 | 0.000106721 | 0.001864889 |
| 5613 | PAVSALVSRTADAD | 0 | 2.80879E-05 | 0.00324663 |
| 5614 | LQLWDTWADEAEKALGPLLARD | 0.003562335 | 0 | 0 |
| 5615 | ANYSLNTDDPLIFK | 0 | 0.008612999 | 0 |
| 5616 | LNTDDPLIFK | 0 | 0.003931265 | 0 |
| 5617 | NYSLNTDDPLIFK | 0.003367341 | 0.01396441 | 0 |
| 5618 | YSLNTDDPLIFK | 0.000102369 | 0.002946647 | 0 |
| 5619 | NYSLNTDDPLIFKS | 0 | 0.000224296 | 0 |
| 5620 | LFSVESDDTDTENER | 3.07291E-05 | 0.000777983 | 0 |
| 5621 | APVDSIQAEEWYFG | 0 | 0.001530889 | 0 |
| 5622 | KLNTLETEEW | 0 | 0.0010289 | 0.000170258 |
| 5623 | VAKLNTLETEEW | 5.01337E-05 | 0.003737629 | 0.001169942 |
| 5624 | VAKLNTLETEEWFFK | 0 | 0.008301352 | 0.000306022 |
| 5625 | AVHLESLEGDGEEE | 0 | 0.006989367 | 0 |
| 5626 | AVHLESLEGDGEEET | 0 | 0.004720132 | 0.000116711 |
| 5627 | DPESEEEPDLP | 0 | 0.001792576 | 0 |
| 5628 | IDPESEEEPDLP | 0 | 0.000665569 | 0 |
| 5629 | IDPESEEEPDLPE | 0 | 0.001854365 | 0 |
| 5630 | IDPESEEEPDLPEVD | 0 | 0.006330543 | 0 |
| 5631 | LIDPESEEEPDLPEVD | 0 | 0.004853306 | 0 |
| 5632 | IDPESEEEPDL | 0 | 0.00014675 | 0 |
| 5633 | ESEEEPDLPEV | 0 | 0.00312679 | 0 |
| 5634 | LIDPESEEEPDLPEV | 0.000270044 | 0.003014662 | 0 |
| 5635 | PESEEEPDLPEV | 0 | 0.003716254 | 0 |
| 5636 | GDKWLTEEAQSEQQT | 0 | 0.001575942 | 0 |
| 5637 | DDVQVETIEPEPEP | 0 | 0.000857671 | 0 |
| 5638 | AVEWESNGQPENN | 0 | 0 | 0.012890105 |
| 5639 | AVEWESNGQPENNYK | 0 | 0.000230128 | 0.018967101 |
| 5640 | DIAVEWESNGQPENNYK | 0 | 6.13249E-05 | 0.000775984 |
| 5641 | IAVEWESNGQPENNYK | 0 | 0 | 0.005236172 |
| 5642 | PSDIAVEWESNGQPENNYK | 0.001533332 | 0.000115514 | 0.002715714 |
| 5643 | VEWESNGQPENN | 0 | 0 | 0.007268998 |
| 5644 | WESNGQPENN | 0 | 0 | 0.000411505 |
| 5645 | YPSDIAVEWESNGQPENNYK | 0 | 0 | 0.00609243 |
| 5646 | IAVEWESNGQPENN | 0 | 0 | 0.003361542 |
| 5647 | PSDIAVEWESNGQPENN | 0 | 0 | 0.000802767 |
| 5648 | YPSDIAVEWESNGQPENN | 0 | 0 | 0.004786156 |
| 5649 | YPSDIAVEWESNGQP | 0.000161435 | 0 | 0 |
| 5650 | YPSDIAVEWESNGQPE | 0.01435461 | 0 | 0 |
| 5651 | VEWESNGQPENNYK | 0 | 0 | 0.011702713 |
| 5652 | WESNGQPENNYK | 0 | 0 | 0.001880166 |
| 5653 | KGFYPSDIAVEWE | 0.000252513 | 0.000834528 | 0 |
| 5654 | KGFYPSDIAVEWESN | 0.000353742 | 0.002510713 | 0 |
| 5655 | VKGFYPSDIAVEWESN | 0.000634392 | 0.001934938 | 0 |
| 5656 | VKGFYPSDIAVEWE | 0.000668748 | 0.001606475 | 0 |
| 5657 | DFESETDTETIAK | 0.000814467 | 0.005201228 | 0.000636263 |
| 5658 | GGYFHIETDAQTNEGIV | 0 | 0.000318613 | 0 |
| 5659 | GYFHIETDAQTNEG | 0 | 0.009044128 | 0 |
| 5660 | GYFHIETDAQTNEGIV | 0 | 0.004020489 | 0 |
| 5661 | YFHIETDAQ | 0 | 0.000258535 | 0 |
| 5662 | YFHIETDAQTNEGIV | 0 | 0.004208058 | 0 |
| 5663 | LALSQSEAEEKER | 0 | 0.00021628 | 9.96563E-05 |
| 5664 | LALSQSEAEEKERLR | 0 | 0.000143282 | 0 |
| 5665 | NPQTQTEERF | 0.000106735 | 0.000214388 | 0 |
| 5666 | NPQTQTEERFR | 2.94863E-05 | 0.001042353 | 2.18052E-05 |
| 5667 | NPQTQTEERFRP | 0 | 0.000311022 | 0 |
| 5668 | HLPDAESDEDEDFK | 0 | 0.001540649 | 0 |
| 5669 | LPDAESDEDEDFK | 0 | 0.002796311 | 0 |
| 5670 | RQPLPDSNPEESSV | 0 | 0.000189363 | 0 |
| 5671 | DLKVHFLTDPENEM | 0.000824844 | 0.017666526 | 0 |
| 5672 | DLKVHFLTDPENEMK | 0.001756167 | 0.015946815 | 0 |
| 5673 | DLKVHFLTDPENEMKEK | 0.000544239 | 0.015987418 | 0 |
| 5674 | DLKVHFLTDPENEMKEKL | 0.000177162 | 0.017586583 | 0 |
| 5675 | EDLKVHFLTDPENEMKEKL | 0 | 0.005185463 | 0 |
| 5676 | KVHFLTDPENEM | 0.000452948 | 0.004269225 | 0 |
| 5677 | LKVHFLTDPENEM | 0 | 0.007769141 | 0.00048723 |
| 5678 | VHFLTDPENEM | 0 | 0.001848456 | 0 |
| 5679 | LKVHFLTDPENEMK | 0.000793203 | 0.010984465 | 0 |
| 5680 | KVHFLTDPENEMK | 0.002463214 | 0.007077301 | 9.19096E-05 |
| 5681 | KVHFLTDPENEMKE | 0.000915464 | 0.000301319 | 0 |
| 5682 | KVHFLTDPENEMKEK | 0.00036056 | 0.004021361 | 0 |
| 5683 | LKVHFLTDPENEMKEK | 0.000271886 | 0.007585676 | 0 |
| 5684 | LKVHFLTDPENEMKEKL | 7.85955E-05 | 0.00239165 | 0 |
| 5685 | VHFLTDPENEMK | 0.000239897 | 0.001759268 | 0 |
| 5686 | EDLKVHFLTDPENEM | 3.90604E-05 | 0.007453196 | 6.58861E-05 |
| 5687 | EDLKVHFLTDPENEMK | 0.00069654 | 0.012415416 | 0 |
| 5688 | EDLKVHFLTDPENEMKEK | 0 | 0.00464225 | 0 |
| 5689 | DTDSEEEIR | 1.47745E-05 | 0.000490166 | 0 |
| 5690 | DTDSEEEIRE | 0 | 0.001558853 | 0.00032206 |
| 5691 | DTDSEEEIREA | 0 | 0.002746565 | 0.000618938 |
| 5692 | DTDSEEEIREAF | 0 | 0.001552019 | 0 |
| 5693 | DTDSEEEIREAFR | 0 | 0.003071164 | 0 |
| 5694 | KMKDTDSEEEIREA | 0 | 0.000325996 | 0 |
| 5695 | LPRWKFCVSDTENNL | 0 | 0.001050902 | 0 |
| 5696 | LPRWKFCVSDTENNLG | 0 | 0.008335889 | 0 |
| 5697 | LPRWKFCVSDTENNLGFA | 0 | 0.001235045 | 0 |
| 5698 | EPGVWVAEAEDEPLL | 0.001810677 | 0.000547777 | 0.000304055 |
| 5699 | EPGVWVAEAEDEPLLT | 0 | 0 | 0 |
| 5700 | IKNMINTEEGITLENS | 0 | 0.000468671 | 9.3583E-05 |
| 5701 | DHSSQSEEEVVE | 0 | 0.001157146 | 0 |
| 5702 | DHSSQSEEEVVEG | 0 | 0.000714003 | 0 |
| 5703 | DHSSQSEEEVVEGE | 0 | 0.001146137 | 0 |
| 5704 | DHSSQSEEEVVEGEK | 0 | 0.002149089 | 0 |
| 5705 | DHSSQSEEEVVEGEKE | 0 | 0.00055902 | 0 |
| 5706 | RDHSSQSEEEVVE | 0 | 0.001154107 | 0 |
| 5707 | RDHSSQSEEEVVEG | 0 | 0.001159728 | 5.71209E-05 |
| 5708 | RDHSSQSEEEVVEGE | 0 | 0.001437485 | 2.11359E-05 |
| 5709 | RDHSSQSEEEVVEGEK | 0 | 0.004133309 | 0.000223844 |
| 5710 | SRDHSSQSEEEVVEGEK | 0 | 0.001543037 | 0 |
| 5711 | SRDHSSQSEEEVVE | 0 | 0.000456985 | 0 |
| 5712 | SRDHSSQSEEEVVEG | 0 | 0.00048648 | 3.24552E-06 |
| 5713 | SRDHSSQSEEEVVEGE | 0 | 0.000552867 | 0 |
| 5714 | TSRDHSSQSEEEVVEGE | 0 | 0.001661352 | 0 |
| 5715 | TSRDHSSQSEEEVVE | 0 | 0.001446084 | 0 |
| 5716 | TSRDHSSQSEEEVVEG | 0 | 0.002883345 | 0 |
| 5717 | KRATLDEEDLVDS | 0 | 0.000709883 | 0 |
| 5718 | RENEVLEAW | 0 | 0.000690376 | 0.000229919 |
| 5719 | KPKKENIIAFEEIIEPY | 0.002314664 | 0 | 0 |
| 5720 | EEVVEGEKEVEALK | 0.002389423 | 0 | 2.9745E-05 |
| 5721 | VSWLIDANHNMQ | 0.000653128 | 0.001803524 | 0 |
| 5722 | GIDLQTIHSREL | 0 | 0.004322518 | 0 |
| 5723 | GIDLQTIHSRELP | 8.90702E-05 | 0.015797346 | 0.006156034 |
| 5724 | GIDLQTIHSRELPD | 0 | 0.009294948 | 0 |
| 5725 | HEIEALKSF | 0 | 0.001145223 | 0 |
| 5726 | KELESIKETY | 0 | 0.001164618 | 0 |
| 5727 | LDTVKGSSLHVGSD | 0.000385221 | 0 | 0.004083823 |
| 5728 | LDTVKGSSLHVGSDL | 0 | 0 | 0.013935215 |
| 5729 | NLDTVKGSSLHVGSDL | 0 | 0 | 0.006694236 |
| 5730 | NLDTVKGSSLHVGSD | 0 | 0 | 0.002010859 |
| 5731 | ILSLLESNKDLLLT | 0 | 0.000443929 | 0 |
| 5732 | GSKPRAIVVDPVH | 0.001942378 | 0.00849163 | 0 |
| 5733 | GSKPRAIVVDPVHG | 0.002862976 | 0.010121708 | 0 |
| 5734 | KPRAIVVDPVH | 0.002514594 | 0.001240818 | 0 |
| 5735 | SKPRAIVVDPVHG | 0.000285896 | 0.000633101 | 0 |
| 5736 | KPRAIVVDPVHG | 0.004696911 | 0.001189114 | 0 |
| 5737 | KPRAIVVDPVHGF | 0.004329757 | 0 | 0.002513551 |
| 5738 | KPRAIVVDPVHGFM | 0.016920977 | 0 | 0.004468095 |
| 5739 | KPRAIVVDPVHGFMY | 0.002922655 | 0 | 0.000474542 |
| 5740 | HSEEASLDADQARTPD | 0 | 0.000227219 | 0.000428348 |
| 5741 | ADGPRSAQPLRPED | 0 | 0 | 0.000852716 |
| 5742 | GPRSAQPLRPED | 0 | 0 | 0.000374407 |
| 5743 | SADGPRSAQPLRPED | 2.94689E-05 | 0 | 0.004840665 |
| 5744 | SADGPRSAQPLRPEDG | 0 | 0 | 0.004453557 |
| 5745 | SADGPRSAQPLRPEDGH | 0 | 0 | 0.005450033 |
| 5746 | DGPRSAQPLRPEDGH | 0 | 0 | 0.000299872 |
| 5747 | DYQGGRTGEAIVD | 5.69718E-05 | 2.64378E-05 | 0.003281593 |
| 5748 | RPEDYQGGRTGEAIVD | 0 | 1.80848E-05 | 0.019228997 |
| 5749 | RPEDYQGGRTGEAIVDA | 0 | 5.76705E-05 | 0.005336202 |
| 5750 | ADPPKTHVTHHPISDHEVTLR | 0 | 0.001521934 | 0 |
| 5751 | DPPKTHMTHHPISDH | 0 | 0.000395843 | 0 |
| 5752 | DPPKTHMTHHPISDHE | 0 | 0.000103091 | 0 |
| 5753 | DPPKTHVTHHPISDHE | 0 | 0.001392437 | 0 |
| 5754 | DPPKTHVTHHPISDHEVTL | 0 | 0.010545597 | 0 |
| 5755 | DPPKTHVTHHPISDHEVTLR | 0 | 0.074683591 | 0 |
| 5756 | HVTHHPISDHEVTL | 0 | 0.001396466 | 0 |
| 5757 | VTHHPISDHEVTL | 0 | 0.013611475 | 0.000114272 |
| 5758 | HHPISDHEVTLR | 0 | 0.012010264 | 0 |
| 5759 | HPISDHEVTL | 0 | 0.003329979 | 4.51681E-05 |
| 5760 | HPISDHEVTLR | 0 | 0.029748698 | 9.2178E-06 |
| 5761 | HVTHHPISDHEVTLR | 0 | 0.007529496 | 0.000107089 |
| 5762 | PKTHVTHHPISDHEVTLR | 0 | 0.000266936 | 0 |
| 5763 | THHPISDHEVTLR | 0 | 0.003279713 | 0.000232331 |
| 5764 | THVTHHPISDHEVTLR | 0 | 0.00077352 | 0 |
| 5765 | VTHHPISDHEVTLR | 2.99938E-05 | 0.058899462 | 0.000104291 |
| 5766 | APKTHMTHHAVSDHEATL | 0 | 0.005281675 | 0 |
| 5767 | APKTHMTHHAVSDHEATLR | 0 | 0.018950296 | 0 |
| 5768 | DAPKTHMTHHAVSDHEATLR | 0 | 0.006309056 | 0 |
| 5769 | DPPKTHMTHHPISDHEATLR | 3.60166E-05 | 0.056769677 | 0 |
| 5770 | EPPKTHVTHHPLSDHEATLR | 0.00016969 | 0.010573226 | 0 |
| 5771 | HHAVSDHEATL | 0 | 0.002159309 | 0 |
| 5772 | HHPLSDHEATL | 0 | 0.008859474 | 4.04341E-06 |
| 5773 | HHPVSDHEATL | 0 | 0.000643877 | 0 |
| 5774 | HMTHHAVSDHEATL | 0 | 0.008004858 | 0 |
| 5775 | HPLSDHEATL | 0 | 0.046863141 | 0 |
| 5776 | HPVSDHEATL | 0 | 0.017545086 | 0 |
| 5777 | HVTHHPLSDHEATL | 0 | 0.002567016 | 6.42707E-05 |
| 5778 | HVTHHPVSDHEATL | 0 | 0.003553738 | 0 |
| 5779 | MTHHAVSDHEATL | 0 | 0.025665055 | 0 |
| 5780 | MTHHPISDHEATL | 0 | 0.004990042 | 0.000168896 |
| 5781 | PPKTHVTHHPLSDHEATLR | 0 | 0.000304476 | 0 |
| 5782 | TDAPKTHMTHHAVSDHEATLR | 0 | 0.000143383 | 0 |
| 5783 | TDPPKTHMTHHPISDHEATLR | 0 | 0.000590717 | 0 |
| 5784 | THHAVSDHEATL | 0.000193097 | 0.019650436 | 0 |
| 5785 | THHPLSDHEATL | 0.000242625 | 0.003949719 | 0 |
| 5786 | THMTHHAVSDHEATL | 0 | 0.005381746 | 0 |
| 5787 | THVTHHPLSDHEATL | 0 | 0.000285481 | 0 |
| 5788 | THVTHHPVSDHEATL | 0 | 0.001547225 | 8.52511E-05 |
| 5789 | VTHHPLSDHEATL | 2.52094E-05 | 0.016249425 | 0 |
| 5790 | VTHHPVSDHEATL | 0 | 0.005382555 | 1.14654E-05 |
| 5791 | DAPKTHMTHHAVSDHEATL | 0 | 0.001448793 | 0 |
| 5792 | DPPKTHMTHHPISDHEATL | 0 | 0.018235095 | 0 |
| 5793 | EPPKTHVTHHPLSDHEATL | 0 | 0.000589306 | 0 |
| 5794 | HPKTHVTHHPVSDHEATL | 0 | 0.003937329 | 0 |
| 5795 | AVSDHEATLR | 0 | 0.001831418 | 0.00011472 |
| 5796 | EHPKTHVTHHPVSDHEATLR | 0 | 9.47748E-05 | 0 |
| 5797 | HAVSDHEATLR | 0 | 0.003277346 | 0 |
| 5798 | HHAVSDHEATLR | 0 | 0.019889294 | 3.02318E-06 |
| 5799 | HHPLSDHEATLR | 0.000102422 | 0.025264288 | 2.29044E-05 |
| 5800 | HHPVSDHEATLR | 0 | 0.010084612 | 0 |
| 5801 | HMTHHAVSDHEATLR | 0 | 0.04893695 | 5.75902E-05 |
| 5802 | HMTHHPISDHEATLR | 0 | 0.001604604 | 0 |
| 5803 | HPKTHVTHHPVSDHEATLR | 0 | 0.00859638 | 0 |
| 5804 | HPLSDHEATLR | 0.000652339 | 0.203176797 | 0 |
| 5805 | HPVSDHEATLR | 0 | 0.116375034 | 0 |
| 5806 | HVTHHPLSDHEATLR | 0 | 0.005137213 | 0 |
| 5807 | HVTHHPVSDHEATLR | 0 | 0.016340387 | 6.99813E-06 |
| 5808 | KTHVTHHPVSDHEATLR | 0 | 0.000134896 | 0 |
| 5809 | MTHHAVSDHEATLR | 0 | 0.122579783 | 0.000181208 |
| 5810 | MTHHPISDHEATLR | 5.87795E-05 | 0.016511681 | 2.16236E-05 |
| 5811 | PKTHVTHHPLSDHEATLR | 0 | 0.000366044 | 0 |
| 5812 | PLSDHEATLR | 0 | 0.001231103 | 0 |
| 5813 | SDHEATLR | 0 | 6.81698E-05 | 0 |
| 5814 | THHAVSDHEATLR | 5.77462E-06 | 0.119309728 | 0 |
| 5815 | THHPLSDHEATLR | 0 | 0.010019343 | 0 |
| 5816 | THHPVSDHEATLR | 0 | 0.003008758 | 0 |
| 5817 | THMTHHAVSDHEATLR | 0 | 0.030048462 | 9.65268E-06 |
| 5818 | THVTHHPLSDHEATLR | 0 | 0.001027201 | 0 |
| 5819 | THVTHHPVSDHEATLR | 0 | 0.010502784 | 2.78043E-05 |
| 5820 | VAHHPISDHEATLR | 0 | 0.000425894 | 0 |
| 5821 | VTHHPLSDHEATLR | 0.000210378 | 0.032265207 | 0.000325596 |
| 5822 | VTHHPVSDHEATLR | 0 | 0.033475697 | 0 |
| 5823 | HPISDHEVTLRCWALG | 0 | 0.004482152 | 0 |
| 5824 | DHEATLRCWALG | 0 | 0.003705097 | 0 |
| 5825 | HPLSDHEATLRCWALG | 0 | 0.01647869 | 0 |
| 5826 | HPVSDHEATLRCWALG | 0 | 0.00499594 | 0 |
| 5827 | VSDHEATLRCWALG | 0 | 0.016673138 | 0.001470619 |
| 5828 | AEHPKTHVTHHLVSDHEATLR | 0 | 0.00010797 | 0 |
| 5829 | HHLVSDHEATLR | 0 | 0.002359223 | 0.000132396 |
| 5830 | HLVSDHEATLR | 0 | 0.001383469 | 0 |
| 5831 | HPKTHVTHHLVSDHEATLR | 0 | 0.004792354 | 0 |
| 5832 | HVTHHLVSDHEATLR | 0 | 0.017816064 | 0 |
| 5833 | LVSDHEATL | 0 | 0.000669366 | 0 |
| 5834 | LVSDHEATLR | 8.83131E-05 | 0.004585503 | 0.001168137 |
| 5835 | THHLVSDHEATLR | 0 | 0.022480064 | 2.04167E-05 |
| 5836 | THVTHHLVSDHEATLR | 0 | 0.006092095 | 0 |
| 5837 | VTHHLVSDHEATLR | 0 | 0.09680185 | 0 |
| 5838 | HPKTHVTHHLVSDHEATL | 0 | 0.001491006 | 0 |
| 5839 | HVTHHLVSDHEATL | 0 | 0.00668628 | 0 |
| 5840 | THHLVSDHEATL | 0 | 0.007218141 | 0 |
| 5841 | THVTHHLVSDHEATL | 0 | 0.002142843 | 0.000753329 |
| 5842 | VTHHLVSDHEATL | 2.16169E-05 | 0.025508282 | 0 |
| 5843 | EATLRCWALSFYPA | 0 | 0 | 0.001904068 |
| 5844 | DADIGVAEAERD | 0 | 3.67733E-05 | 0.001027077 |
| 5845 | DADIGVAEAERDAGIR | 0.000151809 | 0.000149933 | 0.009125443 |
| 5846 | DIGVAEAERD | 0 | 0 | 0.000165007 |
| 5847 | RDADIGVAEAERDAGIR | 8.53239E-05 | 8.12684E-05 | 0.007175982 |
| 5848 | DIGVAEAERDAGIR | 0 | 8.51491E-05 | 0.004747256 |
| 5849 | IPDPEAVKPDDWD | 0.001455046 | 0 | 3.99311E-05 |
| 5850 | RPKIPDPEAVKPDDWD | 0.001580582 | 0.000603823 | 0 |
| 5851 | RPKIPDPEAVKPDDWDED | 0.001282365 | 0 | 0 |
| 5852 | LAQPESIRSDLE | 3.55612E-05 | 0.002096368 | 6.64106E-05 |
| 5853 | LAQPESIRSDLESSD | 0 | 0.000925184 | 0 |
| 5854 | KPWDISLAQPESIRSD | 0 | 0 | 0.00419081 |
| 5855 | KPGSDTIKPDVQ | 0 | 0.001556127 | 0 |
| 5856 | DNSSLTGESEPQTRSPD | 0.000579268 | 0 | 0 |
| 5857 | NSSLTGESEPQTR | 0.000284895 | 1.02526E-05 | 0 |
| 5858 | NSSLTGESEPQTRSPD | 0.001092614 | 0 | 0 |
| 5859 | VDNSSLTGESEPQTRSPD | 0.00394165 | 0 | 0 |
| 5860 | VDNSSLTGESEPQTRSPDF | 0.001853326 | 0 | 0 |
| 5861 | SSLTGESEPQTRSPD | 0.001336318 | 0 | 0 |
| 5862 | VDNSSLTGESEPQTR | 0.002184379 | 1.10352E-05 | 0 |
| 5863 | GGHSSVSTESESSS | 0 | 0.000604312 | 0 |
| 5864 | GGHSSVSTESESSSF | 0 | 0.002729699 | 0 |
| 5865 | GGHSSVSTESESSSFH | 0 | 0.036332632 | 0 |
| 5866 | GGHSSVSTESESSSFHS | 1.16272E-05 | 0.00100012 | 0 |
| 5867 | RGGHSSVSTESESSSFHS | 0 | 7.94864E-05 | 0 |
| 5868 | GHSSVSTESESSSF | 0 | 0.005758855 | 0 |
| 5869 | GHSSVSTESESSSFH | 0 | 0.016322663 | 0 |
| 5870 | GKRGGHSSVSTESESSSFH | 0 | 0.003528898 | 0 |
| 5871 | HSSVSTESESSSF | 6.99714E-06 | 0.008627302 | 0 |
| 5872 | KGKRGGHSSVSTESESSSFH | 0 | 0.000393422 | 0 |
| 5873 | KRGGHSSVSTESESSSFH | 0 | 0.002716156 | 2.86038E-05 |
| 5874 | RGGHSSVSTESESSSFH | 2.88722E-06 | 0.005546511 | 0 |
| 5875 | HSSVSTESESSSFH | 0 | 0.006386262 | 0 |
| 5876 | SSVSTESESSSFH | 0 | 0.000277848 | 0 |
| 5877 | LSGDTQTDATSFYDR | 0 | 0.001731159 | 0 |
| 5878 | LSGDTQTDATSFYDRV | 0 | 0.000952367 | 0 |
| 5879 | SGDTQTDATSFYDRV | 0 | 0.000939759 | 0 |
| 5880 | VPVMVPAQSQAGS | 0 | 0 | 0.001998896 |
| 5881 | VPVMVPAQSQAGSLV | 0 | 0 | 0.000544249 |
| 5882 | LPVQLQRAMAAEAEAS | 0 | 0 | 0.00293107 |
| 5883 | LPVQLQRAMAAEAEASRE | 0 | 0 | 0.00371317 |
| 5884 | LPVQLQRAMAAEAEASREA | 0 | 0 | 0.001342513 |
| 5885 | LPVQLQRAMAAEAEASREAR | 0 | 0 | 0.001867686 |
| 5886 | LQRAMAAEAEASR | 0 | 0 | 0.000432621 |
| 5887 | LQRAMAAEAEASRE | 1.54313E-06 | 0 | 0.001923028 |
| 5888 | LQRAMAAEAEASREA | 0 | 1.08846E-05 | 0.001656045 |
| 5889 | EAESETDKQAALAG | 0 | 0.002881359 | 0 |
| 5890 | EAESETDKQAALAGN | 0 | 0.002557372 | 0 |
| 5891 | NEAESETDKQAALAGN | 0 | 0.000848825 | 0 |
| 5892 | EYSETEKEAPWQ | 0 | 0.001538898 | 0 |
| 5893 | IEDFVQKGSAQSVK | 4.14135E-05 | 0 | 0.005430193 |
| 5894 | IEDFVQKGSAQSVKK | 0 | 0 | 0.003978144 |
| 5895 | IEDFVQKGSAQSVKKVK | 0 | 0 | 0.001033528 |
| 5896 | AGIIASARAGETR | 0 | 0 | 0.001185122 |
| 5897 | AGIIASARAGETRFT | 0 | 1.14331E-05 | 0.001528102 |
| 5898 | IIASARAGETR | 0 | 1.51914E-06 | 0.00381797 |
| 5899 | KAGIIASARAGETR | 0 | 4.44553E-06 | 0.001941922 |
| 5900 | VPTTHPGQPARHP | 0 | 0 | 0.007572782 |
| 5901 | VPTTHPGQPARHPA | 0 | 0 | 0.002024955 |
| 5902 | APKAGLPGATGP | 0 | 0.000206629 | 0.008649481 |
| 5903 | SAPKAGLPGATGP | 1.42542E-05 | 0 | 0.017903635 |
| 5904 | SAPKAGLPGATGPM | 0 | 0 | 0.005713801 |
| 5905 | SPNSAPKAGLPGATGP | 0 | 2.59527E-05 | 0.009410766 |
| 5906 | GPSHGGLPGASSEK | 1.63482E-06 | 2.52945E-05 | 0.000237939 |
| 5907 | EASPRSAMSTATVTVN | 0 | 0.000187721 | 0.001333764 |
| 5908 | LPPLIPGGHATVSGPG | 0 | 0 | 0.003564892 |
| 5909 | KGLFRAAVPSGAST | 0 | 0 | 0.000476623 |
| 5910 | KGLFRAAVPSGASTG | 0 | 7.39898E-05 | 0.003508634 |
| 5911 | SKGLFRAAVPSGASTG | 0 | 0 | 0.010963684 |
| 5912 | TSKGLFRAAVPSGASTG | 0 | 0.000315465 | 0.012129164 |
| 5913 | EPFASGKTSESGEL | 0 | 0 | 0.006741374 |
| 5914 | EPFASGKTSESGELH | 0 | 0 | 0 |
| 5915 | EPFASGKTSESGELHG | 0 | 0 | 0.002338033 |
| 5916 | TWEPFASGKTSESGELHG | 0 | 0 | 0.001741994 |
| 5917 | WEPFASGKTSESGELHG | 0 | 0 | 0.007037308 |
| 5918 | WEPFASGKTSESGELH | 0 | 0 | 0.004698931 |
| 5919 | TWEPFASGKTSESGEL | 0 | 0 | 0.00575599 |
| 5920 | WEPFASGKTSESGEL | 0 | 0 | 0.038003915 |
| 5921 | AHRIVAPGKGILAADE | 0 | 0 | 0.001630315 |
| 5922 | DPVVIVSAARTIIGSFN | 0 | 0 | 0.000549625 |
| 5923 | VVIIGAGKPAA | 0 | 0 | 0.002823922 |
| 5924 | VVIIGAGKPAAVV | 0 | 0 | 0.001129846 |
| 5925 | VVIIGAGKPAAVVLQ | 0 | 0 | 0.002292625 |
| 5926 | VGGTRSSAVCAFSLLD | 0 | 0.000543956 | 0.001991741 |
| 5927 | DLSRGLTSARAAEIL | 0 | 0 | 0.000940806 |
| 5928 | DLSRGLTSARAAEILAR | 0 | 0 | 0.000805408 |
| 5929 | DLSRGLTSARAAEILARD | 0 | 0 | 0.000217993 |
| 5930 | LSRGLTSARAAEIL | 0 | 0.000223918 | 0.001950719 |
| 5931 | SRGLTSARAAEIL | 0 | 0.00010291 | 0.00295952 |
| 5932 | LSRGLTSARAAEILAR | 0 | 8.70917E-05 | 0.002340864 |
| 5933 | LSRGLTSARAAEILARD | 0 | 0 | 0.000924249 |
| 5934 | RGLTSARAAEILAR | 0 | 0 | 0.001329756 |
| 5935 | SRGLTSARAAEILAR | 0 | 0 | 0.013449373 |
| 5936 | SRGLTSARAAEILA | 0 | 0.000138412 | 0.00392061 |
| 5937 | LEFEARTLSPGHTWEE | 0 | 0.000747765 | 0 |
| 5938 | DPPRAAVPDAVG | 0 | 0 | 0.000796266 |
| 5939 | DPPRAAVPDAVGK | 0 | 0 | 0.007026321 |
| 5940 | PPRAAVPDAVGK | 0 | 3.23305E-05 | 0.000681329 |
| 5941 | NSGHSGLANGTVNDEPT | 0 | 7.53054E-05 | 0.001901371 |
| 5942 | ITYSYAGARAP | 3.1395E-05 | 0 | 0.020369399 |
| 5943 | ITYSYAGARAPMG | 0.0013156 | 5.87251E-05 | 0.017379176 |
| 5944 | ITYSYAGARAPMGQG | 0 | 2.66209E-05 | 0.005380386 |
| 5945 | YSYAGARAPMG | 0 | 0 | 0.001395136 |
| 5946 | YDYSYAGGRGSYGDLG | 0 | 0 | 0.000889316 |
| 5947 | LPFWAYSAAKSW | 0 | 0.000258147 | 0 |
| 5948 | LPFWAYSAAKSWV | 0.000624968 | 0.002456363 | 0.000990708 |
| 5949 | LPFWAYSAAKSWVFG | 0 | 0.004596326 | 0.004374629 |
| 5950 | LPFWAYSAAKSWVFGVH | 0.000123923 | 0.001888885 | 0.001919107 |
| 5951 | WAYSAAKSWVFG | 0 | 0.003303112 | 0.005061043 |
| 5952 | WAYSAAKSWVFGVH | 0 | 0.000458774 | 0.00072356 |
| 5953 | TPGSKAALANLCIGDV | 0.000681482 | 0 | 0.00644136 |
| 5954 | VTPGSKAALANLCIGDV | 0 | 0 | 0.017302283 |
| 5955 | VTPGSKAALANLCIGDVI | 0 | 0 | 0.002884454 |
| 5956 | VTPGSKAALANLCIG | 0 | 0 | 0.0045438 |
| 5957 | GKGSITISAEEIK | 0 | 0.000182475 | 0 |
| 5958 | RPAGKGSITISAEEIK | 0 | 0.003763208 | 0 |
| 5959 | SAKASVSVTAEDEGTQ | 0 | 0.000907503 | 0 |
| 5960 | APKGSVLQEAK | 7.97315E-06 | 0 | 0.000210499 |
| 5961 | SAPKGSVLQEAK | 0 | 0 | 0.055077337 |
| 5962 | SAPKGSVLQEAKL | 0 | 0 | 0.019231365 |
| 5963 | TSAPKGSVLQEAKL | 0 | 0 | 0.004091089 |
| 5964 | TSAPKGSVLQEAK | 0 | 0 | 0.038599221 |
| 5965 | FLRYAVVL | 0.000858395 | 0 | 0 |
| 5966 | LPENVKYGIVLDAGSS | 0 | 0 | 0.024378717 |
| 5967 | GIVLDAGSSHTSL | 0 | 0 | 0.001970163 |
| 5968 | IVLDAGSSHTSL | 0 | 0 | 0.001118232 |
| 5969 | AQHGPHSAVARISR | 0 | 0 | 0.001231366 |
| 5970 | GPHSAVARISR | 0 | 0 | 0.004479775 |
| 5971 | HGPHSAVARISR | 0 | 0 | 0.000440627 |
| 5972 | QHGPHSAVARISR | 8.71834E-06 | 0 | 0.001157506 |
| 5973 | GPHSAVARISRAQ | 0 | 0 | 0.000393411 |
| 5974 | GPHSAVARISRAQYN | 0 | 0 | 0.000454228 |
| 5975 | QYDTPKAGKPGLPAPD | 0 | 0 | 0.000983981 |
| 5976 | VPQSPKAGKPS | 0 | 0 | 0.000320152 |
| 5977 | LVVWEAGKAG | 0.001031333 | 0.0003771 | 0.002118286 |
| 5978 | LVVWEAGKAGLEE | 0 | 0 | 0.007446684 |
| 5979 | VVMFQTAVGHSFK | 0 | 0.002092351 | 0 |
| 5980 | DSATDTIDIAPNHR | 0 | 0.000996772 | 0 |
| 5981 | HDSATDTIDIAPNHR | 5.67818E-06 | 0.001035615 | 2.0198E-05 |
| 5982 | RHDSATDTIDIAPNHR | 7.95382E-05 | 0.001329335 | 0 |
| 5983 | VRHDSATDTIDIAPNHR | 0 | 0.000947245 | 3.95246E-05 |
| 5984 | VRHDSATDTIDIAPN | 0 | 0.000572722 | 0 |
| 5985 | RPAPNSVDVDDFIN | 0 | 0.00056884 | 0 |
| 5986 | GPPRTIYENRIY | 0 | 0.000350383 | 2.88421E-05 |
| 5987 | IIGPPRTIYENRIY | 0 | 0.003943805 | 0 |
| 5988 | IIGPPRTIYENR | 0 | 0.001049246 | 0.000778115 |
| 5989 | DGTGGKSIYGERFPDE | 0 | 0.025113313 | 0 |
| 5990 | DGTGGKSIYGERFPDEN | 0 | 0.019875451 | 0 |
| 5991 | GDGTGGKSIYGERFPDEN | 0 | 0.019897778 | 0 |
| 5992 | GGDFTRGDGTGGKSIYGERFPDEN | 0 | 0.000888535 | 0 |
| 5993 | GGKSIYGERFPDE | 0 | 0.000188409 | 0 |
| 5994 | GTGGKSIYGERFPDE | 0 | 0.019884677 | 0 |
| 5995 | RGDGTGGKSIYGERFPDEN | 0.000438153 | 0.004063696 | 0 |
| 5996 | GDGTGGKSIYGERFPDE | 4.67114E-05 | 0.012868989 | 0 |
| 5997 | GTGGKSIYGERFPD | 0 | 0.000935147 | 0 |
| 5998 | GGKSIYGERFPDEN | 0.00015788 | 0.000963555 | 0.000275989 |
| 5999 | GTGGKSIYGERFPDEN | 0 | 0.01546882 | 0.000164349 |
| 6000 | GGKSIYGEKFEDEN | 0.000659253 | 0.001585943 | 0 |
| 6001 | GGKSIYGEKFEDENFI | 0.00027106 | 0.003967486 | 0 |
| 6002 | GTGGKSIYGEKFEDE | 0 | 0.000362548 | 0 |
| 6003 | GTGGKSIYGEKFEDEN | 0.000452851 | 0.003883777 | 0 |
| 6004 | RHVFWGSGSHTL | 8.84096E-06 | 0.003357789 | 0 |
| 6005 | SPFRHVFWGSGSHTL | 0 | 0.017825132 | 0 |
| 6006 | SPFRHVFWGSGSHTLP | 0 | 0.00464488 | 0 |
| 6007 | SPFRHVFWGSGSHTLPA | 0 | 0.007654163 | 0 |
| 6008 | SPFRHVFWGSGSHTLPAL | 0 | 0.013251336 | 0 |
| 6009 | APGEPTSRSQGEDCVMMRG | 0.000760748 | 0.000183163 | 0 |
| 6010 | DESTLIEDGGARSD | 0 | 0 | 0.002095373 |
| 6011 | DESTLIEDGGARSDG | 0 | 1.58349E-05 | 0.000776452 |
| 6012 | DESTLIEDGGARSDGSL | 0 | 3.67476E-05 | 0.037787351 |
| 6013 | DESTLIEDGGARSDGSLE | 0.000128085 | 0 | 0.00329564 |
| 6014 | SDESTLIEDGGARSDGSLE | 0 | 0 | 0.002088415 |
| 6015 | STLIEDGGARSD | 0 | 0 | 0.000861019 |
| 6016 | ESTLIEDGGARSDGSL | 0 | 6.467E-05 | 0.010827467 |
| 6017 | IEDGGARSDGSL | 0 | 0 | 0.000712302 |
| 6018 | SDESTLIEDGGARSDGSL | 0 | 0 | 0.004303855 |
| 6019 | STLIEDGGARSDGSL | 0 | 0 | 0.023136312 |
| 6020 | EGNLAAAHANTGPEE | 0 | 0 | 0.001236388 |
| 6021 | GEGNLAAAHANTGPEE | 0 | 4.51293E-06 | 0.00067908 |
| 6022 | GPARETELPAAAEPGAE | 0 | 0 | 0.005664292 |
| 6023 | GPARETELPAAAEPGAEN | 0 | 0 | 0.011132505 |
| 6024 | TPTQPASEKEPEMPGPR | 0.000334312 | 0.001499998 | 0 |
| 6025 | DEYLKTTGKPIE | 0 | 0 | 0 |
| 6026 | SEAGSHTLQW | 0 | 0.005561836 | 0 |
| 6027 | VDPWTVQTSSAKGIDYD | 0 | 0.002773767 | 0 |
| 6028 | GPGAHRGEPAPED | 0.007435267 | 9.95803E-06 | 0.028382948 |
| 6029 | GPGAHRGEPAPEDP | 0.001689116 | 0 | 0.003159855 |
| 6030 | GPGAHRGEPAPEDPP | 0.193859465 | 0 | 0.368830456 |
| 6031 | GPGAHRGEPAPEDPPK | 0.00060468 | 0 | 0.008706772 |
| 6032 | IYYFFREDNPDKNPEAPL | 0.004500906 | 0 | 0 |
| 6033 | NTDHQTQLLY | 0.00991919 | 0.002593948 | 0 |
| 6034 | EEDSSGHTRAY | 0 | 0.000316653 | 0 |
| 6035 | AAAEVAGQFVIKLTHD | 0.003539568 | 0 | 6.1842E-05 |
| 6036 | ACLPNLDHVGHPTERCFVF | 0 | 0.001222965 | 0.002827514 |
| 6037 | ELNKVARAAAEVAG | 0 | 0 | 0.00111948 |
| 6038 | IERIPELNKVARAAAEVAGQFVIK | 0 | 0 | 0.000455347 |
| 6039 | IPELNKVARAAAEVAG | 0.011568352 | 0 | 0.649806767 |
| 6040 | IPELNKVARAAAEVAGQ | 0.000585846 | 0 | 0.172300217 |
| 6041 | IPELNKVARAAAEVAGQF | 0.027798423 | 0 | 1.266123661 |
| 6042 | IPELNKVARAAAEVAGQFV | 0.000497251 | 0 | 0.107980025 |
| 6043 | IPELNKVARAAAEVAGQFVI | 0 | 0 | 0.020957384 |
| 6044 | IPELNKVARAAAEVAGQFVIK | 0 | 0 | 0.023648859 |
| 6045 | KVARAAAEVAG | 0 | 0 | 0.022765637 |
| 6046 | LNKVARAAAEVAG | 0.000362509 | 0 | 0.022706641 |
| 6047 | NKVARAAAEVAG | 0 | 0 | 0.000682216 |
| 6048 | RIPELNKVARAAAEVAGQFVIK | 0 | 0 | 0.000984708 |
| 6049 | LNKVARAAAEVA | 0 | 0 | 0.00096978 |
| 6050 | LNKVARAAAEVAGQ | 7.98224E-06 | 0 | 0.007566273 |
| 6051 | LNKVARAAAEVAGQF | 0.004764192 | 5.78132E-05 | 0.050260649 |
| 6052 | IPELNKVARAAAE | 0 | 0 | 0.005518546 |
| 6053 | IPELNKVARAAAEV | 0 | 0 | 0.007416945 |
| 6054 | IPELNKVARAAAEVA | 0.008117156 | 0 | 0.130695167 |
| 6055 | RIPELNKVARAAAEVA | 0 | 0 | 0.002182317 |
| 6056 | RIPELNKVARAAAEVAG | 0 | 0 | 0.003055971 |
| 6057 | KVARAAAEVAGQ | 0 | 0 | 0.005772131 |
| 6058 | KVARAAAEVAGQF | 0.001386427 | 0 | 0.119819696 |
| 6059 | KVARAAAEVAGQFV | 0.000424329 | 4.48897E-05 | 0.018440917 |
| 6060 | KVARAAAEVAGQFVIK | 4.25863E-05 | 0 | 0.001486672 |
| 6061 | QFVIKLTHDVEL | 0 | 0.000709208 | 5.59226E-05 |
| 6062 | QFVIKLTHDVELN | 0 | 0.000766331 | 0.000706697 |
| 6063 | DYHDYSSTDVSPEESPS | 0 | 0.000527787 | 0 |
| 6064 | YHDYSSTDVSPEESPS | 0 | 0.000431701 | 0 |
| 6065 | DRVGHSTAHDEIIP | 3.01705E-05 | 0.012178532 | 0.000210101 |
| 6066 | RVGHSTAHDEIIP | 0 | 0.003378683 | 0.000156748 |
| 6067 | AEVDKVTGRF | 0 | 0.0040085 | 0 |
| 6068 | APGVLAENPDIF | 0.008386963 | 4.26407E-05 | 0 |
| 6069 | NAPGVLAENPDIF | 0.003617253 | 0 | 0 |
| 6070 | ETTLVANQLR | 0 | 0.001822898 | 0 |
| 6071 | ITRLQETTLVANQLR | 0 | 0.008671661 | 0 |
| 6072 | RLQETTLVANQLR | 0 | 0.002000914 | 0 |
| 6073 | TRLQETTLVANQLR | 0 | 0.004234941 | 6.21963E-05 |
| 6074 | IGRSIATLMTDEIFHD | 0.000501358 | 0.004184954 | 0 |
| 6075 | KEGTLITDHPNIQ | 0 | 0.001069468 | 0 |
| 6076 | NKEGTLITDHPNIQ | 0 | 0.002294897 | 0.004097054 |
| 6077 | DEVDTDQSGVLSDR | 0 | 0.001874607 | 5.28004E-05 |
| 6078 | DEVDTDQSGVLSDRE | 0 | 0.002805224 | 0 |
| 6079 | EVDTDQSGVLSDR | 0 | 2.72997E-05 | 0 |
| 6080 | VFDEVDTDQSGVLSDRE | 0 | 0.009147715 | 0 |
| 6081 | VFDEVDTDQSGVLSDR | 0 | 0.008230483 | 0 |
| 6082 | KNINADEAAAMG | 0.000895103 | 7.98933E-05 | 0 |
| 6083 | LGKNINADEAAAMG | 4.44824E-05 | 0.000823649 | 0 |
| 6084 | KPQSLDTDDPATL | 0 | 0.000457505 | 0 |
| 6085 | KPQSLDTDDPATLY | 0 | 0.000516829 | 0 |
| 6086 | KPQSLDTDDPATLYA | 0.00591704 | 0.001403594 | 6.00536E-05 |
| 6087 | KPQSLDTDDPATLYAV | 0.001729372 | 0.000285219 | 0 |
| 6088 | EPLSYTRFSLARQ | 0 | 0.000143633 | 0.003060028 |
| 6089 | EPLSYTRFSLARQVD | 4.81932E-05 | 0.000110796 | 0.043267697 |
| 6090 | EPLSYTRFSLARQVDG | 8.75005E-05 | 0.000169735 | 0.033544672 |
| 6091 | EPLSYTRFSLARQVDGD | 0 | 0.000280923 | 0.018000979 |
| 6092 | EPLSYTRFSLARQVDGDN | 0 | 0 | 0.124849902 |
| 6093 | GEPLSYTRFSLARQVDGDN | 0 | 0 | 0.002538957 |
| 6094 | SYTRFSLARQVDGDN | 0 | 0 | 0.001428545 |
| 6095 | GEPLSYTRFSLAR | 0.000128944 | 0.00031044 | 0.000756074 |
| 6096 | GEPLSYTRFSLARQ | 0.000216255 | 0.00144134 | 0.014093915 |
| 6097 | GEPLSYTRFSLARQV | 0 | 0.000304084 | 0.002872512 |
| 6098 | GEPLSYTRFSLARQVD | 0.000841894 | 0.005033135 | 0.049828754 |
| 6099 | GEPLSYTRFSLARQVDG | 0 | 0 | 0.011763076 |
| 6100 | GGEPLSYTRFSLARQVDG | 0 | 6.70347E-05 | 0.000428174 |
| 6101 | GGEPLSYTRFSLARQ | 0 | 0.0001328 | 0.003434544 |
| 6102 | GGEPLSYTRFSLARQVD | 0 | 0.000998849 | 0.020155083 |
| 6103 | DDIEEAGIVAREFG | 0 | 0 | 0 |
| 6104 | DIEEAGIVAREFG | 0.000424636 | 0 | 0.005334198 |
| 6105 | IDGWPSDDIEEAGIVAREFG | 0 | 0 | 0.000805508 |
| 6106 | WPSDDIEEAGIVAREFG | 0.001423602 | 0 | 0.049413575 |
| 6107 | WPSDDIEEAGIVAREF | 0 | 0 | 0.003398445 |
| 6108 | WPSDDIEEAGIVAREFGVN | 0 | 0 | 0.002126006 |
| 6109 | LATDDPSLLK | 7.97091E-05 | 0.000419596 | 0 |
| 6110 | TSGENTAVVAVEPDRR | 0 | 0.000399637 | 0 |
| 6111 | QKENAGEDPGLA | 0.000874779 | 5.23243E-05 | 0.000100117 |
| 6112 | YDFSFKSSVITLNTN | 0 | 0 | 0.003283726 |
| 6113 | APYLKVDTIAAD | 0 | 0.000614576 | 0 |
| 6114 | APYLKVDTIAADE | 0 | 0.000747743 | 0.000109925 |
| 6115 | APYLKVDTIAADES | 0.004712354 | 0.004077037 | 0 |
| 6116 | APYLKVDTIAADESFS | 0 | 0.018999692 | 0 |
| 6117 | APYLKVDTIAADESFSQ | 0 | 0.012436777 | 0 |
| 6118 | DPVVNPVATEDTAGLPG | 0 | 0.00384337 | 0 |
| 6119 | VNPVATEDTAGLPG | 7.4903E-05 | 0.001262861 | 0 |
| 6120 | SSGVSTNDASPLDPI | 0 | 0.003851424 | 0 |
| 6121 | GKESQLNFDSISPEDAGSY | 0.001367112 | 0.002867909 | 0 |
| 6122 | SQLNFDSISPEDAG | 0.006275168 | 0.001158511 | 0 |
| 6123 | SQLNFDSISPEDAGSY | 0.014942801 | 0.026092599 | 0.001926101 |
| 6124 | SQLNFDSISPEDAGSYS | 7.71094E-05 | 0.002922434 | 0 |
| 6125 | ISRDNTPALEPQ | 0.000856335 | 0 | 3.74535E-06 |
| 6126 | LAGAVARENAGPA | 0 | 0 | 0.000107936 |
| 6127 | HPEDPASVVEARK | 0 | 0.001534117 | 0 |
| 6128 | SPDDPAGLLDLRQ | 0.001705569 | 0 | 0 |
| 6129 | EDVLPGKLPETP | 0 | 0 | 0.001975883 |
| 6130 | VDEDVLPGKLPETP | 0 | 0 | 0.006397172 |
| 6131 | GPLHQLAQSVLEGMAAG | 0 | 0.000295505 | 2.38925E-05 |
| 6132 | LAQSVLEGMAAGTF | 0 | 0 | 0.002179196 |
| 6133 | LAQSVLEGMAAGTFL | 0 | 0 | 0.006082107 |
| 6134 | LVETIAATAREIE | 0 | 0 | 0.000178175 |
| 6135 | LVETIAATAREIEEN | 0 | 0 | 0.002365116 |
| 6136 | KMKEIAEAY | 0 | 0 | 0.002120205 |
| 6137 | LPGYRVREITENLM | 0 | 0.001241867 | 2.98571E-05 |
| 6138 | LPLPGYRVREITENLM | 0.0022825 | 0.007936047 | 0 |
| 6139 | LPLPGYRVREITENLMATG | 0.000591681 | 0.002149568 | 0 |
| 6140 | HAGTYSCVAENIL | 9.92239E-05 | 0.001907631 | 0 |
| 6141 | LPWHAGTYSCVAENIL | 0.000634648 | 0.004720288 | 0.000465478 |
| 6142 | LPWHAGTYSCVAENILG | 0.000458181 | 0.005926556 | 0 |
| 6143 | LPWHAGTYSCVAENILGTG | 0 | 0.001358864 | 0 |
| 6144 | LPWHAGTYSCVAENI | 0.000215749 | 0.001161194 | 0 |
| 6145 | IHSMADQVLPPGWQ | 0 | 0.001000947 | 0 |
| 6146 | IYDEEAMVEAVAL | 0 | 0 | 0 |
| 6147 | IYDEEAMVEAVALYNPV | 0 | 0 | 0 |
| 6148 | VPSPKVSDTVVEPYN | 0 | 0.004759413 | 0.00015108 |
| 6149 | VPSPKVSDTVVEPYNAT | 0 | 0.000667524 | 0 |
| 6150 | AAFNSGKVDIVA | 0.001447297 | 0.001980906 | 0.000638568 |
| 6151 | AAFNSGKVDIVAIN | 0 | 0.000177276 | 0.003167283 |
| 6152 | RAAFNSGKVDIVAIN | 9.88235E-05 | 0.008398124 | 0.032464126 |
| 6153 | TRAAFNSGKVDIVAIN | 0 | 0.015123679 | 0.031506972 |
| 6154 | VTRAAFNSGKVDIVAIN | 0 | 0.016682043 | 0.02525738 |
| 6155 | RAAFNSGKVDIVAI | 0 | 0.00199915 | 0 |
| 6156 | LVTRAAFNSGKVDIVA | 0 | 0.002330493 | 0.001085769 |
| 6157 | RAAFNSGKVDIV | 0 | 0.002920075 | 0.000625134 |
| 6158 | RAAFNSGKVDIVA | 0 | 0.020764487 | 0.017469922 |
| 6159 | TRAAFNSGKVDIVA | 0 | 0.034702464 | 0.026241721 |
| 6160 | VTRAAFNSGKVDIVA | 0.002072578 | 0.046875159 | 0.020334456 |
| 6161 | TRAAFNSGKVDIV | 0 | 0.004648537 | 0.001549988 |
| 6162 | VTRAAFNSGKVDIV | 0 | 0.00886082 | 0.005867228 |
| 6163 | GKVDIVAINDPFID | 0.032553757 | 0 | 0 |
| 6164 | GKVDIVAINDPFIDL | 0.002383591 | 0 | 0.000127694 |
| 6165 | GKVDIVAINDPFIDLN | 0.002055099 | 0 | 0 |
| 6166 | KVDIVAINDPFID | 0.013441928 | 0 | 7.42865E-05 |
| 6167 | SGKVDIVAINDPFID | 0.013979726 | 0 | 0 |
| 6168 | EDIEIIPIQEEE | 0.003911733 | 0.000144025 | 0 |
| 6169 | EDIEIIPIQEEEE | 0.0078313 | 0.001311464 | 0 |
| 6170 | EDIEIIPIQEEEEE | 0.006798664 | 0.003590261 | 0 |
| 6171 | EDIEIIPIQEEEEEE | 0.002593957 | 0.000641108 | 0 |
| 6172 | EDIEIIPIQEEEEEET | 0.011534859 | 0.005104576 | 0 |
| 6173 | EDIEIIPIQEEEEEETE | 0.001932619 | 0.001743054 | 0 |
| 6174 | IEIIPIQEEE | 0.001699861 | 0 | 0 |
| 6175 | EEEEEETETNFPEPP | 0 | 0.042088625 | 0 |
| 6176 | EEEEEETETNFPEPPQ | 0 | 0.054693335 | 0 |
| 6177 | EEEEEETETNFPEPPQD | 0 | 0.001431739 | 0 |
| 6178 | EEEEEETETNFPEPPQDQ | 0 | 0.006205257 | 0 |
| 6179 | EEEEETETNFPEPP | 0 | 0.038729591 | 0 |
| 6180 | EEEETETNFPEPP | 0 | 0.006297148 | 0 |
| 6181 | EEETETNFPEPP | 0 | 0.007571651 | 4.1468E-05 |
| 6182 | EETETNFPEPP | 0 | 0.00727949 | 0 |
| 6183 | IPIQEEEEEETETNFPEPPQDQ | 0 | 0.004869757 | 0 |
| 6184 | EEEEETETNFPEPPQ | 0.003661056 | 0.036535033 | 0 |
| 6185 | EEEEETETNFPEPPQDQ | 0 | 0.003385895 | 0 |
| 6186 | EEEETETNFPEPPQ | 0 | 0.005358281 | 0 |
| 6187 | EEETETNFPEPPQ | 0 | 0.009611165 | 0 |
| 6188 | EETETNFPEPPQ | 0 | 0.008879655 | 0 |
| 6189 | EEEETETNFPEPPQDQ | 0 | 0.001081457 | 0 |
| 6190 | EEETETNFPEPPQDQ | 0 | 0.001762864 | 0 |
| 6191 | EETETNFPEPPQDQ | 0 | 0.000490847 | 0 |
| 6192 | IPIQEEEEEETE | 0.001422584 | 2.94646E-05 | 0 |
| 6193 | IPIQEEEEEETETNFPEPP | 0 | 0.076347386 | 0 |
| 6194 | IPIQEEEEEETETNFPEPPQ | 0 | 0.097370558 | 0 |
| 6195 | SKTSKYLTDSEYTEGS | 0 | 0.001146831 | 0 |
| 6196 | TSKYLTDSEYTEG | 0 | 0.000415976 | 0.000194391 |
| 6197 | YPSRQMVSLEDDVA | 0.00016227 | 0.00048302 | 0 |
| 6198 | AQPAGATVQEQLR | 0 | 0 | 0.000430067 |
| 6199 | GAQPAGATVQEQLR | 0 | 0 | 0.001409081 |
| 6200 | SPGAQPAGATVQEQLR | 0 | 0.000889189 | 0.024301978 |
| 6201 | SPGAQPAGATVQEQLRQ | 0 | 8.97978E-05 | 0.009487381 |
| 6202 | SPATIENELIK | 0 | 0.002185613 | 0 |
| 6203 | AYDGKDYLALNEDLR | 0.041599919 | 0.000334339 | 0.000264956 |
| 6204 | AYDGKDYLALNEDLRSW | 0.0108502 | 0 | 0 |
| 6205 | AYDGKDYLALNEDLRSWT | 0.011103832 | 0 | 0 |
| 6206 | DAYDGKDYIALNEDLRSWT | 0.002109863 | 0 | 0 |
| 6207 | DGKDYLALNED | 0.005048283 | 0 | 0 |
| 6208 | DGKDYLALNEDLR | 0.444899094 | 0 | 0 |
| 6209 | DGKDYLTLNEDLR | 0 | 0.012035384 | 0 |
| 6210 | YDGKDYLALNEDLR | 0.192652319 | 0 | 0.000151804 |
| 6211 | YDGKDYLTLNEDLR | 0 | 0.000881391 | 0 |
| 6212 | YDGKDYLALNEDLRS | 0.012411529 | 0.002557998 | 0 |
| 6213 | YDGKDYLALNEDLRSW | 0.044774931 | 0 | 0.000132959 |
| 6214 | YDGKDYLALNEDLRSWT | 0.042317925 | 0 | 0 |
| 6215 | YDGKDYLTLNEDLRSW | 0 | 0.000794462 | 0 |
| 6216 | DQSAYDGKDYIALNEDLR | 0.005791606 | 0 | 0 |
| 6217 | DQSAYDGKDYIALNEDLRSWT | 0.001367525 | 0 | 0 |
| 6218 | YDGKDYLALNED | 0.006694585 | 0 | 6.5148E-05 |
| 6219 | YDGKDYLALNEDL | 0 | 0 | 0 |
| 6220 | DAYDGKDYIALNEDLR | 0.006441368 | 0 | 0 |
| 6221 | DYLALNEDLR | 0.009753819 | 2.96365E-05 | 0 |
| 6222 | GKDYLALNEDLR | 0.090806272 | 3.028E-05 | 7.71062E-05 |
| 6223 | KDYLALNEDLR | 0.033901949 | 2.3128E-05 | 4.10456E-05 |
| 6224 | LALNEDLR | 0.000761216 | 0 | 0.000104897 |
| 6225 | QDAYDGKDYIALNEDLR | 0.005889914 | 0 | 0 |
| 6226 | RQDAYDGKDYIALNEDLR | 0.002044168 | 0.000198365 | 0 |
| 6227 | DGKDYISLNEDLRSW | 0.000219721 | 0.000458384 | 0 |
| 6228 | DGKDYLALNEDLRS | 0.043275296 | 0.002896747 | 0 |
| 6229 | DGKDYLALNEDLRSW | 0.17440749 | 0.000355758 | 0.00017759 |
| 6230 | DGKDYLALNEDLRSWT | 0.099476073 | 0 | 0.000666535 |
| 6231 | DGKDYLALNEDLRSWTA | 0.012930191 | 0 | 0.001432363 |
| 6232 | DGKDYLALNEDLRSWTAA | 0.000448887 | 0.000768368 | 0.000807758 |
| 6233 | DGKDYLTLNEDLRS | 0.000934904 | 0.000843753 | 0 |
| 6234 | DGKDYLTLNEDLRSW | 0 | 0.016978086 | 0 |
| 6235 | DYLALNEDLRS | 0.000320539 | 0 | 0 |
| 6236 | GKDYLALNEDLRS | 0.011588277 | 0.000921 | 0 |
| 6237 | KDYLALNEDLRS | 0.004561574 | 0.002358939 | 0.000860419 |
| 6238 | DYLALNEDLRSW | 0.001360642 | 0.000120505 | 0 |
| 6239 | GKDYLALNEDLRSW | 0.02876373 | 0.000406865 | 0 |
| 6240 | GKDYLALNEDLRSWT | 0.027466834 | 0 | 3.6619E-05 |
| 6241 | GKDYLALNEDLRSWTA | 0.005738971 | 9.39296E-05 | 0.000704878 |
| 6242 | GKDYLTLNEDLRSW | 0 | 0.001635343 | 0 |
| 6243 | KDYLALNEDLRSW | 0.008837795 | 0 | 0 |
| 6244 | KDYLALNEDLRSWT | 0.004863942 | 0.00023873 | 0.00121203 |
| 6245 | KDYLALNEDLRSWTA | 3.24271E-05 | 0 | 0.001088188 |
| 6246 | DGKDYIALNEDLSSWT | 0 | 0.001203732 | 0 |
| 6247 | DLRSWTAADMAAQ | 0.000359087 | 0 | 0.001253294 |
| 6248 | DLRSWTAADMAAQIT | 0.007469275 | 0 | 0.011253772 |
| 6249 | DLRSWTAADMAAQITK | 0.011336266 | 0.000253156 | 0.012087339 |
| 6250 | DLRSWTAADMAAQITKR | 0.043980901 | 0 | 0.003314757 |
| 6251 | DLRSWTAADMAAQITKRK | 0.001965892 | 0 | 0 |
| 6252 | LRSWTAADMAAQITKR | 0.015279205 | 0 | 0.000550386 |
| 6253 | LRSWTAADMAAQITKRK | 0.002335482 | 0 | 6.18792E-05 |
| 6254 | RSWTAADMAAQITK | 0.001079883 | 5.99349E-05 | 0.000145666 |
| 6255 | RSWTAADMAAQITKR | 0.007395602 | 0.002204918 | 0 |
| 6256 | EDLRSWTAADMAAQ | 0.001735801 | 0.000145748 | 0.000569816 |
| 6257 | EDLRSWTAADMAAQIT | 0.008814841 | 0.000310778 | 0.005518336 |
| 6258 | EDLRSWTAADMAAQITK | 0.002422005 | 0 | 0.018817468 |
| 6259 | EDLRSWTAADMAAQITKR | 0.001285196 | 0 | 0.002354101 |
| 6260 | NEDLRSWTAADMAAQIT | 0.003257741 | 0 | 0 |
| 6261 | DLRSWTAADMAAQITQ | 0 | 0 | 0 |
| 6262 | DLRSWTAADMAAQITQR | 0 | 0.000813011 | 0 |
| 6263 | RSWTAADMAAQIT | 0.011029298 | 0.000116963 | 0 |
| 6264 | RSWTAADMAAQITQ | 0 | 0.001126956 | 0 |
| 6265 | EDLRSWTAADMAAQITQ | 0 | 0.000239221 | 0.003279004 |
| 6266 | LRSWTAADMAAQITQR | 0.002457811 | 0.001725541 | 0.000190847 |
| 6267 | RSWTAADMAAQITQR | 0 | 0.003618229 | 0 |
| 6268 | DLRSWTAADMAAQT | 0 | 0 | 0.000814927 |
| 6269 | DLRSWTAADMAAQTT | 0 | 0 | 0.000877035 |
| 6270 | DLRSWTAADMAAQTTK | 0.001146506 | 0.000115341 | 0.002056663 |
| 6271 | DLRSWTAADMAAQTTKH | 0 | 0 | 0.001039657 |
| 6272 | EDLRSWTAADMAAQTTKH | 0 | 0 | 0.000869745 |
| 6273 | EDLRSWTAADMAAQTTK | 0 | 0 | 0.002579876 |
| 6274 | LNEDLRSWTAADM | 0 | 0.000251313 | 5.03116E-05 |
| 6275 | DLRSWTAADTAAQ | 0.005728463 | 0 | 0.020360653 |
| 6276 | DLRSWTAADTAAQI | 0.000337295 | 0 | 0.001152609 |
| 6277 | DLRSWTAADTAAQIT | 0.087667976 | 0 | 0.200225069 |
| 6278 | DLRSWTAADTAAQITQ | 0.108672909 | 0 | 0.019293921 |
| 6279 | DLRSWTAADTAAQITQR | 0.169535623 | 0 | 0.0079272 |
| 6280 | DLRSWTAADTAAQITQRK | 0.017151486 | 0 | 3.53642E-05 |
| 6281 | EDLRSWTAADTAAQITQRK | 0.00776667 | 0 | 5.20128E-05 |
| 6282 | EDLRSWTAADTAAQITQRKWE | 0.00259872 | 0 | 0.002009674 |
| 6283 | LRSWTAADTAAQ | 0.000325558 | 0 | 0.001288884 |
| 6284 | EDLRSWTAADTAAQ | 0.0182982 | 0.000435036 | 0.009077604 |
| 6285 | EDLRSWTAADTAAQI | 0.001653412 | 0 | 0.000438845 |
| 6286 | EDLRSWTAADTAAQIT | 0.054711689 | 1.94529E-05 | 0.050842276 |
| 6287 | EDLRSWTAADTAAQITQ | 0.056053313 | 0 | 0.030663955 |
| 6288 | EDLRSWTAADTAAQITQR | 0.053554432 | 1.32866E-05 | 0.010321865 |
| 6289 | NEDLRSWTAADTAAQITQR | 0 | 0 | 0 |
| 6290 | LRSWTAADTAAQITQ | 0.069558515 | 8.21303E-05 | 0.001142094 |
| 6291 | LRSWTAADTAAQITQR | 0.049425499 | 0.000548835 | 0 |
| 6292 | LRSWTAADTAAQITQRK | 0.002665149 | 0 | 0 |
| 6293 | RSWTAADTAAQIT | 0.061347434 | 0 | 0.008185572 |
| 6294 | RSWTAADTAAQITQ | 0.031526079 | 0.002185568 | 0.000387057 |
| 6295 | LNEDLRSWTAADTAAQIT | 0.008659914 | 0 | 0.001891079 |
| 6296 | LNEDLRSWTAADTAAQITQ | 0.005508637 | 0 | 0.001036768 |
| 6297 | LRSWTAADTAAQI | 0.00026161 | 0.000375167 | 0.001355498 |
| 6298 | LRSWTAADTAAQIT | 0.046601633 | 0 | 0.018953545 |
| 6299 | NEDLRSWTAADTAAQIT | 0.022411488 | 0.000201183 | 0 |
| 6300 | NEDLRSWTAADTAAQ | 0.006071393 | 0.000167093 | 0.001519414 |
| 6301 | NEDLRSWTAADTAAQITQ | 0.003852128 | 0 | 0.000428312 |
| 6302 | DLSSWTAADTAAQ | 0 | 0 | 0.005683764 |
| 6303 | DLSSWTAADTAAQIT | 0 | 0 | 0.091756001 |
| 6304 | DLSSWTAADTAAQITQ | 0 | 0.000324686 | 0.001571885 |
| 6305 | DLSSWTAADTAAQITQR | 0 | 0 | 0.01041806 |
| 6306 | DLSSWTAADTAAQITQRK | 0 | 9.61534E-05 | 0.000971863 |
| 6307 | EDLSSWTAADTAAQITQRKW | 0 | 0 | 0.003708667 |
| 6308 | EDLSSWTAADTAAQITQRKWE | 0 | 0.000183934 | 0.00959865 |
| 6309 | SSWTAADTAAQITQR | 0 | 0.002621707 | 0.00044822 |
| 6310 | EDLSSWTAADTAAQ | 0 | 0 | 0.002374184 |
| 6311 | EDLSSWTAADTAAQIT | 0 | 0 | 0.040710142 |
| 6312 | EDLSSWTAADTAAQITQ | 0 | 0 | 0.020925355 |
| 6313 | EDLSSWTAADTAAQITQR | 0 | 0 | 0.026715034 |
| 6314 | LNEDLSSWTAADTAAQITQ | 0 | 0 | 0.000978485 |
| 6315 | LSSWTAADTAAQIT | 0 | 0 | 0.043056509 |
| 6316 | SSWTAADTAAQIT | 0 | 0 | 0.013514745 |
| 6317 | RSWTAADTAAQITQR | 0.017359126 | 0.002202473 | 0.000194698 |
| 6318 | RSWTAADTAAQITQRK | 0.000780671 | 0 | 0 |
| 6319 | SWTAADTAAQITQR | 7.57109E-05 | 0.000769542 | 0 |
| 6320 | LGTEITVEDQLAR | 0 | 0.000244121 | 0.001144749 |
| 6321 | GSWKLLTIDQDLM | 0 | 0.000272008 | 5.35547E-05 |
| 6322 | DEEATGTIQEDYLR | 0 | 0.003875399 | 0.000205972 |
| 6323 | DEEATGTIQEDYLRE | 0 | 0.001834505 | 0 |
| 6324 | DEEATGTIQEDYLREL | 0 | 0.010200796 | 0 |
| 6325 | DEEATGTIQEDYLRELL | 0 | 0.000721545 | 0 |
| 6326 | EEATGTIQEDYLR | 0.000923635 | 0.002231955 | 0.000325709 |
| 6327 | EEATGTIQEDYLREL | 0 | 0.00453884 | 0 |
| 6328 | LPPKPGTMEEEEEDDD | 0 | 0.004898261 | 0 |
| 6329 | LPPKPGTMEEEEEDDDY | 0 | 0.004392087 | 0 |
| 6330 | YPDPPGTMEEEEEDDD | 0 | 0.00187725 | 0 |
| 6331 | YPDPPGTMEEEEEDDDY | 0 | 0.00626969 | 0 |
| 6332 | YPDPPGTMEEEEEDDDYE | 0 | 0.000292191 | 0 |
| 6333 | APAALYQTIEENIK | 0 | 0.002188813 | 0 |
| 6334 | APAALYQTIEENIKIF | 0 | 0.000356973 | 0 |
| 6335 | ISRKLFDTLNEDLF | 0 | 0.000272614 | 0.000172096 |
| 6336 | ISRKLFDTLNEDLFQ | 0 | 0.005083291 | 0 |
| 6337 | ISRKLFDTLNEDLFQK | 0 | 0.002857652 | 7.63078E-05 |
| 6338 | ISRKLFDTLNEDLFQKI | 0 | 0.005940354 | 0 |
| 6339 | ISRKLFDTLNEDLFQKIL | 0 | 0.006369826 | 0 |
| 6340 | KLFDTLNEDLFQ | 0 | 0.001023569 | 0 |
| 6341 | RKLFDTLNEDLFQ | 0 | 0.000988596 | 0 |
| 6342 | RKLFDTLNEDLFQK | 0.000195001 | 0.001935765 | 0 |
| 6343 | RKLFDTLNEDLFQKI | 0 | 0.001343437 | 0 |
| 6344 | RKLFDTLNEDLFQKIL | 0 | 0.000342859 | 0 |
| 6345 | AQSPGAVEEILDRE | 0 | 0 | 0 |
| 6346 | ARAQSPGAVEEILDREN | 0.000262916 | 0 | 0 |
| 6347 | RAQSPGAVEEILDRE | 0.000562667 | 0 | 0 |
| 6348 | RAQSPGAVEEILDREN | 0.004285816 | 0 | 0.000226967 |
| 6349 | RAQSPGAVEEILDR | 0.001293854 | 0.008084021 | 0.000919984 |
| 6350 | ISVLAGSTVEDVLK | 0 | 0.002423286 | 0.023184807 |
| 6351 | ISVLAGSTVEDVLKK | 0 | 0.000576555 | 0.011656475 |
| 6352 | ISVLAGSTVEDVLKKA | 0 | 0.00241408 | 0.000819309 |
| 6353 | ISVLAGSTVEDVLKKAH | 0 | 0.000292904 | 0.000299506 |
| 6354 | ISVLAGSTVEDVLKKAHE | 0 | 0.00145825 | 0 |
| 6355 | SVLAGSTVEDVLK | 0.000104796 | 0.000942928 | 0 |
| 6356 | SISVLAGSTVEDVLKK | 0 | 2.0167E-05 | 0.000411369 |
| 6357 | QSISVLAGSTVEDVLK | 0 | 0 | 0 |
| 6358 | SISVLAGSTVEDVLK | 0.000111829 | 0 | 0.002957083 |
| 6359 | AMGIMNSFVNDIFER | 0.001544243 | 0.001408179 | 0.004387383 |
| 6360 | FVNDIFERI | 0 | 0 | 0.005152716 |
| 6361 | MNSFVNDIFERIA | 0 | 7.99219E-05 | 0 |
| 6362 | MNSFVNDIFERIAGE | 0 | 0.001110466 | 0.001433931 |
| 6363 | SVLDSFLKY | 0 | 0.000744124 | 0 |
| 6364 | DDIDLSDVELDDLGK | 0 | 0.000401784 | 0 |
| 6365 | DDIDLSDVELDDLGKD | 0 | 0 | 0 |
| 6366 | DIDLSDVELDDLGK | 0 | 0.002880122 | 0 |
| 6367 | LPVEDDIDLSDVELDDLGKD | 0 | 0.002761511 | 0 |
| 6368 | DIDLSDVELDDLGKD | 0 | 0.0025473 | 2.64894E-05 |
| 6369 | KPEGWLDDEPEYVPDPD | 0.029079818 | 0.024295394 | 0.001168315 |
| 6370 | RQFMAETQF | 0 | 7.05842E-05 | 0.001859878 |
| 6371 | DPFNEHTDEELWN | 0 | 0.00370189 | 0 |
| 6372 | DPFNEHTDEELWNA | 0 | 0.001597623 | 0 |
| 6373 | LDPFNEHTDEELWNA | 0 | 0.002084044 | 0 |
| 6374 | LDPFNEHTDEELWNAL | 0 | 0.000342196 | 0 |
| 6375 | LDPFNEHTDEELWN | 0 | 0.005476795 | 0 |
| 6376 | PFNEHTDEELWNALQEV | 0 | 0.001648365 | 0 |
| 6377 | TENDIYNFF | 0 | 0.00202746 | 0 |
| 6378 | DSSHAFTLDELRGKR | 0 | 0.000248913 | 0 |
| 6379 | FHTAWEPSRPFPVD | 0 | 0.00058397 | 0.002236752 |
| 6380 | TAWEPSRPFPVD | 0 | 0.000187484 | 0.000685299 |
| 6381 | EPSDTIENVKA | 0.005540022 | 0 | 0 |
| 6382 | EPSDTIENVKAK | 0.010067984 | 0 | 9.26971E-05 |
| 6383 | EPSDTIENVKAKIQ | 0.014851207 | 0.000121611 | 9.02046E-05 |
| 6384 | EPSDTIENVKAKIQD | 0.017941432 | 0 | 7.37121E-05 |
| 6385 | EPSDTIENVKAKIQDK | 0.036825248 | 0 | 0.000148255 |
| 6386 | EPSDTIENVKAKIQDKE | 0.158594423 | 4.5081E-05 | 0 |
| 6387 | EPSDTIENVKAKIQDKEG | 0.088524158 | 0 | 0.000151303 |
| 6388 | VEPSDTIENVKAK | 0.000786743 | 0 | 0 |
| 6389 | LDITKSVNELRMF | 0 | 4.96045E-05 | 0 |
| 6390 | YNSLEEIHIF | 0.000163479 | 0.00076568 | 0 |
| 6391 | AEYQDTTYINHVV | 0 | 0.000894348 | 0.000183836 |
| 6392 | EELGFRPEY | 0 | 0.002311054 | 0 |
| 6393 | ISWRSDYEDPA | 0.011778248 | 0.019223338 | 0 |
| 6394 | ISWRSDYEDPAF | 0.004101521 | 0.002399241 | 0 |
| 6395 | ISWRSDYEDPAFY | 0.007998045 | 0.00954572 | 0 |
| 6396 | ISWRSDYEDPAFYM | 0.000438358 | 0.002456431 | 0 |
| 6397 | AQAGWLQHDY | 0 | 8.69209E-05 | 0.002320437 |
| 6398 | DQVANSAFVERLR | 0 | 0.002262864 | 0 |
| 6399 | EQVANSAFVERVRKR | 0 | 0.000546129 | 0 |
| 6400 | KDQVANSAFVERLRKHG | 0 | 3.35371E-05 | 0 |
| 6401 | EPEPAFIDEDKMDQ | 0.001216711 | 0.002191451 | 0 |
| 6402 | EPEPEPAFIDEDKMDQ | 0.018436139 | 0.029969364 | 0 |
| 6403 | EPEPEPAFIDEDKMDQL | 0.021990866 | 0.010516388 | 0 |
| 6404 | EPEPEPAFIDEDKM | 0.005763987 | 0.008342384 | 0.000287682 |
| 6405 | EPEPEPAFIDEDKMD | 0.008933139 | 0.012084124 | 0 |
| 6406 | IEPEPEPAFIDEDKMDQ | 0 | 0.000183255 | 0 |
| 6407 | ALQNAVTTFVNRMK | 0 | 0.000714883 | 0 |
| 6408 | LQNAVTTFVNRMK | 0 | 0.000292682 | 0 |
| 6409 | DDEDSLSSAYIQRVN | 0 | 0 | 0.007203677 |
| 6410 | DDEDSLSSAYIQRVNT | 0 | 0 | 0.010410369 |
| 6411 | DDEDSLSSAYIQRVNTE | 0.000798402 | 0 | 0.022553407 |
| 6412 | DDEDSLSSAYIQRVNTEL | 0 | 0.000686042 | 0.019630955 |
| 6413 | DDEDSLSSAYIQRVNTELMK | 0.007991049 | 0 | 0 |
| 6414 | DEDSLSSAYIQRVN | 0 | 4.98633E-05 | 0.004120865 |
| 6415 | GDDEDSLSSAYIQRVNTELMK | 0.003585954 | 0.000483096 | 0 |
| 6416 | DEDSLSSAYIQRVNT | 0 | 0 | 0.006884002 |
| 6417 | DEDSLSSAYIQRVNTE | 0 | 0 | 0.019608012 |
| 6418 | DEDSLSSAYIQRVNTEL | 0 | 0 | 0.003367186 |
| 6419 | GDDEDSLSSAYIQRVNTEL | 0 | 0 | 0.013413368 |
| 6420 | GDDEDSLSSAYIQRVNTELM | 0 | 0 | 0.00208111 |
| 6421 | EDSLSSAYIQRVNTE | 0 | 0 | 0.001176547 |
| 6422 | GDDEDSLSSAYIQRVNTE | 0 | 0 | 0.005256931 |
| 6423 | GDDEDSLSSAYIQRVN | 0 | 0 | 0.002936306 |
| 6424 | GDDEDSLSSAYIQRVNT | 0 | 0 | 0.001803419 |
| 6425 | IQRVNTELMK | 0.000617573 | 2.44694E-05 | 0 |
| 6426 | SAYIQRVNTELMK | 0.016638602 | 0.006697153 | 0 |
| 6427 | SSAYIQRVNTELMK | 0.003126148 | 0 | 0.000169695 |
| 6428 | SAYIQRVNTELM | 0.006457868 | 0 | 0.000801813 |
| 6429 | DENGIVSQSEVIRAYDTT | 0 | 0 | 0.002072367 |
| 6430 | ENGIVSQSEVIRAYDTT | 0 | 0 | 0.00098187 |
| 6431 | GIVSQSEVIRAYDTT | 0 | 0 | 0.00596318 |
| 6432 | DITQSIEDDPLL | 0 | 0.002593437 | 0 |
| 6433 | LDITQSIEDDPLL | 0 | 0.001391209 | 0 |
| 6434 | LDITQSIEDDPLLD | 0 | 0.000602625 | 0.000375269 |
| 6435 | LDITQSIEDDPL | 0.000148789 | 0.001143212 | 0 |
| 6436 | GVQQDTILAEGLHFR | 0 | 0.000840126 | 0 |
| 6437 | ESLIPETIDDLMTPEEK | 0 | 0 | 0 |
| 6438 | IIIVVSNPVDIL | 0 | 0.014753643 | 0 |
| 6439 | IIIVVSNPVDILT | 0 | 0.003729944 | 0 |
| 6440 | IIIVVSNPVDILTYV | 0 | 0.000253496 | 0 |
| 6441 | IVSNPVDIL | 0 | 0.000516475 | 0 |
| 6442 | LLIVSNPVDIL | 0 | 0.000898518 | 0 |
| 6443 | KLLIVSNPVDIL | 0 | 0.000885588 | 0 |
| 6444 | VLIANSLNDDISL | 0 | 0.003727381 | 0 |
| 6445 | HVDDMPNA | 0.000467977 | 0.001523113 | 0.002311307 |
| 6446 | VAHVDDMPNA | 0.002020199 | 0 | 0.003920461 |
| 6447 | LENLSQSIQLSK | 0.000903602 | 0.001377897 | 0 |
| 6448 | LPAQAVDMIIDTQLCRGILF | 0 | 0.001693031 | 0 |
| 6449 | DLVGKYETLEEDSN | 0 | 0.003101543 | 0 |
| 6450 | DLVGKYETLEEDSNYV | 0 | 0.004079703 | 0 |
| 6451 | KYETLEEDSN | 0 | 0.000646246 | 0 |
| 6452 | LVGKYETLEEDSN | 0.000265361 | 0.000607388 | 0 |
| 6453 | VGKYETLEEDSN | 0 | 0.001895369 | 0.000306158 |
| 6454 | VGKYETLEEDSNYV | 0 | 0.002101884 | 0.00017868 |
| 6455 | DGDVVHGFYNPAVSRIVEAG | 0 | 0 | 0.002754445 |
| 6456 | DVVHGFYNPAVSRIVE | 0 | 0 | 0.006195242 |
| 6457 | GDVVHGFYNPAVSRIVE | 0 | 0.00030624 | 0.009288105 |
| 6458 | VHGFYNPAVSRIV | 0 | 0.000253138 | 0.00339909 |
| 6459 | VHGFYNPAVSRIVE | 0 | 0.010088988 | 0.095273479 |
| 6460 | VVHGFYNPAVSRIVE | 0 | 0.003892257 | 0.008923211 |
| 6461 | VDGDVVHGFYNPAVSRIVE | 0 | 0 | 0.003284607 |
| 6462 | VHGFYNPAVSRIVEA | 0 | 0 | 0.000513171 |
| 6463 | VHGFYNPAVSRIVEAG | 0 | 0 | 0.029836763 |
| 6464 | VVHGFYNPAVSRIVEAG | 0 | 8.7557E-05 | 0.004120677 |
| 6465 | EVIVDGDVVHGFY | 0.00227523 | 0 | 4.84746E-05 |
| 6466 | FGEVIVDGDVVHGFYNPA | 0 | 0 | 0.002608174 |
| 6467 | IADFGLARVIEDN | 0 | 0 | 0.000245489 |
| 6468 | IGPTHGQMTVTR | 0 | 0 | 0.007701462 |
| 6469 | IGPTHGQMTVTRL | 0 | 0 | 0.000741066 |
| 6470 | IGPTHGQMTVTRLVN | 0 | 0 | 0.000794759 |
| 6471 | LPLAHVLEL | 0 | 0 | 0.000513467 |
| 6472 | IIGRLLEV | 0 | 0 | 0.000599084 |
| 6473 | SIIGRLLEV | 0 | 0 | 0.005363906 |
| 6474 | FAHQASVARVVG | 0 | 0 | 0.000275441 |
| 6475 | GGNFAHQASVARVVG | 0 | 4.83393E-05 | 0.003904461 |
| 6476 | GGNFAHQASVARVVGQQ | 0 | 0 | 0.013863157 |
| 6477 | GNFAHQASVARVVG | 0 | 0 | 0.013821898 |
| 6478 | LFGGNFAHQASVARVVGQQ | 0 | 0 | 0.000169178 |
| 6479 | NFAHQASVARVVG | 0 | 8.26441E-06 | 0.001319648 |
| 6480 | GNFAHQASVARVVGQQ | 0 | 1.6208E-05 | 0.018889455 |
| 6481 | GNFAHQASVARVVGQQG | 0 | 0 | 0.000480842 |
| 6482 | NFAHQASVARVVGQQ | 0 | 0 | 0.013360152 |
| 6483 | DPLVEDGIPEFSHETA | 0 | 0 | 0.002005426 |
| 6484 | VEDGIPEFSHETA | 0 | 0 | 0.000418968 |
| 6485 | AEITDKLGL | 0 | 0.000613496 | 0 |
| 6486 | RIYWSDLSQRM | 0.000139001 | 0.003718668 | 0 |
| 6487 | SNRIYWSDLSQRM | 0.000435371 | 0.002617577 | 0 |
| 6488 | SNRIYWSDLSQRMI | 0 | 0.001567062 | 8.26224E-05 |
| 6489 | DFGQLSDVAEKL | 0 | 0.000100086 | 0 |
| 6490 | EVVEETVAEVTEVS | 0 | 0.00084188 | 3.0823E-05 |
| 6491 | HVGDLGNVTADKD | 0.000224627 | 0 | 0.000811616 |
| 6492 | VGDLGNVTADKD | 0.003424839 | 9.2698E-05 | 0 |
| 6493 | VGDLGNVTADKDG | 0.00091367 | 0.000139579 | 0 |
| 6494 | VGDLGNVTADKDGVA | 0.003693428 | 0.000659732 | 0 |
| 6495 | RIADISQVY | 0 | 0.000170788 | 0.002485514 |
| 6496 | VNILLTQITNLVFH | 0 | 0.000295333 | 0 |
| 6497 | AEVTEVSVGANPVQVE | 0 | 0.000778425 | 0 |
| 6498 | EVTEVSVGANPVQ | 0 | 0 | 0 |
| 6499 | EVTEVSVGANPVQV | 0 | 0.004963585 | 0 |
| 6500 | VAEVTEVSVGANPVQVE | 0 | 0.000465363 | 0 |
| 6501 | EPVATDVGDDLK | 0 | 0.000532752 | 0 |
| 6502 | EPVATDVGDDLKDE | 0 | 0.009099227 | 0 |
| 6503 | KEPVATDVGDDLKDE | 0 | 0.000370277 | 0 |
| 6504 | QEVTQDLHY | 0.000240004 | 0.000412218 | 0 |
| 6505 | TEITDDLHFY | 0 | 0.000543083 | 0 |
| 6506 | RDKEISADLADLSEEL | 0.001987182 | 0 | 0 |
| 6507 | IGFIAFSQIPELAK | 0 | 0.00101901 | 0.007421803 |
| 6508 | IGFIAFSQIPELAKR | 0 | 0.002648209 | 0.002752853 |
| 6509 | IGFIAFSQIPELAKRIK | 0 | 0.001218773 | 0 |
| 6510 | KMVYWTDITEPSIG | 0.000858293 | 0.003225503 | 0.00032776 |
| 6511 | VDKMVYWTDITEPSIG | 0 | 0.002913276 | 0 |
| 6512 | AKFYPEDVSEELIQ | 9.35726E-05 | 0.004662376 | 9.97467E-05 |
| 6513 | AKFYPEDVSEELIQD | 0 | 0.000665923 | 0 |
| 6514 | AKFYPEDVSEELIQDIT | 0.000879702 | 0.005383174 | 9.47622E-05 |
| 6515 | DVAEELIQDIT | 0 | 0.000973974 | 0 |
| 6516 | DVSEELIQDIT | 0 | 0.000677878 | 0 |
| 6517 | KFYPEDVSEELIQDIT | 0 | 0.000779001 | 0 |
| 6518 | YPEDVAEELIQDIT | 0 | 0.002825798 | 0 |
| 6519 | YPEDVSEELIQDIT | 0 | 0.011204189 | 0 |
| 6520 | VVSEDLVQQDVQ | 0 | 0 | 0 |
| 6521 | DVLILPEEVEWIK | 0.000411252 | 0 | 0 |
| 6522 | TDVLILPEEVEWIK | 0.002398403 | 0 | 0 |
| 6523 | QEVTNNLEF | 0 | 0.000676424 | 0 |
| 6524 | GVDIYSLVTENIQ | 0.006016607 | 0.008193955 | 0 |
| 6525 | GVDIYSLVTENIQWPN | 0 | 0.01467886 | 0.000391746 |
| 6526 | LITNEIVDYISGG | 0.003758114 | 0 | 0 |
| 6527 | AKSKIEDIRAEQEREG | 0 | 0.000257078 | 5.31089E-05 |
| 6528 | IEDIRAEQEREG | 4.7605E-05 | 0.000281739 | 0.001119738 |
| 6529 | KSKIEDIRAEQEREG | 1.69555E-05 | 0.000271786 | 6.97795E-05 |
| 6530 | AQKSQSTQISQEL | 0 | 0.003558817 | 2.86502E-05 |
| 6531 | AQKSQSTQISQELE | 0 | 0.016306197 | 0.000463023 |
| 6532 | AQKSQSTQISQELEE | 0 | 0.059552811 | 0 |
| 6533 | AQKSQSTQISQELEEL | 0.000136396 | 0.030412581 | 0.000100306 |
| 6534 | AQKSQSTQISQELEELR | 7.0507E-05 | 0.011946804 | 0 |
| 6535 | KSQSTQISQELE | 2.40911E-05 | 0.002208937 | 0 |
| 6536 | KSQSTQISQELEE | 0 | 0.005934968 | 0 |
| 6537 | KSQSTQISQELEELR | 0 | 0.00442178 | 0.000295511 |
| 6538 | QKSQSTQISQELEELR | 0.001445686 | 0.014482813 | 0 |
| 6539 | QKSQSTQISQELEELRAE | 0.014043297 | 0.007078295 | 6.23481E-05 |
| 6540 | QKSQSTQISQELEELRAEQ | 0.046867252 | 0.035284682 | 0.000416645 |
| 6541 | QKSQSTQISQELEELRAEQQ | 0.039485535 | 0.039180208 | 0 |
| 6542 | QKSQSTQISQELEELRAEQQR | 0.018560768 | 0.01381988 | 0 |
| 6543 | QKSQSTQISQELEE | 0 | 0.013505623 | 0 |
| 6544 | QKSQSTQISQELEEL | 0 | 0.005266565 | 0 |
| 6545 | DQMAQKSQSTQISQE | 0 | 0.003516915 | 0 |
| 6546 | DQMAQKSQSTQISQEL | 0 | 0.008842899 | 0.001267014 |
| 6547 | DQMAQKSQSTQISQELE | 0 | 0.001960512 | 0 |
| 6548 | DQMAQKSQSTQISQELEE | 0 | 0.004468767 | 0 |
| 6549 | GDQMAQKSQSTQISQELEE | 0 | 0.006485624 | 0 |
| 6550 | HHGDQMAQKSQSTQISQELEE | 6.93385E-05 | 0.011475967 | 0 |
| 6551 | SHHGDQMAQKSQSTQISQELEE | 0 | 0.001168025 | 0 |
| 6552 | MAQKSQSTQISQELE | 0 | 0.001885781 | 0 |
| 6553 | MAQKSQSTQISQELEE | 0 | 0.00564358 | 5.87134E-05 |
| 6554 | QKSQSTQISQELE | 0 | 0.000606255 | 0 |
| 6555 | GDQMAQKSQSTQISQEL | 0 | 0.008270131 | 0 |
| 6556 | GDQMAQKSQSTQISQELE | 0 | 0.002795457 | 0 |
| 6557 | HGDQMAQKSQSTQISQEL | 0 | 0.002101021 | 0 |
| 6558 | ISQELEELRAEQQ | 0.012970767 | 0.000282142 | 7.25564E-05 |
| 6559 | ISQELEELRAEQQR | 0.017762204 | 0.005643584 | 8.08681E-05 |
| 6560 | ISQELEELRAEQQRL | 0 | 0.000372071 | 0 |
| 6561 | QKSQSTQISQELEELRAEQQRL | 0.028601868 | 0.044880896 | 0 |
| 6562 | QKSQSTQISQELEELRAEQQRLK | 0.004450283 | 0.02532897 | 0 |
| 6563 | SQELEELRAEQQ | 0.000296321 | 0 | 0 |
| 6564 | SQSTQISQELEELRAEQQRL | 0.006402835 | 0.008377903 | 0 |
| 6565 | STQISQELEELRAEQQRL | 0 | 0.000346055 | 0 |
| 6566 | SQELEELRAEQQR | 0.00174619 | 0.001658252 | 0 |
| 6567 | SQSTQISQELEELRAEQQRLK | 0.002379469 | 0.002154146 | 0 |
| 6568 | STQISQELEELRAEQQR | 0.000262582 | 0 | 0 |
| 6569 | SQSTQISQELEELR | 0 | 0.008283643 | 0 |
| 6570 | SQSTQISQELEELRAEQ | 0.002700392 | 0.000221222 | 0 |
| 6571 | SQSTQISQELEELRAEQQ | 0.005457359 | 0.000425326 | 0 |
| 6572 | KPRPTRAEGSDVANAVLD | 0 | 0.001951081 | 0 |
| 6573 | KPRPTRAEGSDVANAVLDG | 0 | 0.00466247 | 1.42748E-05 |
| 6574 | APPEEDCTSVTDLPN | 0 | 0.001014458 | 0 |
| 6575 | APPEEDCTSVTDLPNAF | 0 | 0.000123976 | 0 |
| 6576 | APPEEDCTSVTDLPNAFD | 0 | 0 | 0 |
| 6577 | PPEEDCTSVTDLPNA | 0 | 0.000393529 | 0.000134352 |
| 6578 | IGLSVADLAESIM | 0 | 0.000758967 | 0 |
| 6579 | IGLSVADLAESIMK | 0.002826558 | 0.004102451 | 0 |
| 6580 | IGLSVADLAESIMKNL | 0.000281802 | 0.002662307 | 0 |
| 6581 | IGLSVADLAESIMKNLR | 3.0834E-05 | 0.004650709 | 0 |
| 6582 | LSVADLAESIMK | 0.000122977 | 0.00045079 | 0 |
| 6583 | LSVADLAESIMKNLR | 0 | 0.000468948 | 0 |
| 6584 | SVADLAESIMK | 0 | 0.00097376 | 2.36293E-05 |
| 6585 | SVADLAESIMKNL | 0 | 0.000388457 | 0 |
| 6586 | VADLAESIMKNL | 0 | 0.000127779 | 4.49871E-05 |
| 6587 | IPAPMSELTETVV | 0 | 0.001409704 | 0 |
| 6588 | NATLAVANITNAD | 0 | 0 | 0.000454162 |
| 6589 | VQNATLAVANITNAD | 0 | 0 | 0.000997676 |
| 6590 | VYYRVQNATLAVAN | 0 | 0 | 0.003128179 |
| 6591 | FIFANVSEADDR | 0.00091853 | 0.000206974 | 0 |
| 6592 | SEIPQTERM | 0 | 0.001734271 | 7.07372E-05 |
| 6593 | GNIANSQIAASSVR | 0 | 1.6711E-05 | 0.00358329 |
| 6594 | NPRVNHLYSDLSDAL | 0 | 0.022023857 | 0 |
| 6595 | NPRVNHLYSDLSDALV | 0 | 0.000690462 | 0 |
| 6596 | NPRVNHLYSDLSDALVI | 0 | 0.000482411 | 0 |
| 6597 | NPRVNHLYSDLSDALVIFQ | 0 | 0.001290621 | 0 |
| 6598 | VNHLYSDLSDAL | 0 | 0.012826423 | 0 |
| 6599 | VNHLYSDLSDALV | 0 | 0.001607348 | 0 |
| 6600 | VNPRVNHLYSDLSDAL | 0 | 0.001601891 | 0.000259565 |
| 6601 | EVTQIPQEEHAA | 0 | 0.000916787 | 0.000214607 |
| 6602 | GVEEVTQIPQEEHAA | 0 | 0.011104651 | 0 |
| 6603 | GVEEVTQIPQEEHAAN | 0 | 0.005900533 | 0 |
| 6604 | TGVEEVTQIPQEEHAAN | 0 | 0.003492501 | 0 |
| 6605 | GVEEVTQIPQEEHA | 0 | 0.002532244 | 0.00027132 |
| 6606 | TGVEEVTQIPQEEHAA | 0 | 7.83754E-05 | 0 |
| 6607 | MRKYQMTGVEEVTQIPQ | 0.010252012 | 0 | 5.90546E-05 |
| 6608 | RKYQMTGVEEVTQIPQ | 0.021032819 | 0 | 0 |
| 6609 | VPDGFLSELTQQL | 0.000331464 | 0.003592324 | 0 |
| 6610 | VPDGFLSELTQQLA | 0 | 0.000809926 | 0 |
| 6611 | VPDGFLSELTQQLAQ | 0.000111765 | 0.002054381 | 0 |
| 6612 | VPDGFLSELTQQLAQA | 0 | 0.004028061 | 0 |
| 6613 | AVLEYLTAEILELAG | 0 | 0.000340846 | 0 |
| 6614 | AVLEYLTAEILELAGN | 0 | 0.0001849 | 0 |
| 6615 | YLTAEILEL | 0 | 0 | 0.000728197 |
| 6616 | IVEKVSGSQIVDID | 0 | 0 | 0.00323303 |
| 6617 | IVEKVSGSQIVDIDK | 0 | 0 | 0.013440223 |
| 6618 | IVEKVSGSQIVDIDKR | 0 | 4.60215E-05 | 0.043299508 |
| 6619 | IVEKVSGSQIVDIDKRK | 0 | 2.8916E-05 | 0.008352232 |
| 6620 | VPVIVEKVSGSQIVDIDKRK | 0 | 0 | 0.000285588 |
| 6621 | VEKVSGSQIVDIDK | 0 | 0.000238354 | 0.002986926 |
| 6622 | VEKVSGSQIVDIDKR | 0 | 1.91181E-05 | 0.002631461 |
| 6623 | VPVIVEKVSGSQIVDIDKR | 0 | 0 | 0.009147609 |
| 6624 | KVSGSQIVDIDKR | 0 | 3.38927E-05 | 0 |
| 6625 | KVSGSQIVDIDKRK | 0 | 3.77754E-05 | 1.81587E-05 |
| 6626 | YPDRVPVIVEKVSGSQIVDID | 0 | 0 | 0.000867296 |
| 6627 | SDLVNMGISVVSYTLK | 0 | 0 | 0.002619744 |
| 6628 | SSDLVNMGISVVSYTLK | 0 | 0 | 0.00732536 |
| 6629 | DDVAQTDLLQIDPN | 0 | 0.000781494 | 0 |
| 6630 | KDDVAQTDLLQIDPN | 0 | 0.003463567 | 0 |
| 6631 | EIYETELVDIEK | 0 | 0.002056473 | 0 |
| 6632 | EIYETELVDIEKA | 0 | 0.000734653 | 0 |
| 6633 | IAEIYETELVDIEK | 0 | 0.000235551 | 0.000246092 |
| 6634 | IYETELVDIEKA | 0 | 0.006617156 | 0 |
| 6635 | IYETELVDIEKAI | 0 | 0.000359642 | 0 |
| 6636 | YETELVDIEKA | 0 | 0.001044844 | 0 |
| 6637 | ERESSIRQLEADIMDIN | 0.003191955 | 0.002812419 | 0 |
| 6638 | ERETAIRQLEADILDVN | 0.002028206 | 0.001097065 | 0 |
| 6639 | ESSIRQLEADIMDIN | 0.005186473 | 0.004227227 | 0 |
| 6640 | ETAIRQLEADILDVN | 0.00376136 | 0.002192106 | 0 |
| 6641 | RESSIRQLEADIMDIN | 0.001727099 | 0.002651615 | 0 |
| 6642 | RETAIRQLEADILDVN | 0 | 0.001223797 | 0 |
| 6643 | PVNDLNADLIQV | 0 | 0.001545647 | 0 |
| 6644 | VPVNDLNADLIQVA | 0 | 0.00080581 | 0 |
| 6645 | VPVNDLNADLIQ | 0.000175821 | 0.006603552 | 0 |
| 6646 | VHARYACDDMEGQLV | 0.001724826 | 0.00026802 | 0 |
| 6647 | IDQINTDLNLERSHAQ | 0.002010192 | 0 | 0 |
| 6648 | EISLVGDDLDPER | 0.008307733 | 0 | 0 |
| 6649 | EISLVGDDLDPERE | 0.01212253 | 0.000193798 | 0 |
| 6650 | EISLVGDDLDPERES | 0.002606541 | 0.000906151 | 0 |
| 6651 | ISLVGDDLDPER | 0.000145932 | 0 | 0 |
| 6652 | ISLVGDDLDPERE | 0.003880306 | 0 | 0 |
| 6653 | EVELNELEPEKQP | 0.031043598 | 0.000262482 | 0 |
| 6654 | EVELNELEPEKQPM | 0.004879906 | 0 | 0 |
| 6655 | EVELNELEPEKQPMN | 0.005501258 | 0.000321968 | 0 |
| 6656 | KEVELNELEPEKQPMN | 0.001548414 | 0 | 0 |
| 6657 | KEVELNELEPEKQP | 0.013149842 | 0.000102862 | 0 |
| 6658 | MKEVELNELEPEKQP | 0.002265094 | 0 | 0.000240949 |
| 6659 | TPPSYESVDDINADK | 0 | 0 | 0 |
| 6660 | TPPSYESVDDINADKN | 0.000796885 | 0.00109344 | 0.000141087 |
| 6661 | SQHQAIERF | 0 | 0 | 0.000109759 |
| 6662 | TPEQKKELSDIAHRIV | 0 | 0.002426591 | 0 |
| 6663 | TPEQKKELSDIAHRIVAPG | 0 | 0.000844764 | 0 |
| 6664 | ADAEDLSGSIASPDVK | 0 | 0 | 0.000992752 |
| 6665 | AEDLSGSIASPDVK | 0 | 0 | 0.001084697 |
| 6666 | DAEDLSGSIASPDVK | 0 | 0 | 0.011087485 |
| 6667 | DLSGSIASPDVK | 0 | 0 | 0.00066234 |
| 6668 | EDLSGSIASPDVK | 0 | 0 | 0.001509653 |
| 6669 | LPAALSATEIEKSIS | 0 | 0.000250471 | 0.001565375 |
| 6670 | DGNGYISAAELRHVMTN | 0 | 0 | 0 |
| 6671 | GYISAAELRHV | 0 | 0 | 0.00084392 |
| 6672 | GYISAAELRHVM | 0 | 0 | 0.003211281 |
| 6673 | GYISAAELRHVMT | 8.55656E-05 | 0.000460548 | 0.018288879 |
| 6674 | GYISAAELRHVMTN | 0 | 0 | 0.005035497 |
| 6675 | EIRQMSGAQIKIANPVE | 0 | 0.00020769 | 0.002700019 |
| 6676 | EVAGGQLR | 0 | 0 | 0.001945012 |
| 6677 | EPEDFVIATGEVHSVR | 0 | 6.15098E-05 | 0.008950758 |
| 6678 | EPEDFVIATGEVHSVREF | 0 | 0 | 0.001428287 |
| 6679 | VGTSVASAEQDELSQR | 0 | 0.000756575 | 0 |
| 6680 | ARPDDEEGAAVAPG | 0 | 0 | 0.000837669 |
| 6681 | ARPDDEEGAAVAPGHP | 0 | 4.00867E-05 | 0.036323736 |
| 6682 | ARPDDEEGAAVAPGHPL | 0 | 0 | 0.000355308 |
| 6683 | ARPDDEEGAAVAPGHPLA | 0 | 0 | 0.000404589 |
| 6684 | RPDDEEGAAVAPG | 0 | 0 | 0.003022452 |
| 6685 | DDEEGAAVAPGHP | 0 | 0 | 0.018437499 |
| 6686 | DEEGAAVAPGHP | 0 | 0 | 0.006243485 |
| 6687 | RPDDEEGAAVAPGHP | 0 | 1.12939E-05 | 0.086347414 |
| 6688 | RPDDEEGAAVAPGHPL | 0 | 0.001216138 | 0.005194015 |
| 6689 | EPETDLSPGLPAAH | 0 | 2.29144E-05 | 0.004599436 |
| 6690 | LPEEEPETDLSPGLP | 0 | 0.01030078 | 0 |
| 6691 | LPEEEPETDLSPGLPA | 0 | 0.01254897 | 0 |
| 6692 | TPEIPAGLPSPRSE | 0.003634425 | 0.000476797 | 0.000974802 |
| 6693 | VTPEIPAGLPSPRSE | 0.000151629 | 0 | 0.003074958 |
| 6694 | EEEDGAAVAEKSHR | 0 | 0 | 3.24911E-05 |
| 6695 | EFDAATVSEHTM | 0 | 6.11682E-05 | 0 |
| 6696 | FEFDAATVSEHTM | 0 | 0.001638987 | 0 |
| 6697 | VPTDEEQATGLEREIM | 7.6711E-05 | 0.002959444 | 0 |
| 6698 | YVTPVNRNV | 0 | 9.28545E-05 | 9.26947E-05 |
| 6699 | DARSYTAAVAN | 0 | 0 | 0.000698353 |
| 6700 | DARSYTAAVANRAKGGG | 0 | 0 | 0.002965849 |
| 6701 | DIHENVDTDLPGSLG | 0 | 0.02740005 | 0.000650901 |
| 6702 | DIHENVDTDLPGSLGQS | 0 | 0.004742636 | 0.00023218 |
| 6703 | IHENVDTDLPGSL | 0 | 0.008189622 | 0 |
| 6704 | IHENVDTDLPGSLG | 0 | 0.027123516 | 0 |
| 6705 | EGDIHENVDTDLPGSLG | 0 | 0.000122035 | 0 |
| 6706 | GDIHENVDTDLPGSLG | 0 | 0.004964861 | 0 |
| 6707 | GEGDIHENVDTDLPGSLG | 0 | 0.004163577 | 0 |
| 6708 | IHENVDTDLPGSLGQ | 0 | 0.002790465 | 0 |
| 6709 | IHENVDTDLPGSLGQS | 0 | 0.004575997 | 9.61313E-05 |
| 6710 | EGLYSRTLAGSIT | 0 | 0.000692865 | 0.000218853 |
| 6711 | EGVAESLFLPRTDY | 0 | 0.001750931 | 0.000114783 |
| 6712 | EGVAESLFLPRTDYS | 0 | 0.002664051 | 0 |
| 6713 | VTEGVAESLFLPRTDYS | 0 | 0.001399574 | 0.000110844 |
| 6714 | ELVTEGVAESLF | 0.000219443 | 0.000310585 | 3.11801E-05 |
| 6715 | ELVTEGVAESLFLP | 0.000473066 | 0.000433208 | 0.00036272 |
| 6716 | ELVTEGVAESLFLPR | 0.032028436 | 0.000255663 | 0.008800212 |
| 6717 | GELVTEGVAESLFLPR | 0.027321159 | 0.000723534 | 0.052110915 |
| 6718 | NGELVTEGVAESLFLPR | 0 | 8.73122E-05 | 0.070849392 |
| 6719 | GELVTEGVAESLFLP | 0.000142307 | 0.000183001 | 0.00213671 |
| 6720 | NGELVTEGVAESLFLP | 0.000221022 | 0 | 0.002450938 |
| 6721 | VTEGVAESLFLP | 0.0001366 | 0.000642447 | 0.001083731 |
| 6722 | LVTEGVAESLFLPR | 0.006017377 | 0 | 0.00023874 |
| 6723 | VTEGVAESLFLPR | 0.010469189 | 0.000338011 | 0.005802775 |
| 6724 | NGELVTEGVAESLF | 0 | 3.35824E-05 | 0.001296465 |
| 6725 | FLPRTDYSF | 0 | 0.005195269 | 0.000517909 |
| 6726 | GKPVTTGVSETVF | 0.013617448 | 0.008081765 | 0 |
| 6727 | GKPVTTGVSETVFLP | 0.000320315 | 0.001556312 | 0.000228276 |
| 6728 | GKPVTTGVSETVFLPR | 0.078887148 | 0.011754748 | 0.005468482 |
| 6729 | GKPVTTGVSETVFLPRED | 0.000702046 | 0 | 0 |
| 6730 | KPVTTGVSETVF | 0.004442451 | 0.001251848 | 0 |
| 6731 | NGKPVTTGVSETVF | 0.000198654 | 0.002354616 | 0 |
| 6732 | NGKPVTTGVSETVFLPR | 0.014936864 | 0.002015302 | 0.001426083 |
| 6733 | RNGKPVTTGVSETVFLPR | 0 | 9.45572E-05 | 0 |
| 6734 | KPVTTGVSETVFLPR | 0.016047786 | 0.001310113 | 0 |
| 6735 | KPVTTGVSETVFLPRED | 0 | 0.003106654 | 0.000133181 |
| 6736 | KPVTTGVSETVFLPREDH | 0 | 0.031582188 | 0 |
| 6737 | KPVTTGVSETVFLPREDHL | 0 | 0.009895785 | 0 |
| 6738 | TGVSETVFLPRED | 0 | 0.003351889 | 0 |
| 6739 | TTGVSETVFLPRED | 0.000127204 | 0.001007884 | 9.9703E-05 |
| 6740 | TTGVSETVFLPREDH | 0.000152507 | 0.0170466 | 0.001348416 |
| 6741 | GVSETVFLPRED | 4.92385E-05 | 0.007652607 | 0.000796557 |
| 6742 | GVSETVFLPREDH | 0 | 0.079376755 | 0.000331063 |
| 6743 | GVSETVFLPREDHL | 0 | 0.021455492 | 0 |
| 6744 | GVSETVFLPREDHLF | 5.11791E-05 | 0.016181613 | 0 |
| 6745 | KPVTTGVSETVFLPREDHLF | 0 | 0.023117656 | 0 |
| 6746 | TGVSETVFLPREDHLF | 0 | 0.034134995 | 0 |
| 6747 | TTGVSETVFLPREDHLF | 0 | 0.007775923 | 0.000700518 |
| 6748 | SETVFLPREDH | 0 | 0.005232286 | 0.000654872 |
| 6749 | TGVSETVFLPREDH | 0 | 0.144720782 | 0 |
| 6750 | TGVSETVFLPREDHL | 0 | 0.03155012 | 0.001336707 |
| 6751 | TTGVSETVFLPREDHL | 0 | 0.003636068 | 0.000138745 |
| 6752 | VSETVFLPREDH | 0.000285111 | 0.007180289 | 0 |
| 6753 | VSETVFLPREDHLF | 0 | 0.000464391 | 0.002096875 |
| 6754 | GVKQIATGVSDTI | 0 | 0.000363945 | 0 |
| 6755 | SPGFGVKQIATGVSDTI | 0 | 0.001942398 | 0.000345527 |
| 6756 | VTWLRNGKPVTTGVS | 0 | 0 | 0.008649585 |
| 6757 | TYQEIWTSLGSAMPEPE | 0 | 0.001384592 | 0 |
| 6758 | YQEIWTSLGSAMPEPE | 0 | 0.008457913 | 0 |
| 6759 | GERFTCTVTHTDLPSPLK | 0 | 0.000290007 | 0 |
| 6760 | HTDLPSPLK | 0 | 0.000532387 | 0 |
| 6761 | THTDLPSPLK | 0 | 0.009571363 | 8.30132E-05 |
| 6762 | TVTHTDLPSPLK | 0 | 0.057330135 | 2.53782E-05 |
| 6763 | VTHTDLPSPLK | 0 | 0.080787889 | 0 |
| 6764 | VTHTDLPSPLKQT | 0 | 0.003556345 | 0 |
| 6765 | TVTHTDLPSPL | 0.000740655 | 0.000821604 | 0.000184329 |
| 6766 | VTHTDLPSPL | 0 | 0.001226614 | 0 |
| 6767 | LIQDAGRTQIAPG | 0 | 0 | 0.002037328 |
| 6768 | LIQDAGRTQIAPGS | 0 | 0 | 0.001203836 |
| 6769 | LIQDAGRTQIAPGSQ | 0 | 0 | 0.001504335 |
| 6770 | VSLIQDAGRTQIAPGSQ | 0 | 0 | 0.001207186 |
| 6771 | VSLIQDAGRTQIAPGS | 0 | 0 | 0.000937504 |
| 6772 | DRAGVLAGHDNRVS | 0 | 0 | 0.004206053 |
| 6773 | EPAEVTATVLASRDDHG | 0 | 0.003839893 | 0 |
| 6774 | EPAEVTTTVLVR | 0 | 0.000536504 | 0 |
| 6775 | EPAEVTTTVLVRRD | 0 | 0.000656981 | 0 |
| 6776 | EPAEVTTTVLVRRDHHG | 0 | 0.000102304 | 0 |
| 6777 | LKREPAVGEPAEVTTT | 0 | 0 | 0.014012101 |
| 6778 | GPWSPEVPSTLEVYSCHPPRSP | 0 | 0 | 0.003304543 |
| 6779 | GPWSPEVPSTLEVYSCHPPRSPA | 0 | 0.000131997 | 0.001222625 |
| 6780 | TLEVYSCHPPRSP | 6.15303E-05 | 0 | 0.00157724 |
| 6781 | VPSTLEVYSCHPPRSP | 0 | 0 | 0.002270801 |
| 6782 | DIKHHCAAITPGRG | 0 | 0 | 0.00139395 |
| 6783 | LDIKHHCAAITPGRG | 0 | 0 | 0.00050803 |
| 6784 | KVYYREDLSPSITQ | 0.001018466 | 0 | 0 |
| 6785 | AVSFGRSVPEAPVGRS | 0 | 7.13844E-05 | 0 |
| 6786 | VSFGRSVPEAPVG | 0 | 0.001826058 | 2.28753E-05 |
| 6787 | VSFGRSVPEAPVGR | 0 | 7.439E-05 | 0 |
| 6788 | HLAVSFGRSVPEAPV | 0 | 0 | 0.001211407 |
| 6789 | LAVSFGRSVPEAPV | 0 | 0 | 0.010824944 |
| 6790 | THLAVSFGRSVPEAPV | 0 | 3.50419E-05 | 0.034830802 |
| 6791 | THLAVSFGRSVPEAPVG | 0 | 0 | 0.000609292 |
| 6792 | FPHGITNGASWYNVPG | 0.000294937 | 0 | 0.011529366 |
| 6793 | DGILGMAYPRISVN | 0 | 0 | 0.001269998 |
| 6794 | DGILGMAYPRISVNNV | 0 | 0 | 0.001256292 |
| 6795 | FDGILGMAYPRISVN | 0 | 0 | 0.000535848 |
| 6796 | SFARLSLTY | 0 | 0.000299369 | 0 |
| 6797 | GPIPPSTPKFAYGKVTLEK | 0 | 0 | 0.000710794 |
| 6798 | IKTYTITEGSLR | 0 | 0.000394711 | 0 |
| 6799 | LPVSRIKTYTITEGSLR | 0 | 0.009532887 | 0 |
| 6800 | SRIKTYTITEGSLR | 0 | 0.002207422 | 9.1347E-05 |
| 6801 | LPVSRIKTYTITEGSL | 0 | 0.002433832 | 0 |
| 6802 | ILGLSYAMANTGIAL | 0 | 0.000294465 | 0.003823242 |
| 6803 | ILGLAYAMAHTGV | 0 | 0 | 0.001717114 |
| 6804 | LFLVRAANAYGISDPS | 0 | 0 | 0 |
| 6805 | GHSVTEGVSETSF | 0.002027632 | 0.000942463 | 0.011029415 |
| 6806 | GHSVTEGVSETSFL | 0.001118667 | 0.000469533 | 0.022320326 |
| 6807 | GHSVTEGVSETSFLS | 0.03064688 | 0 | 0.10884713 |
| 6808 | GHSVTEGVSETSFLSK | 0.010199807 | 5.43346E-06 | 0.070996915 |
| 6809 | GHSVTEGVSETSFLSKS | 0 | 4.9007E-05 | 0.001489891 |
| 6810 | GHSVTEGVSETSFLSKSDH | 0 | 4.37938E-05 | 0.00150915 |
| 6811 | HSVTEGVSETSFLS | 0.00770058 | 0.000166816 | 0.000681176 |
| 6812 | NGHSVTEGVSETSFLS | 0.00349491 | 0.00051385 | 0.043965937 |
| 6813 | NGHSVTEGVSETSFLSK | 0.000883477 | 0.000152452 | 0.037594839 |
| 6814 | SNGHSVTEGVSETSFLSK | 0 | 0 | 0.005212298 |
| 6815 | NGHSVTEGVSETSF | 0 | 0 | 0.00087784 |
| 6816 | NGHSVTEGVSETSFL | 0.000225928 | 0 | 0.008831324 |
| 6817 | SNGHSVTEGVSETSFLS | 0 | 0 | 0.003490745 |
| 6818 | KSNVSDAVAQSTR | 0 | 0 | 0.000550684 |
| 6819 | LKSNVSDAVAQSTR | 0 | 0 | 0.004994435 |
| 6820 | NVSDAVAQSTR | 0 | 0 | 0.001734833 |
| 6821 | SNVSDAVAQSTR | 3.2861E-05 | 0 | 0.002313679 |
| 6822 | GVSETSFLSKSDHSFFKISYLTFLP | 0.000120683 | 0.00728396 | 0 |
| 6823 | SDHSFFKISYLTLLPS | 4.34887E-05 | 0 | 0.003428638 |
| 6824 | SFFKISYLTLLPSAEES | 0 | 0 | 0.0001259 |
| 6825 | LPKLIDFSAQIAEGMAY | 0.001047152 | 0 | 9.75478E-05 |
| 6826 | DHGGIITAIAQGMET | 0 | 0.003312552 | 0 |
| 6827 | DHGGIITAIAQGMETN | 0.000212878 | 0.001681357 | 0.000260161 |
| 6828 | GDHGGIITAIAQGMETN | 0 | 0.000895926 | 0 |
| 6829 | GDHGGIITAIAQGMET | 0.001802485 | 0.000868667 | 0 |
| 6830 | KVNQIGSVTESLQ | 0.002008775 | 0.005007176 | 0 |
| 6831 | LKVNQIGSVTESLQ | 0.000294776 | 0.002023113 | 0 |
| 6832 | VNQIGSVTESLQ | 0.000392079 | 0.001190049 | 0 |
| 6833 | DTIRASLISAVSDK | 0 | 0 | 0 |
| 6834 | SEDTIRASLISAVSDK | 0 | 0.000180658 | 0.003560115 |
| 6835 | GVSKGLAESLQPDYSER | 0 | 0.000996079 | 0 |
| 6836 | KGLAESLQPDYSER | 1.56065E-05 | 0.002190366 | 0 |
| 6837 | KGLAESLQPDYSERL | 0.00213008 | 0.000586749 | 0 |
| 6838 | SGVSKGLAESLQPDYS | 0.001323831 | 0.000253865 | 0.000248826 |
| 6839 | WSGVSKGLAESLQPDYS | 0.00615407 | 0 | 0.000379855 |
| 6840 | WSGVSKGLAESLQPD | 0.003359028 | 0 | 0.014323883 |
| 6841 | LGKTVTNAVVTVPA | 0 | 0.000816809 | 0 |
| 6842 | DLKTRTYHSVGDSVLR | 0.001600594 | 0.015178666 | 8.83258E-05 |
| 6843 | KTRTYHSVGDSVLR | 0.000263145 | 0.002054478 | 9.20504E-05 |
| 6844 | LKTRTYHSVGDSVLR | 0.000310116 | 0.002554923 | 0 |
| 6845 | TRTYHSVGDSVLR | 2.35811E-05 | 0.000896548 | 8.29111E-05 |
| 6846 | MIATLSDACEREFG | 0 | 0.000470122 | 0 |
| 6847 | AQKNPGSVAENNL | 0 | 0.000361464 | 0 |
| 6848 | NPGSVAENNL | 0 | 0.001506689 | 0 |
| 6849 | DPTTLTDEEIN | 0 | 0.000801204 | 0 |
| 6850 | DPTTLTDEEINR | 0 | 0.003438277 | 8.23989E-05 |
| 6851 | KTDPTTLTDEEINR | 6.02972E-05 | 0.001143448 | 0.000303716 |
| 6852 | TDPTTLTDEEINR | 0 | 0.002199256 | 0.001570774 |
| 6853 | IEKTDPTTLTDEEIN | 0 | 0.001091059 | 0 |
| 6854 | KTDPTTLTDEEIN | 0 | 0.004331333 | 0.000566514 |
| 6855 | FTDKTGTLTENEMQ | 0 | 0.00123522 | 0.000435959 |
| 6856 | TDKTGTLTENEMQ | 0 | 0.001345698 | 0 |
| 6857 | EVAQTTALPDEDDDL | 0 | 0.004929143 | 0 |
| 6858 | HDLEVAQTTALPDEDDDL | 0 | 0 | 0 |
| 6859 | GRDKYEPAAVSEQGDK | 0 | 5.8204E-06 | 0.0004399 |
| 6860 | DGPKNVRSDISDQEEDEE | 0.000687038 | 0.002185507 | 0 |
| 6861 | KVAILTDDEEEQKR | 0 | 0.000402205 | 0 |
| 6862 | SPLSEEEEEDEQK | 0 | 0.001123838 | 0 |
| 6863 | SPLSEEEEEDEQKEP | 0 | 0.000716251 | 0 |
| 6864 | EAADVADDQEEPAT | 0.000163629 | 0.000588839 | 0 |
| 6865 | ILQPTQVPEQEME | 0.00194123 | 0.011653095 | 0 |
| 6866 | SILQPTQVPEQEME | 0.000330119 | 0.002409955 | 6.79341E-05 |
| 6867 | VLNEIVSILQPTQVPE | 0 | 0.001975337 | 0 |
| 6868 | IAEFTTNLTEEEEK | 0 | 0.001309816 | 0.000701951 |
| 6869 | IAEFTTNLTEEEEKS | 0 | 0.003007208 | 5.75443E-05 |
| 6870 | RIAEFTTNL | 0 | 0 | 0.000956016 |
| 6871 | DDVSEKTSLADQEE | 0 | 0.000870721 | 0 |
| 6872 | DDVSEKTSLADQEEVR | 0 | 0.001822501 | 2.86611E-05 |
| 6873 | DRRYADLTEDQLPS | 0 | 0.000152291 | 0 |
| 6874 | ISKDRRYADLTEDQLPS | 0.001145397 | 0.001006742 | 0 |
| 6875 | KDRRYADLTEDQLPS | 0.000916441 | 0.002787853 | 0 |
| 6876 | SKDRRYADLTEDQLPS | 0 | 0.002224493 | 0 |
| 6877 | IIHRNGIPDEQIV | 0.001218946 | 8.92411E-05 | 0 |
| 6878 | QIIHRNGIPDEQIV | 0.001254036 | 0 | 0 |
| 6879 | IPDEQIVVM | 0.000595831 | 0 | 0 |
| 6880 | IPDEQIVVMM | 0.001705126 | 0 | 0 |
| 6881 | VPKDEEWELAQDQLIR | 0.002961241 | 4.57107E-05 | 0.005326482 |
| 6882 | VPKDEEWELAQDQLIRNSG | 0.001165264 | 0 | 0.000219058 |
| 6883 | SPVDYDGGRTR | 0 | 0 | 0.000402681 |
| 6884 | VIADYESGRAIPNN | 0 | 0 | 0.00659481 |
| 6885 | VIADYESGRAIPNNQ | 0 | 0 | 0.000583414 |
| 6886 | VIADYESGRAIPNNQV | 0 | 0 | 0.001883049 |
| 6887 | VPAAYRGVGDDQ | 0.005097869 | 0 | 9.40012E-06 |
| 6888 | VPAAYRGVGDDQL | 0.006887003 | 7.59802E-05 | 0 |
| 6889 | VPAAYRGVGDDQLG | 0.013620264 | 0 | 0.000102423 |
| 6890 | VPAAYRGVGDDQLGE | 0.011704981 | 0 | 0 |
| 6891 | VPAAYRGVGDDQLGEE | 0.022448265 | 0 | 0.000216477 |
| 6892 | EDVLKEAVANKGP | 0 | 0.000101952 | 0.010577133 |
| 6893 | EDVLKEAVANKGPVS | 4.67084E-05 | 0 | 0.025336908 |
| 6894 | GREDVLKEAVANKGPVS | 0 | 0 | 0.01532357 |
| 6895 | LPYGREDVLKEAVANKGPVS | 0 | 0 | 0.03248252 |
| 6896 | REDVLKEAVANKGPVS | 0 | 0 | 0.019378465 |
| 6897 | GREDVLKEAVANKGP | 0 | 0 | 0.005954338 |
| 6898 | LPYGREDVLKEAVANKGP | 0 | 0 | 0.009765351 |
| 6899 | REDVLKEAVANKGP | 0 | 0 | 0.006184806 |
| 6900 | AHLQVKTTDVQLQ | 0.000317406 | 0.00119643 | 2.55945E-05 |
| 6901 | SAHLQVKTTDVQLQ | 0 | 0.001140269 | 0 |
| 6902 | DIEVDLLK | 0 | 0.000313767 | 0 |
| 6903 | DIEVDLLKN | 0 | 0.000264625 | 0 |
| 6904 | FHPSDIEVDLLKN | 0.009969844 | 0.288130515 | 0 |
| 6905 | FHPSDIEVDLLKNG | 0.002334051 | 0.123575085 | 0 |
| 6906 | FHPSDIEVDLLKNGE | 0 | 0.082094849 | 0.000905102 |
| 6907 | FHPSDIEVDLLKNGER | 0 | 0.040603501 | 0 |
| 6908 | GFHPSDIEVDLLKNGER | 0 | 0.078018474 | 0.000269252 |
| 6909 | HPSDIEVDLLKN | 0.008734987 | 0.523221832 | 0.000192233 |
| 6910 | SGFHPSDIEVDLLKNGER | 0.000837835 | 0.275814597 | 0.000422306 |
| 6911 | SGFHPSDIEVDLLKNGERIE | 0 | 0.007987808 | 0 |
| 6912 | SGFHPSDIEVDLLKNGERIEK | 0 | 0.004049532 | 0 |
| 6913 | VSGFHPSDIEVDLLKNGERIEK | 0 | 0.003381098 | 0 |
| 6914 | IEVDLLKN | 0 | 0.000285875 | 0 |
| 6915 | PSDIEVDLLKNGER | 0 | 0.001782 | 0 |
| 6916 | FHPSDIEVD | 0 | 0.001218335 | 0 |
| 6917 | FHPSDIEVDLL | 0.001378989 | 0.064986799 | 0 |
| 6918 | FHPSDIEVDLLK | 0.019552734 | 0.573335979 | 0 |
| 6919 | GFHPSDIEVDLLK | 0.066433122 | 1.034849532 | 0 |
| 6920 | GFHPSDIEVDLLKN | 0.039451997 | 0.46724267 | 0 |
| 6921 | GFHPSDIEVDLLKNG | 0.022749479 | 0.351788294 | 0.001577891 |
| 6922 | GFHPSDIEVDLLKNGE | 0.001447311 | 0.16167407 | 0.001905276 |
| 6923 | SGFHPSDIEVDLLKNGE | 0.003774549 | 0.175865969 | 0.001641415 |
| 6924 | VSGFHPSDIEVDLLKNGE | 0.001571591 | 0.134007044 | 0.003185906 |
| 6925 | VSGFHPSDIEVDLLKNGER | 0.001117091 | 0.2166449 | 0 |
| 6926 | VSGFHPSDIEVDLLKNGERIE | 0 | 0.007666207 | 0 |
| 6927 | HPSDIEVDLLK | 0.021503511 | 1.066545614 | 0.005097098 |
| 6928 | HPSDIEVDLLKNG | 0.000922849 | 0.148733121 | 0.000956354 |
| 6929 | HPSDIEVDLLKNGE | 0 | 0.187369427 | 6.68821E-05 |
| 6930 | HPSDIEVDLLKNGER | 0.000566761 | 0.122594931 | 0.000866018 |
| 6931 | HPSDIEVDLLKNGERIE | 0 | 0.002116969 | 4.88642E-05 |
| 6932 | HPSDIEVDLLKNGERIEK | 0 | 0.001665611 | 0 |
| 6933 | PSDIEVDLLK | 5.09598E-05 | 0.000737714 | 0 |
| 6934 | SDIEVDLLK | 0 | 0.001761682 | 0 |
| 6935 | GFHPSDIEVDLL | 0.003348606 | 0.114353022 | 0 |
| 6936 | HPSDIEVDLL | 0.002361056 | 0.052095164 | 9.68774E-05 |
| 6937 | SGFHPSDIEVDLL | 0.008534235 | 0.212343232 | 0 |
| 6938 | SGFHPSDIEVDLLK | 0.10690896 | 1.760210018 | 0 |
| 6939 | SGFHPSDIEVDLLKN | 0.048790368 | 0.641403962 | 0 |
| 6940 | SGFHPSDIEVDLLKNG | 0.051241644 | 0.667703718 | 0.001869592 |
| 6941 | VSGFHPSDIEVDLLKNG | 0.025097119 | 0.153080154 | 0.000111638 |
| 6942 | YVSGFHPSDIEVDLLKNG | 0 | 0.001092034 | 0.000114641 |
| 6943 | YVSGFHPSDIEVDLLKNGER | 0 | 0.005960612 | 0 |
| 6944 | SGFHPSDIEVD | 0 | 0.003885956 | 0.000613589 |
| 6945 | SGFHPSDIEVDL | 0 | 0.000824455 | 0 |
| 6946 | VSGFHPSDIEVDLL | 0.008291627 | 0.119047111 | 0.00010084 |
| 6947 | VSGFHPSDIEVDLLK | 0.046829647 | 0.553675547 | 0.001847323 |
| 6948 | VSGFHPSDIEVDLLKN | 0.037003046 | 0.241261688 | 0 |
| 6949 | YVSGFHPSDIEVDLLKN | 0.001979557 | 0.02072477 | 0.000481977 |
| 6950 | YVSGFHPSDIEVDLL | 0.000677222 | 0.008165865 | 0.000175983 |
| 6951 | YVSGFHPSDIEVDLLK | 0.003300393 | 0.02389915 | 0 |
| 6952 | EENTVDVEILR | 0 | 0.000789208 | 2.11328E-05 |
| 6953 | SVEENTVDVEILR | 0 | 0.002475466 | 0.001344524 |
| 6954 | ELNHEQESLEVDMAR | 0 | 0.001984092 | 0.000787118 |
| 6955 | ELNHEQESLEVDMARLQ | 0 | 0.000135048 | 0 |
| 6956 | LNHEQESLEVD | 6.39717E-06 | 0.000340412 | 0 |
| 6957 | LNHEQESLEVDMA | 0.00020213 | 0.005519798 | 0 |
| 6958 | LNHEQESLEVDMAR | 0 | 0.025926819 | 0 |
| 6959 | LNHEQESLEVDMARL | 0 | 0.001414044 | 0.000105554 |
| 6960 | LNHEQESLEVDMARLQ | 0 | 0.0108538 | 0 |
| 6961 | NHEQESLEVDMAR | 0 | 0.002627336 | 0 |
| 6962 | DDEVVQREEEAIQ | 0.001707122 | 0 | 0 |
| 6963 | DEVVQREEEAIQ | 0.001426054 | 0.000124466 | 0.000394736 |
| 6964 | TDDEVVQREEEAIQ | 0.000115331 | 0 | 0 |
| 6965 | DLGKSREGSRTDDEVVQREEE | 0 | 0.000195093 | 0 |
| 6966 | KSREGSRTDDEVVQREEE | 0 | 0.00019371 | 0 |
| 6967 | REGSRTDDEVVQRE | 0 | 7.54342E-05 | 0 |
| 6968 | SREGSRTDDEVVQRE | 0 | 0.000728279 | 0 |
| 6969 | SREGSRTDDEVVQREE | 0 | 0.000367248 | 0 |
| 6970 | SREGSRTDDEVVQREEE | 1.06617E-05 | 0.001436781 | 0 |
| 6971 | SREGSRTDDEVVQ | 0 | 7.17732E-05 | 0 |
| 6972 | DYEGSRTQEEIVAK | 0 | 0.001710928 | 0 |
| 6973 | YEGSRTQEEIVAK | 0 | 0.000684734 | 0 |
| 6974 | LFDLRADQELMT | 0.0012608 | 0 | 0 |
| 6975 | KSRVVAGEVQVQR | 0 | 0 | 0.000264902 |
| 6976 | VFKSRVVAGEVQVQR | 6.58908E-06 | 5.7146E-06 | 0.003364126 |
| 6977 | VFKSRVVAGEVQVQRL | 0 | 0 | 0.000130121 |
| 6978 | VFKSRVVAGEVQVQRLQ | 0 | 0 | 0.00152844 |
| 6979 | DYTGEDVTPQN | 0.006392525 | 0 | 0 |
| 6980 | GVPKDYTGEDVTPQN | 0.031026256 | 0 | 0 |
| 6981 | GVPKDYTGEDVTPQNFL | 0.017399919 | 6.01322E-05 | 0 |
| 6982 | KDYTGEDVTPQN | 0.018337433 | 6.16222E-05 | 0 |
| 6983 | VPKDYTGEDVTPQN | 0.168335383 | 9.52631E-05 | 0 |
| 6984 | VPKDYTGEDVTPQNF | 0.059680927 | 0 | 0 |
| 6985 | KDYTGEDVTPQNF | 0.001960025 | 0.000376873 | 0 |
| 6986 | KDYTGEDVTPQNFL | 0.00212495 | 0 | 0 |
| 6987 | VPKDYTGEDVTPQNFL | 0.104152862 | 0.00021238 | 0 |
| 6988 | GVPKDYTGEDVTPQ | 0.008610478 | 0 | 0 |
| 6989 | KDYTGEDVTPQ | 0.00269447 | 0 | 0 |
| 6990 | VPKDYTGEDVTPQ | 0.089771395 | 5.10827E-05 | 0 |
| 6991 | KPANDITSQLEINFGDL | 0 | 0.000373255 | 0 |
| 6992 | IGGGQTKTFAPEEIS | 0 | 0.007391663 | 3.19757E-05 |
| 6993 | IGGGQTKTFAPEEISA | 0 | 0.002789332 | 0 |
| 6994 | IQVDIGGGQTKTFAPE | 0 | 0 | 0 |
| 6995 | IDVQISTPRPVK | 0 | 0 | 0.001442238 |
| 6996 | IIDVQISTPRPVK | 0 | 0 | 0.002800038 |
| 6997 | VPVVNVEVSPFT | 0.010986723 | 0 | 0.000660875 |
| 6998 | EQLDVTTSEYEKEK | 0 | 0.003279771 | 0 |
| 6999 | IEQLDVTTSEYEKEK | 0 | 0.002448327 | 0 |
| 7000 | QLDVTTSEYEKEK | 0 | 0.000723175 | 0 |
| 7001 | VVIGMDVAASEFFR | 0 | 0 | 0.000898905 |
| 7002 | TPVKEPNSENVDISSG | 0 | 0.000298634 | 0 |
| 7003 | GDSDLQLDRISVYYNEA | 0.004618925 | 0 | 0 |
| 7004 | QLDRISVYY | 0.002733591 | 0 | 0 |
| 7005 | DSRTYSVGVCTFAVGPE | 0 | 0 | 0.006408563 |
| 7006 | GPRSYTIAVASLGK | 0 | 0 | 0.000592484 |
| 7007 | GPRSYTIAVASLGKG | 1.902E-05 | 0.000409842 | 0.02081797 |
| 7008 | GPRSYTIAVASLGKGVA | 0.00081524 | 0.000323501 | 0.005676157 |
| 7009 | GWEGAKYAVAVGSL | 0 | 0 | 0.007648275 |
| 7010 | VGWEGAKYAVAVGSL | 0 | 0 | 0.001891142 |
| 7011 | WEGAKYAVAVGSL | 0 | 0 | 0.009540867 |
| 7012 | NAKYAISMARKIG | 0 | 0.002839107 | 0.000236327 |
| 7013 | NAKYAISMARKIGAR | 0 | 0.000487398 | 0 |
| 7014 | EHVRVTKAFSVN | 0 | 0.000604216 | 0 |
| 7015 | EHVRVTKAFSVNIF | 0 | 0.002926366 | 0 |
| 7016 | IPVSRGFPIAE | 0 | 0 | 0.004870312 |
| 7017 | IPVSRGFPIAEV | 0 | 0 | 0.004136788 |
| 7018 | IPVSRGFPIAEVF | 0 | 0 | 0.012589874 |
| 7019 | IPVSRGFPIAEVFT | 0 | 0 | 0.008386205 |
| 7020 | LPHPFAITIFEDTIY | 0 | 0.000630502 | 0 |
| 7021 | FPIAEVFTLKPLEFGKPN | 0 | 0.00389105 | 0 |
| 7022 | GNPITIFQERDP | 0 | 0.001498681 | 0.000227547 |
| 7023 | GNPITIFQERDPSK | 0 | 0.0123951 | 0 |
| 7024 | GNPITIFQERDPSKI | 0 | 0.002599913 | 0 |
| 7025 | GNPITIFQERDPSKIK | 0 | 0.003133499 | 0 |
| 7026 | INGNPITIFQERDPSKIK | 0.000771571 | 0.003296352 | 0 |
| 7027 | NPITIFQERDP | 0 | 0.001687368 | 0 |
| 7028 | VINGNPITIFQERDPSKIK | 0 | 0.001344483 | 0 |
| 7029 | NPITIFQERDPSK | 0 | 0.002617792 | 4.34228E-05 |
| 7030 | NPITIFQERDPSKI | 0 | 0.00142922 | 0.000197386 |
| 7031 | NPITIFQERDPSKIK | 0 | 0.002241607 | 0 |
| 7032 | INGNPITIFQERDPS | 0 | 0.004257549 | 0 |
| 7033 | INGNPITIFQERDPSK | 0 | 0.004465094 | 0 |
| 7034 | INGNPITIFQERDPSKI | 0 | 0.002087091 | 0 |
| 7035 | NPITIFQERDPS | 0 | 0.00318982 | 0 |
| 7036 | VINGNPITIFQERDPSKI | 0 | 0.000826827 | 0 |
| 7037 | VINGNPITIFQERDPSK | 0 | 0.000603297 | 0 |
| 7038 | AGPAQVPEEGAVT | 0.002418658 | 0 | 0 |
| 7039 | LPEQTTKALPVR | 0 | 0.000488149 | 0 |
| 7040 | NPPELPEQTTKALPVR | 0 | 0.008206351 | 0 |
| 7041 | RTGKPIAVKL | 0 | 0.001005749 | 0 |
| 7042 | DGVAVLKV | 0 | 0 | 0.000214353 |
| 7043 | SPASRSISLLRLSFH | 0.000168801 | 0.002090824 | 0 |
| 7044 | DDVWDSVSIISFPEK | 0 | 0.002091097 | 0 |
| 7045 | DVWDSVSIISFPEK | 0.000134277 | 0.002066122 | 0.000103272 |
| 7046 | SPKDDVWDSVSIISFPEK | 0.013866194 | 0.036313256 | 0 |
| 7047 | SPKDDVWDSVSIISFPEKE | 0.002098146 | 0.016381277 | 0 |
| 7048 | SPKDDVWDSVSIISFPEKEQ | 0 | 0.002900159 | 0 |
| 7049 | SPKDDVWDSVSIISFPE | 0.00892885 | 0.011168608 | 0 |
| 7050 | DVWDSVSIISFPEKE | 0 | 0.001015454 | 0.000105978 |
| 7051 | DPMSVLLARSLEVRH | 0 | 0 | 0.001591967 |
| 7052 | DPVTVVVDDLR | 0.000229615 | 0.001517693 | 0.001940067 |
| 7053 | KTLAVLLDNILQRIGKL | 0.009084064 | 0 | 0 |
| 7054 | TLAVLLDNILQRIGKL | 0 | 0 | 0 |
| 7055 | RTISVILFL | 0 | 0 | 0.003249759 |
| 7056 | FSGQQRSVILDNL | 0 | 0.015475833 | 0 |
| 7057 | FSGQQRSVILDNLP | 0 | 0.004112991 | 0 |
| 7058 | FSGQQRSVILDNLPH | 0 | 0.005810605 | 0 |
| 7059 | FSGQQRSVILDNLPHPY | 0 | 0.011220143 | 0.000197829 |
| 7060 | FSGQQRSVILDNLPHPYA | 0 | 0.00153401 | 0 |
| 7061 | GQQRSVILDNL | 0 | 0.00451222 | 0 |
| 7062 | SGQQRSVILDNL | 0 | 0.002510767 | 0 |
| 7063 | GQQRSVILDNLP | 0 | 0.001561038 | 2.73067E-05 |
| 7064 | GQQRSVILDNLPH | 0 | 0.004034803 | 0 |
| 7065 | SGQQRSVILDNLPH | 0 | 0.000911636 | 0 |
| 7066 | APNTITVLELE | 0 | 0.000310643 | 0 |
| 7067 | TPKEVPAESVTVWIDPL | 0 | 0.000278117 | 0 |
| 7068 | TPKEVPAESVTVWIDPLD | 0 | 0.001825156 | 0.000431452 |
| 7069 | TPKEVPAESVTVWIDPLDAT | 0 | 0.000596285 | 0 |
| 7070 | VPAESVTVWIDPLD | 0 | 0.003065272 | 0 |
| 7071 | VPAESVTVWIDPLDAT | 0.056972343 | 0.008989241 | 0 |
| 7072 | ALGLTVGLVGIIIGTIFIIKGLRK | 5.25362E-05 | 0 | 0.000366696 |
| 7073 | AVRDMRQTVAVGVI | 0 | 0.000547101 | 3.75159E-05 |
| 7074 | AVRDMRQTVAVGVIK | 0 | 0.011781406 | 0 |
| 7075 | VRDMRQTVAVGVI | 0 | 0.002048072 | 0 |
| 7076 | DMRQTVAVGVIK | 0 | 0 | 0 |
| 7077 | RDMRQTVAVGVIK | 0 | 0.003996476 | 0.000221078 |
| 7078 | VRDMRQTVAVGVIK | 0 | 0.006729578 | 8.8669E-05 |
| 7079 | RQTVAVGVIKAVDK | 0 | 0 | 0.001749948 |
| 7080 | VGVIKAVDKKAAGAG | 0 | 0 | 0.000964736 |
| 7081 | DTISLSVIVPVSRP | 0 | 4.07787E-05 | 0.000961865 |
| 7082 | HSDTISLSVIVPVSRP | 0 | 0 | 0.00421008 |
| 7083 | SDTISLSVIVPVSRP | 0 | 0 | 0.010246985 |
| 7084 | LPVGLIVGVTALGL | 0.000740754 | 0.001693745 | 0 |
| 7085 | LVVPVGGLSFLVNHD | 0 | 0 | 0.005344968 |
| 7086 | LVVPVGGLSFLVNHDF | 0 | 0 | 0.007688516 |
| 7087 | VVPVGGLSFLVNHD | 0 | 0 | 0.00178405 |
| 7088 | VPVGGLSFLVNHDF | 0.000121641 | 0 | 0.005016952 |
| 7089 | VVPVGGLSFLVNHDF | 0.000816083 | 0 | 0.004548624 |
| 7090 | IPETVGYSLLPLAPVG | 0 | 0 | 0.003983778 |
| 7091 | TVGYSLLPLAPVG | 0 | 0 | 0.000327527 |
| 7092 | EPHQKKQTRASVVIPVTG | 0 | 0 | 0.003169285 |
| 7093 | IPALLLTIPDFIFA | 0 | 0.000544357 | 0 |
| 7094 | IPALLLTIPDFIFAN | 0 | 0 | 0 |
| 7095 | IPALLLTIPDFIFANV | 0 | 0 | 0 |
| 7096 | LLLTIPDFIFA | 0 | 0 | 0 |
| 7097 | LLLTIPDFIFAN | 0 | 0.000122064 | 0 |
| 7098 | ADDELSFKRGDILKVLNEE | 0 | 0 | 0.007471268 |
| 7099 | DDELSFKRGDILKVLN | 0.000112854 | 0 | 0.007078262 |
| 7100 | DDELSFKRGDILKVLNEE | 0.000452882 | 0 | 0.017362876 |
| 7101 | DELSFKRGDILKVLN | 0 | 0 | 0.00622964 |
| 7102 | DELSFKRGDILKVLNEE | 0 | 0 | 0.00973206 |
| 7103 | DYEARTEDDLSFHKG | 0 | 0.002594631 | 0 |
| 7104 | DYEARTEDDLSFHKGE | 0 | 0.003339061 | 0.00016032 |
| 7105 | DYEARTEDDLSFHKGEK | 0 | 0.001062992 | 5.9304E-05 |
| 7106 | YDYEARTEDDLSFHKGE | 0 | 0.002303839 | 0 |
| 7107 | DFMKPVISIVDELLE | 0 | 0.004555942 | 0 |
| 7108 | EDFMKPVISIVDELLE | 0 | 0.001282888 | 0 |
| 7109 | EEDFMKPVISIVDELLE | 0 | 0.002551832 | 0 |
| 7110 | FMKPVISIVDELL | 0 | 0.001041483 | 0 |
| 7111 | FMKPVISIVDELLE | 0 | 0.007629598 | 0 |
| 7112 | KPVISIVDELL | 0 | 0.00202083 | 0 |
| 7113 | MKPVISIVDELL | 0 | 0.013002508 | 0 |
| 7114 | MKPVISIVDELLE | 0.000324364 | 0.05514562 | 0 |
| 7115 | FMKPVISIVDELLEAG | 0 | 0 | 0 |
| 7116 | KPVISIVDELLE | 0.001035116 | 0.010076255 | 0 |
| 7117 | KPVISIVDELLEAG | 0 | 0.001427058 | 1.17525E-05 |
| 7118 | MKPVISIVDELLEAG | 0 | 0.002130655 | 0 |
| 7119 | SVIEQIVYV | 0 | 0 | 0.000389625 |
| 7120 | KPYEECQTLIDEMVN | 0 | 0.003892424 | 0 |
| 7121 | KPYEECQTLIDEMVNT | 0 | 0.001176678 | 0 |
| 7122 | KPYEECQTLIDEMVNTI | 0 | 0.000598648 | 0.000432644 |
| 7123 | IRQGKEATAVVDQIL | 0 | 0.004991211 | 0.002287475 |
| 7124 | DVVPGGAVVDEIH | 0 | 0 | 0.005146035 |
| 7125 | DVVPGGAVVDEIHS | 0.000524659 | 0 | 0.018534274 |
| 7126 | DVVPGGAVVDEIHSL | 0.001550394 | 0 | 0.015132891 |
| 7127 | DVVPGGAVVDEIHSLI | 0 | 7.30733E-05 | 0.008811506 |
| 7128 | RDVVPGGAVVDEIHSLI | 0 | 0 | 0.000820729 |
| 7129 | RDVVPGGAVVDEIHS | 0.000560805 | 0 | 0.001085679 |
| 7130 | VPGGAVVDEIHS | 0 | 0 | 0.002943231 |
| 7131 | FSEVLHGLIGRTQ | 0 | 0 | 0.000159878 |
| 7132 | GVIRSLMAF | 0 | 0 | 0.000546227 |
| 7133 | QIMKSIIPVVM | 0 | 0.000191671 | 0 |
| 7134 | RPEQIMKSIIPVVM | 7.41597E-05 | 0.002098228 | 0 |
| 7135 | IQNVIKLVDELME | 0.002259084 | 1.32106E-05 | 4.71039E-05 |
| 7136 | IQNVIKLVDELMEAPG | 0 | 0 | 0 |
| 7137 | LADFAELLRALRRDLG | 0 | 0 | 0.000196732 |
| 7138 | VFLENVIR | 0.003519173 | 0.001865718 | 0.002907879 |
| 7139 | AIIDPGDSDIIRSMPEQTGEK | 0.003047329 | 0.003858559 | 0.004711489 |
| 7140 | PGDSDIIRSMPEQTGEK | 0.018629139 | 0.017462301 | 0.012894432 |
| 7141 | SDIIRSMPEQTGEK | 0.000740568 | 0.000965558 | 8.02417E-05 |
| 7142 | LGTVMRSLGQNPTEA | 0 | 0.000353572 | 0.000129526 |
| 7143 | EPAEGNESSEAPSPVN | 0.038569289 | 0.015586841 | 0 |
| 7144 | NTEEAANTMVNYY | 0.00380809 | 0 | 0 |
| 7145 | RIFQEPTEPK | 0 | 0.000474885 | 0.000118715 |
| 7146 | SENPGQTHF | 0 | 0.000457275 | 0 |
| 7147 | EANLMKTLQHDKL | 0 | 0.000735773 | 8.36916E-05 |
| 7148 | QLAQEAAQKRLQAEE | 0 | 0 | 0.000821001 |
| 7149 | ALMASLDAEKAQGQK | 0.000134144 | 0.000545089 | 0 |
| 7150 | LMASLDAEKAQGQ | 0 | 0.002391732 | 0 |
| 7151 | LMASLDAEKAQGQK | 0 | 0.006657047 | 0.001045315 |
| 7152 | MALMASLDAEKAQGQK | 0.004894624 | 0.019937496 | 3.90838E-05 |
| 7153 | MASLDAEKA | 3.00861E-05 | 0.00037759 | 0 |
| 7154 | MASLDAEKAQG | 0 | 0.000168414 | 0 |
| 7155 | MASLDAEKAQGQ | 0 | 0.001200385 | 0 |
| 7156 | LMASLDAEKAQGQKK | 0 | 0.00056308 | 0.000145491 |
| 7157 | MASLDAEKAQGQK | 2.51382E-06 | 0.001985261 | 1.12915E-05 |
| 7158 | MASLDAEKAQGQKK | 0 | 7.28193E-05 | 0 |
| 7159 | MALMASLDAEKA | 0.002722353 | 0.003866588 | 0 |
| 7160 | MALMASLDAEKAQG | 3.83419E-05 | 0.002986392 | 0 |
| 7161 | MALMASLDAEKAQGQ | 4.06676E-05 | 0.005554219 | 0.000327982 |
| 7162 | DSGVYACGAGMNTDRG | 6.21393E-06 | 0 | 0.009037996 |
| 7163 | DSGVYACGAGMNTDRGK | 0 | 0 | 0.004422836 |
| 7164 | DSGVYACGAGMNTDRGKT | 0 | 6.59109E-05 | 0.000867907 |
| 7165 | QPYSLVTTLNSDLK | 0 | 0.035709838 | 0 |
| 7166 | SVRVTTMDAELE | 0 | 0.000680259 | 0 |
| 7167 | DEIQCQLAIPVSSL | 0 | 0 | 0.001228057 |
| 7168 | DEIQCQLAIPVSSLN | 0 | 6.14373E-05 | 0.010380071 |
| 7169 | DEIQCQLAIPVSSLNSQ | 0 | 0 | 0.000364843 |
| 7170 | EIQCQLAIPVSSLN | 5.88888E-05 | 0 | 0.001533451 |
| 7171 | FTLTISSLQAEDVA | 0.002484898 | 0 | 0 |
| 7172 | TISSLQAEDVA | 0.000525085 | 0 | 0 |
| 7173 | TLTISSLQAEDVA | 0.001242931 | 0 | 0 |
| 7174 | TLTISSLQAEDVAV | 0.004624337 | 0 | 0 |
| 7175 | TISSLQAEDVAVY | 0.002155977 | 0 | 0 |
| 7176 | FTLTISSLQPEDF | 0.002716407 | 0.001684229 | 0 |
| 7177 | TLTISSLQPEDF | 0.002910414 | 0.001715379 | 0 |
| 7178 | VTTVETQNLEGLH | 0 | 0.001710632 | 0 |
| 7179 | VTTVETQNLEGLHH | 0 | 0.001159163 | 0 |
| 7180 | VTTVETQNLEGLHHD | 0 | 0.001618308 | 0 |
| 7181 | VTTVETQNLEGLHHDG | 0 | 0.002972303 | 0 |
| 7182 | GALQPDEAEEWPGRP | 0 | 0.000165877 | 0 |
| 7183 | SPMGALQPDEAEEWPGRP | 0 | 0.0003759 | 0 |
| 7184 | IVPSLNPDGRERAQEKD | 0.001004593 | 2.17926E-05 | 0 |
| 7185 | IAAVDTDSPREV | 0 | 7.82363E-05 | 0.000104169 |
| 7186 | SPLDPETTRSSEL | 0 | 0.000902181 | 0 |
| 7187 | SPLDPETTRSSELT | 0 | 0.001633598 | 0.000215628 |
| 7188 | FHRDLSGLDAETLLK | 0.000449536 | 0 | 0 |
| 7189 | RDLAAVNL | 0.001008987 | 0.001079568 | 0 |
| 7190 | RQIGSVDTFRIP | 0 | 0.000704114 | 0 |
| 7191 | AVILRALSL | 0.002042762 | 0 | 0 |
| 7192 | ILHSLSVPEEAISPG | 0 | 0.001160133 | 9.856E-05 |
| 7193 | DIAVDGEPLG | 0 | 0.002686594 | 0 |
| 7194 | FDIAVDGEPLG | 0.00120447 | 0.002961445 | 0 |
| 7195 | NPTVFFDIAVDGEPLG | 0.003924131 | 0.015094556 | 0.000114018 |
| 7196 | NPTVFFDIAVDGEPLGR | 0.000973786 | 0.00519013 | 0.004033317 |
| 7197 | VNPTVFFDIAVDGEPLGR | 0.006348986 | 0.013656488 | 5.04194E-05 |
| 7198 | VNPTVFFDIAVDGEPLGRVS | 0.002648799 | 0.004262966 | 0 |
| 7199 | NPTVFFDIAVDGEP | 0.000402472 | 0.006979885 | 0 |
| 7200 | NPTVFFDIAVDGEPL | 0.002202804 | 0.006836203 | 0 |
| 7201 | VNPTVFFDIAVDGEPLG | 0.015162668 | 0.031744985 | 1.63601E-05 |
| 7202 | VNPTVFFDIAVDGEP | 0.002081087 | 0.007084765 | 0 |
| 7203 | VNLPINGNGKQ | 9.63281E-06 | 0.000611778 | 0.000771673 |
| 7204 | HPSAIDPEAIP | 0.005095187 | 0 | 2.82954E-05 |
| 7205 | SHPSAIDPEAIP | 0.00566977 | 0 | 0 |
| 7206 | SHPSAIDPEAIPVK | 0.00182225 | 3.45606E-05 | 0 |
| 7207 | DPAVIRSLTLEPD | 0.002105956 | 0.006017935 | 9.52888E-05 |
| 7208 | DPAVIRSLTLEPDP | 0 | 0.001183912 | 0 |
| 7209 | DPAVIRSLTLEPDPI | 0.006265034 | 0.005393484 | 0 |
| 7210 | DPAVIRSLTLEPDPII | 0.029724872 | 0.010121943 | 0 |
| 7211 | DPAVIRSLTLEPDPIIVP | 0.005584992 | 0.004277365 | 0 |
| 7212 | DPAVIRSLTLEPDPIIVPG | 0.003418949 | 0.004040423 | 0 |
| 7213 | VIRSLTLEPD | 0 | 0 | 0 |
| 7214 | KDPAVIRSLTLEPDPII | 0.005938213 | 0.001382144 | 0.0080161 |
| 7215 | KDPAVIRSLTLEPDPIIVP | 0.001862207 | 0.003485524 | 0 |
| 7216 | VIRSLTLEPDPII | 0.000859915 | 0.001753915 | 0 |
| 7217 | PAVIRSLTLEPDPIIVPG | 0 | 0.000473916 | 0 |
| 7218 | VIRSLTLEPDPIIVP | 0.000108089 | 0.000939188 | 0 |
| 7219 | VIRSLTLEPDPIIVPG | 0.000138223 | 0.023760435 | 0 |
| 7220 | IDDQYLLGDALLVHPV | 0 | 0 | 0.000804002 |
| 7221 | LPGRENYSSVDANGIQ | 0.000208505 | 0.001056382 | 6.69127E-05 |
| 7222 | EDSIKGLQPEDVN | 0.000494417 | 0 | 0 |
| 7223 | SEDSIKGLQPEDVN | 0.004183632 | 0.000596399 | 0.000955716 |
| 7224 | SEDSIKGLQPEDVNL | 0.001523258 | 0 | 0 |
| 7225 | EVTVEGEELNHEQ | 0.0021369 | 0 | 0 |
| 7226 | EVTVEGEELNHEQE | 0.009613265 | 0 | 0 |
| 7227 | EVTVEGEELNHEQES | 0.00401203 | 0 | 0 |
| 7228 | HPSQLVLTLEGEDLGEL | 0.000640745 | 0.002362696 | 0.000147918 |
| 7229 | IVHDLDTCEVTVE | 0 | 0.000412654 | 0 |
| 7230 | IVHDLDTCEVTVEGEE | 9.59447E-05 | 0.011672249 | 0 |
| 7231 | VHDLDTCEVTVEGEE | 0 | 0.001868594 | 0 |
| 7232 | DINSDGVLDE | 0 | 0.000611041 | 0 |
| 7233 | DINSDGVLDEQ | 4.73016E-05 | 0.017426792 | 0 |
| 7234 | DINSDGVLDEQE | 0.000309445 | 0.047950534 | 0 |
| 7235 | DINSDGVLDEQEL | 0.000131116 | 0.0406239 | 0 |
| 7236 | DINSDGVLDEQELE | 0 | 0.015065566 | 0.002377075 |
| 7237 | FILHDINSDGVLDEQELE | 0 | 0.017464321 | 0 |
| 7238 | HDINSDGVLDEQELE | 0 | 0.004003101 | 0 |
| 7239 | ILHDINSDGVLDEQELE | 0.00177489 | 0.072004636 | 0 |
| 7240 | LHDINSDGVLDEQELE | 0 | 0.011001017 | 0 |
| 7241 | NPKTFFILHDINSDGVLDEQELE | 0 | 0.001018357 | 0 |
| 7242 | HDINSDGVLDEQE | 0.000455213 | 0.005283989 | 0 |
| 7243 | HDINSDGVLDEQEL | 0 | 0.008580016 | 0 |
| 7244 | ILHDINSDGVLDEQEL | 0.008193079 | 0.181678708 | 0.000601493 |
| 7245 | ILHDINSDGVLDEQELEA | 0 | 0.002001196 | 0 |
| 7246 | LHDINSDGVLDEQEL | 0.000318418 | 0.024996619 | 0.000246817 |
| 7247 | FILHDINSDGVLDEQ | 0 | 0.006751407 | 0.000563986 |
| 7248 | FILHDINSDGVLDEQE | 0.000550012 | 0.009098196 | 0.000418611 |
| 7249 | FILHDINSDGVLDEQEL | 0 | 0.022972014 | 6.27743E-05 |
| 7250 | ILHDINSDGVLDE | 6.62021E-05 | 0.013569229 | 0 |
| 7251 | ILHDINSDGVLDEQ | 0.002631379 | 0.041935267 | 0 |
| 7252 | LHDINSDGVLDE | 0 | 0.001040565 | 0 |
| 7253 | ILHDINSDGVLDEQE | 0.008873954 | 0.144460123 | 0 |
| 7254 | LHDINSDGVLDEQE | 0.000907321 | 0.020135573 | 0.000340761 |
| 7255 | RDNGPDGMEPEGVIE | 0.001079291 | 0 | 0 |
| 7256 | DKDGLLDDEEFALAN | 0 | 0.000647104 | 0 |
| 7257 | VDKDGLLDDEEFALANH | 0 | 0.001875771 | 0 |
| 7258 | IVHNVDSDDLISMG | 0 | 0.001010019 | 0 |
| 7259 | VVHNVNSNELLNM | 0 | 0.001457658 | 0 |
| 7260 | VHGLRNVQAEEMVE | 0.000324661 | 0.001350666 | 0 |
| 7261 | KAGYGAAVVHNVN | 0 | 0 | 0.008342617 |
| 7262 | KAGYGAAVVHNVNSN | 0 | 1.04325E-06 | 0.007244006 |
| 7263 | QKAGYGAAVVHNVN | 0 | 0 | 0.00323023 |
| 7264 | QRAGYKAAIVHNVDSD | 0 | 0 | 0.000421363 |
| 7265 | RAGYKAAIVHNVD | 9.82144E-06 | 0.000205716 | 0.002139918 |
| 7266 | RAGYKAAIVHNVDSD | 0 | 0.000199236 | 0.001776962 |
| 7267 | LTREGETIIELKYR | 0 | 0.001061001 | 0 |
| 7268 | DPQSILLRLGQAQGSL | 0 | 0 | 0.001605558 |
| 7269 | DPQSILLRLGQAQGSLS | 0 | 0 | 0.001560515 |
| 7270 | EIRMAEGEAEGSVK | 0 | 0 | 0.001688841 |
| 7271 | VEIRMAEGEAEGSVK | 0.001849557 | 6.27223E-05 | 0.007092263 |
| 7272 | VEIRMAEGEAEGSVKE | 0 | 0.000297442 | 0.002220838 |
| 7273 | IRKIGEAEAAVIE | 0 | 0 | 0.001044008 |
| 7274 | TPVDAYSNGQAALMG | 0 | 0.001517877 | 0.001650715 |
| 7275 | NPNTVIILIG | 0 | 0 | 0 |
| 7276 | GKSATLTCESDANPPV | 0 | 0.001001367 | 0 |
| 7277 | HPHFLNADPVLAEAVT | 0.001710174 | 0.000142801 | 0 |
| 7278 | GVNPFTNGCCNNVSR | 0 | 0 | 0.000458084 |
| 7279 | YRVYYTMEPEHPVG | 0.000655872 | 2.71929E-05 | 0.00028672 |
| 7280 | AASPRGEPRAPWVEQEGPEYWD | 0.000618055 | 0 | 0 |
| 7281 | AASPRGEPRAPWVEQEGPEYWDRE | 0.006349326 | 0 | 0 |
| 7282 | APWIEQEGPEYW | 0.023478424 | 0.013193369 | 0 |
| 7283 | APWIEQEGPEYWD | 0.042461135 | 0.03331932 | 0 |
| 7284 | EPRAPWIEQEGPEYWD | 0.329160208 | 0.223420517 | 8.86073E-05 |
| 7285 | EPRAPWVEQEGPEYWD | 0.013801178 | 0.000430537 | 0 |
| 7286 | GEPRAPWVEQEGPEYWD | 0.001969064 | 0 | 0 |
| 7287 | RAPWIEQEGPEYWD | 0 | 0.028040077 | 0 |
| 7288 | SPRGEPRAPWVEQEGPEYWD | 0.003658411 | 0.000288166 | 0 |
| 7289 | SPRGEPRAPWVEQEGPEYWDR | 0.005105332 | 8.37841E-05 | 0 |
| 7290 | SPRGEPRAPWVEQEGPEYWDRE | 0.026882055 | 0.01069683 | 0 |
| 7291 | SPRGEPRAPWVEQEGPEYWDRETQ | 0.003060161 | 0.003973266 | 0 |
| 7292 | SPRGEPRAPWVEQEGPEYWDRETQN | 0.00094965 | 0 | 0 |
| 7293 | APWIEQEGPEYWDR | 0.4321974 | 0.150375042 | 0 |
| 7294 | APWIEQEGPEYWDRE | 0.001443842 | 0.207947156 | 0 |
| 7295 | APWIEQEGPEYWDRET | 0.000181591 | 0.025135392 | 0 |
| 7296 | APWIEQEGPEYWDRETQ | 0.002219931 | 0.008944718 | 0 |
| 7297 | APWIEQEGPEYWDRETQISK | 0 | 0.003123939 | 0 |
| 7298 | APWIEQEGPEYWDRN | 0.42789169 | 0.001344505 | 0 |
| 7299 | APWIEQEGPEYWDRNT | 0.011749315 | 0 | 0 |
| 7300 | APWVEQEGPEYWDR | 0.039110839 | 0.05231566 | 0 |
| 7301 | APWVEQEGPEYWDRE | 0.037363958 | 0.061585999 | 0 |
| 7302 | APWVEQEGPEYWDRET | 0.001556709 | 0.007470147 | 0 |
| 7303 | IEQEGPEYWDR | 0.018256599 | 0.013782559 | 0 |
| 7304 | VEQEGPEYWDR | 0.00452619 | 0.010207418 | 0 |
| 7305 | WIEQEGPEYWDR | 0.000137381 | 0.001809928 | 0 |
| 7306 | WVEQEGPEYWDR | 0 | 0.000126768 | 0 |
| 7307 | KEPRAPWIEQEGPEYWDR | 0 | 0.00191349 | 0 |
| 7308 | KEPRAPWIEQEGPEYWDRE | 0 | 0.004833514 | 0 |
| 7309 | KEPRAPWIEQEGPEYWDRET | 0 | 0.008687361 | 0 |
| 7310 | RKEPRAPWIEQEGPEYWDRET | 0 | 0.009654614 | 0 |
| 7311 | SPRKEPRAPWIEQEGPEYWDRET | 0 | 0.142022008 | 0 |
| 7312 | SPRKEPRAPWIEQEGPEYWDRETQ | 0 | 0.019310612 | 0 |
| 7313 | RAPWIEQEGPEYWDRE | 0 | 0.00877774 | 0 |
| 7314 | RAPWIEQEGPEYWDRN | 0.018188797 | 0 | 0 |
| 7315 | RAPWVEQEGPEYWDRE | 0 | 0.000246699 | 0 |
| 7316 | RKEPRAPWIEQEGPEYWDRE | 0 | 0.004555884 | 0 |
| 7317 | SPRKEPRAPWIEQEGPEYWDRE | 0 | 0.13029368 | 0 |
| 7318 | WIEQEGPEYWDRE | 0 | 0.000828062 | 0 |
| 7319 | WIEQEGPEYWDRN | 0.003109062 | 0.000341781 | 0 |
| 7320 | WVEQEGPEYWDRE | 6.08632E-05 | 0.000626062 | 0 |
| 7321 | ASPRGEPRAPWVEQEGPEYWDRET | 0.000317172 | 0 | 0 |
| 7322 | EPRAPWIEQEGPE | 0.000322655 | 0.007404777 | 0 |
| 7323 | EPRAPWIEQEGPEY | 0.004821766 | 0.001518591 | 0 |
| 7324 | EPRAPWIEQEGPEYW | 0.287272274 | 0.093468114 | 0 |
| 7325 | EPRAPWIEQEGPEYWDR | 0.894354805 | 0.244595869 | 0 |
| 7326 | EPRAPWIEQEGPEYWDRE | 0.008179866 | 0.518973708 | 0 |
| 7327 | EPRAPWIEQEGPEYWDRET | 0.000741539 | 0.17922306 | 0 |
| 7328 | EPRAPWIEQEGPEYWDRN | 2.457957119 | 0 | 0.00050616 |
| 7329 | EPRAPWIEQEGPEYWDRNT | 0.156754341 | 0 | 0.000172716 |
| 7330 | EPRAPWVEQEGPEYW | 0.010349876 | 0.000599738 | 0 |
| 7331 | EPRAPWVEQEGPEYWDR | 0.067729525 | 0.021747289 | 8.28883E-05 |
| 7332 | EPRAPWVEQEGPEYWDRE | 0.286881137 | 0.164963061 | 0 |
| 7333 | EPRAPWVEQEGPEYWDRET | 0.048625043 | 0.107096032 | 0 |
| 7334 | GEPRAPWVEQEGPEYWDRET | 0.019317925 | 0.016118698 | 0 |
| 7335 | RGEPRAPWVEQEGPEYWDRET | 0.000433014 | 0.000143912 | 0 |
| 7336 | SPRGEPRAPWVEQEGPEYWDRET | 0.020205787 | 0.032702921 | 0 |
| 7337 | EPRAPWIEQEGPEYWDRETQ | 0 | 0.003669442 | 0 |
| 7338 | EPRAPWVEQEGPEYWDRETQ | 0.001436466 | 0.003223099 | 0 |
| 7339 | GEPRAPWVEQEGPEYWDRETQ | 0.003382662 | 0.001466612 | 0 |
| 7340 | GEPRAPWVEQEGPEYWDRETQKY | 0 | 0.00742071 | 0 |
| 7341 | IEQEGPEYWDRE | 0 | 0.019590306 | 8.09654E-05 |
| 7342 | IEQEGPEYWDRN | 0.018248702 | 0.000114671 | 0 |
| 7343 | IEQEGPEYWDRNT | 0.00071635 | 0 | 0 |
| 7344 | RAPWIEQEGPEYWDRET | 0.010181587 | 0.002397306 | 0 |
| 7345 | RGEPRAPWVEQEGPEYWDRETQKY | 0 | 0.00482528 | 0 |
| 7346 | VEQEGPEYWDRE | 0.001231337 | 0.016708883 | 0 |
| 7347 | VEQEGPEYWDRET | 0 | 0.001708433 | 0 |
| 7348 | GEPRAPWVEQEGPEYW | 0.001642522 | 0 | 0 |
| 7349 | GEPRAPWVEQEGPEYWDR | 0.00740182 | 0 | 0 |
| 7350 | GEPRAPWVEQEGPEYWDRE | 0.022501841 | 0.008069133 | 0 |
| 7351 | GEPRAPWVEQEGPEYWDRETQN | 0.002183769 | 0 | 0 |
| 7352 | GEPRAPWVEQEGPEYWDRETQNY | 0.001487977 | 0 | 0 |
| 7353 | EPRAPWIEQEGPEYWDRNTRK | 0 | 0.006590818 | 0 |
| 7354 | PRAPWIEQEGPEYWDR | 0.001083949 | 0 | 0 |
| 7355 | RAPWIEQEGPEYWDR | 0.010833506 | 0.00492332 | 0.001346187 |
| 7356 | EPRAPWIEQEGPEYWDGE | 0 | 0 | 0.000970862 |
| 7357 | RAPWIEQEGPEYW | 0.003441882 | 0.001857042 | 0 |
| 7358 | AASQKMEPRAPWIEQEGPEYWDQ | 0.001372353 | 0 | 0 |
| 7359 | AASQKMEPRAPWIEQEGPEYWDQE | 0.002601961 | 0 | 0 |
| 7360 | AASQKMEPRAPWIEQEGPEYWDQET | 0.00393371 | 0 | 0 |
| 7361 | APWIEQEGPEYWDQ | 0.077396659 | 0.01282052 | 0 |
| 7362 | EPRAPWIEQEGPEYWDQ | 0.674679038 | 0.053393485 | 0 |
| 7363 | IEQEGPEYWD | 0.002579165 | 0.002766771 | 0 |
| 7364 | IEQEGPEYWDQ | 0.013474578 | 0.004776859 | 0 |
| 7365 | KMEPRAPWIEQEGPEYWDQ | 0.017334451 | 0.002387754 | 0 |
| 7366 | PRAPWIEQEGPEYWDQ | 0.00102478 | 0 | 0 |
| 7367 | QKMEPRAPWIEQEGPEYWDQ | 0.008156651 | 0 | 0 |
| 7368 | RAPWIEQEGPEYWDQ | 0.006310556 | 0.000801372 | 0 |
| 7369 | SQKMEPRAPWIEQEGPEYWDQE | 0.002872157 | 0 | 0 |
| 7370 | APWIEQEGPEYWDQE | 0.154226872 | 0.042412838 | 0 |
| 7371 | APWIEQEGPEYWDQET | 0.015408212 | 0.031981102 | 0 |
| 7372 | APWIEQEGPEYWDQETR | 0.000742117 | 0.000819382 | 0 |
| 7373 | EPRAPWIEQEGPEYWDQETR | 0.016129767 | 0.00047694 | 0 |
| 7374 | EPRAPWIEQEGPEYWDQETRN | 0.036232013 | 0.018773897 | 0 |
| 7375 | EPRAPWIEQEGPEYWDQETRNM | 0.007557768 | 0 | 0 |
| 7376 | IEQEGPEYWDQE | 0.030176278 | 0.02073142 | 0 |
| 7377 | KMEPRAPWIEQEGPEYWDQETRNM | 0.000312975 | 0 | 0 |
| 7378 | MEPRAPWIEQEGPEYWDQETRNM | 0.007066414 | 0 | 0 |
| 7379 | QKMEPRAPWIEQEGPEYWDQETRNM | 0.044510317 | 0 | 0 |
| 7380 | WIEQEGPEYWDQE | 0.001970204 | 0.000420372 | 0 |
| 7381 | RAPWIEQEGPEYWDQET | 0.002376589 | 0.000978443 | 0 |
| 7382 | EPRAPWIEQEGPEYWDQE | 2.110396426 | 0.306457547 | 0 |
| 7383 | EPRAPWIEQEGPEYWDQET | 0.236761943 | 0.210025113 | 0 |
| 7384 | KMEPRAPWIEQEGPEYWDQET | 0.036878173 | 4.78067E-05 | 0 |
| 7385 | KMEPRAPWIEQEGPEYWDQETR | 0.005170728 | 0 | 0 |
| 7386 | KMEPRAPWIEQEGPEYWDQETRN | 0.003715136 | 0 | 0 |
| 7387 | MEPRAPWIEQEGPEYWDQET | 0.014134086 | 0.007123347 | 0 |
| 7388 | PRAPWIEQEGPEYWDQE | 0.004685835 | 0 | 0 |
| 7389 | QKMEPRAPWIEQEGPEYWDQETRN | 0.013666203 | 0 | 0 |
| 7390 | RAPWIEQEGPEYWDEE | 0 | 0 | 0 |
| 7391 | RAPWIEQEGPEYWDQE | 0.024843398 | 0.004208161 | 0 |
| 7392 | MEPRAPWIEQEGPEYWDQE | 0.014763885 | 0 | 0 |
| 7393 | MEPRAPWIEQEGPEYWDQETR | 0.0046169 | 0.002071373 | 0 |
| 7394 | MEPRAPWIEQEGPEYWDQETRN | 0.005316303 | 0 | 0 |
| 7395 | KMEPRAPWIEQEGPEYWD | 0.007004892 | 0 | 0 |
| 7396 | KMEPRAPWIEQEGPEYWDQE | 0.046839363 | 0 | 0 |
| 7397 | MEPRAPWIEQEGPEYW | 0 | 0.000336215 | 0 |
| 7398 | QKMEPRAPWIEQEGPEYWDQE | 0.054066224 | 0 | 0 |
| 7399 | QKMEPRAPWIEQEGPEYWDQET | 0.035497742 | 0 | 0 |
| 7400 | QKMEPRAPWIEQEGPEYWDQETR | 0.014510384 | 0 | 0 |
| 7401 | RMEPRAPWIEQEGPEYWD | 0 | 0.003501861 | 0 |
| 7402 | QRMEPRAPWIEQEGPEYW | 0 | 0.002002881 | 0 |
| 7403 | QRMEPRAPWIEQEGPEYWD | 0 | 0.006316901 | 0 |
| 7404 | QRMEPRAPWIEQEGPEYWDL | 0 | 0.004029221 | 0 |
| 7405 | AASQRMEPRAPWIEQEGPEYWDLQ | 0 | 0.000845016 | 0 |
| 7406 | AASQRMEPRAPWIEQEGPEYWDLQT | 0 | 0.001997739 | 0 |
| 7407 | APWIEQEGPEYWDL | 0 | 0.042821934 | 0 |
| 7408 | APWIEQEGPEYWDLQ | 0.000283884 | 0.131647681 | 0 |
| 7409 | EPRAPWIEQEGPEYWDLQ | 0.000694653 | 0.719607374 | 0 |
| 7410 | IEQEGPEYWDL | 0 | 0.012090931 | 0 |
| 7411 | MEPRAPWIEQEGPEYWDLQ | 0 | 0.00831124 | 0 |
| 7412 | PRAPWIEQEGPEYWDLQ | 0 | 0.002599373 | 0.000289306 |
| 7413 | QRMEPRAPWIEQEGPEYWDLQ | 0 | 0.021388328 | 0 |
| 7414 | RAPWIEQEGPEYWDLQ | 0 | 0.014744693 | 0.000317361 |
| 7415 | RMEPRAPWIEQEGPEYWDLQ | 0 | 0.013038391 | 0 |
| 7416 | SQRMEPRAPWIEQEGPEYWDLQ | 0 | 0.001875671 | 0 |
| 7417 | WIEQEGPEYWDL | 0 | 0.001554537 | 0 |
| 7418 | EPRAPWIEQEGPEYWDL | 0.000285256 | 0.117867165 | 0 |
| 7419 | EPRAPWIEQEGPEYWDLQT | 0 | 0.256886826 | 0 |
| 7420 | EPRAPWIEQEGPEYWDLQTR | 0 | 0.003599893 | 0 |
| 7421 | MEPRAPWIEQEGPEYWDLQTR | 0 | 0.005135484 | 0 |
| 7422 | QRMEPRAPWIEQEGPEYWDLQTR | 0 | 0.004598044 | 0 |
| 7423 | QRMEPRAPWIEQEGPEYWDLQTRN | 0 | 0.010272037 | 0 |
| 7424 | RAPWIEQEGPEYWDL | 0.002256037 | 0.002256558 | 0 |
| 7425 | WIEQEGPEYWD | 0 | 0.000478443 | 0 |
| 7426 | MEPRAPWIEQEGPEYWDLQT | 0 | 0.018539307 | 0 |
| 7427 | QRMEPRAPWIEQEGPEYWDLQT | 0 | 0.068752006 | 0 |
| 7428 | RAPWIEQEGPEYWDLQT | 0 | 0.000149869 | 0 |
| 7429 | RMEPRAPWIEQEGPEYWDLQT | 0 | 0.013744748 | 0 |
| 7430 | SQRMEPRAPWIEQEGPEYWDLQT | 0 | 0.00272423 | 0 |
| 7431 | APWIEQEGPEYWDLQT | 0.000113886 | 0.014132633 | 0 |
| 7432 | APWIEQEGPEYWDLQTR | 0 | 0.001387743 | 0 |
| 7433 | APWIEQEGPEYWDLQTRN | 0 | 0.001057333 | 0 |
| 7434 | EPRAPWIEQEGPEYWDLQTRN | 0 | 0.005352318 | 0 |
| 7435 | EPRAPWIEQEGPEYWDLQTRNV | 0 | 0.008430082 | 0 |
| 7436 | IEQEGPEYWDLQ | 0 | 0.032479964 | 0.000100081 |
| 7437 | MEPRAPWIEQEGPEYWDLQTRNV | 0 | 0.008359146 | 0 |
| 7438 | QRMEPRAPWIEQEGPEYWDLQTRNV | 0 | 0.071805794 | 0 |
| 7439 | RMEPRAPWIEQEGPEYWDLQTRNV | 0.136787143 | 0.149535788 | 0 |
| 7440 | WIEQEGPEYWDLQ | 0 | 0.005270813 | 0 |
| 7441 | SPRKEPRAPWIEQEGPEYW | 0 | 0.005541327 | 0 |
| 7442 | SPRKEPRAPWIEQEGPEYWD | 0 | 0.014686547 | 0 |
| 7443 | SPRKEPRAPWIEQEGPEYWDR | 0 | 0.064799913 | 0 |
| 7444 | WIEQEGPEYWDRETQISK | 0 | 0.002314283 | 0 |
[truncated: 284,369 more chars]
